# Copper-catalyzed asymmetric hydrogenation of 2-substituted ketones *via* dynamic kinetic resolution†
†Electronic supplementary information (ESI) available. CCDC 1816601 and 1816602. For ESI and crystallographic data in CIF or other electronic format see DOI: 10.1039/c8sc00434j


**DOI:** 10.1039/c8sc00434j

**Published:** 2018-04-23

**Authors:** Olga V. Zatolochnaya, Sonia Rodríguez, Yongda Zhang, Kendricks S. Lao, Sergei Tcyrulnikov, Guisheng Li, Xiao-Jun Wang, Bo Qu, Soumik Biswas, Hari P. R. Mangunuru, Daniel Rivalti, Joshua D. Sieber, Jean-Nicolas Desrosiers, Joyce C. Leung, Nelu Grinberg, Heewon Lee, Nizar Haddad, Nathan K. Yee, Jinhua J. Song, Marisa C. Kozlowski, Chris H. Senanayake

**Affiliations:** a Chemical Development , Boehringer Ingelheim Pharmaceuticals, Inc. , 900 Old Ridgebury Road , Ridgefield , CT 06877 , USA . Email: yongda.zhang@boehringer-ingelheim.com ; Email: sonrodrod@hotmail.com; b Department of Chemistry , University of Pennsylvania , Philadelphia , PA 19104 , USA . Email: marisa@sas.upenn.edu

## Abstract

A new class of tunable heterophosphole dimeric ligands have been designed and synthesized. These ligands have enabled the first examples of Cu-catalyzed hydrogenation of 2-substituted-1-tetralones and related heteroaryl ketones *via* dynamic kinetic resolution, simultaneously creating two contiguous stereogenic centers with up to >99 : 1 dr and 98 : 2 er. The ligand-Cu complexes were isolated and characterized by single crystal X-ray, and DFT calculations revealed a novel heteroligated dimeric copper hydride transition state.

## Abstract 2

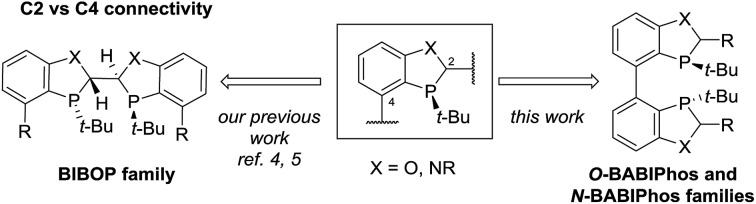
A new class of tunable heterophosphole dimeric ligands have been designed and synthesized.

## 

Transition metals such as Pd, Pt, Rh, Ru and Ir have been widely utilized for a large variety of catalytic transformations.^1^ However, the long-term sustainability of such processes has become an increasing concern due to the high cost, limited abundance and observed toxicity of noble metals.^2^ To overcome these issues, in recent years, there has been a major endeavor in the field to explore new synthetic methods utilizing non-precious metal catalysis.^3^ To this end, we have recently reported several novel methodologies employing Fe, Ni, Co and Cu-centered catalysts.^4^ For example, our laboratories have developed the first Cu-catalyzed asymmetric propargylation reaction of aldehydes4*a* whereas the key to obtaining high enantioselectivities was the use of our bidentate MeO-BIBOP ligand to control the stereochemical course of the reaction. MeO-BIBOP belongs to a family of P-chiral benzooxaphosphole (BOP) ligands which have demonstrated superior abilities to catalyze a range of asymmetric transformations.^5^ The modular design of the ligand core allows for high tunability of steric and electronic properties of the ligand to afford the desired reactivities and stereoselectivities (Scheme 1). Encouraged by our success with the BIBOP-Cu system, we aspired to design new bidentate benzooxaphosphole ligands to promote other non-precious metal catalyzed fundamental reactions such as asymmetric hydrogenation of ketones. Herein we report the discovery of a new class of heterophosphole dimeric ligands (BABIPhos) and their application in the first copper-catalyzed asymmetric hydrogenation of 2-substituted-1-tetralones and related heteroaryl ketones *via* dynamic kinetic resolution.

**Scheme 1:**
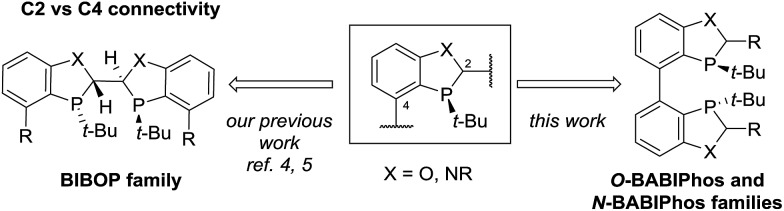
Dimeric benzoheterophosphole ligands.

The Cu-catalyzed asymmetric reduction of ketones has been described in the literature using both silanes and hydrogen.^6^ The silane-based methods often require cryogenic temperatures and generate stoichiometric amounts of silane byproducts rendering them unattractive for scaleup.^7^ On the other hand, greener alternatives based on catalytic hydrogenation are more challenging and significantly less developed. Shimizu and co-workers reported the first asymmetric Cu-catalyzed ketone hydrogenation using BDPP as ligand obtaining good selectivities with *ortho*-substituted aryl and heteroaryl ketones.^8^ Beller's group described a more general system using monodentate binaphthophosphepine ligands that provided selectivities up to 77% ee.^9^ More recently, Hatcher and co-workers reported the use of modified BoPhoz ligands for ketone hydrogenation to afford optically enriched alcohol products with 75 : 25 to 98 : 2 er.^10^ As part of our long-standing interest in non-precious metal catalysis and engineering innovative catalytic systems, we sought to develop more efficient methods for Cu-catalyzed asymmetric ketone hydrogenation.

2-Aryl-1-tetralone derivatives were chosen as initial substrates for the study because the resulting chiral alcohols were required for one of our ongoing programs. Furthermore the relatively low p*K*_a_ of the keto-benzylic position offers an opportunity for dynamic kinetic resolution (DKR), creating two adjacent stereocenters in one single operation. It should be noted that no examples of Cu-catalyzed asymmetric reduction of ketones *via* DKR have been previously reported.^11^

The asymmetric hydrogenation of racemic 2-phenyl-1-tetralone **4a** was first evaluated using protocols reported by Shimizu,^8^ Beller^9^ and Hatcher^10^ (Table 1). Low conversions were observed under Shimizu's BDPP and Beller's CatASium KPh conditions. Hatcher's modified BoPhoz ligand provided 70% conversion after 24 h at 400 psi with moderate stereoselectivity of 88 : 12 dr and 83 : 17 er for the major diastereoisomer. Evaluation of chiral BIBOP ligand provided full conversion exclusively to the *cis*-diastereomer with 77 : 23 er, while the reaction with MeO-BIBOP gave less gratifying results. Phenyl and cyclohexyl BIBOP analogs exhibited no reactivity in this reaction. Despite the observed modest stereoselectivities in the initial screening, these results clearly showed that the anticipated DKR has occurred under the reaction conditions.

**Table 1:** Cu-Catalyzed Asymmetric Hydrogenation of 2-Phenyl-1-tetralone^*a*^

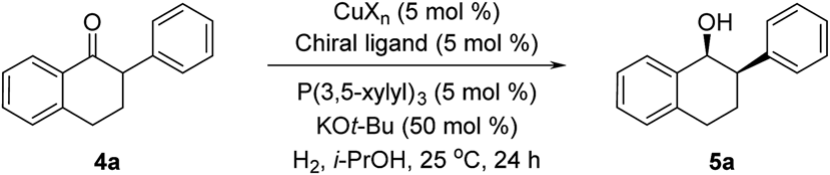
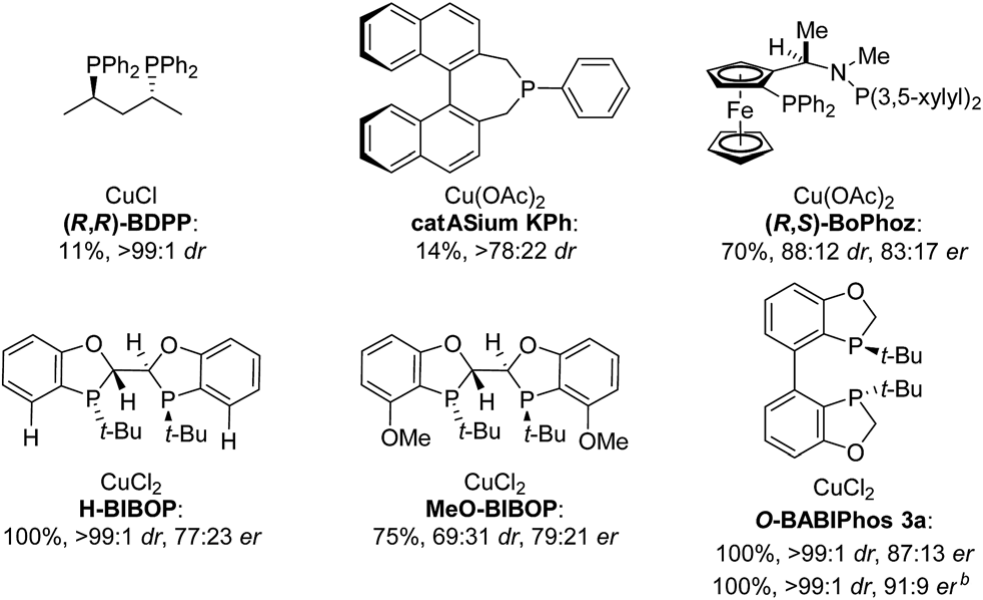

^*a*^Conversions were determined by comparison of relative HPLC integration of alcohol to ketone at 220 nm, diastereo- and enantiomeric ratios (dr and er) were determined by chiral HPLC.

^*b*^ *t-*AmOH as solvent.

To further improve the reactivity and selectivity of the reaction, we decided to synthesize the alternative BOP dimer *via* the C4-coupling which might provide new ligands with different properties (Scheme 1).^12^ Our quest for this type of biaryl ligands began by exploring the reductive homocoupling of the corresponding benzooxaphosphole oxide triflate **1a** (Scheme 2). After extensive investigation of the Pd-13 and Ni-catalyzed^14^ protocols for dimerizing aryl halides and triflates,^15^ it was found that under Pd-catalysis in the presence of BI-DIME, aryl triflate **1a** was converted to the corresponding biaryl **2a** in 84% yield. Additionally, the oxaphosphole ring of **2a** was alkylated to form C2, C2′-substituted oxide analogs **2c–e**. The phosphine oxides were subsequently reduced to the corresponding phosphines **3a,c–e** (*O*-BABIPhos) giving access to a new class of bidentate ligands. This route was demonstrated on several hundred grams scale.

**Scheme 2:**
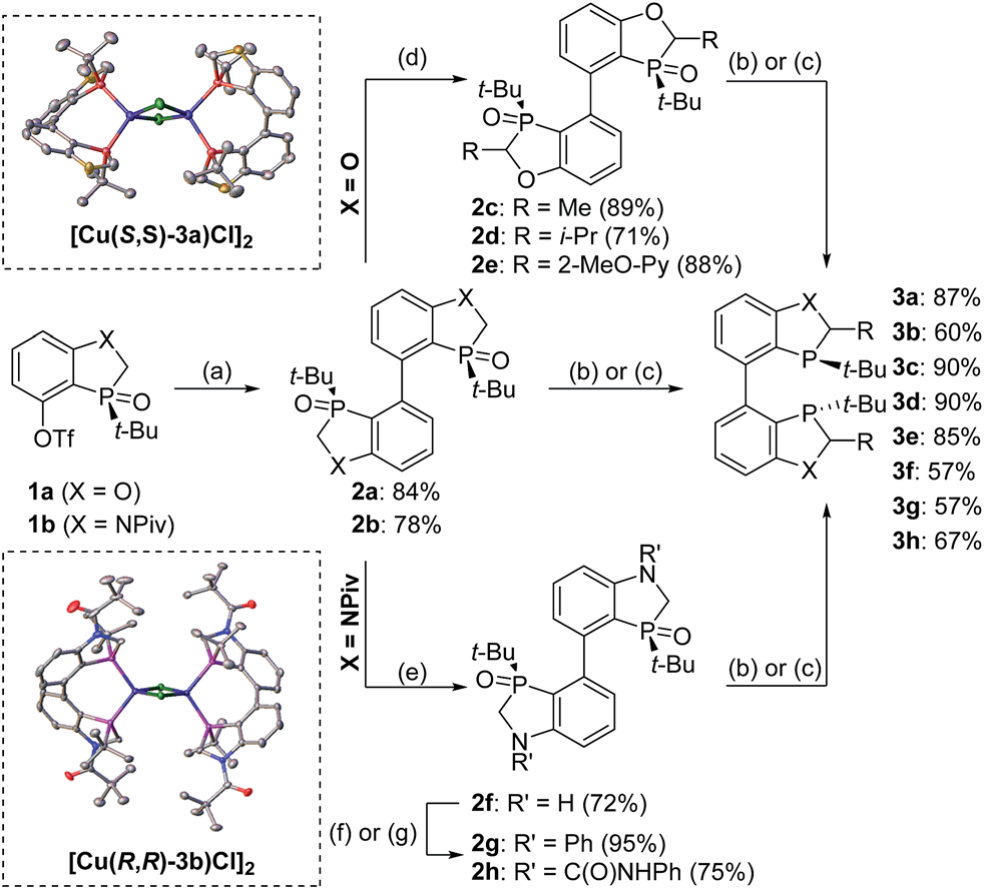
Synthesis of *O*-BABIPhos and *N*-BABIPhos. Conditions: (a) **2a** (1 equiv.), Pd_2_(dba)_3_ (2.5 mol%), BI-DIME (5 mol%, **2a**) or DTBPF (5 mol%, **2b**), Et_4_NI (2 equiv.), Zn dust (3 equiv.), DMAc, 140 °C; (b) (Me_2_HSi)_2_O (3 equiv.), Ti(Oi-Pr)_4_ (2.3 equiv.), THF, 65 °C; (c) Et_3_SiH (3 equiv.), Et_3_N (3 equiv.), toluene, 110 °C; (d) LDA (2.5 equiv.); then RX (2.5 equiv.; **2c**: MeI, **2d**: i-PrI, **2e**: 2-methoxy-6-(phenylsulfonyl)pyridine), THF, –78 °C to r.t.; (e) NaOH, MeOH/H_2_O, 50 °C; (f) **2g**: PhBr, Pd_2_(dba)_3_ (5 mol%), RuPhos (20 mol%), NaO*t-*Bu (2.4 equiv.), THF, reflux; (g) **2h**: PhNHCO (10 equiv.), i*-*Pr_2_NEt (3.6 equiv.), THF, reflux. BI-DIME = 3-(*tert*-butyl)-4-(2,6-dimethoxyphenyl)-2,3-dihydrobenzo[*d*] [1,3]oxaphosphole, DTBPF = 1,1′-bis(di-*tert*-butylphosphino)ferrocene, LDA = lithium diisopropylamide, RuPhos = 2-dicyclohexylphosphino-2′,6′-diisopropoxybiphenyl.

With these ligands in hand, the ketone hydrogenation was carried out, and we were pleased to find that the reaction with *O*-BABIPhos ligand **3a** provided high reactivities and selectivities up to 91 : 9 er of single diastereoisomer **5a** (Table 1). However, modification of the R group in *O*-BABIPhos did not further improve the enantioselectivity. To obtain more selective ligands, a new class of dimeric azaphosphole ligands, *N*-BABIPhos were designed and prepared to further modulate the electronic properties of the ligand by introducing various functionalities on the nitrogen atom (Scheme 2). Dimerization of the nitrogen-containing triflate **1b** proved to be much more challenging and a wide-scope ligand screen led to the identification of DTBPF as the best ligand that gave good yield of biaryl product **2b**. Further modification at nitrogen gave quick access to *N*-BABIPhos analogs **3f–h**.

We were delighted to find that the Cu-catalyzed hydrogenation of 2-phenyl-1-tetralone in *t*-AmOH using *N*-BABIPhos **3b** as the ligand proceeded in complete diastereoselectivity and 97 : 3 er (Table 2, entry 1). Reactions with other azaphospholes bearing different *N*-substitutions gave overall less satisfactory results (entries 2–4). *t*-AmOH proved to be the solvent of choice for this transformation. Lower reactivity was observed when performing the reactions in *t*-BuOH as solvent while i*-*PrOH provided good reactivity but only 92 : 8 er (entries 5–6)^16^. The nature of ancillary phosphine played a key role in the reactivity of our new catalytic system. Thus, no reaction was observed in the absence of ancillary phosphine or using electron-deficient triarylphosphines (entries 7–8). The source of this phenomenon was further elucidated *via* DFT calculations (*vide infra*).

**Table 2:** Optimization of reaction conditions^*a*^

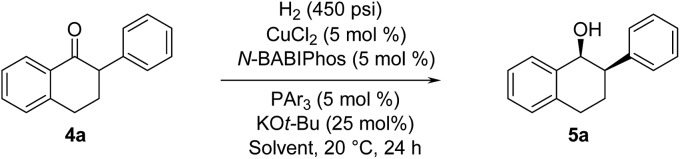					
Entry	Ligand	PAr_3_	%**5a**^*b*^	dr^*c*^	er^*c*^
1	**3b**	P(3,5-xylyl)_3_	100	>99 : 1	97 : 3
2	**3f**	P(3,5-xylyl)_3_	50	>99 : 1	96 : 4
3	**3g**	P(3,5-xylyl)_3_	100	>99 : 1	86 : 14
4	**3h**	P(3,5-xylyl)_3_	50	>99 : 1	94 : 6
5^*d*^	**3b**	P(3,5-xylyl)_3_	66	>99 : 1	96 : 8
6^*e*^	**3b**	P(3,5-xylyl)_3_	100	>99 : 1	92 : 8
7	**3b**	—	0	—	—
8	**3b**	P(3,5-CF_3_-Ph)_3_	1	—	—

^*a*^Conditions: H_2_ (450 psi), CuCl_2_ (5 mol%), *N*-BABIPhos (5 mol%), PAr_3_ (5 mol%), KO*t*-Bu (25 mol%), *t*-AmOH, 20 °C.

^*b*^Determined by comparison of relative HPLC integration of alcohol to ketone at 220 nm.

^*c*^Determined by chiral HPLC.

^*d*^ *t*-BuOH as solvent.

^*e*^i-PrOH as solvent.

Both the Cu–*O*-BABIPhos and Cu–*N*-BABIPhos complexes were obtained in quantitative yields by treatment of CuCl with a stoichiometric amount of respective ligand in dichloromethane at reflux temperature for 2 h. The molecular structures of the complexes were unambiguously characterized by single crystal X-ray crystallography^17^ revealing a chloride-bridged dicopper complex such as [Cu(μ-Cl) (κ_2_-*N*-BABIPhos)]_2_ with a distorted tetrahedral geometry at both copper(i) centers, as observed for similar Cu complexes in the literature (Scheme 2).^18^

The scope of the reaction was next examined (Table 3). A variety of racemic 2-aryl-1-tetralones were smoothly reduced under our standard conditions with excellent enantioselectivities (96 : 4 to 98 : 2 er, products **5a–h**). Reactions with heterocycle-containing substrates were equally effective. Pyridine, furan, thiophene and pyrazole-fused cyclohexanones underwent the Cu-catalyzed hydrogenation in high stereoselectivities (96 : 4 to 97 : 3 er, products **5i–l**). Furthermore, the hydrogenative DKR of 2-alkyl-1-tetralones **4m** and **4n** proved to be highly enantioselective with good diastereoselectivities. Substituted indanone substrate **4o** was reduced under the reaction conditions, albeit with a more modest er (76 : 24). Finally, hydrogenation of a 1-cyclohexyl-2-tetralone (**4p**) was also successful furnishing the desired product in synthetically useful enantioselectivity (84 : 16).

**Table 3:** Scope of Cu-catalyzed asymmetric hydrogenation *via* DKR^*a*^

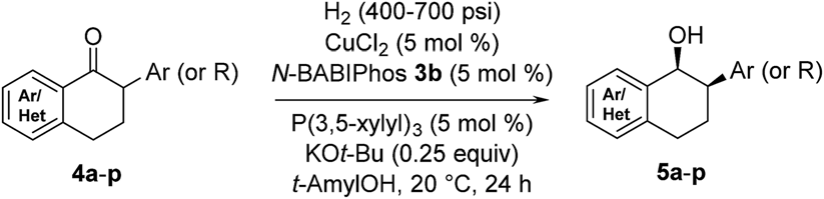
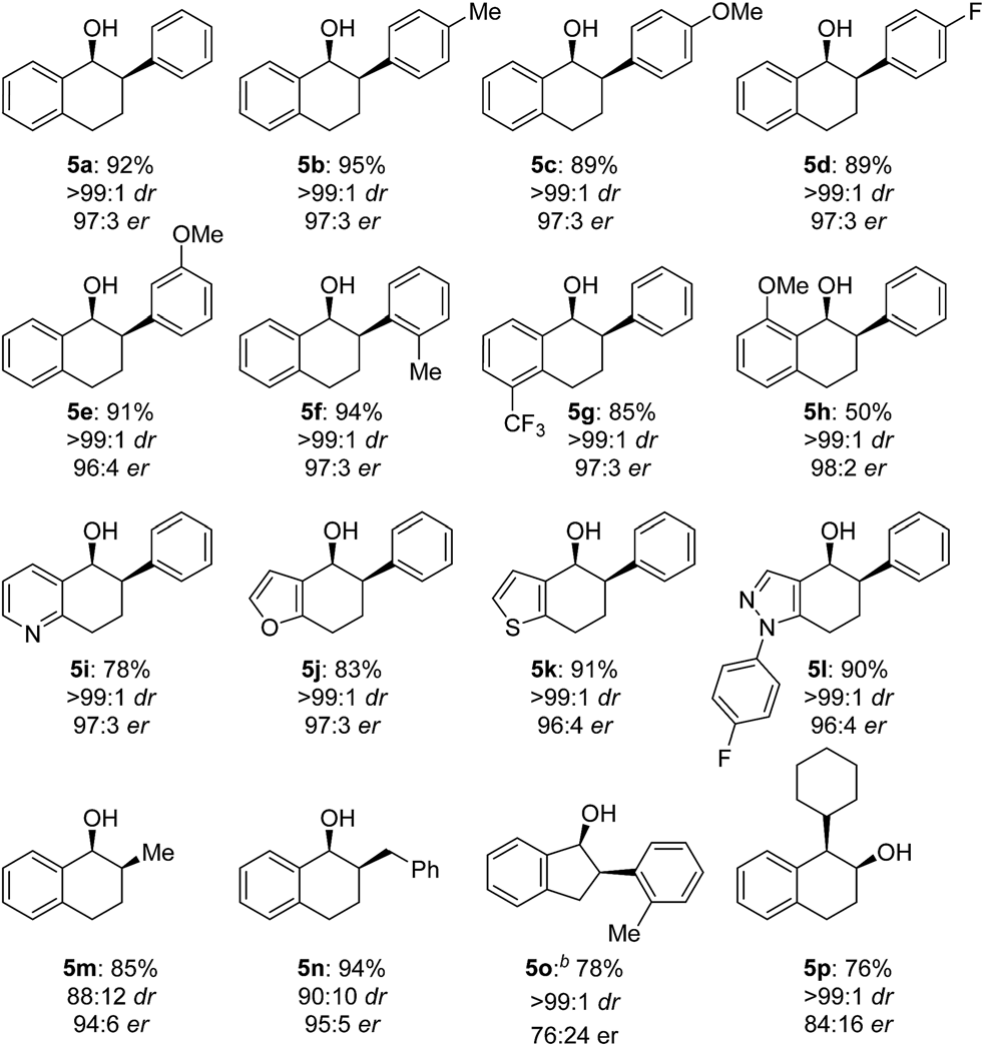

^*a*^Isolated yields; diastereomeric and enantiomeric ratios (dr and er) were determined by chiral HPLC.

^*b*^i-PrOH as solvent.

The resulting optically enriched tetralols are valuable building blocks for synthesis of bioactive molecules. For example, compound **5q** (>99 : 1 dr, 97 : 3 er from asymmetric hydrogenation of **4q**, Scheme 3) can be readily deoxygenated to 2-aryltetralin **6** (97 : 3 er) which is a key precursor to pharmaceutically important compounds for treatment of arrhythmias by inhibiting the Na^+^/Ca^2+^ exchange mechanism.^19^ The only known asymmetric route to such 2-aryltetralins required the use of Pd-catalyzed arylation of 1-tetralone employing an equimolar amount of highly toxic tributyltin methoxide.^20^

**Scheme 3:**
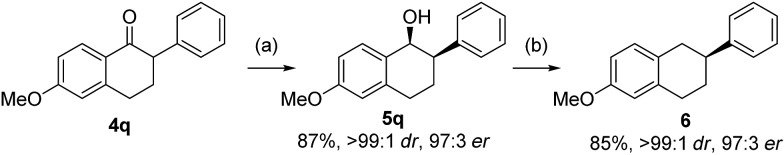
Synthesis of 7-methoxy-2-phenyl tetralin. Conditions: (a) H_2_ (700 psi), CuCl_2_ (5 mol%), **3b** (5 mol%), P(3,5-xylyl)_3_ (5 mol%), KO*t*-Bu (0.25 equiv.), *t*-AmOH, 20 °C, 24 h; (b) Et_3_SiH (8 equiv.), TFA (4 equiv.), CH_2_Cl_2_, 0 °C.

To understand the observed mixed ligand effect and to facilitate further development of the efficient catalysts, we initiated DFT study of the mechanism of this transformation. The reaction is believed to proceed *via in situ* formation of copper hydrides, where multiple species could be acting as the hydride source (Scheme 4).^21^

**Scheme 4:**
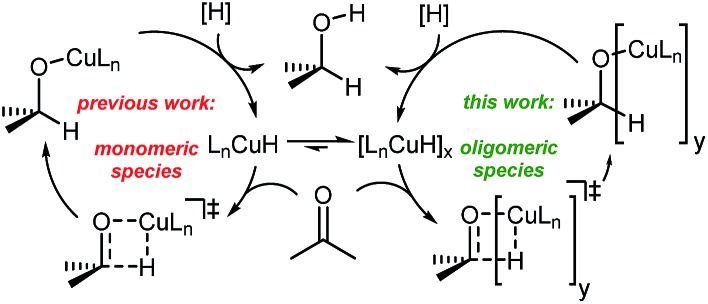
Generic catalytic cycle for the Cu-catalyzed ketone reduction.

Copper hydrides are known to exist in oligomeric form, with the exact nature of the oligomer strongly depending on the identity of the ligand and the reaction conditions^22^. Thus, for the LCuH species, where L is a phosphine, there is evidence for existence of the corresponding dimers^23^, trimers^24^, pentamers^25^, hexamers^26^ and octamers^27^. For biaryl bidentate ligands, analogous to our ligand of interest, only a SEGPHOS-based system^28^ has been examined^29^; NMR and MS experiments did not provide any conclusive results about the exact nature of this LCuH species, but suggested the presence of oligomers. Kinetic studies also pointed to the oligomeric character of the copper hydride. Previously published computational studies support the relative instability of the monomer^30^. Given the well-known propensity of the LCuH species to form less reactive oligomers, the greater reactivity observed here when two different ligands are employed [bidentate L and monodentate P(3,5-xylyl)_3_] may arise from the formation of different aggregates. Specifically, we theorize that the homoaggregates [L alone or P(3,5-xylyl)_3_ alone] are less reactive than mixed aggregates incorporating the two different ligands.

DFT analysis of some model higher aggregates demonstrated that this is not the case, and the heteroligated aggregates average the stabilities of the homoligated systems (Scheme 5). This finding led us to propose the involvement of transition states incorporating two Cu atoms, where different ligands can be coordinated to each Cu, to explain the mixed ligand effect. To the best of our knowledge, such transition states incorporating dimer Cu species had not been previously calculated for this type of reaction.

**Scheme 5:**
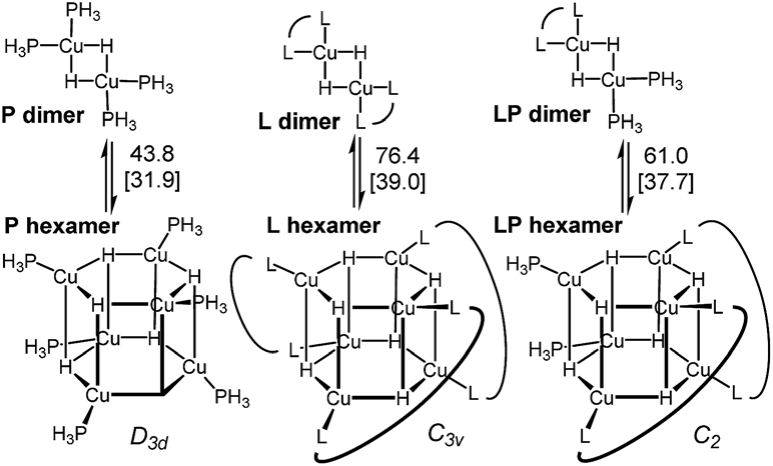
Energy of the model dimers relative to the corresponding hexameric CuH aggregates. Ligands are truncated (see ESI† for details).^31^

In analyzing hydride transfer transition states for the reduction of substituted tetralone, transition states incorporating two copper centers were lower than those incorporating one copper center for both monodentate and bidentate ligands (Scheme 6).

**Scheme 6:**
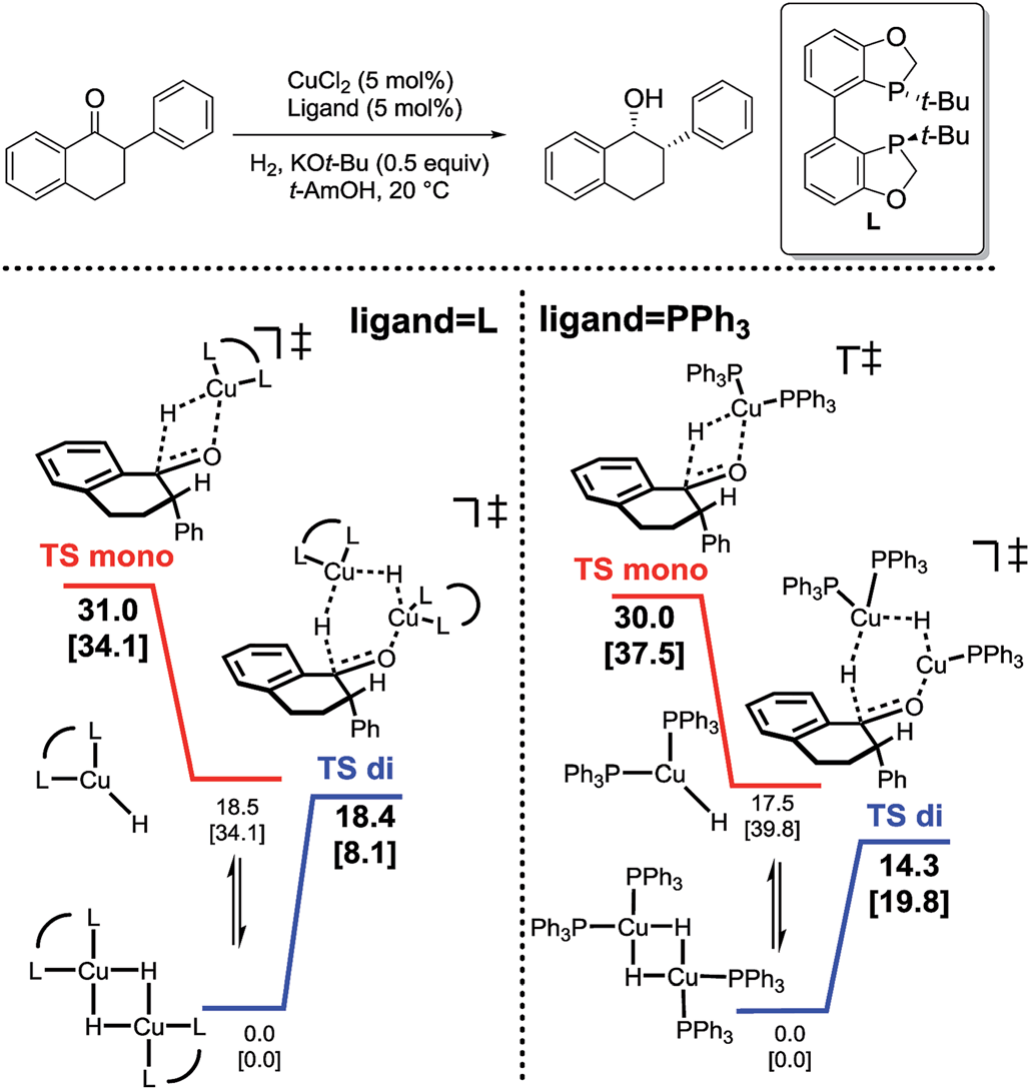
Relative energetics of monomeric (red) and dimeric (blue) hydride transfer pathways for bidentate (left) and monodentate (right) ligands.^31^

Following discovery of this lower energy reaction pathway, we analyzed the relative energetics of the most stable homo- and hetero-ligated dimeric transition states (Scheme 7).

**Scheme 7:**
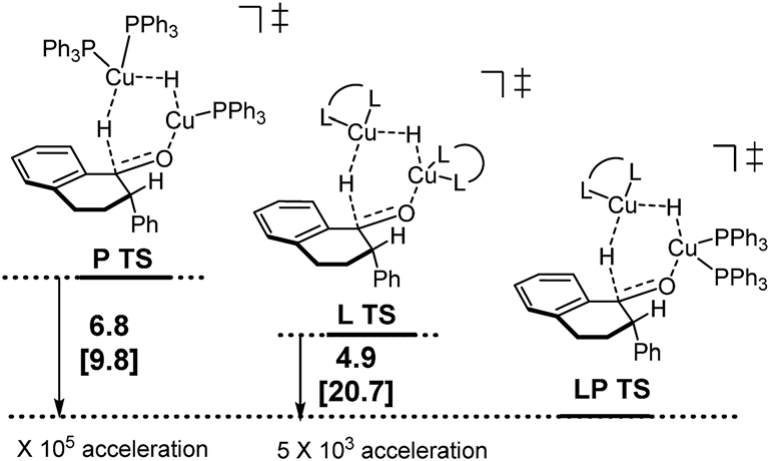
Relative energetics of the homoligated (leftmost and center) and mixed dimeric hydride transfer transition states.

These results indicate that the heteroligated dimeric transition state is lower in energy than the monoligated dimeric transition states, providing more than 1000-fold acceleration of the hydride transfer. Interestingly, the stabilization of the heteroligated dimeric transition state is not a purely entropic phenomenon. That is, P TS and LP TS differ in energy, even though both have the same molecularity. We hypothesize that the mixed ligand environment better supports the charge distribution required for the effective hydride transfer (Scheme 8). Specifically, one Cu center engages in Lewis acid activation of the carbonyl, for which an electron-deficient L2 would be superior. And, the second copper center delivers nucleophilic hydride, which is assisted by an electron-rich L1.

**Scheme 8:**
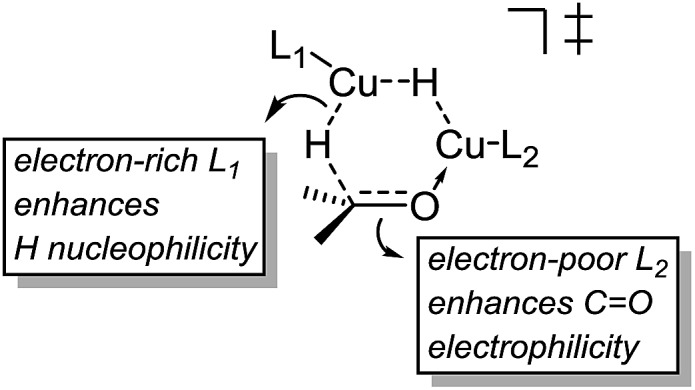
Favorable electronics for the dimeric hydride transfer transition state.

In summary, a new class of heterophosphole dimeric ligands (*O*-BABIPhos and *N*-BABIPhos) have been designed and synthesized. These ligands are highly modular and tunable and have enabled the first example of Cu-catalyzed hydrogenation of 2-substituted-1-tetralones and related heteroaryl ketones *via* dynamic kinetic resolution, simultaneously establishing two contiguous stereogenic centers with up to >99 : 1 dr and 98 : 2 er. The ligand–Cu complexes were isolated and characterized by single crystal X-ray, and DFT calculations revealed a novel heteroligated dimeric copper hydride transition state. Application of this new series of oxa- and azaphosphole bisphosphine ligands to other important catalytic reactions is currently under investigation and will be disclosed in due course.

## Conflicts of interest

The authors declare no conflict of interest.

## Supplementary Material

Supplementary information

Crystal structure data
