# Supplementary material for: Copper-catalyzed asymmetric hydrogenation of 2-substituted ketones via dynamic kinetic resolution
Source: Chem Sci. 2018 Apr 23;9(19):4505–10. doi: 10.1039/c8sc00434j (PMC5958347; doi:10.1039/c8sc00434j)
Supplement: Supplementary file 1 [file SC-009-C8SC00434J-s001.pdf]

Electronic Supplementary Material (ESI) for Chemical Science.
This journal is © The Royal Society of Chemistry 2018

Supporting Information for

Copper-Catalyzed Asymmetric Hydrogenation of 2-Substituted Ketones via Dynamic Kinetic Resolution

Olga V. Zatolochnaya, Sonia Rodríguez,* Yongda Zhang,* Kendricks S. Lao, Sergei Tcyrulnikov,† Guisheng Li, Xiao-Jun Wang, Bo Qu, Soumik Biswas, Hari P. R. Mangunuru, Daniel Rivalti, Joshua D. Sieber, Jean-Nicolas Desrosiers, Joyce C. Leung, Nelu Grinberg, Heewon Lee, Nizar Haddad, Nathan K. Yee, Jinhua J. Song, Marisa C. Kozlowski,*† Chris H. Senanayake

Chemical Development US, Boehringer Ingelheim Pharmaceuticals, Inc.

900 Ridgebury Rd., Ridgefield, CT 06877 (US)

†Department of Chemistry, University of Pennsylvania, Philadelphia, PA 19104, USA

Table of Contents

1. General remarks---2
2. Preparation of ligands ---3
3. Dynamic kinetic resolution of α-substituted tetralones---16
   3.1 Synthesis of α-aryl tetralone derivatives---16
   3.2 Screening of reaction parameters for reduction of 2-phenyl-1-tetralone---------------------19
   3.3 Dynamic kinetic resolution of α-substituted tetralones---24
4. Details of computational studies--- 31
5. NMR and HPLC data ---54

1 General remarks

All NMR spectra were collected on Bruker Avance spectrometers equipped with a 5 mm BBI probe (^{1}H, ^{13}C, ^{31}P) each with z-gradient, at 30 ^{o}C, unless otherwise indicated. ^{1}H and ^{13}C chemical shifts were calibrated vs. the deuterated solvent used. ^{31}P NMR chemical shifts were calibrated vs. external 85% H_3PO_4 (δ 0.0 ppm) contained in coaxial insert tubes (Wilmad WGS-5BL). MS was measured on Agilent 1100 Series LC/MSD mass spectrometer. Accurate mass measurements were performed on a Time of Flight mass spectrometer (LC/MSD TOF) operating in a positive electrospray ionization mode with the capillary voltage of 3 kV. The mass spectrometer was tuned and calibrated using a tuning mix prior to sample analysis. Samples were introduced to the mass spectrometer by flow injection using an HPLC system. Solvents and reagents were used as received without additional purification unless otherwise indicated.

2 Preparation of ligands[1]

Table S1. Survey of known Pd-catalyzed methods for homocoupling of triflate **1a**:

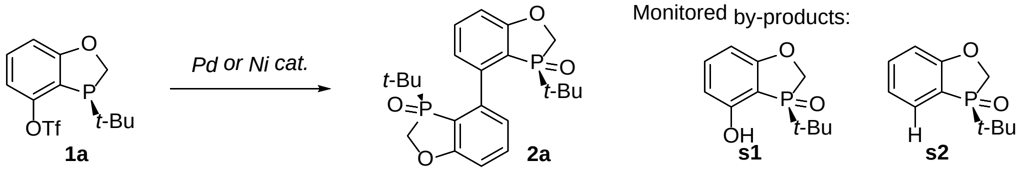

Entry	Conditions	% **1a**[a]	% **2a**[a]	% **s1**[a]	% **s2**[a]
1[2]	$Pd(OAc)_2$ (5 mol %), (*o*-Tol_3)P (5 mol %), hydroquinone (0.5 equiv), Cs_2CO_3 (1 equiv), DMAc, 140 °C	0	0	100	0
2[3]	$PdCl_2$dppf (10 mol %), CsF (7.5 equiv), DMSO, 140 °C	0	0	100	0
3[4]	$PdCl_2(PhCN)_2$ (5 mol %), TDAE (2 equiv), DMF, 80 °C	63	0	36	1
4[5]	$PdCl_2$dppf (4 mol %), B_2Pin_2 (0.5 equiv), K_2CO_3 (3 equiv), dioxane, 80 °C	30	0	70	0

[a]Relative HPLC integration.

[1] Li, G.; Zatolochnaya O. V.; Wang, X.-J.; Rodríguez S.; Qu, B; Desrosiers, J.-N.; Mangunuru H. P. R.; Biswas, S.; Rivalti, D.; Karyakarte, S. D.; Sieber, J. D.; Grinberg, N.; Wu, L.; Lee H.; Haddad N.; Fandrick, D. R.; Yee, N. K.; Song, J. J. and Senanayake, C. H. *Org. Lett.* 2018 ASAP. **DOI:** 10.1021/acs.orglett.8b00139
[2] Hennings, D. D.; Iwama, T.; Rawal, V. H. *Org. Lett.* **1999**, *1*, 1205.
[3] Qi, C.; Sun, X.; Lu, C.; Yang, J.; Du, Y.; Wu, H.; Zhang, X.-M. *J. Organomet. Chem.* **2009**, *694*, 2912.
[4] (a) Kuroboshi, M.; Waki, Y.; Tanaka, H. *Synlett* **2002**, *4*, 637. (b) Kuroboshi, M.; Waki, Y.; Tanaka, H. *J. Org. Chem.* **2003**, *68*, 3938.
[5] Nising, C. F.; Schmid,, U. K.; Nieger, M.; Bräse, S. *J. Org. Chem.* **2004**, *69*, 6830.

Table S2. Screening of ligands for Pd-catalyzed reductive homocoupling of triflate **1a**:[6]

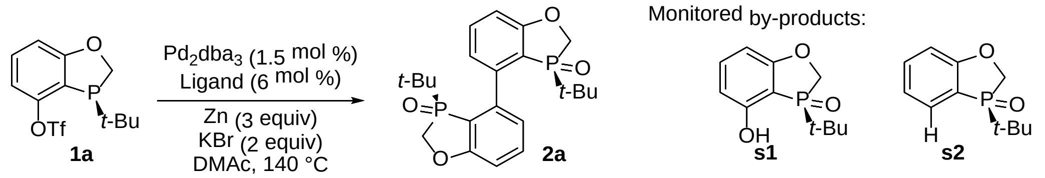

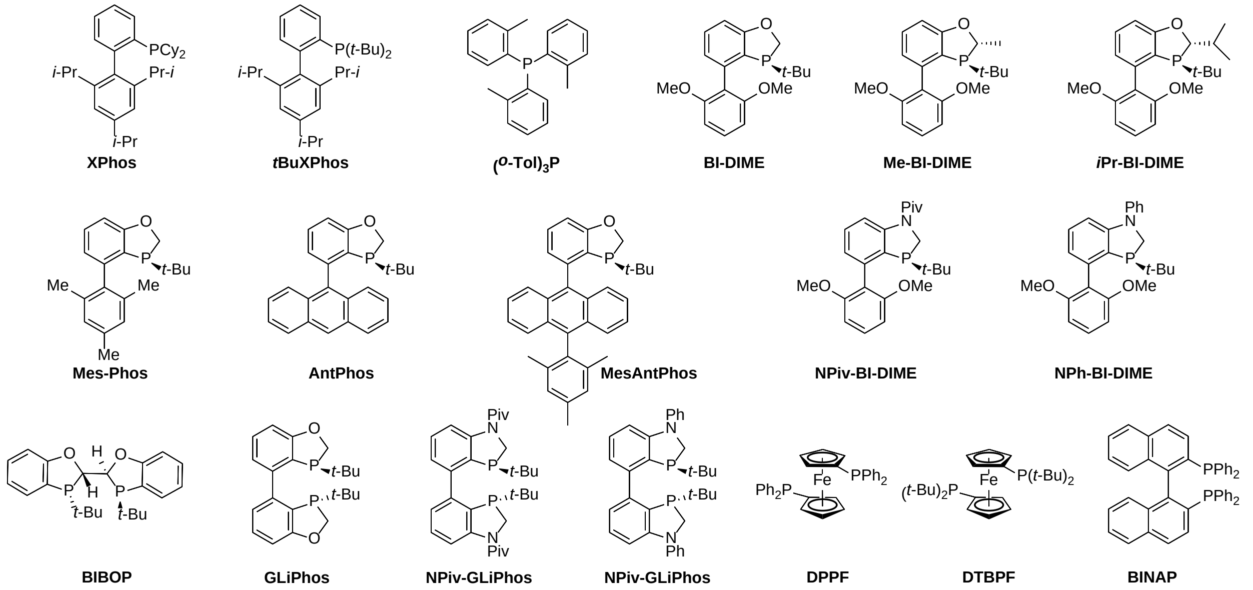

Entry	Ligand	% **1a**[a]	% **2a**[a]	% **s1**[a]	% **s2**[a]
1	XPhos	0	81	9	8
2	*t*-BuXPhos	56	5	34	5
3	*o*-Tol_3P	41	40	15	4
4	BI-DIME	0	83	10	7
5	Me-BI-DIME	6	78	8	8
6	*i*Pr-BI-DIME	47	30	19	4
7	Mes-Phos	11	74	7	8
8	AntPhos	32	45	14	9
9	MesAntPhos	38	47	7	8
10	NPiv-BI-DIME	32	53	8	7
11	NPh-BI-DIME	29	58	6	7
12	BIBOP	53	2	24	21
13	BABIPhos	34	0	33	33
14	NPiv-BABIPhos	39	14	32	15

[6] Based on: Jutand, A.; Mosleh, A. *J. Org. Chem.* **1997,** *62*, 261.

15	NPh-BABIPhos	50	19	21	10
16	DPPF	68	0	27	5
17	DTBPF	0	92	1	7
18	BINAP	56	1	27	16

[a]Relative HPLC integration.

Table S3. Screening of additives for Pd-catalyzed reductive homocoupling of triflate **1a**:

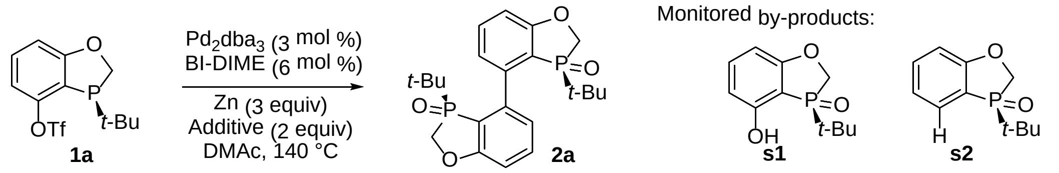

Entry	Additive	% **SM**[a]	% **P**[a]	% **OH**[a]	% **H**[a]
1	KBr	0	83	10	7
2	*n*-Bu_4NBr	0	49	27	24
3	Et_4NI	0	90	4	5
4	none	80	7	10	3

[a]Relative HPLC integration.

Synthesis of ligand 3a:

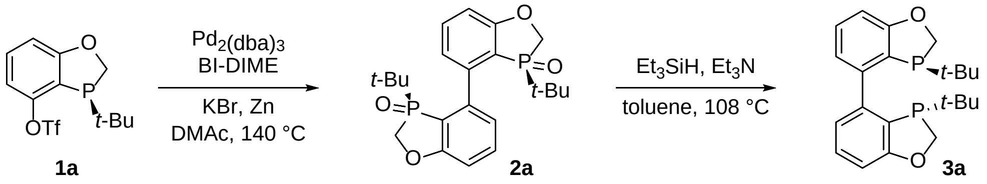

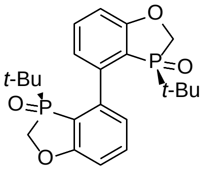

2a: A two-neck round bottom flask was charged with 2,3-dihydrobenzo[*d*][1,3]oxaphosphol-4-yl trifluoromethanesulfonate **1a** (5.37 g, 15 mmol), tetraethylammonium iodide (7.71 g, 30 mmol, 2 equiv), zinc powder (2.94 g, 45 mmol), Pd_2dba_3 (343 mg, 0.375, 2.5 mol %), and BI-DIME (248 mg, 0.75 mmol, 5 mol %). The flask was backfilled with nitrogen three times. DMAc (75 mL) was added and the mixture was stirred at 140 °C for 3h. The reaction mixture solution was cooled to room temperature and filtered through a pad of celite eluting with DCM. The filtrate was washed with 0.03% citric acid, 1M NaOH and brine. The combined organic layers were dried over $MgSO_4$, filtered and concentrated. The residue was purified by crystallization from DCE/heptane to afford the product as white solid (1.9 g, 60%). The mother liquor was concentrated and purified by column chromatography on silica gel using 0 to 5% MeOH in dichloromethane as eluent to obtain second crop of the product (0.58g, 18%). **^{1}H NMR** (500 MHz, $CDCl_3$) δ 8.14 (dd, *J* = 7.4, 3.7 Hz, 2H), 7.60 (t, *J* = 8.0, 2H), 6.98 (dd, *J* = 8.3, 2.9 Hz, 2H), 4.62

(dd, J = 13.8, 2.2 Hz, 2H), 4.45 (dd, J = 13.9, 11.2 Hz, 2H), 0.95 (d, J = 16.1 Hz, 18H); **^{13}C NMR** (125 MHz, $CDCl_3$) δ 166.7 (d, J = 19.0 Hz), 144.5 (d, J = 3.4 Hz), 135.4 (d, J = 1.6 Hz), 127.0 (d, J = 6.9 Hz), 114.5 (d, J = 5.2 Hz), 112.2 (d, J = 85.7 Hz), 65.5 (d, J = 63.3 Hz), 34.2 (d, J = 70.9 Hz), 24.4 (d, J = 1.1 Hz); **^{31}P NMR** (202 MHz, $CDCl_3$) δ 65.6; **HRMS** (ESI^+) calcd for $C_{22}H_{29}O_4P_2$ $[M+H]^+$, 419.1541; found, 419.1535.

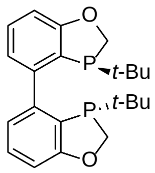

(3*S*,3'*S*)-3,3'-Di-*tert*-butyl-2,2',3,3'-tetrahydro-4,4'-bibenzo[*d*][1,3]oxa phosphole 3a: To the suspension of phosphine oxide **2a** (19.18 g, 45.84 mmol) in THF (40 mL) under argon was added 1,1,3,3-tetramethyldisiloxane (18.47, 137.52 mmol, 3.00 eq) and Ti(O*i*-Pr)$_4$ (29.97 g, 105.43 mmo, 2.30 eq). The mixture was stirred at 65 ^oC for ~2 h for complete reduction as indicated by ^{31}P NMR. Volatiles (~20 mL) were removed by distillation at normal pressure. Degassed 2-propanol (100 mL) was added and solvents (~80 mL) was removed by distillation at normal pressure. The remaining mixture was cooled to ~55 ^oC to crystallize the product. The suspension was cooled in an ice-water bath for ~1 h and filtered. The wet cake was rinsed with degassed chilled 2-propanol (80 mL) and dried to constant weight to give the desired bis-phosphine as white crystalline solid (15.43 g, 87%). **^{1}H NMR** (500 MHz, $CDCl_3$) δ 7.30 (t, J = 15.6 Hz, 2H), 6.98 (d, J = 6.7 Hz, 2H), 6.90 (dd, J = 8.2 Hz, 0.6 Hz, 2H), 4.83 (d, J = 12.8 Hz, 2H), 4.68-4.59 (m, 2H), 0.68-0.64 (m, 18H); **^{13}C NMR** (125 MHz, $CDCl_3$) δ 163.7, 145.9 (d, J = 7.7 Hz), 146.2 (d, J = 7.7 Hz), 131.0, 122.7 (d, J = 6.9 Hz), 122.6 (d, J = 7.0 Hz), 122.4 (d, J = 1.9 Hz), 122.3 (d, J = 1.9 Hz), 109.9, 70.0 (d, J = 13.8 Hz), 69.8 (d, J = 13.7 Hz), 31.35 (d, J = 7.5 Hz), 31.28 (d, J = 7.4 Hz), 26.8 (d, J = 7.4 Hz), 26.7 (d, J = 7.6 Hz); ^{31}P NMR (202 MHz, $CDCl_3$) δ −6.4; HRMS (ESI^+) calcd for $C_{22}H_{29}O_2P_2$ $[M+H]^+$: 387.1643; found: 387.1637.

Synthesis of C2,C2'-substituted ligands 3c-e:

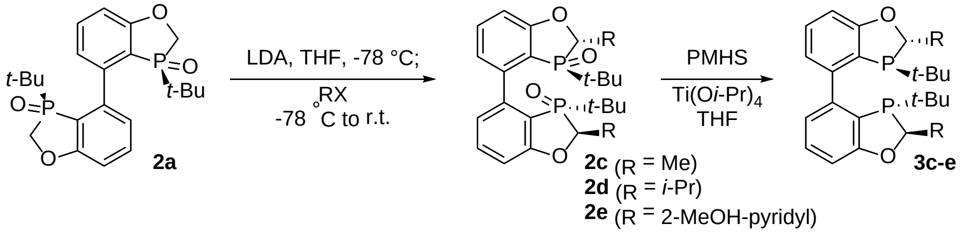

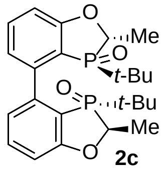

2c: To a solution of bisphosphine oxide **2a** (0.50 g, 1.19 mmol, 1 equiv) in THF (10 mL) at -78 ^oC was added LDA (1.5 mL, 2 M in THF/toluene, 2.99 mmol, 2.5 equiv). The mixture was stirred at -78 ^oC for 1 h before addition of iodomethane (0.19 mL, 2.99

mmol, 2.5 equiv). The resulting mixture was kept at -78 ^{o}C for 1.5 h before it was warmed to r.t. After stirring overnight, water and DCM were added. The organic layer was separated, dried over magnesium sulfate, concentrated, and purified by silica gel column chromatography (0-10% methanol in dichloromethane) to give the desired product (0.47 g, 1.06 mmol, 89%) as white solid. ^{1}**H NMR** (500 Mz, $CDCl_3$): δ 8.11-8.07 (m, 2H), 7.58-7.53 (m, 2H), 6.96-6.92 (m, 2H), 4.68-4.61 (m, 2H), 1.66-1.60 (m, 6H), 0.94 (s, 9H), 0.91 (s, 9H); ^{13}**C NMR** (125 MHz, $CDCl_3$): δ 165.2, 165.0, 144.6 (d, *J* = 1.6 Hz), 144.5 (d, *J* = 1.6 Hz), 135.11, 135.10, 127.2, 127.1, 114.4, 114.3, 112.0, 111.4, 69.9 (t, *J* = 64.9 Hz), 33.8 (d, *J* = 70.7 Hz), 31.6 (d, *J* = 7.5 Hz), 24.5, 15.8 (d, *J* = 1.4 Hz); ^{31}**P NMR** (202 MHz, $CDCl_3$): δ 63.4.

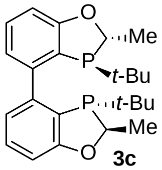

3c

(2*S*,2'*S*,3*S*,3'*S*)-3,3'-di-*tert*-butyl-2,2'-dimethyl-2,2',3,3'-tetrahydro-4,4'-bibenzo[*d*][1,3]oxaphosphole 3c:To a solution of bisphosphine oxide **2c** (400 mg, 0.90 mmol) in THF (4 mL) at r.t. was added PMHS (0.6 g) and Ti(O*i*-Pr)$_4$ (0.6 mL, 2.06 mmol, 2.3 equiv). The mixture was stirred at 60 °C for 20 h, and then concentrated under vacuum to remove most THF. 30% aqueous NaOH solution (4 mL) was carefully added to the residue. Gas was generated during the addition. The resulting mixture was further stirred at 65 ^{o}C for 0.5 h. To the mixture at r.t. was added MTBE (3x4 mL). The MTBE solution was dried, concentrated, and purified by passing through a neutral alumina plug affording the desired product as white solid (334 mg, 0.81 mmol, 90%). ^{1}**H NMR** (500, Mz $CDCl_3$): δ 7.29 (t, *J* = 7.8 Hz, 2H), 6.96 (bs, 2H), 6.87 (d, *J* = 8.1 Hz, 2H), 5.00 (q, *J* = 7.1 Hz, 2H), 1.57-1.49 (m, 6H), 0.67-063 (m, 18H); ^{13}**C NMR** (125 MHz, $CDCl_3$): δ 163.2, 146.7 (t, *J* = 7.4 Hz), 131.0, 122.3 (t, *J* = 1.9 Hz), 110.3, 79.4 (t, *J* = 13.0 Hz), 31.7 (d, *J* = 7.5 Hz), 31.6 (d, *J* = 7.5 Hz), 29.7, 27.1 (t, *J* = 7.5 Hz), 21.3 (t, *J* = 15.6 Hz); ^{31}**P NMR** (202 MHz, $CDCl_3$): δ 9.4; **HRMS** (ESI^+) calcd for $C_{24}H_{33}O_2P_2$ $[M+H]^+$: 415.1956; found: 415.1950.

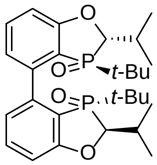

2d: To a solution of bisphosphine oxide **2a** (400 mg, 0.96 mmol, 1 equiv) in THF (4 mL) at -78 ^{o}C was added LDA (1.2 mL, 2 M in THF/toluene, 2.39 mmol, 2.5 equiv). The mixture was stirred at -78 ^{o}C for 1 h before addition of *i*-PrI (0.24mL, 2.39 mmol, 2.5 equiv). The resulting mixture was kept at -78 ^{o}C for 1.5 h before it was warmed to r.t. After stirring overnight, water and DCM were added. The organic layer was separated, dried over magnesium sulfate, concentrated, and purified by silica gel column chromatography (0-10% methanol in EtOAc) to give the desired product (341 mg, 0.68 mmol, 71%) as white solid. ^{1}**H NMR** (500 MHz $CDCl_3$): δ 8.16-8.11 (m, 2H), 7.58-7.52 (m, 2H), 7.96-7.92 (m, 2H), 4.18-4.14 (m, 2H), 2.46-2.36 (m,

2H), 1.20 (d, J = 6.7 Hz, 12H), 1.16 (d, J = 6.7 Hz, 12H), 0.93 (s, 9H), 0.90 (s, 9H); **^{13}C NMR** (125 MHz, $CDCl_3$): δ 165.4, 165.2, 144.5 (d, J = 1.4 Hz), 144.6 (d, J = 1.4 Hz), 134.93, 134.92, 127.31, 127.25, 113.86, 113.82, 112.1, 111.5, 77.9 (d, J = 63.3 Hz), 34.0 (d, J = 70.2 Hz), 30.1, 24.2, 19.7 (d, J = 4.5 Hz), 18.4 (d, J = 5.4 Hz); **^{31}P NMR** (202 MHz, $CDCl_3$): δ 63.9. HRMS (ESI^+) calcd for $C_{28}H_{41}O_4P_2$ $[M+H]^+$: 503.2480; found: 503.2476.

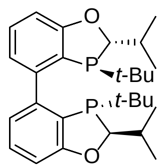

(2*S*,2'*S*,3*S*,3'*S*)-3,3'-di-*tert*-butyl-2,2'-diisopropyl-2,2',3,3'-tetrahydro-4,4'-bibenzo[*d*][1,3]oxaphosphole 3d: To a solution of bisphosphine oxide **2d** (300 mg, 0.60 mmol) in THF (3 mL) at r.t. was added PMHS (0.5 g) and Ti(O*i*-Pr)$_4$ (0.44 mL, 1.49 mmol, 2.5 equiv). The mixture was stirred at 60 °C for 20 h, and then concentrated under vacuum to remove most THF. 30% aqueous NaOH solution (4 mL) was carefully added to the residue. Gas was generated during addition. The resulting mixture was further stirred at 65 ^{o}C for 0.5 h. To the mixture was added MTBE (3x4 mL) at r.t. The MTBE solution was dried, concentrated, and purified by passing through a neutral alumina plug affording the desired product as white solid (253 mg, 0.54 mmol, 90%). **^{1}H NMR** (500 MHz $CDCl_3$): δ 7.26 (t, J = 7.8 Hz, 2H), 6.90 (bs, 2H), 6.86 (d, J = 7.8 Hz, 2H), 4.58 (d, J = 7.1 Hz, 2H), 2.20-2.08 (m, 2H), 1.10-1.04 (m, 12H), 0.66-0.60 (m, 18H); **^{13}C NMR** (125 MHz, $CDCl_3$): δ 163.4, 145.3 (t, J = 4.2 Hz), 129.7, 121.6 (t, J = 2.1 Hz), 121.2 (t, J = 9.4 Hz), 108.5, 98.0 (t, J = 14.4 Hz), 31.8 (t, J = 11.0 Hz), 30.4 (d, J = 7.8 Hz), 30.3 (d, J = 7.8 Hz), 26.0 (t, J = 7.6 Hz), 18.1 (m), 18.0 (m); **^{31}P NMR** (202 MHz, $CDCl_3$): δ -3.0. **HRMS** (ESI^+) calcd for $C_{28}H_{41}O_2P_2$ $[M+H]^+$: 471.2582; found: 471.2576.

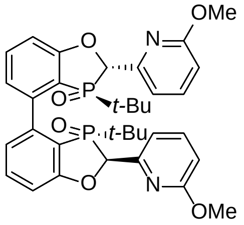

2e: To a solution of bisphosphine oxide **2a** (1.4g, 2.72 mmol, 1 equiv) and 2-methoxy-6-(phenylsulfonyl)pyridine (1.39g, 5.59 mmol, 2.05 equiv) in THF (20 mL) at -20 ^{o}C was added LDA (8.2 mL, 2 M in THF/toluene, 16.35 mmol, 6.0 equiv). The mixture was stirred at -20 ^{o}C for 0.5 h. Upon completion MeOH (10 ml) was added. After stirring overnight, additional amount of MeOH was added resultin in formation of a slurry. The solids were separated by filtration, rinsed with THF-water, water, and MTBE to give the desired product (1.52 g, 2.40 mmol, 88%) as white solid. **^{1}H NMR** (400 MHz, $CDCl_3$) δ 7.95 (m, 2H), 7.55 (m, 4H), 7.10 (dd, J = 8.4 Hz, J = 3.2 Hz, 2H), 6.93 (d, J = 7.6 Hz, 2H), 6.63 (d, J = 8.0 Hz, 2H), 5.62 (s, 2H), 3.85 (s, 6H), 1.05 (d, J = 16.4 Hz,18H); **^{13}C NMR** (100 MHz, $CDCl_3$) δ 165.5 (d, J = 18.2 Hz), 163.5 (d, J = 1.7 Hz) , 151.7 (d, J = 4.6 Hz), 144.4 (d, J = 4.1 Hz), 139.3 (d, J = 2.0 Hz), 135.5 (d, J = 1.2 Hz), 127.7 (d, J = 7.2 Hz), 114.3 (d, J = 5.2 Hz), 113.2 (d, J = 3.2

Hz), 111.2 (d, J = 83.6 Hz), 110.1 (d, J = 2.1 Hz), 76.6 (d, J = 34.0 Hz), 53.4, 34.5 (d, J = 70.3 Hz), 24.6; ^{31}**P NMR** (162 MHz, $CDCl_3$) δ 65.4.

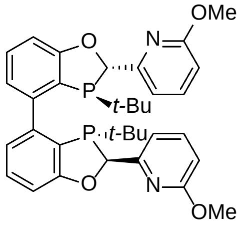

6,6'-((2*S*,2'*S*,3*S*,3'*S*)-3,3'-di-*tert*-butyl-2,2',3,3'-tetrahydro-[4,4'-bibenzo[*d*][1,3] oxaphosphole]-2,2'-diyl)bis(2-methoxypyridine) 3e: To a solution of bismethylated bisphosphine oxide **2e** (1.45 g, 2.29 mmol) in THF (6 mL) at r.t. was added PMHS (3.7 g) and Ti(O*i*-Pr)$_4$ (1.63 mL, 5.73 mmol, 2.5 equiv). The mixture was stirred at 80 °C for 18 h, and then concentrated under vacuum to remove most THF. 30% aqueous NaOH solution (4 mL) was carefully added to the residue. Gas was generated during addition. The resulting mixture was further stirred at 65 ^{o}C for 0.5 h. To the mixture was added MTBE (3x4 mL) at r.t. The MTBE solution was dried, concentrated, and purified by passing through a neutral alumina plug affording the desired product as white solid (1.17 mg, 1.95 mmol, 8%). ^{1}**H NMR** (400 MHz, CDCl3) δ 7.39-7.32 (m, 4H), 7.20-6.95 (m, 4H), 6.66 (d, J = 7.2 Hz, 2H), 6.49 (d, J = 8.4 Hz, 2H), 5.71 (s, 2H), 3.76 (s, 6H), 0.69 (br s, 18H); ^{13}**C NMR** (100 MHz, CDCl3) δ 164.8, 163.2, 158.2, 146.4, 138.2, 131.2, 122.9, 121.8, 111.8, 109.9, 109.4, 85.7, 53.0, 32.2 (t, J = 27.9 Hz), 26.9 (t, J = 7.7 Hz); ^{31}**P NMR** (162 MHz, CDCl3) δ 17.2. **HRMS** (ESI^{+}) calcd for $C_{34}H_{39}O_4N_2P_2$ $[M+H]^{+}$: 601.2385; found: 601.2379.

Synthesis of 1b:

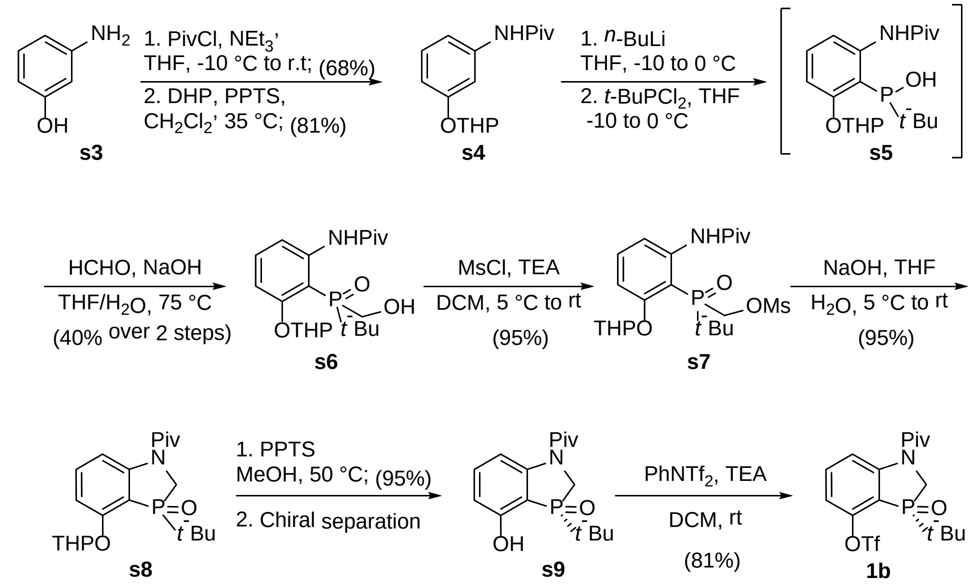

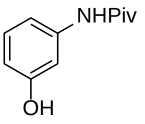

***N*-(3-Hydroxyphenyl)pivalamide**: A solution of 3-aminophenol (300 g, 2.75 mol) in THF (2.4 L) was cooled to −10 °C. Pivaloyl chloride (339 mL, 2.75 mol) was added below −5 °C. After complete addition, the mixture was kept for 2 h. Triethylamine (113 mL, 0.825 mol) was added slowly below −5 °C. After 2 h, additional triethylamine (0.1 eq) was added to achieve complete conversion. The reaction mixture was warmed to 23 °C and acidified to pH = 1 with concentrated HCl. THF was removed by distillation under vacuum. The solid was collected by filtration and then washed with H_2O to give the product as a white solid (360 g, 68% yield). **^{1}H NMR** (400 MHz, $CDCl_3$) δ 8.19 (s, 1H), 7.96 (t, *J* = 2.2, 1H), 7.41 (s, 1H), 7.15, (t, *J* = 2.2, 1H), 6.66 (ddd, *J* = 8.2, 2.4, 0.8 Hz, 1H), 6.52 (ddd, *J* = 8.0, 2.0, 0.8 Hz, 1H), 1.34 (s, 9H); **^{13}C NMR** (100 MHz, $CDCl_3$) δ 117.8, 157.8, 138.5, 129.7, 112.0, 110.5, 107.5, 39.8, 27.6.

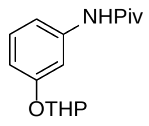

s4: To a solution of *N*-(3-hydroxyphenyl)pivalamide (360 g, 1.87 mol) and pyridinium *para*-toluenesulfonate (PPTS) (46.9 g, 0.187 mol) in CH_2Cl_2 (2 L) was added DHP (485 mL, 5.60 mol). The reaction mixture was then heated at 35 °C overnight. Additional DHP (1 eq) was added to push the reaction to completion. The reaction was quenched with saturated aqueous $NaHCO_3$ (400 mL) and then washed with H_2O (2 × 400 mL). The organic layer was dried ($MgSO_4$) and concentrated. The crude product was recrystallized from EtOAc/hexanes to produce the product as a white solid (420 g, 81% yield). **^{1}H NMR** (400 MHz, $CDCl_3$) δ 7.35 (t, *J* = 2.2 Hz, 1H), 7.29 (bs, 1H), 7.21 (t, *J* = 8.1 Hz, 1H), 7.12 (qd, *J* = 8.0, 0.9 Hz, 1H), 6.80 (ddd, *J* = 8.2, 2.5, 0.9 Hz, 1H), 5.44 (t, *J* =

3.2 Hz, 1H) 3.90 (m, 1H), 3.61 (dtd, J = 11.3, 4.1, 1.2 Hz, 1H), 1.99 (m, 1H), 1.84 (m, 2H), 1.55−1.75 (m, 3H), 1.31 (s, 9H); ^{13}**C NMR** (100 MHz, $CDCl_3$) δ 176.5, 157.6, 139.1, 129.6, 113.1, 112.4, 108.3, 96.3, 61.9, 39.6, 30.3, 27.6, 25.2, 18.6.

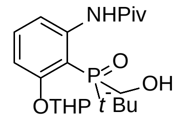

s6: A mixture of **s4** (50.00 g, 180.3 mmol) and THF (500 mL) was cooled to −10 °C under argon. A solution of *n*-BuLi (2.5 M in hexanes, 181 mL, 453 mmol) was added dropwise below 0 °C. The mixture was then kept at 2 to 5 °C for 2 h. A solution of *t*-BuPCl$_2$ (72.5% w/w in THF, 51.4 g solution, 235 mmol) was added dropwise below 0 °C. After complete addition, the reaction mixture was warmed to 23 °C and left overnight at rt. H_2O (50 mL) was added, followed by the addition of aqueous NaOH (6 M, 60 mL, 360 mmol) and a solution of formaldehyde (37 w/v% in H_2O, 30 mL, 360 mmol). The mixture was heated to remove the organic solvent to reach an internal temperature 70−75 ^{o}C. Another portion of formaldehyde (37 w/v% in H_2O, 44 mL, 540 mmol) was added. The mixture was kept at 70−75 °C until the reaction was complete (4 h). The mixture was cooled to 40−45 °C over at least 0.5 h. MTBE (150 mL) was added. The slurry was further cooled to 20−25 °C over at least 0.5 h. After being stirred at 20−25 °C for at least 0.5 h, the solid was collected by filtration and washed with MTBE (100 ml). After drying under vacuum, the product was isolated as a white solid (40% yield). ^{1}**H** NMR (400 MHz, $CDCl_3$) δ 11.75 (d, J = 33.2 Hz, 1H), 8.32 (dd, J = 8.4, 3.2 Hz, 1H), 7.42 (t, J = 8.4 Hz, 1H), 6.91 (dd, J = 8.4, 4.4 Hz, 0.4H, diastereomer 1), 6.86 (dd, J = 8.4, 4.4 Hz, 0.6H, diastereomer 2), 5.40−5.36 (m, 0.6H, diastereomer 2), 5.31−5.28 (m, 0.4H, diastereomer 1), 4.47−4.35, (m, 1.6H), 4.14 (ddd, J = 13.8, 3.4, 1.7 Hz, 0.4H, diastereomer 1), 3.90−3.81 (m, 1H), 3.71−3.60 (m, 1H), 3.33 (ddd, J = 8.0, 7.0, 4.2 Hz, 0.6H, diastereomer 2), 3.19 (dt, J = 10.0, 1.8 Hz, 0.4H, diastereomer 1), 2.03−1.62 (m, 6H), 1.32 (s, 9H), 1.21 (m, 9H); ^{13}**C NMR** (100 MHz, $CDCl_3$) δ 177.9, 147.1, 146.7, 134.6, 134.4, 115.8, 115.7, 115.6, 115.5, 108.5, 108.4, 108.1, 108.1, 98.9, 97.5, 64.0, 63.5, 59.8, 59.1, 59.1, 58.4, 40.2, 35.9, 35.4, 30.2, 30.1, 27.5, 24.7, 24.6, 24.4, 24.4, 20.2, 20.0; ^{31}**P** NMR (162 MHz, $CDCl_3$) δ 59.11, 58.42. **HRMS** (ESI$^+$) calcd for $C_{21}H_{34}NNaO_5P$ $[M+Na]^+$: 434.2072; found: 434.2075.

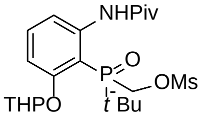

s7: To a solution of **s6** (16.39 g, 39.83 mmol) in CH_2Cl_2 (160 mL) was added triethylamine (28 mL, 200 mmol). After the solution was cooled to −5 °C, MsCl (4.7 mL, 60 mmol) was added dropwise below 5 °C. After complete addition, the reaction mixture was stirred at rt for 1.5 h. The reaction was quenched with H_2O (160 mL) and extracted with CH_2Cl_2 (3 × 32 mL). The organic layers were combined, dried over anhydrous $MgSO_4$ and concentrated to remove most of the CH_2Cl_2. Hexane was added, and the remaining CH_2Cl_2 was removed. The solid

was collected by filtration to give the product as a white solid (18.4 g, 95% yield). **^{1}H** NMR (500 MHz, $CDCl_3$) δ 11.72−11.62 (m, 1H), 8.34 (dt, *J* = 8.3, 3.4 Hz, 1H), 7.44 (t, *J* = 8.4 Hz, 1H), 6.96−6.88 (m, 1H), 5.42−5.28 (m, 1H), 5.13−4.85 (m, 2H), 3.94−3.80 (m, 1H), 3.74−3.60 (m, 1H), 3.22−3.18 (m, 2H), 2.05−1.62 (m, 6H), 1.31 (s, 9H), 1.24 (m, 9H); **^{13}C NMR** (125 MHz, $CDCl_3$) δ 177.9, 158.2, 158.2, 147.3, 135.0, 134.9, 115.9, 115.9, 115.8, 108.6, 108.5, 108.5, 108.4, 100.5, 99.2, 97.7, 64.7, 64.1, 64.1, 63.8, 63.6, 40.3, 38.3, 38.3, 36.5, 36.5, 35.9, 30.1, 27.6, 27.5, 24.7, 24.5, 24.5, 20.2, 19.9, 18.6; **^{31}P NMR** (202 MHz, $CDCl_3$) δ 55.25. **HRMS** (ESI^+) calcd for $C_{22}H_{36}NNaO_7PS$ $[M+Na]^+$: 512.1848; found: 512.1859.

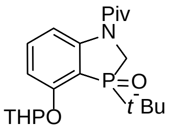

s8: A solution of **s7** (61.5 g, 126 mmol) in THF (600 mL) was cooled to 0 °C. Aqueous NaOH (1 M, 132 mL, 132 mmol) was added slowly while keeping the temperature below 5 °C. After addition, the reaction mixture was stirred at rt for 17 h. The reaction mixture was diluted with H_2O (300 mL). THF was removed under vacuum. The white solid was collected to give the product (47.0 g, 95% yield). **^{1}H NMR** (400 MHz, $CDCl_3$) δ 7.91−7.82 (m, 1H), 7.42 (t, *J* = 8.4 Hz, 1H), 6.98 (dd, *J* = 8.2, 4.4 Hz, 0.3H, diastereomer 1), 6.86 (dd, *J* = 8.3, 4.4 Hz, 0.7H, diastereomer 2), 5.73, (bs, 0.7H), 5.37 (bs, 0.3H), 4.35 (dd, *J* = 13.7, 6.8 Hz, 1H), 4.03 (t, *J* = 14.4 Hz, 1H), 3.95 (dq, *J* = 10.3, 2.5 Hz, 1H), 3.70 (td, *J* = 11.6, 3.4 Hz, 0.3H, diastereomer 1), 3.55 (m, 0.7H, diastereomer 2), 2.27−1.83 (m, 3H), 1.74−1.55 (m, 3H), 1.42 (s, 9H), 1.31−1.21 (m, 9h); **^{13}C NMR** (125 MHz, $CDCl_3$) δ 177.9, 135.0, 134.9, 115.8, 115.8, 108.6, 108.5, 99.2, 97.6, 64.8, 64.1, 63.8, 63.6, 40.3, 38.4, 38.3, 36.5, 30.1, 27.6, 27.5, 24.7, 24.5, 24.5, 20.2, 19.9; **^{31}P NMR** (162 MHz, $CDCl_3$) δ 58.70. **HRMS** (ESI^+) calcd for C21H32NNaO_4P $[M+Na]^+$: 416.1967; found: 416.1981.

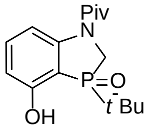

s9: To a solution of **s8** (33.00 g, 83.87 mmol) in MeOH (165 ml) was added PPTS (4.22 g, 16.8 mmol). The mixture was heated at 50 °C for 2 h. After most of the MeOH was removed by distillation, water was added. The product was isolated as a white solid (89% yield). Enantiomers were separated by preparative SFC (2.1 x 25.0 cm Chiralcel OX-H from Chiral Technologies (West Chester, PA); co-solvent: methanol with 0.25% isopropylamine, 35% Co-solvent at 80 g/min, isocratic, 110 bar, 25 °C) providing **(*S*)-s9** in 99.9 %ee and **(*R*)-s9** in 99.6 %ee. **^{1}H NMR** (400 MHz, $CDCl_3$) δ 9.98 (bs, 1H), 7.73 (dd, *J* = 8.5, 3.1 Hz, 1H), 7.29 (t, *J* = 8.3 Hz, 1H), 6.68 (dd, *J* = 8.1, 4.5 Hz, 1H), 4.29 (dd, *J* = 13.9, 5.7 Hz, 1H), 4.06 (t, *J* = 14.1 Hz, 1H), 1.14 (s, 9H), 1.24 (m, 9H); **^{13}C NMR** (100 MHz, $CDCl_3$) δ 177.1, 177.1, 159.8, 150.5, 150.4, 135.9, 112.5, 112.5, 111.9, 111.8, 46.6, 46.0, 41.0, 34.2, 33.5, 28.3, 24.4; **^{31}P NMR** (162 MHz, $CDCl_3$) δ 62.74. **HRMS** (ESI^+) calcd for $C_{16}H_{25}NO_3P$ $[M+H]^+$: 310.1572; found: 310.1578.

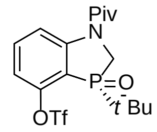

1b: A solution of 1-(3-(*tert*-butyl)-4-hydroxy-3-oxido-2-hydrobenzo[*d*][1,3]azaphosphol-1-yl)-2,2-dimethylpropan-1-one (20.3 g, 65.5 mmol) in CH_2Cl_2 (30 mL) was charged with triethylamine (18 mL, 131 mmol) and phenyl triflimide (25.8 g, 72.1 mmol). The solution was stirred at rt for 1 h. Upon completion of the reaction, the mixture was cooled to 0 °C and washed successively with cold 5% aqueous NaOH (2 × 100 mL), cold aqueous HCl (1M, 100 mL), and cold H_2O (100 mL). The organic layer was dried ($MgSO_4$) and concentrated. The residue was triturated with hexanes. The solid was collected by filtration and washed with hexanes to give the product (23.3 g, 81%). **^{1}H NMR** (400 MHz, $CDCl_3$) δ 8.27 (dd, *J* = 8.6, 2.2 Hz, 1H), 7.58 (t, *J* = 8.4 Hz, 1H), 7.17 (dd, *J* = 8.2, 3.7 Hz, 1H), 4.40 (dd, *J* = 13.9, 7.0 Hz, 1H), 4.17 (dd, *J* = 15.3, 14.0 Hz, 1H), 1.43 (s, 9H), 1.24 (d, *J* = 16.5 Hz, 9H); **^{13}C NMR** (125 MHz, $CDCl_3$) δ 177.5, 177.4, 151.3, 151.2, 150.0, 135.7, 135.7, 132.1, 131.0, 130.0, 122.3, 120.6, 120.6, 119.8, 117.2, 115.8, 115.8, 115.7, 115.7, 114.7, 112.2, 111.5, 46.4, 46.0, 45.8, 41.2, 34.9, 34.3, 28.1, 23.9, 23.9, 8.6; **^{31}P NMR** (162 MHz, $CDCl_3$) δ 57.91. **HRMS** (ESI$^+$) calcd for $C_{17}H_{24}F_3NO_5PS$ [M+H]$^+$: 442.1065; found: 442.1095. Optical Rotation: $[\alpha]_D^{22}$ +78.89 (c = 0.0188, $CHCl_3$)

Table S4. Screening of ligands for Pd-catalyzed reductive homocoupling of benzoazaphosphol triflate **1b**:

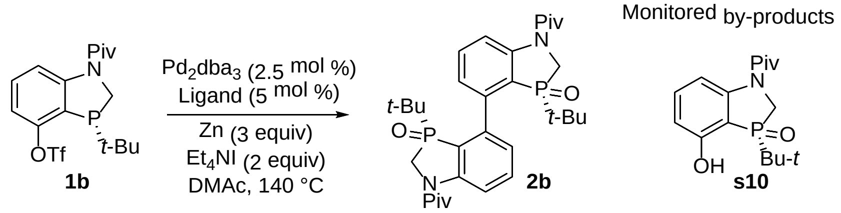

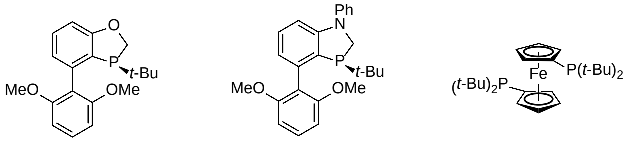

BI-DIME **NPh-BI-DIME** **DTBPF**

Entry	Ligand	% **1b**^{a}	% **2b**^{a}	% **s10**^{a}	**% s11**^{a}
1	BI-DIME	33	50	9	8
2	NPh-BI-DIME	3	83	3	11
3	DTBPF	0	92	1	7

^{a}Relative HPLC integration.

Synthesis of 3b:

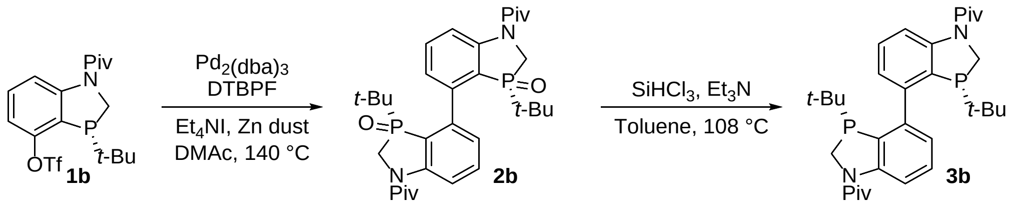

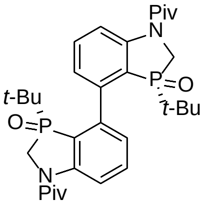

2b: To a mixture of *S*-3-(*tert*-butyl)-1-pivaloyl-2,3-dihydro-1*H*-benzo[*d*][1,3]azaphosphol-4-yl trifluoromethanesulfonate **1b** (3.34 g, 7.56 mmol), tetraethylammonium iodide (3.89 g, 15.12 mmol), zinc dust (1.48 g, 22.68 mmol), Pd_2dba_3 (173.1 mg, 0.19 mmol), and 1,1'-bis(di-*tert*-butylphosphino)ferrocene (DTBPF) (86.7 mg, 0.19 mmol) was added DMAc (33 mL) under nitrogen. The reaction mixture was stirred at 140 °C for 1h under nitrogen. Upon completion, the reaction mixture was cooled to room temperature and filtered through a pad of celite eluting with EtOAc. Organic layer was separated, washed with 0.1 M aqueous citric acid and brine (3 times), dried over Na_2SO_4, filtered and concentrated. The residue was purified by column chromatography on silica gel using 0 to 10% MeOH in EtOAc as eluent followed by recrystallization from MTBE(5V)/Heptane(30V) to afford the desired product as pale brown solid (1.71 g, 78%). The product is a mixture of atropisomers in 1.25:1 ratio: **^{1}H NMR** (500 MHz, $CDCl_3$) δ 8.34 (dd, *J* = 8.4, 2.5 Hz, 0.8H), 8.24 (dd, *J* = 7.5, 4.0 Hz, 1H), 8.12 (dd, *J* = 8.4, 2.0 Hz, 1H), 7.62 (t, *J* = 8.1 Hz, 1H), 7.50 (t, *J* = 7.9 Hz, 0.8H), 7.17 (dd, *J* = 7.1, 2.7 Hz, 0.8H), 4.43 (dd, *J* = 13.6, 6.5 Hz, 1H), 4.36-4.20 (m, 1.6H), 4.08 (t, *J* = 14.3 Hz, 1H), 1.44 (s, 9H), 1.42 (s, 7.2H), 0.92 (d, *J* = 16.0 Hz, 9H), 0.83 (d, *J* = 15.0 Hz, 7.2H); **^{13}C NMR** (125 MHz, $CDCl_3$) δ 177.2 (d, *J* = 3.3 Hz), 177.1 (d, *J* = 3.6 Hz), 151.1 (d, *J* = 14.9 Hz), 147.4 (d, *J* = 15.9 Hz), 144.1 (m), 140.7 (m), 133.4, 132.1, 129.9 (d, *J* = 7.9 Hz), 125.5 (d, *J* = 8.2 Hz), 121.7 (d, *J* = 8.1 Hz), 120.5 (d, *J* = 8.6 Hz), 118.2, 117.5, 46.4 (d, *J* = 65.6 Hz), 44.9 (d, *J* = 64.6 Hz), 41.1, 34.33 (d, *J* = 70.5 Hz), 34.25 (d, *J* = 69.5 Hz), 28.3, 24.2, 24.1; **^{31}P NMR** (202 MHz, $CDCl_3$) δ 61.8 (2P), 55.6 (1.6P). **HRMS** (ESI^+) calcd for $C_{32}H_{47}O_4N_2P_2$ $[M+H]^+$ $[M+H]^+$, 585.3011; found, 585.3007. Optical Rotation: $[\alpha]_D^{22}$ +210.95 (c = 0.0092, $CHCl_3$)

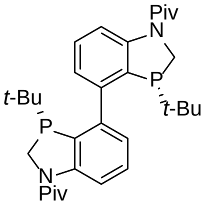

1,1'-((3*R*,3'*R*)-3,3'-di-*tert*-butyl-2,2',3,3'-tetrahydro-1*H*,1'*H*-[4,4'-bibenzo[*d*][1,3] azaphosphole]-1,1'-diyl)bis(2,2-dimethylpropan-1-one) (3b): To a solution of **2b** (585.0 mg, 1.0 mmol) in toluene were added trimethylamine (0.81 g, 8.0 mmol) and trichlorosilane (1.08 g, 8.0 mmol) under argon. The reaction mixture was stirred at

108 °C for 4h. Upon completion, the reaction mixture was cooled to room temperature and slowly quenched with argon-purged 30% aqueous NaOH and stirred at room temperature for 30 min. To this solution, EtOAc/DCM (1:1, argon-pured) mixture was added and organic layer was separated and passed through the plug of neutral alumina under argon. The solution was concentrated to afford the product as pale yellow solid (330 mg, 60%). **^{1}H NMR** (500 MHz, $CDCl_3$) δ 8.23 (d, *J* = 8.4 Hz, 2H), 7.39 (t, *J* = 7.8 Hz, 1H), 7.38 (t, *J* = 7.3 Hz, 1H), 7.09 (d, *J* = 7.3 Hz, 2H), 4.38 (d, *J* = 13.2 Hz, 2H), 4.10-4.02 (m, 2H), 1.46 (s, 18H), 0.67-0.61 (m, 18H). **^{13}C NMR** (125 MHz, $CDCl_3$) δ 177.0 (t, *J* = 1.2 Hz), 149.4, 145.3 (t, *J* = 9.1 Hz), 130.3, 127.2 (t, *J* = 4.8 Hz), 125.8 (t, *J* = 1.9 Hz), 119.2, 46.3 (t, *J* = 12.5 Hz), 41.0, 31.92 (d, *J* = 7.7 Hz), 31.86 (d, *J* = 7.6 Hz), 28.4, 27.2 (*t*, *J* = 7.6 Hz). **^{31}P NMR** (202 MHz, $CDCl_3$) δ -10.8. **HRMS** (ESI^+) calcd for $C_{32}H_{47}O_2N_2P_2$ $[M+H]^+$: 553.3113; found: 553.3108. Optical Rotation: $[\alpha]_D^{22}$ +374.72 (c = 0.0096, $CHCl_3$)

3 Dynamic kinetic resolution of α-substituted tetralones

3.1 Synthesis of α-aryl tetralone derivatives

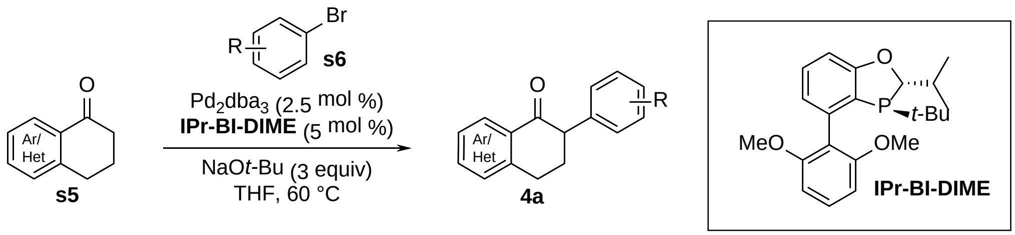

General procedure for the synthesis of α-aryl tetralone derivatives a-m. A mixture of Pd_2dba_3 (2.5 mol %), IPr-BI-DIME (5 mol %), and NaO*t*-Bu (3 equiv) was suspended in THF. Ketone **s5** (1 equiv) and corresponding aryl halide **s6** (1.2 equiv) were added subsequently. The reaction mixture was stirred at 60 °C for 2 h under Ar atmosphere. Upon completion, the reaction mixture was filtered through pad of celite, diluted with EtOAc, washed with saturated NH_4Cl solution, dried over anhydrous Na_2SO_4, filtered, and concentrated. The residue was purified by silica gel column chromatography or recrystallization to afford corresponding α-aryl tetralone derivative **4**. Compounds **4a**,[7] **b**,[8], **c**, **d**, **e**, **f**, **q**,[9] are known and their analytical data matched the reported data.

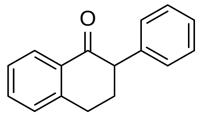

4a:[7] white solid (60%). **^{1}H NMR** (500 MHz, $CDCl_3$) δ 8.09 (dd, *J* = 7.8, 0.8 Hz, 1H), 7.50 (dt, *J* = 7.5, 1.3 Hz, 1H), 7.36-7.31 (m, 3H), 7.29-7.34 (m, 2H), 7.21-7.17 (m, 2H), 3.84-3.76 (m, 1H), 3.15-3.00 (m, 2H), 2.48-2.40 (m, 2H); **^{13}C NMR** (125 MHz, $CDCl_3$) δ 198.2, 144.1, 139.8, 133.4, 132.9, 128.8, 128.5, 128.4, 127.8, 126.9, 126.8, 54.4, 31.2, 28.8.

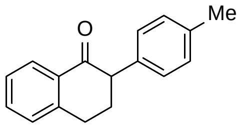

4b:[8]white solid (57%). **^{1}H NMR** (400 MHz, $CDCl_3$) δ 8.13 (dd, *J* = 7.9, 0.9 Hz, 1H), 7.52 (dt, *J* = 7.5, 1.3 Hz, 1H), 7.36 (t, *J* = 7.8 Hz, 1H), 7.31 (d, *J* = 7.8 Hz, 1H), 7.10-7.19 (m, 4H), 3.78-3.82 (m, 1H), 3.04-3.18 (m, 2H), 2.42-2.48 (2H, m), 2.37 (s, 3H); **^{13}C NMR** (100 MHz, $CDCl_3$) δ 198.4, 144.1, 136.6, 136.5, 133.3, 132.9, 129.2, 128.7, 128.3, 127.8, 126.7, 54.0, 31.2, 28.8, 21.1.

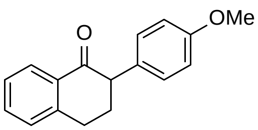

4c:[9]pale yellow solid (64%). **^{1}H NMR** (500 MHz, $CDCl_3$) δ 8.0.9 (dd, *J* = 8.0, 0.7 Hz, 1H), 7.49 (dt, *J* = 7.5, 1.4 Hz, 1H), 7.33 (t, *J* = 7.5 Hz, 1H), 7.27 (d, *J* = 7.3 Hz, 1H), 7.13-7.09 (m, 2H), 6.90-6.86 (m, 2H), 3.79 (s, 3H), 3.75 (t, *J* = 8.0 Hz,

[7] Marion, N.; Ecarnot, E. C.; Navarro, O.; Amoroso, D.; Bell, A.; Nolan, S. P. *J. Org. Chem.* **2006**, *71*, 3816-3821.
[8] Crawford, S. M.; Alsabeh, P. G.; Stradiotto, M. *Eur. J. Org. Chem.* **2012**, 6042-6050.
[9] Yin, H.-Y.; Lin, X.-L.; Li, S.-W.; Shao, L.-X. *Org. Biomol. Chem.* **2015**, *13*, 9012-9021

1H), 3.15-3.00 (m, 2H), 2.43-2.37 (m, 2H) ppm. **^{13}C NMR** (125 MHz, $CDCl_3$) δ 198.5, 158.5, 144.1, 133.4, 132.9, 131.8, 129.4, 128.8, 127.8, 126.7, 114.0, 55.3, 53.6, 31.2, 28.8.

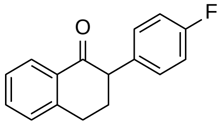

4d:[9] pale yellow solid (63%). 500 MHz, $CDCl_3$): δ 8.09 (d, *J* = 7.9 Hz, 1H), 7.51 (t, *J* = 3.7 Hz, 1H), 7.34 (t, *J* = 7.6 Hz, 1H), 7.29 (d, *J* = 7.1 Hz, 1H), 7.16 (d, *J* = 7.1 Hz, 2H), 7.03 (d, *J* = 8.5 Hz, 2H), 3.18-3.11 (m, 1H), 3.05 (dt, *J* = 16.6, 4.2 Hz, 1H), 2.43-2.40 (m, 2H); **^{13}C NMR** (125 MHz, $CDCl_3$): δ 197.9, 161.8 (d, *J* = 243.5 Hz), 160.8, 143.9, 135.4 (d, *J* = 3.3 Hz), 133.5, 132.7, 129.9 (d, *J* = 7.9 Hz), 128.8, 127.8, 126.8, 115.3 (J = 21.3 Hz), 53.7, 31.2, 28.9.

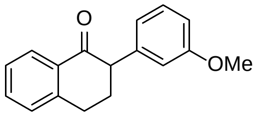

4e:[9] white solid (54%). **^{1}H NMR** (400 MHz, $CDCl_3$) δ 8.1 (d, *J* = 7.9 Hz, 1H), 7.51 (t, *J* = 7.6 Hz, 1H), 7.33 (t, *J* = 7.6 Hz, 1H), 7.23-7.28 (m, 2H), 6.74-6.83 (m, 3H), 3.78 (s, 3H), 3.75-3.79 (m ,1H), 3.02-3.11 (m, 2H), 2.40-2.45 (m, 2H); **^{13}C NMR** (100 MHz, $CDCl_3$) δ 198.0, 159.7, 144.1, 141.3, 133.4, 132.8, 129.5, 128.8, 127.8, 126.8, 120.8, 114.5, 112.2, 55.2, 54.4, 31.1, 28.7.

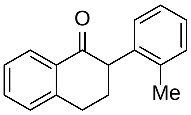

4f:[9] pale yellow oil (72%). **^{1}H NMR** (500 MHz, $CDCl_3$) δ 8.10 (d, *J* = 7.8 Hz, 1H), 4.50 (dt, *J* = 1.1 Hz, 7.5 Hz, 1H), 7.34 (t, *J* = 7.5 Hz, 1H), 7.29 (d, *J* = 7.7 Hz, 1H), 7.22-7.13 (m, 3H), 7.08-7.02 (m, 1H), 3.98 (dd, *J* = 4.7 Hz, 12.0 Hz, 1H), 3.19-3.02 (m, 2H), 2.48-2.30 (m, 5H); **^{13}C NMR** (125 MHz, $CDCl_3$) δ 198.1, 144.1, 138.7, 136.5, 133.4, 133.1, 130.6, 128.8, 127.8, 127.6, 126.9, 126.8, 126.2, 51.5, 30.4, 29.5, 19.9.

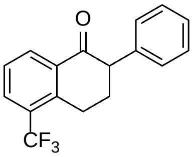

4g: white solid (59%). **^{1}H NMR** (500 MHz, $CDCl_3$) δ 8.31 (d, *J* = 7.9 Hz, 1H), 7.85 (d, *J* = 7.7 Hz, 1H), 7.44 (t, *J* = 7.8 Hz, 1H), 7.38-7.32 (m, 2H), 7.31-.26 (m, 1H), 7.20-7.16 (m, 2H), 3.85-3.80 (m, 2H), 3.33 (dt, *J* = 3.9 Hz, 17.9 Hz, 1H), 3.23-3.14 (m, 1H), 2.49-2.41 (m, 2H); **^{13}C NMR** (125 MHz, $CDCl_3$) δ 197.0, 142.4 (q, *J* = 1.4 Hz), 138.9, 134.4, 131.7 (q, *J* = 1.1 Hz), 130.7 (q, *J* = 5.6 Hz), 128.9 (q, *J* = 30.2 Hz), 128.7, 128.3, 127.2, 126.6, 124.1 (q, *J* = 274.0 Hz), 53.8, 30.1, 25.4 (q, *J* = 2.3 Hz); **^{19}F NMR** (470 MHz, $CDCl_3$) δ -60.9 (s, 3F); **HRMS** calcd for $C_{17}H_{14}F_3O^+$ $[M+H]^+$: 291.0997, found: 291.0991.

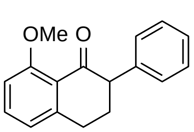

4h: yellow solid (55%). **^{1}H NMR** (500 MHz, $CDCl_3$): δ 7.41 (t, *J* = 8.0 Hz, 1H), 7.31 (t, *J* = 7.4 Hz, 2H), 7.16-7.27 (m, 3H), 6.85 (t, *J* = 7.6 Hz, 2H), 3.88 (s, 3H), 3.79 (dd, *J* = 9.0 Hz, *J* = 6.2 Hz, 1H), 2.97-3.11 (m, 2H), 2.30-2.43 (m, 2H); **^{13}C NMR** (100 MHz, $CDCl_3$): δ 197.4, 160.7, 146.6, 140.2, 134.0, 128.5, 128.4, 126.7, 122.6, 120.7, 110.1, 55.96, 55.70, 30.64, 29.48. **HRMS** calcd for $C_{17}H_{17}O_2^+$ $[M+H]^+$: 253.1729, found: 253.1223.

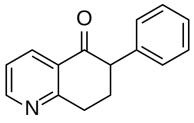

4i: pale yellow oil (35%). ^{1}**H NMR** (500 MHz, $CDCl_3$) δ 8.71 (dd, *J* = 4.75 Hz, 1.75 Hz, 1H), 8.34 (dd, *J* = 7.88 Hz, 1.73 Hz, 1H), 7.38-7.34 (m, 2H), 7.35-7.28 (m, 2H), 7.21-7.16 (m, 2H), 3.87-3.81 (m, 1H), 3.31-3.29 (m, 2H), 2.53-2.47 (m, 2H) ppm. ^{13}**C NMR** (125 MHz, $CDCl_3$) δ 197.55, 163.09, 153.39, 138.85, 135.72, 128.60, 128.38, 127.16, 122.39, 53.80, 31.76, 29.59 ppm. **HRMS** calcd for $C_{15}H_{14}NO^+$ $[M+H]^+$: 224.1075, found: 224.1070.

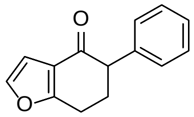

4j: yellow solid (4%). ^{1}**H NMR** (500 MHz, $CDCl_3$): δ 7.34-7.30 (m, 3H), 7.24-7.28 (m, 1H), 7.19-7.14 (m, 2H), 6.74 (d, *J* = 2.0 Hz, 1H), 3.71 (dd, *J* = 8.7 Hz, 5.8 Hz, 1H), 2.98-2.94 (m, 2H), 2.47-2.40 (m, 2H) ppm; ^{13}**C NMR** (125 MHz, $CDCl_3$): δ 194.1, 166.5, 142.9, 139.2, 128.6, 128.4, 127.0, 121.5, 107.0, 53.1, 31.0, 22.7. **HRMS** calcd for $C_{14}H_{13}O_2^+$ $[M+H]^+$: 213.0916, found: 213.0910.

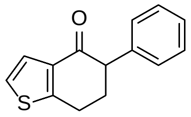

4k: pale yellow solid (45%). ^{1}**H NMR** (500 MHz, $CDCl_3$) δ 7.44 (d, *J* = 5.1 Hz, 1H), 7.32 (m, 2H), 7.25 (m, 1H), 7.17 (m, 2H), 7.09 (d, *J* = 5.1 Hz, 1H), 3.78-3.73 (m, 1H), 3.13-3.09 (m, 2H), 2.51-2.45 (m, 2H). ^{13}**C NMR** (125 MHz, $CDCl_3$): δ 193.1, 155.4, 139.4, 137.6, 128.7, 128.6, 127.1, 125.5, 123.6, 53.3, 32.9, 24.7. **HRMS** calcd for $C_{14}H_{13}OS^+$ $[M+H]^+$: 229.0687, found: 229.0682.

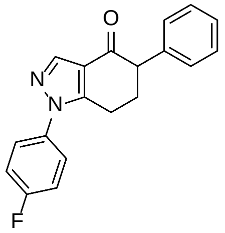

4l: pale yellow solid (48%) ^{1}H NMR (500 MHz, $CDCl_3$): δ 8.12 (s, 1H), 7.53-7.47 (m, 2H), 7.38-7.31 (m, 2H), 7.30-7.25 (m, 1H), 7.23-7.16 (m, 4H), 3.77 (t, *J* = 7.0 Hz, 2H), 3.08-2.92 (m, 2H), 2.48-2.41 (m, 1H) ppm. ^{13}**C NMR** (125 MHz, $CDCl_3$): δ 192.8, 162.1 (d, *J* = 249.0 Hz), 148.7, 139.1, 138.8, 134.8 (d, *J* = 3.1 Hz), 128.7, 128.4, 127.1, 125.4 (d, *J* = 8.7 Hz), 120.9, 116.4 (d, *J* = 23.1 Hz), 53.1, 31.8, 22.1 ppm. ^{19}**F NMR** (470 MHz, $CDCl_3$) δ -112.5 (s, 1F) ppm. **HRMS** calcd for $C_{19}H_{16}FN_2O^+$ $[M+H]^+$: 307.1247, found: 307.1242.

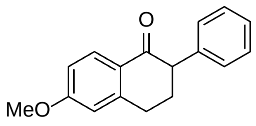

4q:[9] yellow solid (55%). ^{1}**H NMR** (400Hz, $CDCl_3$) δ 8.07 (d, *J* = 8.8 Hz, 1H), 7.32 (t, *J* = 7.1 Hz, 2H), 7.28-7.23 (m, 1H), 7.18 (d, *J* = 7.2 Hz, 2H), 6.85 (dd, *J* = 8.7, 2.2 Hz, 1H), 6.72 (d, *J* = 2.1Hz, 1H), 3.87 (s, 3H), 3.76 (t, *J* = 7.6 Hz, 1H), 3.11-2.95 (m, 2H), 2.44-2.38 (m, 2H); ^{13}**C NMR** (100 Hz, $CDCl_3$) δ 197.0, 163.6, 145.6, 140.0, 130.3, 128.5, 128.4, 126.8, 113.3, 112.6, 55.5, 54.0, 31.3, 29.0.

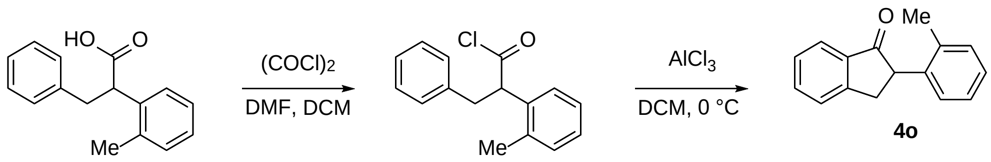

4o: To a stirred solution of 3-phenyl-2-(*o*-tolyl)propanoic acid (2.1g, 8.0 mmol) and oxalyl chloride (1.2 equiv, 4.8 mL, 2M in DCM) in DCM (10V), solution of DMF (10 mol %) in DCM was slowly added at 0 °C. The reaction mixture was stirred for 1 h at this temperature and was allowed to warm up to room temperature over 1h. Solvent and excess of oxalyl chloride was then removed under reduced pressure to afford 3-phenyl-2-(*o*-tolyl)propanoyl chloride, which was directly submitted to the next step. 3-Phenyl-2-(*o*-tolyl)propanoyl chloride was redissolved in DCM (10V) and $AlCl_3$ (3.4g, 25.6 mmol, 3.2 equiv) was subsequently added to this solution at 0 °C. The reaction mixture was stirred for 1 h at this temperature. Upon completion, the reaction was quenched with saturated aqueous NH_4Cl, extracted with DCM, dried over $MgSO_4$, filtered, and concentrated. The residue was purified by silica gel column chromatography (Hexanes → 10% EtOAc in Hexanes) to afford the 2-(*o*-tolyl)-2,3-dihydro-1*H*-inden-1-one **XX** as a white solid(1.2g, 67%).**^{1}H NMR** (500 MHz, $CDCl_3$) δ 7.81 (d, *J* = 7.7 Hz, 1H), 7.61 (t, *J* = 7.5 Hz, 1H), 7.48 (d, *J* = 7.8 Hz, 1H), 7.39 (t, *J* = 7.5 Hz, 1H), 7.21-7.06 (m, 3H), 6.94 (d, *J* = 7.6 Hz, 1H), 4.07 (dd, *J* = 4.4 Hz, 8.4 Hz, 1H), 3.66 (dd, *J* = 8.4 Hz, 17.4 Hz, 1H), 3.12 (dd, *J* = 4.4 Hz, 17.4 Hz, 1H), 2.33 (s, 3H). **^{13}C NMR** (125 MHz, $CDCl_3$) δ 206.7, 153.5, 138.6, 137.7, 136.7, 135.0, 130.8, 127.8, 127.5, 127.1, 126.6, 126.5, 124.3, 50.92, 35.6, 20.1. **HRMS** calcd for $C_{16}H_{15}O^+$ $[M+H]^+$: 223.1123, found: 223.1118.

3.2 Screening of reaction parameters for reduction of 2-phenyl-1-tetralone

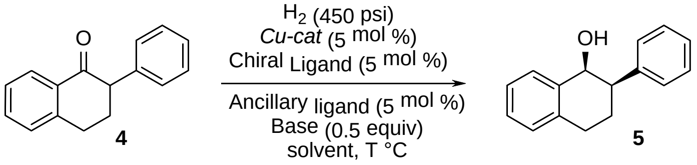

General procedure for screening of reaction parameters for reduction of 2-phenyl-1-tetralone. To a mixture of Cu-source (5 mol %), chiral ligand (5 mol %), ancillary ligand (5 mol %), and base (25 mol %) were added 2-pheny-1-tetralone **4a** (1 equiv) and *t*-AmylOH (0.5 M) under nitrogen. The reaction vessel was transferred into an autoclave and pressurized with hydrogen to 450 psi. The reaction mixture was stirred at this pressure and appropriate temperature for 24 h. After release of the hydrogen and purging with nitrogen, the reaction mixture was filtered through a plug of celite, the solvent was

removed under reduced pressure, and the crude mixtures were analyzed by HPLC to determine relative ration of alcohol and ketone and chiral HPLC or SFC analyses to determine *dr* and *er*.

Table S5. Screening of chiral ligands

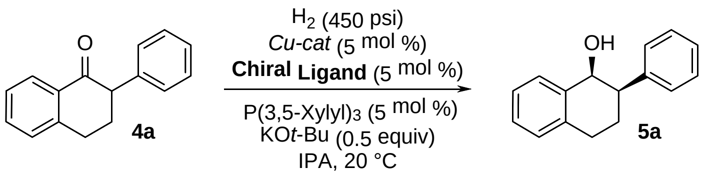

Entry	Cu-source	Ligand	% **5a**[a]	*dr*[b]	*er*[b]
1	CuCl	BDPP	11	>99:1	-
2	$Cu(OAc)_2$	CatASium KPh	14	78:22	-
3	$Cu(OAc)_2$	GSK-BoPhoz	70	88:12	83:17
4	$CuCl_2$	H-BIBOP	100	>99:1	77:23
5	$CuCl_2$	BABIPhos (**3a**)	100	>99:1	87:13
6[c]	$CuCl_2$	BABIPhos (**3a**)	100	98:2	91:9
7	$CuCl_2$	Me-BABIPhos (**3c**)	24	53:47	97:3
8	$CuCl_2$	*i*-Pr-BABIPhos (**3d**)	17	65:35	96:4
9	$CuCl_2$	2-MeO-Py-BABIPhos (**3e**)	35	89:11	76:24
10[c]	$CuCl_2$	NPiv-BABIPhos (**3b**)	100	>99:1	97:3
11[c]	$CuCl_2$	NH-BABIPhos (**3f**)	50	>99:1	96:4
12[c]	$CuCl_2$	NPh-BABIPhos (**3g**)	100	>99:1	86:14
13[c]	$CuCl_2$	NC(O)NHPh-BABIPhos (**3h**)	50	>99:1	96:4

[a]Determined by comparison of relative HPLC integration of alcohol to ketone at 220 nm. [b]Determined by chiral HPLC. [c]*t*-AmOH used as solvent.

Table S6. Screening of copper sources:

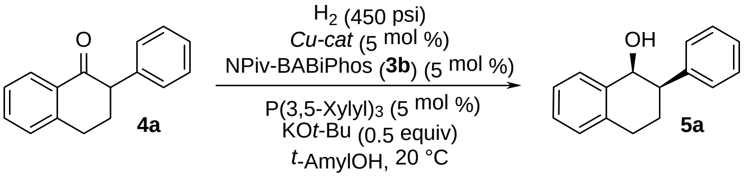

Entry	Cu-source	% **5a**[a]	*dr*[b]	*er*[b]
1	$CuCl_2$	99	>99:1	97:3
2	$Cu(OAc)_2$	99	>99:1	97:3
3	$Cu(acac)_2$	98	>99:1	97:3
4	$CuBr_2$	3	-	-
5	$Cu(eth)_2$	97	>99:1	97:3
6	$Cu(OTf)_2$	99	>99:1	97:3
7	$CuCl_2$(phen)	98	>99:1	97:3
8	$Cu(TFA)_2 \cdot H_2O$	99	>99:1	97:3
9	CuOAc	29	>99:1	97:3
10	CuCl	99	>99:1	97:3
11	CuBr	59	>99:1	97:3
12	CuI	0	-	-
13	Cu(thiophene-2-carboxylate)	97	>99:1	97:3
14	$Cu(PF_6)(NeCH)_4$	95	>99:1	97:3

[a]Determined by comparison of relative HPLC integration of alcohol to ketone at 220 nm. [b]Determined by chiral HPLC.

Table S7. Screening of ancillary ligands:

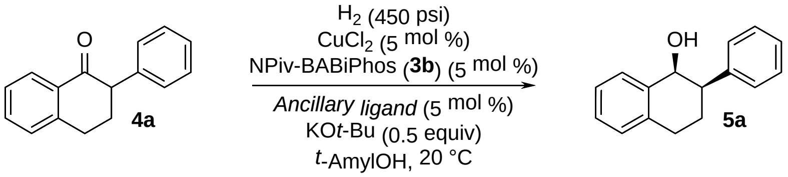

Entry	Cu-source	% **5a**[a]	*dr*[b]	*er*[b]
1	$P(3,5\text{-xylyl})_3$	99	>99:1	97:3
2	$P(4\text{-OMe-}3,5\text{-xylyl})_3$	76	>99:1	96:4
3	PPh_3	57	>99:1	90:10
4	P(*p*-tol)$_3$	9	-	-
5	P(*p*-CF$_3$-Ph)$_3$	3	-	-
6	P(*o*-furyl)$_3$	26	>99:1	89:11
7	$P(2,4,6\text{-MeO-Ph})_3$	0	-	-
8	$P(C_6F_5)_3$	0	-	-
9	P(3,5-bisCF$_3$-Ph)$_3$	1	-	-
10	P(*m*-tolyl)$_3$	75	>99:1	95:5
11	P(*o*-tolyl)$_3$	0	-	-
12	P(*p*-MeO-Ph)$_3$	60	>99:1	85:15
13	BINAP	0	-	-
14	none	0	-	-

[a]Determined by comparison of relative HPLC integration of alcohol to ketone at 220 nm. [b]Determined by chiral HPLC.

Table S8. Screening of base:

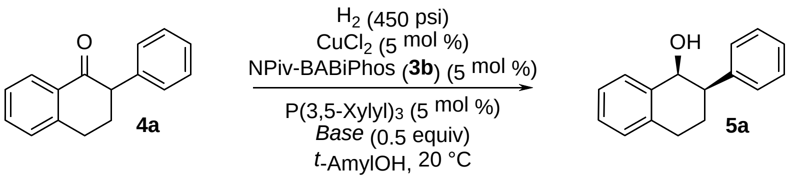

Entry	Base	% **5a**[a]	*dr*[b]	*er*[b]
1	KO*t*-Bu	99	>99:1	97:3
2	NaO*t*-Bu	97	>99:1	97:3
3	LiO*t*-Bu	0	-	-
4	NaO*t*-Amyl	97	>99:1	97:3
5	NaOMe	5	-	-

[a]Determined by comparison of relative HPLC integration of alcohol to ketone at 220 nm. [b]Determined by chiral HPLC.

Table S9. Screening of solvents:

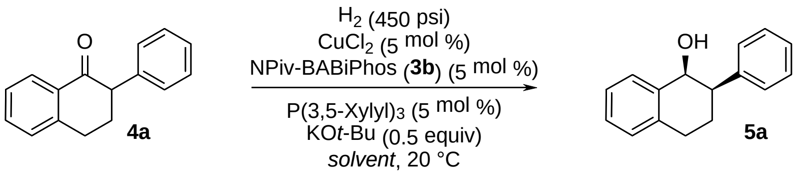

Entry	Base	% **5a**[a]	*dr*[b]	*er*[b]
1	*t*-BuOH	66	>99:1	96:4
2	*t*-AmylOH	99	>99:1	97:3
3	IPA	99	>99:1	92:8
4	MeOH	0	-	-
5	DCM	29	>99:1	91:9
6	NMP	4	-	-

[a]Determined by comparison of relative HPLC integration of alcohol to ketone at 220 nm. [b]Determined by chiral HPLC.

Table S10. Screening of reaction temperature:

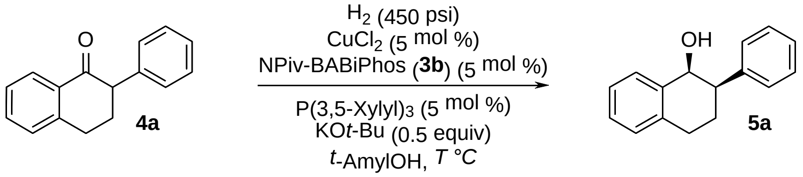

Entry	Temperature, °C	% **5a**[a]	*dr*[b]	*er*[b]
1	0	26	>99:1	97:3
2	10	33	>99:1	97:3
3	20	99	>99:1	97:3
4	40	99	>99:1	96:4
5[c]	40	99	>99:1	95:5

[a]Determined by comparison of relative HPLC integration of alcohol to ketone at 220 nm. [b]Determined by chiral HPLC. [c]*t*-BuOH was used as solvent.

3.3 Dynamic kinetic resolution of α-substituted tetralones

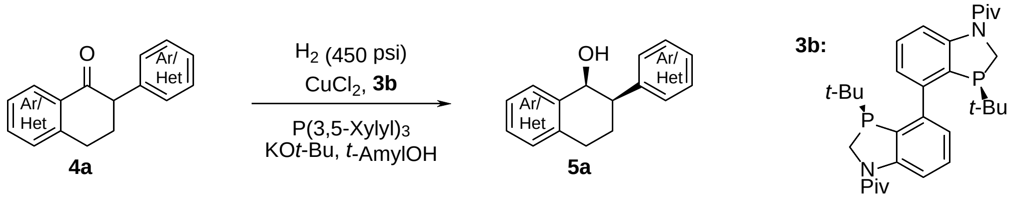

General procedure for Cu-catalyzed reduction. To a mixture of $CuCl_2$ (5 mol %), **3b** (5 mol %), *tris*(3,5-dimethylphenyl)phosphine (5 mol %), and KO*t*-Bu (25 mol %) were added corresponding tetralone **4** (1 equiv) and *t*-AmylOH (0.5 M) under nitrogen. The reaction vessel was transferred into an autoclave and pressurized with hydrogen to 450 psi. The reaction mixture was stirred at this pressure and 25 °C for 24 h. After release of the hydrogen and purging with nitrogen, the reaction mixture was filtered through a plug of celite, the solvent was removed under reduced pressure, and the product was purified by silica gel column chromatography to afford the alcohol **5**. The enantiomeric excess of the products was determined by chiral HPLC or SFC analysis.

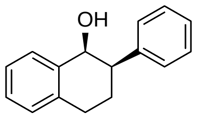

5a:[10] 205.6 mg, 92%, >99:1 *dr*, 97:3 *er*. Chiral HPLC: Lux Cellulose-3 (4.6 x 150 mm) column; heptane/denatured ethanol = 95:5, 1.5 mL/min, 10 °C, rt (major) = 12.55 min, rt (minor) = 20.04 min. **^{1}H NMR** (500 MHz, $CDCl_3$) δ 7.39-7.15 (m, 9H), 4.73 (d, J = 2.6 Hz, 1H), 3.07 (dt, J = 12.9 Hz, 2.6 Hz, 1H), 3.04-2.97 (m, 1H), 2.93-2.85 (m, 1H), 2.46-2.35 (m, 1H), 1.96-1.89 (m, 1H), 1.62 (s, 1H) ppm. **^{13}C NMR** (125 MHz, $CDCl_3$) δ 142.7, 137.7, 136.7, 130.5, 129.1, 128.7, 128.3, 128.1, 126.8, 126.2, 71.3, 46.1, 29.7, 21.6 ppm. **HRMS** calcd for $C_{16}H_{15}O^+$ $[M-H]^+$: 223.1123, found: 223.1118. $[\alpha]_D^{23}$ -129.48 (c 1.147, $CHCl_3$). $[\alpha]_D^{23}$ -129.48 (c 1.147, $CHCl_3$).

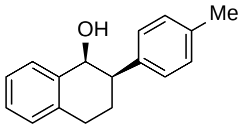

5b: 225.8 mg, 95%, >99:1 *dr*, 97:3 *er*. Chiral SFC: Lux Cellulose-4 column, 4.6 x 100 mm, CO_2, MeOH (1 to 50%, 3 mL/min), rt (major) = 5.25 min, rt (minor) = 4.68 min. **^{1}H NMR** (500 MHz, $CDCl_3$) δ 7.31-7.28 (m, 1H), 7.25-7.14 (m, 7H), 4.70 (t, J = 2.7 Hz, 1H), 3.06-2.95 (m, 2H), 2.92-2.83 (m, 1H), 2.42-2.32 (m, 4H), 1.93-1.86 (m, 1H), 1.63 (d, J = 3.1 Hz, 1H) ppm. **^{13}C NMR** (125 MHz, $CDCl_3$) δ 139.6, 137.8, 136.8, 136.3, 130.5, 129.4, 129.1, 128.13, 128.10, 126.2, 71.3, 45.7, 29.8, 21.7, 21.1 ppm. **HRMS** calcd for $C_{17}H_{17}O^+$ $[M-H]^+$: 237.1279, found: 237.1276. $[\alpha]_D^{23}$ -146.50 (c 1.727, $CHCl_3$).

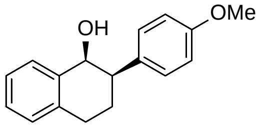

5c: 112.8 mg, 94%, >99:1 *dr*, 96:4 *er*. Chiral HPLC: IC-3 column, 4.6 x 250 mm, heptane/denatured ethanol = 90:10, 1.5 mL/min, 25 °C, rt (minor) = 9.98 min, rt (major) = 12.36 min. **^{1}H NMR** (500 MHz, $CDCl_3$) δ 7.34-7.30 (m, 1H), 7.27-7.15 (m, 5H), 6.94-6.90 (m, 2H), 4.73 (d, J = 2.7 Hz, 1H), 3.80 (s, 3H), 3.08-2.98 (m, 2H), 2.94-2.86 (m, 1H), 2.44-2.33 (m, 1H), 1.95-1.88 (m, 1H), 1.61 (brs, 1H) ppm. **^{13}C NMR** (125 MHz, $CDCl_3$) δ 158.4, 137.7, 136.7, 134.6, 130.5, 129.12, 129.07, 128.1, 126.1, 114.1, 71.3, 55.3, 45.2, 29.7, 21.8 ppm. **HRMS** calcd for $C_{17}H_{17}O_2^+$ $[M-H]^+$: 253.1229, found: 253.1227. $[\alpha]_D^{23}$ -128.28 (c 1.100, $CHCl_3$).

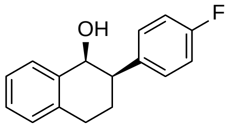

5d: 108.0 mg, 89%, >99:1 *dr*, 97:3 *er*. Chiral HPLC: OJ-3 column, 4.6 x 150 mm, heptane/denatured ethanol = 96:4, 2.0 mL/min, 25 °C, rt (minor) = 7.45 min, rt (major) = 10.67 min. **^{1}H NMR** (500 MHz, $CDCl_3$) δ 7.35-7.16 (m, 6H), 7.09-7.03 (m, 2H), 4.74 (d, J = 2.7 Hz, 1H), 3.10-2.99 (m, 2H), 2.96-2.86 (m, 1H), 2.45-2.34 (m, 1H), 1.96-1.89 (m, 1H), 1.55 (brs, 1H) ppm. **^{13}C NMR** (125 MHz, $CDCl_3$) δ 161.8, (d, J = 244.7 Hz), 138.4 (d, J = 3.2 Hz), 137.6, 136.6, 130.3, 129.6 (d, J = 7.8 Hz), 129.1, 128.2, 126.2, 115.3 (d, J = 21.0 Hz), 71.3 (J =

[10] Peach,P.; Cross, D. J.; Kenny, J. A.; Mann, I.; Houson, I.; Campbell, L.; Walsgroveb, T.; Wills, M. *Tetrahedron* **2006**, *62*, 1864-1876.

1.1 Hz), 55.2, 45.3, 29.6, 21.9 ppm. **^{19}F NMR** (470 MHz, $CDCl_3$) δ -116.4 ppm. **HRMS** calcd for $C_{16}H_{14}O_F^+$ $[M-H]^+$: 241.1029, found: 241.1024. $[\alpha]_D^{23}$ -140.34 (c 1.160, $CHCl_3$).

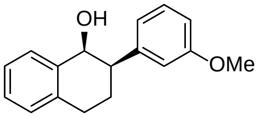

5e: 115.3 mg, 94%, >99:1 *dr*, 96:4 *er*. Chiral HPLC: IC-3 column, 4.6 x 250 mm, heptane/denatured ethanol = 90:10, 1.5 mL/min, 25 °C, rt (minor) = 9.98 min, rt (major) = 12.37 min. **^{1}H NMR** (500 MHz, $CDCl_3$) δ 7.35-7.15 (m, 5H), 6.94-6.87 (m, 2H), 6.84-6.79 (m, 1H), 4.76 (d, *J* = 2.6 Hz, 1H), 3.80 (s, 3H), 3.07 (dt, *J* = 12.9 Hz, 2.7 Hz, 1H), 3.04-2.97 (m, 2H), 2.94-2.85 (m, 1H), 2.46-2.35 (m, 1H), 1.97-1.91 (m, 1H), 1.66 (brs, 1H) ppm. **^{13}C NMR** (125 MHz, $CDCl_3$) δ 159.7, 144.3, 137.6, 136.7, 130.5, 129.6, 129.07, 128.1, 126.2, 120.5, 114.2, 111.9, 71.3, 55.2, 46.1, 29.7, 21.6 ppm. **HRMS** calcd for $C_{17}H_{17}O_2^+$ $[M-H]^+$: 253.1229, found: 253.1223. $[\alpha]_D^{23}$ -137.08 (c 1.263, $CHCl_3$).

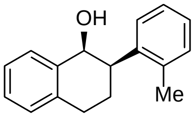

5f: 223.5 mg, 94%, >99:1 *dr*, 97:3 *er*. Chiral SFC: Lux Cellulose-3 column, 4.6 x 100 mm, CO_2, MeOH (1 to 50%, 3 mL/min), rt (major) = 4.32 min, rt (minor) = 4.71 min. **^{1}H NMR** (500 MHz, $CDCl_3$) δ 7.33-7.14 (m, 8H), 4.70 (d, *J* = 2.4 Hz, 1H), 3.30 (dt, *J* = 12.8 Hz, 2.6 Hz, 1H), 3.05-2.98 (m, 1H), 2.96-2.86 (m, 1H), 2.54-2.43 (m, 1H), 2.34 (s, 3H), 1.84-1.78 (m, 1H), 1.64 (brs, 1H) ppm. **^{13}C NMR** (125 MHz, $CDCl_3$) δ 140.4, 137.8, 136.8, 136.0, 130.7, 130.6 129.2, 128.1, 127.9, 126.7, 126.3, 126.1, 68.9, 42.1, 30.2, 22.1, 19.6 ppm. **HRMS** calcd for $C_{17}H_{17}O^+$ $[M-H]^+$: 237.1279, found: 237.1274. $[\alpha]_D^{23}$ -106.48 (c 1.030, $CHCl_3$).

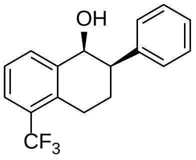

5g: 44.7 mg, 85%, >99:1 *dr*, 97:3 *er*. Chiral SFC: Lux Cellulose-4 column, 4.6 x 100 mm, CO_2, *i*-PrOH (1 to 50%, 3 mL/min), rt (minor) = 4.38 min, rt (major) = 4.68 min. **^{1}H NMR** (500 MHz, $CDCl_3$) δ 7.62 (d, *J* = 7.8 Hz, 1H), 7.53 (d, *J* = 7.7 Hz, 1H), 7.42-7.37 (m, 2H), 7.35-7.28 (m, 4H), 4.81 (d, *J* = 2.8 Hz, 1H), 3.37-3.29 (m, 1H), 3.12 (dt, *J* = 12.9 Hz, 2.8 Hz, 1H), 2.99-2.89 (m, 1H), 2.48-2.38 (m, 1H), 2.05-1.99 (m, 1H), 1.79 (brs, 1H) ppm. **^{13}C NMR** (125 MHz, $CDCl_3$) δ 141.8, 139.3, 135.8 (q, *J* = 1.5 Hz), 134.6 (q, *J* = 1.0 Hz), 128.8, 128.7 (q, *J* = 29.4 Hz), 128.1, 127.1, 126.0, 126.0 (q, *J* = 6.0 Hz), 124.6 (q, *J* = 274.1 Hz), 71.2, 45.2, 26.3 (q, *J* = 2.4 Hz), 20.9 ppm. **^{19}F NMR** (470 MHz, $CDCl_3$) δ -61.5. **HRMS** calcd for $C_{17}H_{14}OF_3^+$ $[M]^+$: 291.0997, found: 291.0992. $[\alpha]_D^{23}$ -71.88 (c 1.493, $CHCl_3$).

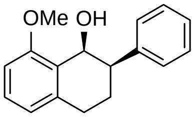

5h: 68.9 mg, 54%, >99:1 *dr*, 98:2 *er*. Chiral SFC: Lux Cellulose-2 column, 4.6 x 100 mm, CO_2, MeOH (1 to 50%, 3 mL/min), rt (minor) = 4.93 min, rt (major) = 5.77 min. **^{1}H NMR** (500 MHz, $CDCl_3$) δ 7.40-7.34 (m, 4H), 7.29-7.24 (m, 1H), 7.21 (t, *J* = 8.2

Hz, 1H), 6.81 (d, J = 7.7 Hz, 1H), 6.73 (d, J = 8.1 Hz, 1H), 5.11 (s, 1H), 3.83 (s, 3H), 3.05-2.85 (m, 3H), 2.48-2.38 (m, 1H), 2.00 (brs, 1H), 1.95-1.89 (m, 1H) ppm. **^{13}C NMR** (125 MHz, $CDCl_3$) δ 157.9, 143.1, 138.2, 128.6, 128.33, 128.31, 127.1, 126.5, 121.5, 107.6, 65.4, 55.4, 45.9, 30.3, 21.3 ppm. **HRMS** calcd for $C_{17}H_{17}O_2^+$ $[M-H]^+$: 253.1229, found: 253.1224. $[\alpha]_D^{23}$ -76.89 (c 1.170, $CHCl_3$).

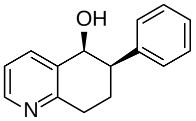

5i: 87.5 mg, 78%, >99:1 *dr*, 97:3 *er*. Chiral SFC: CCC column (ES Industries), 4.6 x 100 mm, CO_2, *i*-PrOH (1 to 50%, 3 mL/min), rt (minor) = 6.11 min, rt (major) = 6.33 min. **^{1}H NMR** (500 MHz, $CDCl_3$) δ 8.46-8.37 (m, 1H), 7.62 (d, J = 7.6 Hz, 1H), 7.53 (d, J = 7.7 Hz, 1H), 7.40-7.25 (m, 5H), 7.16-7.10 (m, 1H), 4.78 (s, 1H), 3.20-3.09 (m, 2H), 3.03-2.93 (m, 1H), 2.58-2.47 (m, 1H), 2.32 (brs, 1H), 2.06-1.99 (m, 1H). **^{13}C NMR** (125 MHz, $CDCl_3$) δ 157.1, 149.2, 141.8, 138.4, 132.9, 128.7, 128.2, 127.0, 121.4, 70.8, 45.7, 32.6, 21.4 ppm. **HRMS** calcd for $C_{15}H_{16}NO^+$ $[M+H]^+$: 226.1232, found: 226.1226. $[\alpha]_D^{23}$ -165.99 (c 1.217, $CHCl_3$).

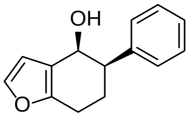

5j: 89.0 mg, 83%, >99:1 *dr*, 97:3 *er*. Chiral SFC: Lux Cellulose-2 column, 4.6 x 100 mm, CO_2, MeOH (1 to 50%, 3 mL/min), rt (minor) = 4.36 min, rt (major) = 4.85 min. **^{1}H NMR** (500 MHz, $CDCl_3$) δ 7.38-7.32 (m, 2H), 7.31-7.24 (m, 4H), 6.39 (d, J = 1.8 Hz, 1H), 4.65 (d, J = 3.2 Hz, 1H), 3.01 (dt, J = 12.9 Hz, 2.8 Hz, 1H), 2.84 (ddd, J = 16.8 Hz, 5.9 Hz, 1.4 Hz, 1H), 2.67 (ddd, J = 16.8 Hz, 11.6 Hz, 5.7 Hz, 1H), 2.48-2.38 (m, 1H), 1.99-1.92 (m, 1H), 1.52 (brs, 1H) ppm. **^{13}C NMR** (125 MHz, $CDCl_3$) δ 152.8, 142.1, 141.4, 128.6, 128.2, 126.9, 119.3, 109.9, 66.4, 46.7, 23.7, 22.1 ppm. **HRMS** calcd for $C_{14}H_{13}O_2^+$ $[M-H]^+$: 213.0916, found: 213.0910. $[\alpha]_D^{23}$ -162.79 (c 1.077, $CHCl_3$).

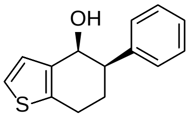

5k: 104.8 mg, 91%, >99:1 *dr*, 96:4 *er*. Chiral SFC: Lux Cellulose-1 column, 4.6 x 100 mm, CO_2, MeOH (1 to 50%, 3 mL/min), rt (minor) = 4.88 min, rt (major) = 5.27 min. **^{1}H NMR** (500 MHz, $CDCl_3$) δ 7.40-7.24 (m, 5H), 7.14 (d, J = 5.1 Hz, 1H), 6.99 (d, J = 5.2 Hz, 1H), 4.79 (d, J = 3.1 Hz, 1H), 3.14-3.04 (m, 2H), 2.89 (ddd, J = 16.9 Hz, 11.7 Hz, 5.4 Hz, 1H), 2.51-2.41 (m, 1H), 2.05-1.99 (m, 1H), 1.52 (brs, 1H) ppm. **^{13}C NMR** (125 MHz, $CDCl_3$) δ 142.2, 139.1, 137.1, 128.7, 128.2, 127.5, 126.9, 123.2, 67.6, 46.3, 25.6, 22.6 ppm. **HRMS** calcd for $C_{14}H_{13}OS^+$ $[M-H]^+$: 229.0687, found: 229.0682. $[\alpha]_D^{23}$ -20.9 (c 0.600, $CHCl_3$).

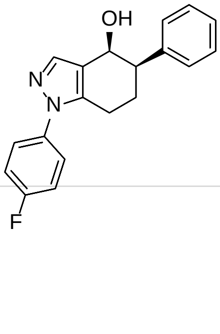

5l: 123.5 mg, 80%, >99:1 *dr*, 96:4 *er*. Chiral SFC: Lux Cellulose-4 column, 4.6 x 100 mm, CO_2, *i*-PrOH (1 to 50%, 3 mL/min), rt (minor) = 7.22 min, rt (major) = 7.44 min. **^{1}H NMR** (500 MHz, $CDCl_3$) δ 7.65 (s, 1H), 7.48-7.44 (m, 2H), 7.41-7.25

(m, 5H), 7.14 (t, *J* = 8.4 Hz, 2H), 4.84 (s, 1H), 3.08 (d, *J* = 12.9 Hz, 1H), 2.92-2.76 (m, 2H), 2.50-2.39 (m, 1H), 2.03-1.96 (m, 1H), 1.78 (brs, 1H) ppm. **^{13}C NMR** (125 MHz, $CDCl_3$) δ 161.5 (d, *J* = 247.2 Hz), 141.9, 139.4, 139.3, 135.96 (d, *J* = 3.0 Hz), 128.7, 128.3, 127.0, 125.2 (d, *J* = 8.5 Hz), 120.5, 116.1 (d, *J* = 22.9 Hz), 65.1, 46.6, 23.8, 22.0 ppm. **^{19}F NMR** (470 MHz, $CDCl_3$) δ -114.4 ppm **HRMS** calcd for $C_{19}H_{18}ON_2F^+$ $[M+H]^+$: 309.1403, found: 309.1398. $[\alpha]_D^{23}$ -178.04 (c 1.143, $CHCl_3$).

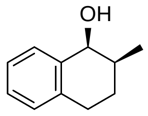

5m: 146.0 mg, 72%, 89:11 *dr*, 94:6 *er* (major diastereomer), 80:20 *er* (minor diastereomer). Chiral SFC: Lux Cellulose-1 column, 4.6 x 100 mm, CO_2, *i*-PrOH (1 to 50%, 3 mL/min), rt (major) = 3.94 min, rt (minor) = 4.04 min (major diastereomer), rt (major) = 4.16 min, rt (minor) = 4.29 min (minor diastereomer). For major isomer: **^{1}H NMR** (500 MHz, $CDCl_3$) δ 7.37-7.32 (m, 1H), 7.23-7.15 (m, 2H), 7.14-7.10 (m, 1H), 4.55 (d, *J* = 2.8 Hz, 1H), 2.90-2.71 (m, 2H), 1.95-1.85 (m, 1H), 1.81-1.71 (m, 1H), 1.68-1.61 (m, 1H), 1.60-1.46 (m, 1H), 1.13 (d, *J* = 6.9 Hz, 3H) ppm. **^{13}C NMR** (125 MHz, $CDCl_3$) δ δ 138.7, 136.7, 129.9, 129.1, 127.8, 126.1, 71.5, 34.2, 28.9, 24.8, 17.0 ppm. **HRMS** calcd for $C_{11}H_{13}O^+$ $[M-H]^+$: 161.0966, found: 161.0961.

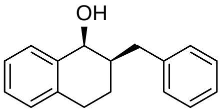

5n: 111.7 mg, 94%, 90:10 *dr*, 95:5 *er* (major diastereomer), 83:17 *er* (minor diastereomer). Chiral SFC: Lux Cellulose-1 column, 4.6 x 100 mm, CO_2, MeOH (1 to 50%, 3 mL/min), rt (minor) = 5.05 min, rt (major) = 4.79 min (major diastereomer), rt (minor) = 6.48 min, rt (major) = 5.16 min (minor diastereomer). For major isomer: **^{1}H NMR** (500 MHz, $CDCl_3$) δ 7.32-7.21 (m, 9H), 4.51 (d, *J* = 1.9 Hz, 1H), 2.95 (dd, *J* = 13.5 Hz, 7.9 Hz, 1H), 2.88 (dd, *J* = 17.1 Hz, 5.1 Hz, 1H), 2.78-2.68 (m, 2H), 2.08-1.98 (m, 1H), 1.88-1.78 (m, 1H), 1.74-1.67 (m, 1H), 1.52 (brs, 1H) ppm. **^{13}C NMR** (125 MHz, $CDCl_3$) δ 140.7, 138.6, 136.9, 130.0, 129.3, 129.1, 128.3, 128.0, 126.2, 125.9, 69.4, 41.8, 38.2, 29.2, 22.6 ppm. **HRMS** calcd for $C_{17}H_{17}O^+$ $[M-H]^+$: 237.1279, found: 237.1274. $[\alpha]_D^{23}$ -70.85 (c 1.043, $CHCl_3$).

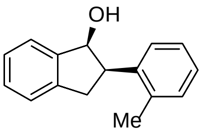

5o: 87.4 mg, 78%, >99:1 *dr*, 76:24 *er*. Chiral SFC: Lux Cellulose-2 column, 4.6 x 100 mm, CO_2, *i*-PrOH (1 to 50%, 3 mL/min), rt (minor) = 4.60 min, rt (major) = 4.83 min. **^{1}H NMR** (500 MHz, $CDCl_3$) δ 7.46-7.41 (m, 1H), 7.36-7.20 (m, 5H), 7.19-7.12 (m, 2H), 5.24 (d, *J* = 6.0 Hz, 1H), 4.00 (q, *J* = 7.1 Hz, 1H), 3.45 (dd, *J* = 15.8 Hz, 7.5 Hz, 1H), 3.16 (dd, *J* = 15.8 Hz, 7.7 Hz, 1H), 2.43 (s, 3H), 1.36 (s, 1H) ppm. **^{13}C NMR** (125 MHz, $CDCl_3$) δ 143.8, 143.2, 137.4, 137.1, 130.6, 128.7, 127.8, 127.0, 126.2, 125.3, 124.7, 76.0, 46.8, 35.9, 20.2 ppm. **HRMS** calcd for $C_{16}H_{15}O^+$ $[M-H]^+$: 223.1123, found: 223.1118. $[\alpha]_D^{23}$ -132.22 (c 1.117, $CHCl_3$).

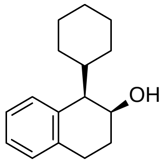

5p: 87.2 mg, 76%, >99:1 *dr*, 86:14 *er*. Chiral SFC: Lux Cellulose-4 column, 4.6 x 100 mm, CO_2, MeOH (1 to 50%, 3 mL/min), rt (minor) = 4.29 min, rt (major) = 4.95 min. **^{1}H NMR** (500 MHz, $CDCl_3$) δ 7.16-7.08 (m, 4H), 4.28-4.22 (m, 1H), 2.99-2.91 (m, 1H), 2.85-2.77 (m, 1H), 2.67 (t, J = 4.1 Hz, 1H), 2.03-1.86 (m, 4H), 1.78-1.72 (m, 1H), 1.69-1.61 (m, 2H), 1.50-1.22 (m, 5H), 1.17-1.07 (m, 1H), 0.87 (qd, J = 12.5 Hz, 3.3 Hz, 1H) ppm. **^{13}C NMR** (125 MHz, $CDCl_3$) δ 137.4, 136.5, 129.1, 128.4, 126.1, 125.3, 69.5, 50.0, 37.3, 33.7, 32.8, 28.5, 27.4, 27.0, 26.9, 26.5 ppm. **HRMS** calcd for $C_{16}H_{21}O_2^+$ $[M-H]^+$: 229.1592, found: 229.1589. $[\alpha]_D^{23}$ +70.56 (c 0.883, $CHCl_3$).

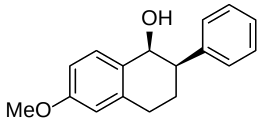

5q: 102.6 mg, 81%, >99:1 *dr*, 97:3 *er*. Chiral SFC: Lux Cellulose-3 column, 4.6 x 100 mm, CO_2, MeOH (1 to 50%, 3 mL/min), rt (minor) = 5.45 min, rt (major) = 5.79 min. **^{1}H NMR** (500 MHz, $CDCl_3$) δ 7.40-7.31 (m, 4H), 7.30-7.23 (m, 2H), 6.81-6.77 (m, 1H), 6.72-6.68 (s, 1H), 4.75 (s, 1H), 3.80 (s, 3H), 3.11-3.05 (m, 1H), 3.03-2.97 (m, 1H), 2.94-2.85 (m, 1H), 2.48-2.38 (m, 1H), 1.96-1.90 (m, 1H), 1.55 (brs, 1H) ppm. **^{13}C NMR** (125 MHz, $CDCl_3$) δ 159.7, 144.3, 137.6, 136.7, 130.5, 129.6, 129.07, 128.1, 126.2, 120.5, 114.2, 111.9, 71.3, 55.2, 46.1, 29.7, 21.6 ppm. **HRMS** calcd for $C_{17}H_{17}O_2^+$ $[M-H]^+$: 253.1229, found: 253.1223. $[\alpha]_D^{23}$ -168.51 (c 1.178, $CHCl_3$).

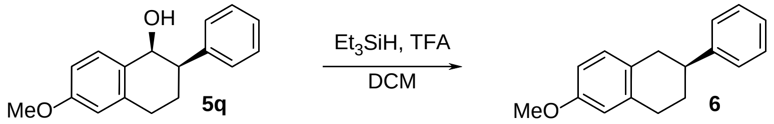

6: To a vial equipped with a magnetic stirrer was added alcohol (0.050 mg, 100 mol%) and DCM (2.00 mL). The solution was cooled to 0 ˚C in an ice-water bath, at which point triethylsilane (0.251 mL, 8 equiv) and TFA (0.061 mL, 4 equiv) were added sequentially. The reaction mixture was stirred at 0 ˚C. After 20 min, the reaction mixture was quenched with sat. aqueous $NaHCO_3$ solution and was diluted with DCM. Aqueous layer was extracted with DCM twice. Combined organics were dried over Na_2SO_4 and filtered. Concentration and purification via silica gel column chromatography (0-5% EtOAc/Hexanes) afforded product as a white solid (397 mg, 85% yield, 97:3 *er*). **^{1}H-NMR** (500 MHz, $CDCl_3$): δ 7.36-7.26 (m, 4H), 7.24-7.20 (m, 1H), 7.01 (d, J = 8.5 Hz, 1H), 6.72-6.67 (m, 2H), 3.79 (s, 2H), 3.01-2.84 (m, 5H), 2.15-2.09 (m, 1H), 1.97-1.87 (m, 1H). **^{13}C-NMR** (125 MHz, $CDCl_3$): δ 157.9,

146.8, 137.5, 129.9, 128.8, 128.5, 126.9, 126.2, 113.6, 112.2, 55.4, 41.1, 37.0, 30.4. **HRMS** calcd for $C_{16}H_{15}O^{+}$ [M-H]$^{+}$: 239.1436, found: 239.1430. $[\alpha]_D^{23}$ -78.17 (c 0.863, $CHCl_3$).

4. Details of computational studies

Optimizations of intermediates and transition states were performed using Gaussian 09[11] software with spin-restricted DFT using RB3LYP[12] functional and split basis set (6-31G(d) for C, P, O, H and LANL2DZ for Cu) in the gas phase. For all species, vibrational frequencies were also computed at the specified level of theory to obtain thermal Gibbs Free Energy corrections (at 298 K) and to characterize the stationary points as transition states (one and only one imaginary frequency) or minima (zero imaginary frequencies). Single point energy calculations were performed on optimized geometries in *tert*-butanol solvent using the PCM[13]-solvation model, with M06[14] functional and split basis set (6-311+G(d,p) for C, P, O, H; SDD for Cu). Obtained single-point energies were converted to the enthalpies and Gibbs free energies using corrections from gas-phase frequency analysis. Extensive conformational analysis of the transition states and intermediates was performed manually.

---

[11] Gaussian 09, Revision D.01, M. J. Frisch, G. W. Trucks, H. B. Schlegel, G. E. Scuseria, M. A. Robb, J. R. Cheeseman, G. Scalmani, V. Barone, B. Mennucci, G. A. Petersson, H. Nakatsuji, M. Caricato, X. Li, H. P. Hratchian, A. F. Izmaylov, J. Bloino, G. Zheng, J. L. Sonnenberg, M. Hada, M. Ehara, K. Toyota, R. Fukuda, J. Hasegawa, M. Ishida, T. Nakajima, Y. Honda, O. Kitao, H. Nakai, T. Vreven, J. A. Montgomery, Jr., J. E. Peralta, F. Ogliaro, M. Bearpark, J. J. Heyd, E. Brothers, K. N. Kudin, V. N. Staroverov, T. Keith, R. Kobayashi, J. Normand, K. Raghavachari, A. Rendell, J. C. Burant, S. S. Iyengar, J. Tomasi, M. Cossi, N. Rega, J. M. Millam, M. Klene, J. E. Knox, J. B. Cross, V. Bakken, C. Adamo, J. Jaramillo, R. Gomperts, R. E. Stratmann, O. Yazyev, A. J. Austin, R. Cammi, C. Pomelli, J. W. Ochterski, R. L. Martin, K. Morokuma, V. G. Zakrzewski, G. A. Voth, P. Salvador, J. J. Dannenberg, S. Dapprich, A. D. Daniels, O. Farkas, J. B. Foresman, J. V. Ortiz, J. Cioslowski, and D. J. Fox, Gaussian, Inc., Wallingford CT, 2013.

[12] (a) Lee, C.; Yang, W.; Parr, R. G. *Phys. Rev. B* **1988**, *37*, 785. (b) Becke, A. D. *J. Chem. Phys.* **1993**, *98*, 5648. (c) Becke, A. D. *J. Chem. Phys.* **1993**, *98*, 1372.

[13] Tomasi, J., Mennucci,B., Cammi, R. *Chem. Rev.*, **2005**, *105*, 2999.

[14] Zhao, Y.; Truhlar, D. *Theor. Chem. Acc.* **2008**, *120*, 215.

Coordinates and thermochemical data for computed intermediates and transition state

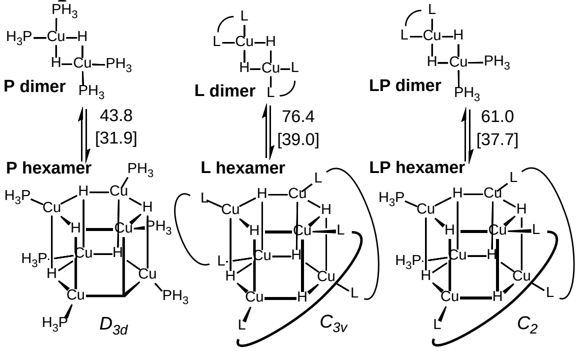

Truncated ligands were used for analysis of these equilibria. Thus, PH3 is used instead of PPh3 and the following simplified bidentate ligand:

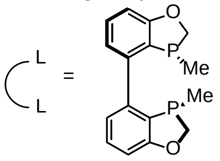

For equation balancing:

PH3

Zero-point correction= 0.024227 (Hartree/Particle)
Thermal correction to Energy= 0.027128
Thermal correction to Enthalpy= 0.028072
Thermal correction to Gibbs Free Energy= 0.003184
Sum of electronic and zero-point Energies= -343.116053
Sum of electronic and thermal Energies= -343.113153
Sum of electronic and thermal Enthalpies= -343.112208
Sum of electronic and thermal Free Energies= -343.137097
Electronic energy -343.13184738

P	-0.00001500	0.00001200	-0.12872700
H	-0.48977800	-1.09188700	0.64360000
H	-0.70071200	0.97000000	0.64375600
H	1.19071300	0.12170000	0.64354700

Model L

Zero-point correction= 0.282229 (Hartree/Particle)
Thermal correction to Energy= 0.300718
Thermal correction to Enthalpy= 0.301662
Thermal correction to Gibbs Free Energy= 0.235209
Sum of electronic and zero-point Energies= -1452.200279
Sum of electronic and thermal Energies= -1452.181790
Sum of electronic and thermal Enthalpies= -1452.180845
Sum of electronic and thermal Free Energies= -1452.247298
Electronic energy -1452.12083472

C	2.89728100	0.12821100	0.58577600
C	1.62149600	0.08247100	0.00104000
C	0.68373300	1.08362900	0.30078000
C	1.06297800	2.12231500	1.16751200
C	2.34108200	2.15135700	1.73150300
C	3.27471300	1.15411600	1.45221300
H	0.34439000	2.90086300	1.40616600
H	2.61032800	2.95932500	2.40670600
H	4.26907500	1.16090700	1.88672300
C	-0.68373300	1.08362900	-0.30078100
C	-1.62149600	0.08247100	-0.00104000
C	-1.06297800	2.12231500	-1.16751200
C	-2.89728100	0.12821100	-0.58577600
C	-2.34108200	2.15135700	-1.73150300
H	-0.34439000	2.90086300	-1.40616700
C	-3.27471300	1.15411600	-1.45221300
H	-2.61032800	2.95932400	-2.40670700
H	-4.26907500	1.16090700	-1.88672300
C	3.10596900	-1.94670300	-0.41667900
H	3.79696700	-2.36352500	-1.15393200
H	2.87601400	-2.71620300	0.33125000
C	-3.10596900	-1.94670300	0.41667900
H	-2.87601300	-2.71620300	-0.33124900
H	-3.79696700	-2.36352400	1.15393200
O	3.77890700	-0.85592000	0.24836500
O	-3.77890600	-0.85592000	-0.24836500
C	-2.18220400	-0.48475400	2.72014800
C	2.18220400	-0.48475500	-2.72014800
P	-1.50154900	-1.32516900	1.18612100
P	1.50154900	-1.32516900	-1.18612100
H	-3.11044300	0.06222000	2.52748400
H	-2.35803400	-1.23862500	3.49575900
H	-1.43002300	0.21608000	3.09667300
H	1.43002300	0.21607900	-3.09667300
H	3.11044300	0.06221900	-2.52748400
H	2.35803400	-1.23862600	-3.49575900

P-hexamer

Zero-point correction= 0.200592 (Hartree/Particle)
Thermal correction to Energy= 0.237365
Thermal correction to Enthalpy= 0.238309
Thermal correction to Gibbs Free Energy= 0.119104
Sum of electronic and zero-point Energies= -3246.581003
Sum of electronic and thermal Energies= -3246.544231
Sum of electronic and thermal Enthalpies= -3246.543286
Sum of electronic and thermal Free Energies= -3246.662491
Electronic energy -3239.81635384

Cu	0.00000000	1.55455400	0.98853300
H	-1.59663100	0.92181500	1.01579500
Cu	-1.34628300	-0.77727700	0.98853300
H	0.00000000	-1.84363100	1.01579500
Cu	1.34628300	-0.77727700	0.98853300
H	1.59663100	-0.92181500	-1.01579500
Cu	1.34628300	0.77727700	-0.98853300
H	0.00000000	1.84363100	-1.01579500
Cu	-1.34628300	0.77727700	-0.98853300
H	-1.59663100	-0.92181500	-1.01579500
Cu	0.00000000	-1.55455400	-0.98853300
H	1.59663100	0.92181500	1.01579500
P	0.00000000	3.56038500	2.11494600
P	-3.08338400	-1.78019200	2.11494600
P	3.08338400	-1.78019200	2.11494600
P	3.08338400	1.78019200	-2.11494600
P	-3.08338400	1.78019200	-2.11494600
P	0.00000000	-3.56038500	-2.11494600
H	-3.37845300	3.16511200	-2.00512100
H	-3.04739000	1.75941100	-3.53310500
H	-4.43029400	1.34327000	-2.00512100
H	3.37845300	3.16511200	-2.00512100
H	4.43029400	1.34327000	-2.00512100
H	3.04739000	1.75941100	-3.53310500
H	-4.43029400	-1.34327000	2.00512100
H	-3.37845300	-3.16511200	2.00512100
H	-3.04739000	-1.75941100	3.53310500
H	3.04739000	-1.75941100	3.53310500
H	3.37845300	-3.16511200	2.00512100
H	4.43029400	-1.34327000	2.00512100
H	0.00000000	-3.51882300	-3.53310500
H	1.05184100	-4.50838200	-2.00512100
H	-1.05184100	-4.50838200	-2.00512100
H	-1.05184100	4.50838200	2.00512100

H	0.00000000	3.51882300	3.53310500
H	1.05184100	4.50838200	2.00512100

P-dimer

Zero-point correction= 0.120061 (Hartree/Particle)
Thermal correction to Energy= 0.138279
Thermal correction to Enthalpy= 0.139223
Thermal correction to Gibbs Free Energy= 0.069582
Sum of electronic and zero-point Energies= -1768.402195
Sum of electronic and thermal Energies= -1768.383977
Sum of electronic and thermal Enthalpies= -1768.383033
Sum of electronic and thermal Free Energies= -1768.452674
Electronic energy -1766.18539711

Cu	-1.17562400	0.00003800	0.00032900
H	0.00070500	0.00102000	1.27458900
H	-0.00043800	-0.00159100	-1.27504400
Cu	1.17569800	0.00005700	-0.00092700
P	-2.41598400	-1.93627000	0.00105400
H	-3.29459500	-2.31556600	1.05126300
H	-3.29409400	-2.31517500	-1.04969400
H	-1.67108000	-3.14197000	0.00098900
P	-2.41589200	1.93607000	-0.00090100
H	-3.29510300	2.31554000	1.04873500
H	-1.67132400	3.14198900	-0.00107500
H	-3.29359200	2.31435000	-1.05222500
P	2.41567900	1.93612900	0.00122100
H	1.67057300	3.14171700	0.00102300
H	3.29164900	2.31415400	1.05405800
H	3.29626800	2.31673800	-1.04688800
P	2.41611900	-1.93604600	-0.00047100
H	1.67116600	-3.14172400	0.00114900
H	3.29295200	-2.31572000	-1.05201600
H	3.29594900	-2.31475900	1.04893400

L-hexamer

Zero-point correction= 0.893231 (Hartree/Particle)
Thermal correction to Energy= 0.966689
Thermal correction to Enthalpy= 0.967633
Thermal correction to Gibbs Free Energy= 0.774615
Sum of electronic and zero-point Energies= -5544.458848
Sum of electronic and thermal Energies= -5544.385390
Sum of electronic and thermal Enthalpies= -5544.384446
Sum of electronic and thermal Free Energies= -5544.577464
Electronic energy -5537.40710307

H	-1.77222900	-0.40256900	1.02698300
H	0.53748000	1.73608000	1.02698300
H	1.74865700	0.49392700	-1.03049300
H	-0.44657500	-1.76134500	-1.03049300
H	-1.30208200	1.26741800	-1.03049300
H	1.23475000	-1.33351100	1.02698300
Cu	0.43691100	1.54705200	-0.94355700
Cu	-0.43514400	-1.55080200	0.94742200
Cu	-1.12546200	1.15224700	0.94742200
Cu	1.56060600	0.39855500	0.94742200
Cu	1.12133100	-1.15190200	-0.94355700
Cu	-1.55824200	-0.39515000	-0.94355700
P	0.22409900	3.52593300	-2.07981400
C	-0.45868200	3.09341100	-3.78268800
C	-1.31188200	4.48251700	-1.76845600
C	1.49436300	4.83371200	-2.47388400
H	0.25621900	3.30615000	-4.58376800
H	-0.72030200	2.03083600	-3.80062400
O	-1.65553000	3.85475400	-4.03397500
C	-2.13338600	4.45803700	-2.90941600
C	-1.76468900	5.13058100	-0.60647300
H	2.37790300	4.34452500	-2.89873600
H	1.79299400	5.32490000	-1.54273800
H	1.11755300	5.58638100	-3.17439500
C	-3.39861900	5.04566300	-2.92358000
C	-3.04191500	5.71673300	-0.61850400
C	-0.90970700	5.34600000	0.60804900
C	-3.84634400	5.66786300	-1.76034500
H	-4.00234600	5.00710800	-3.82444100
H	-3.39042600	6.23687300	0.26885500
C	-1.00402900	4.56128800	1.77037600
C	-0.06222800	6.46680200	0.62106100
H	-4.82957500	6.13062100	-1.74502900
C	-0.27150200	4.92925300	2.91260300
P	-1.89736400	2.98721600	2.07712500
C	0.66749500	6.80489900	1.76418200
H	0.00000000	7.08987100	-0.26626100
C	0.56499100	6.04576300	2.92769500
O	-0.40800800	4.17202900	4.03719300
C	-3.64566800	3.52131100	2.44459300
C	-1.11132300	2.93940600	3.79028500
H	1.31472600	7.67782500	1.74967300
H	1.11304500	6.29822800	3.82959400
H	-3.68967600	4.36433100	3.14216200
H	-4.18957600	2.66736800	2.86333700
H	-4.12976100	3.80449000	1.50496800
H	-1.85314600	2.80370500	4.58380400
H	-0.38626800	2.12053600	3.82666200
P	-3.16559700	-1.56889100	-2.07981400
C	-2.44963100	-1.94393600	-3.78268800
C	-3.22603300	-3.37738200	-1.76845600
C	-4.93329900	-1.12270000	-2.47388400
H	-2.99131900	-1.43118300	-4.58376800
H	-1.39860500	-1.63921800	-3.80062400
O	-2.51055000	-3.36110800	-4.03397500
C	-2.79408000	-4.07658500	-2.90941600
C	-3.56086900	-4.09355600	-0.60647300
H	-4.95142000	-0.11293800	-2.89873600
H	-5.50799600	-1.10967200	-1.54273800
H	-5.39672400	-1.82536100	-3.17439500
C	-2.67036300	-5.46612200	-2.92358000
C	-3.42987900	-5.49274200	-0.61850400
C	-4.17491800	-3.46082900	0.60804900
C	-2.98534100	-6.16496300	-1.76034500
H	-2.33511000	-5.96968700	-3.82444100
H	-3.70607700	-6.05463200	0.26885500
C	-3.44817700	-3.15015900	1.77037600
C	-5.56930100	-3.28729200	0.62106100
H	-2.89448600	-7.24784500	-1.74502900
C	-4.13310700	-2.69975400	2.91260300
P	-1.63832300	-3.13677300	2.07712500
C	-6.22696300	-2.82438200	1.76418200
H	-6.14000800	-3.54493500	-0.26626100
C	-5.51828000	-2.53358500	2.92769500
O	-3.40907900	-2.43936000	4.03719300
C	-1.22671100	-4.91789700	2.44459300
C	-1.98993900	-2.43213700	3.79028500
H	-7.30655400	-2.70032600	1.74967300
H	-6.01094800	-2.18518900	3.82959400
H	-1.93478400	-5.37751900	3.14216200
H	-0.21522000	-4.96196300	2.86333700
H	-1.22990400	-5.47872300	1.50496800
H	-1.50150700	-3.00672400	4.58380400
H	-1.64330400	-1.39478600	3.82666200
P	2.94149800	-1.95704200	-2.07981400
C	2.90831400	-1.14947500	-3.78268800
C	4.53791500	-1.10513500	-1.76845600
C	3.43893600	-3.71101200	-2.47388400
H	2.73510000	-1.87496700	-4.58376800
H	2.11890700	-0.39161800	-3.80062400
O	4.16608000	-0.49364600	-4.03397500
C	4.92746600	-0.38145200	-2.90941600

C	5.32555800	-1.03702500	-0.60647300
H	2.57351700	-4.23158700	-2.89873600
H	3.71500200	-4.21522800	-1.54273800
H	4.27917100	-3.76102000	-3.17439500
C	6.06898200	0.42045900	-2.92358000
C	6.47179400	-0.22399100	-0.61850400
C	5.08462500	-1.88517100	0.60804900
C	6.83168500	0.49710000	-1.76034500
H	6.33745600	0.96257900	-3.82444100
H	7.09650300	-0.18224100	0.26885500
C	4.45220600	-1.41112900	1.77037600
C	5.63152900	-3.17951000	0.62106100
H	7.72406100	1.11722400	-1.74502900
C	4.40460900	-2.22949900	2.91260300
P	3.53568700	0.14955700	2.07712500
C	5.55946800	-3.98051700	1.76418200
H	6.14000800	-3.54493500	-0.26626100
C	4.95328900	-3.51217800	2.92769500
O	3.81708700	-1.73266900	4.03719300
C	4.87237900	1.39658600	2.44459300
C	3.10126200	-0.50726900	3.79028500
H	5.99182800	-4.97749900	1.74967300
H	4.89790300	-4.11303900	3.82959400
H	5.62446000	1.01318800	3.14216200
H	4.40479700	2.29459500	2.86333700
H	5.35966600	1.67423300	1.50496800
H	3.35465300	0.20301900	4.58380400
H	2.02957200	-0.72575000	3.82666200

L-dimer

Zero-point correction= 0.580878 (Hartree/Particle)
Thermal correction to Energy= 0.624124
Thermal correction to Enthalpy= 0.625068
Thermal correction to Gibbs Free Energy= 0.500618
Sum of electronic and zero-point Energies= -3300.330890
Sum of electronic and thermal Energies= -3300.287644
Sum of electronic and thermal Enthalpies= -3300.286699
Sum of electronic and thermal Free Energies= -3300.411149
Electronic energy -3297.90712648

C	4.44318800	1.89950900	-2.34179600
C	4.27038700	1.22213100	-1.12026200
C	5.27485400	0.34386100	-0.66569500
C	6.40661200	0.15613000	-1.48197200
C	6.54520400	0.82430900	-2.70000100
C	5.56889500	1.71254200	-3.14379500
H	7.18099300	-0.52951100	-1.15272600
H	7.42733800	0.64878000	-3.30988300
H	5.66062400	2.25065400	-4.08145500
C	5.27467000	-0.34452700	0.66526000
C	4.26988200	-1.22249800	1.11969400
C	6.40638800	-0.15715200	1.48167500
C	4.44232800	-1.89994800	2.34123700
C	6.54462900	-0.82539100	2.69971000
H	7.18101800	0.52825600	1.15252900
C	5.56799700	-1.71333700	3.14337500
H	7.42674300	-0.65013900	3.30970100
H	5.65945200	-2.25149100	4.08103500
C	2.28190400	2.65265300	-1.94197500
H	1.88480600	3.65413400	-1.75248500
H	1.54963000	2.07420900	-2.51487300
C	2.28087300	-2.65246900	1.94111500
H	1.54865200	-2.07388000	2.51393200
H	1.88357200	-3.65384900	1.75151200
O	3.48361300	2.78876800	-2.72341900
O	3.48243300	-2.78891400	2.72273100
C	3.17715900	-3.07025400	-0.81470800
C	3.17804400	3.07036500	0.81390200
P	2.67089400	-1.71284300	0.36077300
P	2.67148100	1.71302900	-0.36152900
Cu	1.17651200	0.00044600	-0.00025100
H	0.00008300	0.00043500	1.27412400
H	-0.00008500	0.00054500	-1.27456100
Cu	-1.17651200	0.00046900	-0.00020000
P	-2.67101100	-1.71270800	-0.36112900
P	-2.67135800	1.71313200	0.36131600
C	-3.17699000	-3.07021200	0.81436500
C	-4.27018500	-1.22229900	-1.11961700
C	-4.44296100	-1.89970400	-2.34114000
C	-5.27484400	-0.34433500	-0.66488600
C	-5.56883800	-1.71305100	-3.14297300
C	-6.40677300	-0.15691600	-1.48099800
C	-6.54534200	-0.82511000	-2.69902100
H	-5.66054600	-2.25117100	-4.08063000
H	-7.18130900	0.52848900	-1.15162600
H	-7.42761400	-0.64982600	-3.30877400
C	-4.27008200	1.22224600	1.12043500
C	-4.44256000	1.89958100	2.34203900
C	-5.27467500	0.34400200	0.66609600
C	-5.56806000	1.71259000	3.14432400
C	-6.40622300	0.15624900	1.48265900
C	-6.54449200	0.82438100	2.70075100
H	-5.65954100	2.25066800	4.08202700
H	-7.18069500	-0.52937100	1.15358600
H	-7.42646800	0.64883500	3.31085600
O	-3.48317400	-2.78866800	-2.72291600
O	-3.48288300	2.78881800	2.72344800
C	-2.28139400	-2.65221200	-1.94164100
H	-1.88398500	-3.65358200	-1.75221400
H	-1.54936600	-2.07355600	-2.51463800
C	-2.28139700	2.65279800	1.94164500
H	-1.88444100	3.65431400	1.75203200
H	-1.54890600	2.07441600	2.51432500
C	-3.17819600	3.07042300	-0.81404900
H	-3.69798800	2.62018200	-1.66568500
H	-2.27789600	3.56685700	-1.19204800
H	-3.83505000	3.80731800	-0.34065300
H	-3.83373200	-3.80732400	0.34115200
H	-2.27639200	-3.56633600	1.19205900
H	-3.69667400	-2.62018700	1.66618100
H	3.69705600	-2.62016200	-1.66635900
H	2.27665400	-3.56634600	-1.19266700
H	3.83378200	-3.80740800	-0.34139400
H	2.27765300	3.56680900	1.19167300
H	3.69764100	2.62015900	1.66567500
H	3.83500200	3.80724300	0.34062600

LP-dimer

Zero-point correction= 0.350382 (Hartree/Particle)
Thermal correction to Energy= 0.381178
Thermal correction to Enthalpy= 0.382123
Thermal correction to Gibbs Free Energy= 0.283771
Sum of electronic and zero-point Energies= -2534.366748
Sum of electronic and thermal Energies= -2534.335951
Sum of electronic and thermal Enthalpies= -2534.335007
Sum of electronic and thermal Free Energies= -2534.433358
Electronic energy -2532.04672803

C	-2.18911300	-2.94007400	-0.70272400
C	-2.01926700	-1.65875500	-0.14635900
C	-3.02349500	-0.68522900	-0.32505000
C	-4.14994100	-1.03333600	-1.09456400
C	-4.28415500	-2.30463100	-1.65613500
C	-3.30968600	-3.27977400	-1.46053300
H	-4.92404700	-0.28966700	-1.25545900
H	-5.16216100	-2.53629900	-2.25307900
H	-3.39904900	-4.27765200	-1.87695100
C	-3.03088500	0.67027600	0.31505600

C	-2.03197700	1.65082300	0.14526500
C	-4.16599800	1.01012200	1.07552800
C	-2.21511500	2.93044300	0.70142200
C	-4.31356800	2.28000200	1.63689100
H	-4.93617800	0.26095300	1.22955600
C	-3.34410200	3.26190900	1.45024500
H	-5.19801800	2.50516200	2.22677200
H	-3.44363000	4.25875100	1.86684400
C	-0.03635400	-3.30144600	0.09800400
H	0.33926000	-3.97619700	0.87280800
H	0.71178600	-3.20854000	-0.69619800
C	-0.05725800	3.30520400	-0.07743900
H	0.68110100	3.21223700	0.72596400
H	0.32569900	3.98432600	-0.84472200
O	-1.23258100	-3.87896200	-0.45699300
O	-1.26282900	3.87595300	0.46408100
C	-0.92348800	1.91952300	-2.51311900
C	-0.92635400	-1.90824700	2.52141900
P	-0.42947800	1.58798800	-0.74647800
P	-0.42358000	-1.58304900	0.75607000
Cu	1.06030700	0.00536600	0.00392500
H	2.25090700	0.73545200	1.04587900
H	2.24689900	-0.72633700	-1.04277100
P	4.66326900	-1.54290100	1.15246600
Cu	3.41184900	0.00422300	-0.00095800
P	4.65740800	1.55348600	-1.15872000
H	-1.45150500	-1.02654500	2.90222500
H	-0.02429700	-2.05065200	3.12604900
H	-1.57804000	-2.78290700	2.61610500
H	-0.01877500	2.06762500	-3.11235300
H	-1.44382000	1.03787500	-2.90059300
H	-1.57756800	2.79246600	-2.60714600
H	3.91003600	2.53445700	-1.85794300
H	5.49815000	1.21767400	-2.25404500
H	5.57126700	2.47189800	-0.57247100
H	3.91986600	-2.53171900	1.84487400
H	5.58439100	-2.45299500	0.56458900
H	5.49871400	-1.20712900	2.25181600

LP-hexamer

Zero-point correction= 0.663524 (Hartree/Particle)
Thermal correction to Energy= 0.724482
Thermal correction to Enthalpy= 0.725426
Thermal correction to Gibbs Free Energy= 0.559228
Sum of electronic and zero-point Energies= -4778.500611
Sum of electronic and thermal Energies= -4778.439653
Sum of electronic and thermal Enthalpies= -4778.438708
Sum of electronic and thermal Free Energies= -4778.604906
Electronic energy -4771.54503275

H	0.73872600	1.84915800	1.18350100
H	-1.81240900	0.52633800	2.56085800
H	-0.73872600	-1.84915800	1.18350100
H	1.30080200	-0.14482700	-0.52510800
H	1.81240900	-0.52633800	2.56085800
H	-1.30080200	0.14482700	-0.52510800
Cu	0.37046900	-1.32024100	2.33443600
Cu	0.02298300	1.21377000	-0.37021600
Cu	-0.37046900	1.32024100	2.33443600
Cu	-1.81389400	-0.39245800	1.15763200
Cu	-0.02298300	-1.21377000	-0.37021600
Cu	1.81389400	0.39245800	1.15763200
P	3.91374600	0.91592400	0.35684300
C	4.63204500	-0.69343100	-0.30703800
C	4.07692700	1.61686500	-1.33297100
C	5.35938100	1.65486800	1.27563500
H	5.45189800	-1.07142200	0.31133600
H	3.83434800	-1.44142000	-0.35106700
O	5.13418500	-0.48739600	-1.64212100
C	4.69888200	0.68454600	-2.18260400
C	3.65552000	2.85130400	-1.85565000
H	5.48101300	1.11781100	2.22281100
H	5.13427300	2.70123400	1.50400800
H	6.29040600	1.60396500	0.70154200
C	4.89843700	0.93795800	-3.54002000
C	3.85243700	3.10527200	-3.22362500
C	3.11212200	3.96473600	-1.00895900
C	4.46009400	2.15719100	-4.05160500
H	5.38262200	0.19319300	-4.16323800
H	3.54659400	4.06463600	-3.63055700
C	1.73803200	4.21891800	-0.86147500
C	4.02609700	4.86266800	-0.43263100
H	4.60176300	2.37731100	-5.10640100
C	1.31698700	5.36510900	-0.16547300
P	0.28422200	3.22803800	-1.38147600
C	3.58439700	5.98891000	0.26789200
H	5.08995400	4.68493400	-0.55924900
C	2.22442300	6.25946800	0.40340800
O	-0.02140300	5.60397900	-0.06869500
C	0.05291900	3.67168100	-3.17631200
C	-0.81411000	4.47984900	-0.49732800
H	4.31126100	6.66914900	0.70382400
H	1.86180600	7.13477500	0.93245800
H	0.12069500	4.75064600	-3.35034600
H	-0.92650500	3.30616400	-3.50394100
H	0.82112300	3.16198800	-3.76572800
H	-1.61833400	4.85297100	-1.13922300
H	-1.24912800	4.00997100	0.39026800
P	-0.28422200	-3.22803800	-1.38147600
C	0.81411000	-4.47984900	-0.49732800
C	-1.73803200	-4.21891800	-0.86147500
C	-0.05291900	-3.67168100	-3.17631200
H	1.61833400	-4.85297100	-1.13922300
H	1.24912800	-4.00997100	0.39026800
O	0.02140300	-5.60397900	-0.06869500
C	-1.31698700	-5.36510900	-0.16547300
C	-3.11212200	-3.96473600	-1.00895900
H	0.92650500	-3.30616400	-3.50394100
H	-0.82112300	-3.16198800	-3.76572800
H	-0.12069500	-4.75064600	-3.35034600
C	-2.22442300	-6.25946800	0.40340800
C	-4.02609700	-4.86266800	-0.43263100
C	-3.65552000	-2.85130400	-1.85565000
C	-3.58439700	-5.98891000	0.26789200
H	-1.86180600	-7.13477500	0.93245800
H	-5.08995400	-4.68493400	-0.55924900
C	-4.07692700	-1.61686500	-1.33297100
C	-3.85243700	-3.10527200	-3.22362500
H	-4.31126100	-6.66914900	0.70382400
C	-4.69888200	-0.68454600	-2.18260400
P	-3.91374600	-0.91592400	0.35684300
C	-4.46009400	-2.15719100	-4.05160500
H	-3.54659400	-4.06463600	-3.63055700
C	-4.89843700	-0.93795800	-3.54002000
O	-5.13418500	0.48739600	-1.64212100
C	-5.35938100	-1.65486800	1.27563500
C	-4.63204500	0.69343100	-0.30703800
H	-4.60176300	-2.37731100	-5.10640100
H	-5.38262200	-0.19319300	-4.16323800
H	-6.29040600	-1.60396500	0.70154200
H	-5.48101300	-1.11781100	2.22281100
H	-5.13427300	-2.70123400	1.50400800
H	-5.45189800	1.07142200	0.31133600
H	-3.83434800	1.44142000	-0.35106700
P	-0.02298300	-2.62042000	4.25032200
H	-1.31284300	-3.09831300	4.60000300
H	0.61774000	-3.88535000	4.32054800
H	0.33694000	-2.21490200	5.56371700
P	0.02298300	2.62042000	4.25032200

H	1.31284300	3.09831300	4.60000300
H	-0.61774000	3.88535000	4.32054800
H	-0.33694000	2.21490200	5.56371700

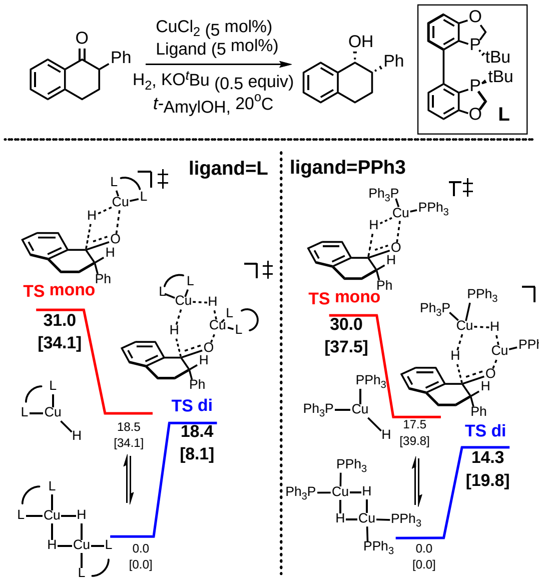

Starting Material

Zero-point correction= 0.257268 (Hartree/Particle)
Thermal correction to Energy= 0.270130
Thermal correction to Enthalpy= 0.271074
Thermal correction to Gibbs Free Energy= 0.216950
Sum of electronic and zero-point Energies= -693.113766
Sum of electronic and thermal Energies= -693.100904
Sum of electronic and thermal Enthalpies= -693.099959
Sum of electronic and thermal Free Energies= -693.154084
Electronic energy -693.02646554

C	-3.84029300	0.38830400	1.28019800
C	-2.91841700	1.31587100	0.79973700
C	-1.80279100	0.90676500	0.05898000
C	-1.63204000	-0.46825900	-0.19749900
C	-2.57085400	-1.39751900	0.28172300
C	-3.66785600	-0.97642400	1.02116100
H	-4.69953800	0.72935100	1.85204400
H	-3.06559500	2.37558400	0.99622300
H	-2.40787200	-2.44676400	0.05747100
H	-4.38784600	-1.69956700	1.39397800
C	-0.47385200	-0.97985400	-0.98696800
O	-0.37949100	-2.16312600	-1.28481300
C	-0.79909100	1.91798400	-0.44903600
H	-1.31292100	2.85384500	-0.70060400
H	-0.10007100	2.16159000	0.36272700
C	-0.02210400	1.39889700	-1.66620000
H	0.73638500	2.12475200	-1.97917200
H	-0.71523500	1.29888700	-2.51166000
C	0.61371800	0.02004600	-1.41110800
H	0.99802200	-0.37314200	-2.36125100
C	1.78366800	-0.00125200	-0.41038800
C	2.44050100	1.16729000	0.00004700
C	2.26515100	-1.23019000	0.07032500
C	3.53226700	1.11322000	0.87014100
H	2.11613100	2.13670500	-0.36355900
C	3.35392600	-1.28516000	0.93902400
H	1.78308200	-2.14943800	-0.24629500
C	3.99217600	-0.11264000	1.34743400

H	4.02157500	2.03591300	1.17166400
H	3.70485200	-2.25002700	1.29599500
H	4.84030900	-0.15515200	2.02552000

Ligand=L

Dimeric Cu hydride_L

Zero-point correction= 0.924321 (Hartree/Particle)
Thermal correction to Energy= 0.982609
Thermal correction to Enthalpy= 0.983553
Thermal correction to Gibbs Free Energy= 0.833213
Sum of electronic and zero-point Energies= -3771.729003
Sum of electronic and thermal Energies= -3771.670715
Sum of electronic and thermal Enthalpies= -3771.669771
Sum of electronic and thermal Free Energies= -3771.820111
Electronic energy -3769.43646444

C	4.50375400	2.33210400	-1.92961300
C	4.30992700	1.43576400	-0.85847500
C	5.31059000	0.47964300	-0.57682000
C	6.45663700	0.46064700	-1.39731200
C	6.61779100	1.35661300	-2.45464700
C	5.64403200	2.31141000	-2.73147200
H	7.22476000	-0.28087100	-1.20249900
H	7.51114300	1.30410000	-3.07106800
H	5.74446200	3.02197000	-3.54522400
C	5.31071900	-0.47957800	0.57649900
C	4.31011800	-1.43568700	0.85837800
C	6.45692500	-0.46056500	1.39677300
C	4.50410700	-2.33197400	1.92953300
C	6.61826400	-1.35649600	2.45410900
H	7.22501400	0.28094300	1.20178900
C	5.64453600	-2.31126200	2.73117200
H	7.51173300	-1.30397400	3.07035900
H	5.74510100	-3.02177100	3.54495300
C	2.31924300	2.96133500	-1.47643000
H	1.87625100	3.90211300	-1.14115200
H	1.63340500	2.47435800	-2.17796800
C	2.31950300	-2.96123700	1.47670800
H	1.63366600	-2.47435700	2.17831000
H	1.87658000	-3.90204900	1.14143800
O	3.54234300	3.26741400	-2.16752600
O	3.54268700	-3.26719300	2.16772400
C	3.03127000	-2.90150800	-1.42218400
C	1.67860700	-3.51792900	-1.83025900
H	0.93443100	-2.73930600	-2.03055200
H	1.27821100	-4.18812000	-1.05955800
H	1.80688500	-4.11292700	-2.74412500
C	4.05439000	-4.00672700	-1.11419300
H	4.19845900	-4.63477700	-2.00392900
H	3.72990700	-4.66079400	-0.29756600
H	5.02835400	-3.58786100	-0.84090400
C	3.54222900	-2.01649300	-2.57516800
H	3.67155600	-2.62957500	-3.47731300
H	4.50862700	-1.55818800	-2.34287900
H	2.83153800	-1.21633900	-2.80924900
C	3.03147900	2.90143600	1.42241200
C	1.67886500	3.51774000	1.83083000
H	1.80727900	4.11262300	2.74475100
H	0.93477000	2.73905400	2.03116400
H	1.27829100	4.18801000	1.06029400
C	3.54271100	2.01632000	2.57519600
H	3.67212100	2.62929700	3.47740000
H	4.50911600	1.55814100	2.34269600
H	2.83213200	1.21606700	2.80927800
C	4.05446000	4.00673300	1.11427000
H	4.19866700	4.63475700	2.00400300
H	3.72974900	4.66080500	0.29773600

H	5.02840300	3.58796700	0.84075900
P	2.67690300	-1.76282800	0.07197900
P	2.67681700	1.76288100	-0.07177200
Cu	1.18451200	-0.00025400	-0.00032200
H	0.00014900	0.00031900	1.27108800
H	0.00014500	0.00006600	-1.27125500
Cu	-1.18455400	-0.00036900	-0.00005000
P	-2.67698900	-1.76284700	-0.07211400
P	-2.67676700	1.76288600	0.07157200
C	-3.03116800	-2.90162400	1.42201600
C	-1.67841800	-3.51794900	1.82997100
H	-1.27797500	-4.18799900	1.05917400
H	-0.93432900	-2.73926600	2.03033800
H	-1.80660300	-4.11307300	2.74376700
C	-4.05421700	-4.00690600	1.11403400
H	-3.72969600	-4.66095800	0.29741200
H	-4.19825700	-4.63496000	2.00377300
H	-5.02820900	-3.58810700	0.84073900
C	-3.54211400	-2.01669300	2.57506500
H	-2.83145600	-1.21651100	2.80914600
H	-4.50855200	-1.55843100	2.34284700
H	-3.67136000	-2.62982900	3.47718400
C	-3.03170300	2.90156400	-1.42244300
C	-3.54297400	2.01649100	-2.57524500
H	-2.83231100	1.21636400	-2.80951000
H	-4.50927400	1.55813900	-2.34264900
H	-3.67260200	2.62953100	-3.47737500
C	-1.67918100	3.51801600	-1.83094600
H	-0.93497300	2.73942300	-2.03126200
H	-1.80769500	4.11283400	-2.74489400
H	-1.27865800	4.18837700	-1.06046100
C	-4.05476000	4.00673900	-1.11411500
H	-3.73003200	4.66078700	-0.29757000
H	-4.19910700	4.63480700	-2.00379400
H	-5.02862900	3.58784900	-0.84054100
C	-4.31031000	-1.43559300	-0.85827100
C	-4.50447200	-2.33181800	-1.92944700
C	-5.31084200	-0.47946100	-0.57620700
C	-5.64501700	-2.31102700	-2.73092600
C	-6.45715400	-0.46036700	-1.39632400
C	-6.61867100	-1.35624400	-2.45368000
H	-5.74571700	-3.02148500	-3.54473300
H	-7.22518300	0.28117000	-1.20121200
H	-7.51222100	-1.30365400	-3.06980700
C	-4.30969600	1.43578000	0.85864000
C	-4.50325700	2.33207700	1.92986500
C	-5.31047100	0.47973500	0.57713000
C	-5.64339000	2.31142600	2.73193100
C	-6.45636800	0.46077200	1.39784100
C	-6.61726800	1.35670700	2.45523900
H	-5.74361800	3.02195200	3.54573700
H	-7.22456800	-0.28069800	1.20315000
H	-7.51051300	1.30423400	3.07181800
O	-3.54311800	-3.26705900	-2.16782000
O	-3.54172000	3.26726800	2.16767700
C	-2.31981000	-2.96109000	-1.47701700
H	-1.87674400	-3.90191500	-1.14197200
H	-1.63415500	-2.47405600	-2.17869400
C	-2.31881400	2.96128000	1.47619800
H	-1.87597700	3.90208200	1.14078000
H	-1.63273900	2.47435000	2.17752400

Monomeric Cu hydride_L

Zero-point correction= 0.460392 (Hartree/Particle)
Thermal correction to Energy= 0.489382
Thermal correction to Enthalpy= 0.490326
Thermal correction to Gibbs Free Energy= 0.402728
Sum of electronic and zero-point Energies= -1885.845383
Sum of electronic and thermal Energies= -1885.816394
Sum of electronic and thermal Enthalpies= -1885.815450
Sum of electronic and thermal Free Energies= -1885.903047
Electronic energy -1884.68958740

C	-2.45979500	0.89843500	-1.74453100
C	-1.49799200	0.74010100	-0.72716400
C	-0.52324900	1.74508400	-0.53970000
C	-0.56783900	2.87427100	-1.37956300
C	-1.53807300	3.00817700	-2.37495000
C	-2.49992300	2.02162600	-2.57031900
H	0.18138000	3.64940700	-1.25337800
H	-1.53578000	3.88954300	-3.01039600
H	-3.26088700	2.09938700	-3.33968200
C	0.52330300	1.74693300	0.53397900
C	1.49804300	0.74258000	0.72494900
C	0.56788100	2.87904700	1.36988400
C	2.45972600	0.90443500	1.74187200
C	1.53805200	3.01641300	2.36486100
H	-0.18128900	3.65377400	1.24090200
C	2.49983000	2.03050100	2.56374100
H	1.53575700	3.90000600	2.99720500
H	3.26075700	2.11091600	3.33286700
C	-3.02726000	-1.28483500	-1.20323000
H	-3.93820400	-1.73190000	-0.79963400
H	-2.57975800	-1.98670800	-1.91718900
C	3.02790000	-1.28052000	1.20759800
H	2.58132200	-1.98081300	1.92366700
H	3.93911200	-1.72783700	0.80490100
O	-3.39019800	-0.08331400	-1.90505400
O	3.39006900	-0.07676100	1.90600900
C	2.79749600	-0.52597200	-1.68043300
C	3.38525900	-1.88070000	-2.12714700
H	2.60454700	-2.63515400	-2.27346200
H	4.10744500	-2.27937000	-1.40542700
H	3.91550000	-1.74933500	-3.07940000
C	3.92324600	0.49266700	-1.43703100
H	4.49974900	0.63177100	-2.36141900
H	4.62023800	0.16274500	-0.65896100
H	3.52631000	1.46909600	-1.14058700
C	1.84071800	-0.00608200	-2.77060600
H	2.39864700	0.14720900	-3.70375900
H	1.38306900	0.94918300	-2.49475800
H	1.03749300	-0.72277400	-2.97664700
C	-2.79780400	-0.52032600	1.68241100
C	-3.38371600	-1.87400500	2.13469600
H	-3.91488600	-1.73931000	3.08597200
H	-2.60188600	-2.62652900	2.28488900
H	-4.10466200	-2.27703400	1.41415900
C	-1.84190400	0.00556000	2.77041100
H	-2.40024800	0.16243000	3.70271900
H	-1.38529900	0.96008100	2.49033500
H	-1.03788200	-0.70921800	2.97993200
C	-3.92500000	0.49571400	1.43477800
H	-4.50130600	0.63828100	2.35876400
H	-4.62193400	0.16143200	0.65853800
H	-3.52943100	1.47130200	1.13376800
P	1.75989300	-0.87172000	-0.11750500
P	-1.75978100	-0.87135300	0.12092100
Cu	0.00030700	-2.40967300	0.00330800
H	-0.00083900	-3.95799500	0.00424500

TS di_L

Zero-point correction= 1.179107 (Hartree/Particle)
Thermal correction to Energy= 1.253036
Thermal correction to Enthalpy= 1.253980
Thermal correction to Gibbs Free Energy= 1.065910
Sum of electronic and zero-point Energies= -4464.806967
Sum of electronic and thermal Energies= -4464.733038

Sum of electronic and thermal Enthalpies= -4464.732094
Sum of electronic and thermal Free Energies= -4464.920164
Electronic energy -4462.44934788

C	-3.82787600	-3.54302100	1.43373100
C	-4.10670400	-2.24542800	0.96073900
C	-5.23663700	-2.04341400	0.14206100
C	-6.05102200	-3.15353500	-0.15541000
C	-5.75400800	-4.42726800	0.33243500
C	-4.63628300	-4.63953200	1.13482500
H	-6.91816600	-3.01013200	-0.79245000
H	-6.39751700	-5.26423800	0.07486200
H	-4.38150600	-5.62010600	1.52326900
C	-5.67843000	-0.71847700	-0.39831800
C	-4.89769200	0.11535600	-1.22752100
C	-7.00120900	-0.32821400	-0.11413400
C	-5.47399500	1.29334900	-1.74235600
C	-7.54106100	0.85126200	-0.62939300
H	-7.60420700	-0.95913600	0.53131500
C	-6.78442200	1.67644700	-1.45577700
H	-8.56183900	1.12876500	-0.38064400
H	-7.18101500	2.59509600	-1.87544600
C	-1.87263800	-2.56140100	2.19815300
H	-1.46197800	-2.42288600	3.20057400
H	-1.05026000	-2.75673500	1.50070600
C	-3.33830200	1.68043200	-2.56857000
H	-2.78542700	2.38493400	-1.94094600
H	-2.96932700	1.74457300	-3.59498000
O	-2.73737900	-3.71212500	2.23070300
O	-4.72494100	2.06534200	-2.57599100
C	-3.24006300	-1.18106300	-3.35593400
C	-1.97010400	-0.90170200	-4.18270500
H	-1.06562200	-0.99982700	-3.57282400
H	-1.97267000	0.10017100	-4.62681900
H	-1.90044100	-1.62270600	-5.00813900
C	-4.49241300	-0.94695100	-4.21525100
H	-4.46985400	-1.60533400	-5.09500300
H	-4.55968400	0.08533800	-4.57601900
H	-5.40860900	-1.16646400	-3.65806000
C	-3.20234300	-2.63898800	-2.86058300
H	-3.16214100	-3.32139700	-3.72055100
H	-4.08942300	-2.89523500	-2.27345600
H	-2.31965900	-2.82947700	-2.23947700
C	-3.55639800	-0.29248700	3.15998800
C	-2.36604900	0.28416200	3.95251700
H	-2.74302700	0.84708600	4.81660900
H	-1.76592900	0.96899300	3.34419700
H	-1.70611200	-0.50119700	4.34027700
C	-4.47660000	0.86628800	2.73058400
H	-4.85761400	1.38059100	3.62336900
H	-5.33885300	0.51368100	2.15546600
H	-3.93530600	1.59984200	2.12358800
C	-4.33654200	-1.29606200	4.02398700
H	-4.69414800	-0.79847600	4.93597400
H	-3.71842400	-2.14653100	4.33157200
H	-5.21026500	-1.69033300	3.49494700
P	-3.15399300	-0.03480100	-1.81198900
P	-2.82803400	-1.06771000	1.56933900
Cu	-1.58554200	0.14075800	-0.00801000
H	-1.33916000	1.74574600	0.12964000
C	0.29761300	2.87562400	0.38432800
C	-0.29709900	3.44851300	1.63050600
C	-1.40218600	4.32498200	1.56626300
C	0.27426800	3.15019900	2.87654800
C	-1.90240000	4.87937500	2.74665900
C	-0.23306000	3.71017800	4.04657900
H	1.11916000	2.47237800	2.90418300
C	-1.32551700	4.57982700	3.98281600
H	-2.75290100	5.55599500	2.69510700
H	0.22248200	3.47536200	5.00545800
H	-1.72357600	5.02675700	4.89025600
C	0.07017400	3.63896800	-0.95091400
H	-0.36129100	2.90639000	-1.63677100
C	-2.01236500	4.60424700	0.22123300
H	-2.61924000	3.73631300	-0.07606000
H	-2.67850600	5.47391700	0.27149700
C	-0.90561700	4.83953600	-0.81605300
H	-1.33458900	5.06567100	-1.79999800
H	-0.33618100	5.72840000	-0.51585200
C	1.38328100	4.11693100	-1.56063300
C	2.37553000	4.74235900	-0.78971900
C	1.58993300	4.01204400	-2.94178800
C	3.53941600	5.23345500	-1.38063300
H	2.23846500	4.83981300	0.28407200
C	2.75088900	4.50923200	-3.54057100
H	0.82781800	3.53914800	-3.55821200
C	3.73265700	5.11969600	-2.76024100
H	4.29729200	5.70825700	-0.76241700
H	2.88587200	4.41690100	-4.61546900
H	4.63885400	5.50503700	-3.22023000
O	1.24886200	2.06388500	0.45404800
H	-0.10499200	-0.69471600	-0.04570400
Cu	1.40730400	-0.10232800	0.11621700
P	3.09615100	-0.34733800	1.73688600
P	2.60064400	-1.12596600	-1.70893200
C	4.82419500	-0.59563200	1.15368000
C	3.64513400	1.31934500	2.40616500
C	2.90275900	-1.43728800	3.29435100
C	3.53503800	-2.60813400	-1.13054400
C	1.37691900	-2.30909500	-2.51270500
C	3.58261200	-0.42075500	-3.19725500
C	5.39486400	-1.58236200	0.32403600
C	5.65129500	0.41794500	1.67755500
H	3.30039700	2.09246000	1.71295200
H	3.25654400	1.53413100	3.40390000
O	5.08236100	1.35500700	2.48149200
C	1.66509200	-0.90553800	4.04415800
C	4.13424300	-1.40991400	4.21470800
C	2.62922100	-2.87906600	2.82522200
C	2.97525600	-3.77455800	-1.68901900
C	4.64771700	-2.73330400	-0.27161600
H	1.12211900	-2.03176300	-3.53757300
H	0.46631800	-2.31934300	-1.90449900
O	1.92730300	-3.63790000	-2.54333600
C	2.59000800	0.43674000	-4.00705000
C	4.68922800	0.49465100	-2.63884800
C	4.19580400	-1.51241600	-4.08840300
C	6.77811000	-1.52167900	0.06900100
C	7.02113000	0.47496900	1.42052800
H	0.78829100	-0.85219500	3.38940800
H	1.83044700	0.09327500	4.46430900
H	1.42763700	-1.57515000	4.88143900
H	3.94421400	-2.02721000	5.10325500
H	4.37319100	-0.39774800	4.55839300
H	5.02060800	-1.81222500	3.71361100
H	2.43527700	-3.51851400	3.69691000
H	3.48232800	-3.30218500	2.28550800
H	1.75432800	-2.93059200	2.16749200
C	3.47463000	-5.05004700	-1.42277400
C	5.15034100	-4.02167700	-0.00875900
H	3.12966100	0.95761800	-4.80891700
H	2.11232500	1.19859200	-3.38201800
H	1.80629200	-0.16545200	-4.48129100
H	5.22208500	0.97305900	-3.47161400
H	5.42431800	-0.06234600	-2.04982600
H	4.27471400	1.29010500	-2.01005100
H	4.70435100	-1.04845400	-4.94496200
H	3.43845300	-2.19813100	-4.48417100
H	4.93669300	-2.10786600	-3.54505200
C	7.57499300	-0.51033000	0.60868100

H	7.22366500	-2.27174800	-0.57672900
H	7.61663300	1.27517700	1.84732900
C	4.56939000	-5.15951100	-0.57128100
H	3.00660900	-5.91680700	-1.87757500
H	6.00138400	-4.12436600	0.65722800
H	8.63775200	-0.48641000	0.38333500
H	4.97441200	-6.14067300	-0.33874500

TS mono_L

Zero-point correction= 0.717722 (Hartree/Particle)
Thermal correction to Energy= 0.760675
Thermal correction to Enthalpy= 0.761619
Thermal correction to Gibbs Free Energy= 0.639956
Sum of electronic and zero-point Energies= -2578.935334
Sum of electronic and thermal Energies= -2578.892381
Sum of electronic and thermal Enthalpies= -2578.891437
Sum of electronic and thermal Free Energies= -2579.013101
Electronic energy -2577.71626782

C	-2.64266700	1.69290600	2.52928000
C	-2.61886200	1.07576700	1.26151500
C	-3.60930200	0.12156800	0.94261400
C	-4.59149200	-0.15885200	1.91325800
C	-4.59742800	0.47620500	3.15624100
C	-3.62231600	1.41414500	3.48111400
H	-5.35120400	-0.90009000	1.68734600
H	-5.36632000	0.22614300	3.88210500
H	-3.59898500	1.91623900	4.44251100
C	-3.76266900	-0.58835100	-0.37085600
C	-2.79711600	-1.42192800	-0.98048600
C	-5.02403200	-0.47826500	-0.98826600
C	-3.13921400	-2.11308500	-2.16091500
C	-5.33153100	-1.16983100	-2.16086200
H	-5.76949400	0.16881600	-0.53730900
C	-4.39371700	-2.00447400	-2.75985600
H	-6.31301600	-1.05168300	-2.61180000
H	-4.60614300	-2.55679400	-3.66911100
C	-0.58199300	2.53194300	1.88434400
H	-0.19866000	3.54189000	1.72659700
H	0.20946600	1.90996800	2.31711100
C	-0.89606900	-2.70348000	-2.18457500
H	-0.33950900	-2.06940800	-2.88368500
H	-0.38937900	-3.66749900	-2.09741000
O	-1.67607400	2.60813300	2.81495700
O	-2.20824000	-2.93499400	-2.72081600
C	-1.05398000	-3.17384400	0.76955100
C	0.38041300	-3.74026200	0.80540300
H	1.12028200	-2.95930800	1.01137200
H	0.65386300	-4.23768700	-0.13327800
H	0.45292700	-4.49198900	1.60207800
C	-2.07071000	-4.28531500	0.46212700
H	-2.01105400	-5.06183800	1.23632300
H	-1.88242300	-4.76674600	-0.50383100
H	-3.09661700	-3.90204400	0.45246700
C	-1.36853200	-2.52662000	2.13189100
H	-1.28962400	-3.28622800	2.92084500
H	-2.38187500	-2.11441700	2.16716000
H	-0.66002800	-1.72491900	2.36618200
C	-1.80582700	3.12122900	-0.79452400
C	-0.55917900	3.90235300	-1.25797800
H	-0.86273000	4.67734300	-1.97386400
H	0.16745100	3.24893500	-1.75298700
H	-0.04739100	4.40469100	-0.42948600
C	-2.49022700	2.48973900	-2.02250100
H	-2.79754600	3.28326900	-2.71651100
H	-3.38517400	1.92137300	-1.74901800
H	-1.81038500	1.81878600	-2.55949000
C	-2.78611500	4.05370400	-0.06485300
H	-3.09325000	4.86488900	-0.73849200
H	-2.33936700	4.51269300	0.82346200
H	-3.68977500	3.52159400	0.24976700
P	-1.04845100	-1.79535000	-0.54469400
P	-1.17614200	1.70315300	0.31024000
Cu	0.23376400	0.06843800	-0.41097700
H	1.43680700	0.30329600	-1.49354500
O	1.93675400	-0.01711200	0.92649800
C	2.56787500	0.25553800	-0.15534400
C	3.06508300	1.66150000	-0.37976900
C	3.68782000	2.00057800	-1.59784100
C	2.93957200	2.62111200	0.63186100
C	4.14483800	3.30748200	-1.78598900
C	3.40241700	3.92190900	0.43318300
H	2.48972100	2.32025800	1.57239600
C	3.99863900	4.26856500	-0.78229600
H	4.62764300	3.57244900	-2.72446400
H	3.30984400	4.66067800	1.22573000
H	4.36435400	5.27958400	-0.94321100
C	3.44082000	-0.87663000	-0.79907800
H	2.73481800	-1.59959800	-1.21325200
C	3.85148300	0.91481200	-2.62791000
H	2.88468600	0.71123200	-3.11123100
H	4.55106500	1.23030400	-3.41140300
C	4.36496600	-0.36528800	-1.94235800
H	4.50570700	-1.16593600	-2.67849400
H	5.35694400	-0.14925900	-1.52811400
C	4.25540600	-1.60435000	0.26418900
C	5.11838300	-0.91588200	1.13119900
C	4.19405400	-2.99938900	0.36540300
C	5.88551700	-1.60170500	2.07173800
H	5.18338300	0.16774300	1.07551400
C	4.96596800	-3.69260100	1.30152600
H	3.53548500	-3.55179800	-0.30162200
C	5.81436400	-2.99470800	2.16047100
H	6.54128800	-1.04703200	2.73834200
H	4.90165500	-4.77653200	1.35799300
H	6.41493500	-3.52849700	2.89245100

Ligand=PPh3

Dimeric Cu hydride_PPh3

Zero-point correction= 1.117172 (Hartree/Particle)
Thermal correction to Energy= 1.190426
Thermal correction to Enthalpy= 1.191371
Thermal correction to Gibbs Free Energy= 0.997535
Sum of electronic and zero-point Energies= -4539.992390
Sum of electronic and thermal Energies= -4539.919135
Sum of electronic and thermal Enthalpies= -4539.918191
Sum of electronic and thermal Free Energies= -4540.112027
Electronic energy -4537.35295386

Cu	-1.23355900	-0.02736500	-0.10062800
H	-0.03846100	-0.02948900	1.16908700
H	-0.02075400	0.02252000	-1.35210700
Cu	1.19212400	0.02209200	-0.08238900
P	-2.60391400	1.92679000	0.17826200
P	-2.65794400	-1.92813000	-0.20687900
P	2.62846300	-1.90244700	0.14192400
P	2.59413700	1.94265700	-0.22218300
C	-1.84681800	-3.58023700	-0.41817300
C	-2.14853300	-4.70527400	0.36260700
C	-0.91999500	-3.70975900	-1.46516800
C	-1.54411200	-5.93627400	0.09423500
H	-2.85839100	-4.62672900	1.17996700
C	-0.32778000	-4.94223100	-1.73943000
H	-0.65629000	-2.83948500	-2.05968700
C	-0.63865900	-6.05915300	-0.95965700
H	-1.78588500	-6.79878800	0.71043100

H	0.38585700	-5.02378300	-2.55334800
H	-0.17148300	-7.01802800	-1.16917600
C	-3.91280600	-2.04807700	-1.57542300
C	-4.55834400	-3.25021500	-1.91488800
C	-4.19623600	-0.90422300	-2.33422400
C	-5.48583100	-3.29220400	-2.95648600
H	-4.32208300	-4.16257400	-1.37553200
C	-5.12198800	-0.94448200	-3.38012900
H	-3.67430100	0.02104000	-2.11954100
C	-5.77432400	-2.13793500	-3.68949500
H	-5.97578400	-4.23093000	-3.20268800
H	-5.31965200	-0.04340000	-3.95481100
H	-6.49223600	-2.17450300	-4.50485300
C	-3.64036300	-2.16289400	1.35002000
C	-3.01875300	-1.77520700	2.55002100
C	-4.93185200	-2.70726300	1.40859000
C	-3.66063900	-1.95586000	3.77614500
H	-2.03492000	-1.31244800	2.51417100
C	-5.57750200	-2.87689400	2.63563900
H	-5.44364900	-2.99571400	0.49623900
C	-4.94207700	-2.50898000	3.82246400
H	-3.16380500	-1.65020900	4.69343800
H	-6.58008800	-3.29674800	2.66123300
H	-5.44631000	-2.64207500	4.77619800
C	-4.17901200	1.78860800	1.16286500
C	-4.28498900	2.26929000	2.47706100
C	-5.28701500	1.12587700	0.60575600
C	-5.46235200	2.09624200	3.20883000
H	-3.44950700	2.78834400	2.93446500
C	-6.46419000	0.96113600	1.33444100
H	-5.23886400	0.74075800	-0.40791600
C	-6.55713600	1.44539000	2.64100700
H	-5.52159400	2.48044400	4.22417100
H	-7.30823400	0.44917900	0.87947500
H	-7.47415200	1.31553700	3.20980900
C	-1.76596300	3.32750400	1.05745400
C	-2.01374400	4.68050200	0.78222800
C	-0.88789400	3.00061700	2.10367300
C	-1.40958900	5.68187500	1.54384200
H	-2.67328200	4.95994000	-0.03222700
C	-0.29631600	4.00440500	2.87406900
H	-0.65991500	1.95595000	2.29610000
C	-0.55671700	5.34731000	2.59714100
H	-1.60753100	6.72535500	1.31204700
H	0.38339500	3.73461800	3.67750500
H	-0.09034300	6.12863300	3.19188800
C	-3.22861000	2.68911900	-1.39691700
C	-4.32928200	3.56114600	-1.45732700
C	-2.57737500	2.34668100	-2.59265400
C	-4.75429200	4.08876800	-2.67783800
H	-4.86651600	3.81792600	-0.54910700
C	-3.00463800	2.87254500	-3.81521100
H	-1.74441900	1.64757600	-2.55843600
C	-4.09181300	3.74756300	-3.85986200
H	-5.60738300	4.76198600	-2.70584900
H	-2.49255700	2.58945900	-4.73155000
H	-4.42788900	4.15431200	-4.81025500
C	2.77178600	-2.90039900	-1.41681300
C	2.44012400	-2.26248500	-2.62222000
C	3.22029500	-4.23031400	-1.46034600
C	2.57063100	-2.93137800	-3.84239000
H	2.06040600	-1.24431700	-2.59801900
C	3.35226200	-4.89804700	-2.67873900
H	3.46013100	-4.75367800	-0.54058900
C	3.03097700	-4.24881200	-3.87432900
H	2.30834700	-2.42122500	-4.76563900
H	3.70245100	-5.92724600	-2.69366100
H	3.13390900	-4.76962300	-4.82298700
C	2.15053800	-3.12959200	1.45256800
C	3.04929000	-4.07475400	1.97907200
C	0.84760300	-3.08227600	1.96554300
C	2.64353000	-4.96359000	2.97455400
H	4.07701900	-4.10374500	1.62932200
C	0.44304100	-3.97053600	2.96525600
H	0.16380800	-2.32620900	1.59443800
C	1.33749400	-4.91453600	3.46922700
H	3.35116600	-5.68739400	3.37145600
H	-0.57181400	-3.91533000	3.35021800
H	1.02462400	-5.60302300	4.25034400
C	4.41126400	-1.66030600	0.61385800
C	4.67822500	-1.03583900	1.84562400
C	5.49591800	-2.05658800	-0.18069500
C	5.98793500	-0.81883500	2.27111800
H	3.85580200	-0.71673400	2.47881900
C	6.80995200	-1.84248900	0.24751000
H	5.32271300	-2.53713400	-1.13745300
C	7.06174900	-1.22373500	1.47192400
H	6.16776200	-0.32990100	3.22491900
H	7.63628100	-2.16372200	-0.38191100
H	8.08371400	-1.05827900	1.80313100
C	4.03886700	1.84038400	-1.39408400
C	3.86623300	2.17097400	-2.74972900
C	5.28819400	1.34515200	-0.98705800
C	4.91027000	2.02414100	-3.66304100
H	2.91446700	2.55959000	-3.09679200
C	6.33496200	1.20749400	-1.90155900
H	5.45501500	1.06476300	0.04670500
C	6.15248500	1.54564000	-3.24230000
H	4.75199100	2.29358300	-4.70436200
H	7.29263200	0.82680000	-1.55715200
H	6.96846500	1.43762400	-3.95222200
C	1.80738400	3.51445400	-0.82119600
C	2.21830800	4.79338300	-0.41608300
C	0.80357300	3.40721000	-1.79625900
C	1.65653400	5.93474000	-0.98897600
H	2.97586000	4.90560000	0.35187500
C	0.25313900	4.55089500	-2.38026100
H	0.45235600	2.42064100	-2.08558100
C	0.68101300	5.81726500	-1.98082500
H	1.98645100	6.91746900	-0.66106400
H	-0.52298000	4.44809400	-3.13300600
H	0.24983100	6.70750700	-2.43178000
C	3.36968600	2.42768100	1.39010100
C	2.75759700	1.94685700	2.55869100
C	4.51191000	3.23669100	1.51494100
C	3.26653900	2.27205700	3.81841300
H	1.88481300	1.30344000	2.46912400
C	5.02285700	3.55878100	2.77377000
H	5.01609600	3.60484100	0.62648200
C	4.40092400	3.07856800	3.92883500
H	2.78160900	1.88606300	4.71179100
H	5.90941300	4.18317600	2.85121500
H	4.80191300	3.32791500	4.90800100

Monomeric Cu hydride_PPh3

Zero-point correction= 0.556768 (Hartree/Particle)
Thermal correction to Energy= 0.593349
Thermal correction to Enthalpy= 0.594293
Thermal correction to Gibbs Free Energy= 0.479617
Sum of electronic and zero-point Energies= -2269.987992
Sum of electronic and thermal Energies= -2269.951411
Sum of electronic and thermal Enthalpies= -2269.950467
Sum of electronic and thermal Free Energies= -2270.065143
Electronic energy -2268.64338478

Cu	0.03828800	0.01737500	-1.48540900
H	-0.06610300	0.04218000	-3.05085000
P	2.03789700	-0.00764600	-0.32165000
P	-1.98600600	0.01008000	-0.32738900

C	3.51090100	0.32394100	-1.38678400
C	4.75832700	0.69757700	-0.85905500
C	3.36589900	0.19287700	-2.77621100
C	5.84268200	0.92468600	-1.70637300
H	4.88112700	0.81819200	0.21371900
C	4.45595200	0.41775200	-3.62097000
H	2.39143300	-0.06345300	-3.18554700
C	5.69340200	0.78228400	-3.08913500
H	6.80281000	1.21610100	-1.28824200
H	4.33187400	0.31621200	-4.69593600
H	6.53888200	0.96275900	-3.74811700
C	2.39581000	-1.64125300	0.46267100
C	1.45747100	-2.15616900	1.37628300
C	3.51518200	-2.41960200	0.13400800
C	1.64813600	-3.40813800	1.95958300
H	0.57799100	-1.57335000	1.63674700
C	3.69636600	-3.67941900	0.71083200
H	4.24698300	-2.04448900	-0.57368900
C	2.76760400	-4.17549000	1.62579300
H	0.91654500	-3.78644400	2.66865300
H	4.56788400	-4.27107300	0.44279000
H	2.91126900	-5.15525300	2.07363400
C	2.24763700	1.21563700	1.04956500
C	2.79392900	0.90687500	2.30346400
C	1.80359700	2.52834800	0.80937600
C	2.89434300	1.88814300	3.29372300
H	3.13458900	-0.10239900	2.51284200
C	1.91146600	3.50811300	1.79586600
H	1.36957600	2.78172900	-0.15543900
C	2.45564300	3.18908900	3.04345100
H	3.31933300	1.63277600	4.26114400
H	1.56566300	4.51818000	1.59257900
H	2.53495400	3.95046600	3.81486300
C	-2.87900200	1.60191500	-0.61899400
C	-2.70147600	2.22094300	-1.86907700
C	-3.71385500	2.21027100	0.33097500
C	-3.35550500	3.41859600	-2.16073600
H	-2.04650900	1.75951300	-2.60428300
C	-4.36209000	3.41161600	0.03555400
H	-3.85313100	1.75173700	1.30525000
C	-4.18606100	4.01686500	-1.21019600
H	-3.21038900	3.88669100	-3.13084900
H	-5.00545700	3.87286800	0.78058600
H	-4.69116100	4.95201000	-1.43790000
C	-2.10889100	-0.22466900	1.50648200
C	-2.82409300	-1.27254800	2.10630800
C	-1.36695600	0.64059100	2.33299000
C	-2.80354500	-1.44615700	3.49333500
H	-3.40125800	-1.95633700	1.49259100
C	-1.35744400	0.47264500	3.71691400
H	-0.78882800	1.44932400	1.89368900
C	-2.07481100	-0.57461100	4.30261100
H	-3.36385800	-2.26440000	3.93868000
H	-0.77869900	1.15406200	4.33469200
H	-2.06123500	-0.71080900	5.38075400
C	-3.12956200	-1.26786600	-1.00946900
C	-2.56746300	-2.38862600	-1.63843400
C	-4.52714500	-1.16173300	-0.92775900
C	-3.38528700	-3.39414900	-2.15786000
H	-1.48803900	-2.45871200	-1.74352500
C	-5.34340800	-2.16453600	-1.45204000
H	-4.97845500	-0.29014600	-0.46209700
C	-4.77352700	-3.28397900	-2.06441000
H	-2.93664700	-4.25517400	-2.64590200
H	-6.42422200	-2.06967800	-1.38640700
H	-5.41097800	-4.06236000	-2.47567700

TS_di_PPh3

Zero-point correction= 1.097193 (Hartree/Particle)
Thermal correction to Energy= 1.168250
Thermal correction to Enthalpy= 1.169194
Thermal correction to Gibbs Free Energy= 0.975724
Sum of electronic and zero-point Energies= -4197.080757
Sum of electronic and thermal Energies= -4197.009700
Sum of electronic and thermal Enthalpies= -4197.008755
Sum of electronic and thermal Free Energies= -4197.202226
Electronic energy -4194.43970599

O	1.80746600	1.17744200	0.72811500
C	1.19973400	1.82361100	1.61322000
C	1.29177500	1.41905700	3.04679000
C	0.92714200	2.30890100	4.08327100
C	1.84736300	0.16903200	3.37346600
C	1.11018100	1.90654900	5.41201900
C	2.02960500	-0.21121100	4.69779000
H	2.13401600	-0.50031900	2.56962400
C	1.65616800	0.66248600	5.72539500
H	0.82518100	2.58734800	6.21153300
H	2.46231300	-1.18111500	4.92788700
H	1.79380600	0.37692100	6.76539800
C	0.72744700	3.24576400	1.29240500
H	0.16273200	3.16801600	0.36060600
C	0.39290400	3.69283500	3.77687900
H	-0.34360100	3.97885500	4.53827200
H	1.21514000	4.41941400	3.86271300
C	-0.22686400	3.77982900	2.38052900
H	-1.13489900	3.16867800	2.34382000
H	-0.50669600	4.81427500	2.15076800
C	1.90465900	4.18736800	1.03029300
C	3.12239600	4.10762400	1.72286800
C	1.75763800	5.19894800	0.06853100
C	4.15622700	5.01194300	1.46486300
H	3.27402500	3.32786200	2.46395100
C	2.78654300	6.10524300	-0.18944000
H	0.82437300	5.27175800	-0.48583900
C	3.99292200	6.01551900	0.50857200
H	5.08965100	4.93028600	2.01663400
H	2.64778000	6.87805100	-0.94153500
H	4.79698600	6.71882100	0.30782100
P	3.40807300	-1.65864900	-0.59316200
C	4.08477500	-2.59586200	0.84665300
C	3.17733200	-3.33867300	1.62335300
C	5.43745100	-2.57715100	1.21703600
C	3.61764600	-4.05697700	2.73509100
H	2.12290800	-3.34685000	1.35788600
C	5.87391600	-3.29049000	2.33642300
H	6.15112600	-2.00071400	0.63670900
C	4.96768200	-4.03256900	3.09529900
H	2.90420400	-4.62767300	3.32357700
H	6.92441400	-3.26357900	2.61413000
H	5.30988500	-4.58569600	3.96600300
C	4.77929800	-0.53082200	-1.10251000
C	4.75643300	0.79047300	-0.62435400
C	5.82581300	-0.93403000	-1.94769600
C	5.77102300	1.68422300	-0.97493100
H	3.93654900	1.12260200	0.00578900
C	6.83606900	-0.03617100	-2.29760700
H	5.84805500	-1.94640300	-2.34057200
C	6.81168200	1.27290900	-1.81042700
H	5.73525800	2.70394000	-0.60061100
H	7.63967800	-0.35937900	-2.95442000
H	7.59713200	1.97118700	-2.08798100
C	3.33824400	-2.90444500	-1.95223600
C	4.04535000	-4.11551000	-1.92488400
C	2.52872700	-2.61162200	-3.06267100
C	3.94794400	-5.01451200	-2.98984900
H	4.66744200	-4.36068300	-1.06918800
C	2.44193300	-3.50655000	-4.12907800
H	1.96260900	-1.68397100	-3.08858800

C	3.14926300	-4.71098000	-4.09367300
H	4.49693400	-5.95199600	-2.95461500
H	1.81323900	-3.26632900	-4.98230100
H	3.07361100	-5.41219000	-4.92064300
Cu	1.37630200	-0.76424900	-0.07908300
H	-0.14062300	-1.23167600	-0.07280200
Cu	-1.29738900	0.01303500	0.22383100
H	-0.78593200	1.05719600	1.37409500
P	-3.07655500	-1.32261100	1.03023500
P	-1.77334400	1.14990000	-1.80237000
C	-3.17734200	0.53529200	-2.84600300
C	-4.17146800	1.36037100	-3.39358500
C	-3.25928100	-0.85170900	-3.06144700
C	-5.22083700	0.81064100	-4.13551200
H	-4.13134000	2.43383300	-3.23829600
C	-4.30161700	-1.39868000	-3.80988300
H	-2.51067700	-1.50766000	-2.62632900
C	-5.28851800	-0.56760500	-4.34684000
H	-5.98517100	1.46377400	-4.54944300
H	-4.35023200	-2.47451600	-3.95450900
H	-6.10774300	-0.99238500	-4.92119000
C	-0.39140800	1.25310800	-3.03453100
C	-0.57019800	1.06835800	-4.41406400
C	0.90208700	1.52078300	-2.55165100
C	0.51705600	1.14608600	-5.28938500
H	-1.55850100	0.86100900	-4.81165300
C	1.98453100	1.60924100	-3.42942300
H	1.07174800	1.65411800	-1.48650100
C	1.79581400	1.41771000	-4.80105800
H	0.35902100	0.99805900	-6.35493000
H	2.97569300	1.81720100	-3.03549300
H	2.64049100	1.47843400	-5.48254300
C	-2.20914100	2.93064800	-1.53702500
C	-1.86304900	3.95787600	-2.42938300
C	-2.92047900	3.25808000	-0.37076500
C	-2.22275800	5.28126000	-2.16176000
H	-1.30754500	3.72600700	-3.33316100
C	-3.29015600	4.57896900	-0.11129600
H	-3.16538300	2.47481900	0.34125400
C	-2.93928700	5.59418000	-1.00420900
H	-1.94464600	6.06647300	-2.86041800
H	-3.84177900	4.81478300	0.79501400
H	-3.21783200	6.62427600	-0.79708800
C	-3.55779200	-2.84437700	0.08390700
C	-4.87321600	-3.31451300	-0.04809400
C	-2.51974600	-3.56018700	-0.53846900
C	-5.14447900	-4.47088700	-0.78451400
H	-5.69164300	-2.77448900	0.41803500
C	-2.79140200	-4.72017800	-1.26473400
H	-1.49871300	-3.19439400	-0.45914700
C	-4.10579600	-5.17773400	-1.39292000
H	-6.17010000	-4.81934500	-0.87892000
H	-1.97587600	-5.26214700	-1.73722700
H	-4.31853200	-6.07786200	-1.96413500
C	-4.69188100	-0.46088100	1.30946800
C	-5.41156500	0.00752700	0.19436500
C	-5.17630000	-0.16081900	2.59190700
C	-6.58685400	0.73964100	0.35998300
H	-5.05557500	-0.20597100	-0.80925300
C	-6.34776100	0.58344400	2.75525900
H	-4.63918700	-0.50878500	3.46813200
C	-7.05827600	1.03383000	1.64235000
H	-7.12952700	1.08575700	-0.51595200
H	-6.70558300	0.80534900	3.75763600
H	-7.97102100	1.60980500	1.77125900
C	-2.68652000	-2.02216900	2.70016300
C	-1.72153800	-1.36990400	3.48287600
C	-3.32257900	-3.16430000	3.21406800
C	-1.41147100	-1.84344500	4.76081400
H	-1.21333800	-0.49776300	3.07814000
C	-3.00540600	-3.63910600	4.48694900
H	-4.06301600	-3.68892800	2.61746200
C	-2.05133000	-2.97695200	5.26434500
H	-0.66114100	-1.32672800	5.35234000
H	-3.50382400	-4.52575800	4.87116400
H	-1.80586600	-3.34757500	6.25671900

TS_mono_PPh3

Zero-point correction= 0.814054 (Hartree/Particle)
Thermal correction to Energy= 0.864584
Thermal correction to Enthalpy= 0.865528
Thermal correction to Gibbs Free Energy= 0.720452
Sum of electronic and zero-point Energies= -2963.070343
Sum of electronic and thermal Energies= -2963.019813
Sum of electronic and thermal Enthalpies= -2963.018869
Sum of electronic and thermal Free Energies= -2963.163944
Electronic energy -2961.67376884

Cu	0.03622500	0.12835000	0.13011700
H	-0.84381400	0.93008400	1.28238300
O	-1.53179800	1.17500900	-1.10124800
C	-1.94961200	1.58867800	0.02965800
C	-1.73233300	3.02053700	0.43398900
C	-2.09138600	3.44722100	1.72964900
C	-1.24476600	3.94575000	-0.49588900
C	-1.93923100	4.79322800	2.06937900
C	-1.10374800	5.28805000	-0.14604900
H	-0.98857700	3.59144200	-1.48847000
C	-1.44705700	5.71255700	1.13966500
H	-2.21684700	5.12522500	3.06790200
H	-0.72532600	6.00143900	-0.87305100
H	-1.34106800	6.75865800	1.41647900
C	-3.23558200	0.92959900	0.62703000
H	-2.98268000	-0.11722500	0.80706100
C	-2.64060600	2.41854500	2.67984300
H	-1.82513600	1.76543800	3.02162100
H	-3.07175500	2.90136500	3.56549300
C	-3.71379200	1.57502500	1.96680700
H	-4.08753000	0.78892100	2.63444900
H	-4.56661700	2.23041700	1.75614900
C	-4.37484700	0.93992300	-0.39046200
C	-4.75302400	2.10873300	-1.07055700
C	-5.11965900	-0.22433400	-0.61552100
C	-5.83367200	2.10670300	-1.95199100
H	-4.19323500	3.02671000	-0.91456600
C	-6.20896100	-0.22854100	-1.49061000
H	-4.84174700	-1.14168200	-0.10233500
C	-6.56954700	0.93794300	-2.16491400
H	-6.10278000	3.02211300	-2.47344300
H	-6.77076100	-1.14649700	-1.64572600
H	-7.41437900	0.93872500	-2.84906800
P	2.15273300	0.95244000	-0.27437400
P	-0.34609900	-2.16173800	0.19575900
C	2.41339500	2.78388300	-0.37457700
C	2.90948900	3.53817000	0.69728800
C	2.07621100	3.44519900	-1.56815000
C	3.07654500	4.91998500	0.57463300
H	3.17584500	3.05062100	1.62949700
C	2.25799600	4.82146400	-1.69280100
H	1.68142400	2.88037000	-2.40818100
C	2.75925800	5.56426200	-0.62074500
H	3.46468900	5.48893200	1.41544800
H	2.00397000	5.31431000	-2.62778500
H	2.89938300	6.63763800	-0.71750100
C	3.07707000	0.38583800	-1.77472400
C	2.58633400	-0.71372500	-2.49191800
C	4.24667600	1.02085300	-2.23052900
C	3.25389600	-1.18238800	-3.62579900
H	1.67720800	-1.20457200	-2.16226200

C	4.91262400	0.55333400	-3.36335200
H	4.62941700	1.89215300	-1.70750600
C	4.41835000	-0.55129800	-4.06268900
H	2.85820400	-2.03842300	-4.16516700
H	5.81467100	1.05548700	-3.70344900
H	4.93623000	-0.91157500	-4.94776800
C	3.22011300	0.44703000	1.15402400
C	4.56987900	0.08379300	1.03356200
C	2.62186500	0.41285400	2.42647000
C	5.30463400	-0.29812800	2.15814000
H	5.04970500	0.08589900	0.06042200
C	3.36189000	0.04120300	3.55074700
H	1.56950000	0.66759200	2.52775600
C	4.70509500	-0.31729400	3.41904200
H	6.34845400	-0.58049200	2.04650600
H	2.88404900	0.02300700	4.52671400
H	5.27955600	-0.61465100	4.29244900
C	-0.73754000	-2.81419800	1.88527300
C	-1.34750300	-1.95038500	2.80766400
C	-0.46759100	-4.13744500	2.27318900
C	-1.70210000	-2.40504600	4.07949800
H	-1.51757000	-0.91374000	2.52939500
C	-0.81857700	-4.58843000	3.54635000
H	0.03185800	-4.81455600	1.58671400
C	-1.44115400	-3.72493500	4.45072700
H	-2.17382000	-1.72321800	4.78242800
H	-0.60183300	-5.61441900	3.83262300
H	-1.71229400	-4.07769300	5.44239600
C	1.08951700	-3.22359300	-0.29651800
C	1.07694500	-4.05090400	-1.42916500
C	2.27238700	-3.13352600	0.45944300
C	2.21740100	-4.77285400	-1.79394400
H	0.17651600	-4.13864400	-2.02816600
C	3.40552700	-3.85970700	0.09721300
H	2.30911200	-2.49206900	1.33561700
C	3.38271800	-4.68158500	-1.03305600
H	2.18800400	-5.41171300	-2.67305900
H	4.30872200	-3.77588000	0.69557100
H	4.26771800	-5.24439700	-1.31770500
C	-1.72266900	-2.78370600	-0.86816900
C	-2.20652700	-1.93721700	-1.87772600
C	-2.27104500	-4.07102500	-0.73024400
C	-3.20298400	-2.38542400	-2.75078400
H	-1.84326900	-0.91493700	-1.94705800
C	-3.26734100	-4.51049800	-1.60065400
H	-1.92324300	-4.73070800	0.05880900
C	-3.72890100	-3.67000200	-2.61890000
H	-3.57947400	-1.71556900	-3.51838100
H	-3.68440200	-5.50754900	-1.48387000
H	-4.50608000	-4.01412500	-3.29662600

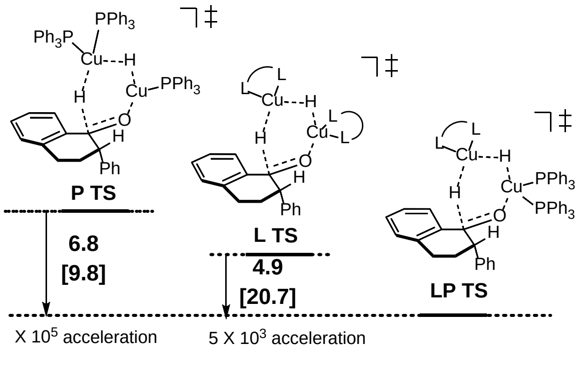

P TS corresponds to **TS_di_PPh3** in the previous section
L TS corresponds to **TS_di_L** in the previous section

LP TS

Zero-point correction= 1.276138 (Hartree/Particle)
Thermal correction to Energy= 1.357267
Thermal correction to Enthalpy= 1.358211
Thermal correction to Gibbs Free Energy= 1.150766
Sum of electronic and zero-point Energies= -4848.944841
Sum of electronic and thermal Energies= -4848.863713
Sum of electronic and thermal Enthalpies= -4848.862768
Sum of electronic and thermal Free Energies= -4849.070213
Electronic energy -4846.42287131

C	-4.32244	0.07380	3.48586
C	-4.48120	0.07864	2.08590
C	-5.54764	-0.64664	1.51630
C	-6.41459	-1.34670	2.37787
C	-6.22951	-1.33895	3.76130
C	-5.17967	-0.62689	4.33458
H	-7.23155	-1.91747	1.94756
H	-6.90947	-1.89964	4.39716
H	-5.01726	-0.60022	5.40712
C	-5.86855	-0.68322	0.05501
C	-4.97340	-1.09738	-0.95401
C	-7.18271	-0.34032	-0.31755
C	-5.43025	-1.16086	-2.28472
C	-7.60305	-0.39976	-1.64704
H	-7.87328	-0.00768	0.45101
C	-6.73163	-0.81543	-2.64950
H	-8.62049	-0.11577	-1.90212
H	-7.03476	-0.87870	-3.68941
C	-2.39048	1.26997	3.01157
H	-2.07777	2.28221	3.27492
H	-1.51150	0.61625	3.01669
C	-3.22250	-1.71626	-2.75090
H	-2.64520	-0.86811	-3.13318
H	-2.79862	-2.64547	-3.14086
O	-3.31353	0.81321	4.01943
O	-4.57082	-1.60887	-3.24025
C	-3.29640	-3.48563	-0.35454
C	-1.94395	-4.11585	-0.73154
H	-1.10912	-3.58487	-0.26471
H	-1.77423	-4.13479	-1.81380
H	-1.91338	-5.15497	-0.37861
C	-4.44507	-4.24715	-1.03575
H	-4.42054	-5.30181	-0.72822
H	-4.37062	-4.22127	-2.12867
H	-5.42266	-3.84150	-0.75658
C	-3.45778	-3.54689	1.17633
H	-3.40659	-4.59362	1.50564
H	-4.41784	-3.13999	1.50593
H	-2.65980	-2.99655	1.68690
C	-4.04926	2.83937	1.06748
C	-2.93249	3.89434	0.97286
H	-3.36725	4.86359	0.69549
H	-2.18856	3.63591	0.21282
H	-2.41776	4.02996	1.93016
C	-4.80089	2.79070	-0.27651
H	-5.24079	3.77573	-0.48383
H	-5.61352	2.05808	-0.26846
H	-4.12405	2.54384	-1.10157
C	-5.01780	3.19772	2.20619
H	-5.43834	4.19772	2.03136
H	-4.51882	3.21352	3.18163
H	-5.85194	2.49154	2.26781
P	-3.21123	-1.63420	-0.86711
P	-3.19072	1.14243	1.31284
Cu	-1.69301	0.11705	-0.21263
H	-1.14912	0.93579	-1.53900

C	0.48664	1.92816	-1.98721
C	-0.18565	3.26257	-2.03443
C	-1.19400	3.50936	-2.99190
C	0.24579	4.30030	-1.19404
C	-1.76567	4.78257	-3.06368
C	-0.32620	5.56835	-1.28461
H	1.04602	4.09897	-0.49023
C	-1.33923	5.80918	-2.21754
H	-2.54602	4.97344	-3.79755
H	0.02359	6.36953	-0.63817
H	-1.78727	6.79668	-2.29558
C	0.66348	1.21514	-3.35441
H	0.53553	0.15147	-3.15608
C	-1.60343	2.37462	-3.88763
H	-2.19123	1.65671	-3.29844
H	-2.23116	2.73679	-4.71092
C	-0.35395	1.66879	-4.44656
H	-0.64844	0.80051	-5.04895
H	0.14603	2.36179	-5.13328
C	2.10005	1.41621	-3.84279
C	2.65017	2.70042	-3.99188
C	2.89562	0.31523	-4.18535
C	3.95428	2.87465	-4.45600
H	2.05451	3.57321	-3.73757
C	4.20131	0.48592	-4.65586
H	2.48913	-0.68689	-4.08942
C	4.73821	1.76636	-4.79025
H	4.35658	3.87932	-4.56306
H	4.79507	-0.38655	-4.91760
H	5.75230	1.90207	-5.15765
O	1.26793	1.64322	-1.04606
H	-0.32640	-0.26459	0.71587
P	2.66328	0.98799	2.00138
C	3.10763	-0.14888	3.38723
C	4.22391	-0.99852	3.30729
C	2.23121	-0.29734	4.47711
C	4.46691	-1.95065	4.29926
H	4.90943	-0.91859	2.46996
C	2.47582	-1.25095	5.46637
H	1.35664	0.34151	4.55908
C	3.59683	-2.07961	5.38304
H	5.33954	-2.59348	4.21881
H	1.78993	-1.34185	6.30484
H	3.78906	-2.82007	6.15493
C	1.97584	2.46886	2.87892
C	0.92604	3.16569	2.26559
C	2.52169	2.97202	4.07167
C	0.44639	4.35530	2.82340
H	0.49989	2.77858	1.34493
C	2.02649	4.14572	4.63827
H	3.33371	2.44482	4.56372
C	0.99102	4.84418	4.01099
H	-0.34814	4.90018	2.32212
H	2.45528	4.52061	5.56421
H	0.61406	5.76609	4.44634
C	4.27790	1.67275	1.39786
C	4.32806	2.09473	0.05910
C	5.41089	1.84873	2.20854
C	5.48912	2.67652	-0.45649
H	3.45540	1.97338	-0.57726
C	6.57210	2.42063	1.68748
H	5.39616	1.53129	3.24705
C	6.61413	2.83602	0.35391
H	5.50826	2.99358	-1.49597
H	7.44353	2.54521	2.32559
H	7.51962	3.28355	-0.04835
Cu	1.27601	-0.07258	0.37695
P	2.29683	-2.11046	-0.32680
C	4.14046	-2.26903	-0.22526
C	4.80005	-3.29692	0.46593
C	4.91156	-1.27269	-0.84947
C	6.19596	-3.32696	0.53060
H	4.22642	-4.07521	0.95815
C	6.30449	-1.30764	-0.78848
H	4.42194	-0.46291	-1.38265
C	6.95132	-2.33402	-0.09493
H	6.69087	-4.13058	1.07043
H	6.88061	-0.52215	-1.26937
H	8.03661	-2.35704	-0.04043
C	1.95862	-2.71759	-2.04431
C	0.70246	-2.43555	-2.60594
C	2.89135	-3.43873	-2.80639
C	0.38488	-2.87544	-3.89276
H	-0.01841	-1.85492	-2.03507
C	2.57297	-3.87352	-4.09549
H	3.87281	-3.65633	-2.39712
C	1.31869	-3.59529	-4.64159
H	-0.58731	-2.64073	-4.31832
H	3.30806	-4.42905	-4.67227
H	1.07323	-3.92940	-5.64612
C	1.69370	-3.51814	0.71441
C	1.20902	-3.23176	1.99981
C	1.70427	-4.85307	0.27713
C	0.75885	-4.25713	2.83424
H	1.17404	-2.20272	2.34255
C	1.24990	-5.87657	1.11036
H	2.06126	-5.09499	-0.71965
C	0.77776	-5.58079	2.39164
H	0.39025	-4.01673	3.82770
H	1.26278	-6.90453	0.75697
H	0.42185	-6.37831	3.03880

For equation balancing:

PPh3

Zero-point correction= 0.274251 (Hartree/Particle)
Thermal correction to Energy= 0.290171
Thermal correction to Enthalpy= 0.291115
Thermal correction to Gibbs Free Energy= 0.227762
Sum of electronic and zero-point Energies= -1036.024001
Sum of electronic and thermal Energies= -1036.008082
Sum of electronic and thermal Enthalpies= -1036.007137
Sum of electronic and thermal Free Energies= -1036.070490
Electronic energy -1035.90589839

P	-0.00003200	0.00012000	-1.20615500
C	1.55444500	0.61188200	-0.40195000
C	2.22121400	1.67368500	-1.03648300
C	2.11612800	0.06981500	0.76430000
C	3.40344200	2.19376200	-0.51015700
H	1.81159500	2.09190000	-1.95322800
C	3.30602600	0.58285500	1.28529600
H	1.62527000	-0.75892600	1.26576400
C	3.95036700	1.64720700	0.65271100
H	3.90276700	3.01774900	-1.01340500
H	3.72894900	0.14939400	2.18818300
H	4.87678800	2.04423600	1.05942500
C	-0.24724200	-1.65220100	-0.40254500
C	0.35070800	-2.75845800	-1.02970000
C	-1.00773400	-1.86976600	0.75652500
C	0.21153500	-4.04219300	-0.50273700
H	0.92603500	-2.61135700	-1.94101200
C	-1.15706100	-3.15679200	1.27792700
H	-1.48863700	-1.03171500	1.25197400
C	-0.54567200	-4.24481500	0.65297600
H	0.68493800	-4.88490500	-1.00000300
H	-1.75186600	-3.30793900	2.17533100
H	-0.66374400	-5.24565000	1.06002300

C	-1.30826300	1.03996300	-0.40309000
C	-2.56707600	1.06978600	-1.02708900
C	-1.11450400	1.81429800	0.75090900
C	-3.60885600	1.83378600	-0.50172100
H	-2.72917000	0.49259200	-1.93470100
C	-2.15408700	2.58888600	1.27055600
H	-0.14706100	1.81597200	1.24388500
C	-3.40365400	2.59806400	0.64896100
H	-4.57669600	1.84098400	-0.99648400
H	-1.98591100	3.18515500	2.16394400
H	-4.21108300	3.20212200	1.05450600

L

Zero-point correction= 0.453022 (Hartree/Particle)
Thermal correction to Energy= 0.479321
Thermal correction to Enthalpy= 0.480266
Thermal correction to Gibbs Free Energy= 0.398198
Sum of electronic and zero-point Energies= -1687.900932
Sum of electronic and thermal Energies= -1687.874633
Sum of electronic and thermal Enthalpies= -1687.873688
Sum of electronic and thermal Free Energies= -1687.955756
Electronic energy -1687.87355699

C	2.18507900	-1.97734000	0.26734700
C	1.40180800	-0.80979700	0.23692400
C	0.45796200	-0.59034900	1.25812000
C	0.38842300	-1.50957100	2.31905400
C	1.20563200	-2.64210100	2.34386300
C	2.10654200	-2.89837000	1.31212700
H	-0.33104600	-1.34229400	3.11533400
H	1.12459900	-3.34411100	3.16954500
H	2.73415500	-3.78355300	1.30334800
C	-0.45794400	0.59031900	1.25812800
C	-1.40179700	0.80977800	0.23694100
C	-0.38838800	1.50953700	2.31906400
C	-2.18505500	1.97732900	0.26737300
C	-1.20558800	2.64207300	2.34388500
H	0.33108800	1.34225300	3.11533600
C	-2.10650400	2.89835400	1.31215600
H	-1.12454100	3.34408000	3.16956700
H	-2.73410600	3.78354400	1.30338300
C	2.79887900	-1.26438500	-1.84631700
H	3.75900500	-1.01625600	-2.30445200
H	2.18038000	-1.78489500	-2.58836200
C	-2.79886200	1.26439800	-1.84629700
H	-2.18035200	1.78490300	-2.58833500
H	-3.75899000	1.01628700	-2.30443800
O	3.05698400	-2.17913100	-0.75884900
O	-3.05695800	2.17913800	-0.75882200
C	-3.28155300	-1.35342100	-0.54641100
C	-3.98490800	-1.92720600	-1.79303900
H	-3.27974900	-2.44438400	-2.45443100
H	-4.49213700	-1.15179600	-2.37949200
H	-4.74922100	-2.65367300	-1.48637600
C	-4.30316000	-0.64144100	0.35315500
H	-5.07169900	-1.35603800	0.67986000
H	-4.81298000	0.17872300	-0.16263800
H	-3.83062100	-0.23019800	1.25067900
C	-2.62467100	-2.50974500	0.23094400
H	-3.39295700	-3.22897500	0.54615900
H	-2.11081300	-2.15574100	1.13000700
H	-1.89349400	-3.04882600	-0.38234200
C	3.28152100	1.35343900	-0.54641300
C	3.98482100	1.92730800	-1.79303400
H	4.74910600	2.65379900	-1.48635700
H	3.27962400	2.44447900	-2.45438900
H	4.49207600	1.15194700	-2.37952800
C	2.62461200	2.50970400	0.23100900
H	3.39287900	3.22894600	0.54624400
H	2.11078400	2.15563800	1.13006600
H	1.89340400	3.04878700	-0.38223700
C	4.30317700	0.64146000	0.35309700
H	5.07169800	1.35607300	0.67981200
H	4.81301400	-0.17866300	-0.16274400
H	3.83067600	0.23016200	1.25061600
P	-1.85869700	-0.24154400	-1.21207800
P	1.85868800	0.24153800	-1.21209000

ADDITIONAL INFORMATION

Explored homoligated reduction transition states

Ligand=L

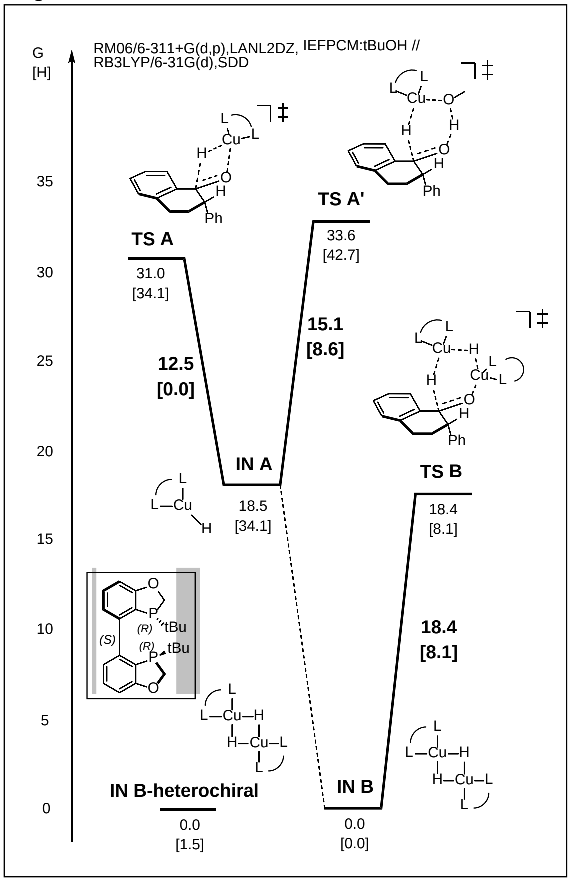

IN B corresponds to **Dimeric Cu hydride_L** in the previous section
IN A corresponds to **monomeric Cu hydride_L** in the previous section
TS A corresponds to **TS_mono_L** in the previous section
TS B corresponds to **TS_di_L** in the previous section

TS A'

Zero-point correction= 0.771572 (Hartree/Particle)

Thermal correction to Energy= 0.818560
Thermal correction to Enthalpy= 0.819504
Thermal correction to Gibbs Free Energy= 0.688228
Sum of electronic and zero-point Energies= -2694.615105
Sum of electronic and thermal Energies= -2694.568117
Sum of electronic and thermal Enthalpies= -2694.567173
Sum of electronic and thermal Free Energies= -2694.698449
Electronic energy -2693.42487006

C	4.04278200	1.77841800	-1.48276300
C	3.41223000	0.95771100	-0.52573100
C	4.08582800	-0.19470100	-0.06193600
C	5.35957300	-0.47410000	-0.59511000
C	5.95551000	0.35306200	-1.54828900
C	5.30598700	1.49680900	-2.00118700
H	5.88104700	-1.36353000	-0.25661900
H	6.93526300	0.09816400	-1.94276500
H	5.74662100	2.16036000	-2.73773600
C	3.61354300	-1.11663600	1.02482300
C	2.41216200	-1.85951100	1.02050400
C	4.50289500	-1.30475600	2.10148400
C	2.15755600	-2.75505400	2.07933000
C	4.22264100	-2.19689200	3.13769800
H	5.42361500	-0.73070600	2.12401400
C	3.04628600	-2.93966200	3.13793900
H	4.92926000	-2.30784800	3.95562900
H	2.80404900	-3.64090900	3.92948700
C	2.01242400	2.90740200	-1.50544400
H	1.74767600	3.92270700	-1.20171400
H	1.40542600	2.63288000	-2.37435600
C	0.07578600	-2.96442100	1.07781400
H	-0.66549000	-2.34792500	1.59872800
H	-0.43310000	-3.81107600	0.61165800
O	3.39404400	2.90265700	-1.89707900
O	1.00519900	-3.47937100	2.04588400
C	1.45319100	-3.06829200	-1.57506300
C	0.13219100	-3.39455900	-2.30277600
H	-0.37248700	-2.48278800	-2.63941500
H	-0.56330200	-3.96082500	-1.67208400
H	0.34730600	-4.01419600	-3.18349100
C	2.14048400	-4.35261600	-1.08558800
H	2.36092900	-5.00213600	-1.94346000
H	1.51156600	-4.92374800	-0.39395300
H	3.08712300	-4.13675100	-0.57886900
C	2.37956600	-2.30358000	-2.53928800
H	2.59889100	-2.93378000	-3.41145100
H	3.33275300	-2.03935300	-2.07075500
H	1.90516800	-1.38422200	-2.89901800
C	1.92430500	2.65757200	1.46923100
C	0.70241800	3.59580900	1.53255300
H	0.70574400	4.13025900	2.49145600
H	-0.24132900	3.04561000	1.45622500
H	0.71634200	4.35356900	0.74066700
C	1.86602500	1.67404800	2.65404900
H	1.88195100	2.23727400	3.59650700
H	2.71868000	0.98782600	2.66153400
H	0.94675400	1.07808400	2.63311100
C	3.22999400	3.46746400	1.51681600
H	3.26595600	4.05306000	2.44502500
H	3.31372100	4.16973900	0.68001700
H	4.10978900	2.81606300	1.49830400
P	0.99575900	-1.88150300	-0.15341200
P	1.74919500	1.64353500	-0.13613900
Cu	0.00381600	0.19202000	-0.42330000
H	-1.48965600	0.34992200	0.23440900
C	-3.15062800	0.44848900	-0.30275300
C	-3.23617300	1.91605400	0.02705800
C	-3.19767600	2.35064500	1.36748300
C	-3.38359100	2.84975700	-1.00611800
C	-3.29384600	3.71796400	1.64097200
C	-3.47305500	4.21174300	-0.72086100
H	-3.43618000	2.48063500	-2.02564000
C	-3.42454700	4.64810600	0.60628300
H	-3.27293800	4.05651300	2.67509400
H	-3.59263200	4.93087800	-1.52763400
H	-3.50299500	5.70763300	0.83699500
C	-3.69149400	-0.55031400	0.77731500
H	-2.89701900	-1.28446600	0.92975800
C	-3.06613000	1.30727700	2.44565100
H	-2.02747800	0.94514000	2.48295000
H	-3.30116800	1.73624500	3.42734700
C	-4.00304200	0.12788700	2.13686300
H	-3.95776200	-0.62418100	2.93411300
H	-5.03352300	0.50370800	2.12433900
C	-4.91028400	-1.30559800	0.26363300
C	-6.04468700	-0.62913500	-0.21095000
C	-4.94359300	-2.70414400	0.30096300
C	-7.17198500	-1.33030100	-0.63532100
H	-6.03907000	0.45693900	-0.26091300
C	-6.07277900	-3.41265500	-0.11802000
H	-4.07351300	-3.24735400	0.66456500
C	-7.19243500	-2.72708200	-0.58819000
H	-8.03680800	-0.78557000	-1.00608600
H	-6.07408700	-4.49924000	-0.07822300
H	-8.07236300	-3.27356800	-0.91769000
O	-0.78994800	0.07029500	-2.51461200
C	-0.70444400	1.03152300	-3.55786200
H	-0.93154400	2.04737400	-3.20457100
H	0.31623700	1.01213700	-3.95294000
H	-1.39425500	0.78657400	-4.37676900
H	-1.74758200	0.04940500	-2.20327400
O	-3.17910600	0.07692300	-1.50729500

Ligand=PPh3

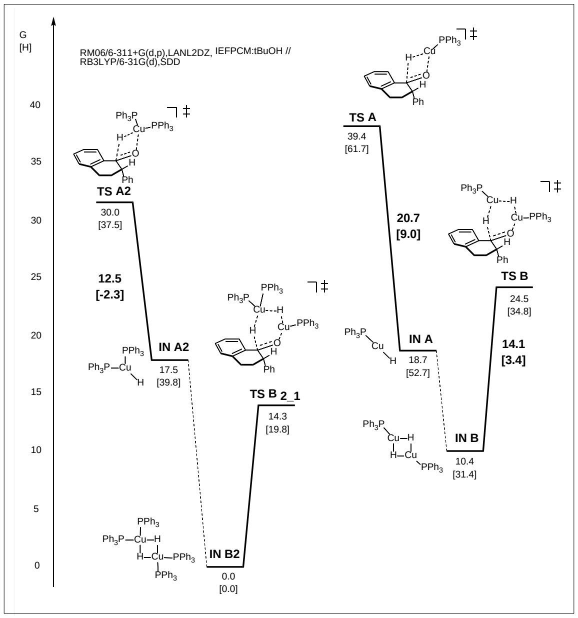

IN B2 corresponds to **Dimeric Cu hydride_PPh3** in the previous section
IN A2 corresponds to **monomeric Cu hydride_ PPh3** in the previous section
TS A2 corresponds to **TS_mono_ PPh3** in the previous section
TS B 2_1 corresponds to **TS_di_ PPh3** in the previous section

IN A

Zero-point correction= 0.281858 (Hartree/Particle)
Thermal correction to Energy= 0.300455
Thermal correction to Enthalpy= 0.301399
Thermal correction to Gibbs Free Energy= 0.231565
Sum of electronic and zero-point Energies= -1233.971836
Sum of electronic and thermal Energies= -1233.953239
Sum of electronic and thermal Enthalpies= -1233.952295
Sum of electronic and thermal Free Energies= -1234.022129
Electronic energy -1232.71520622

C	-1.61482100	0.48870700	-0.22027400
C	-2.37155900	1.46434900	0.45025200
C	-2.11696600	-0.07045500	-1.40507100
C	-3.59815700	1.88402200	-0.06451700
H	-2.00295800	1.88922600	1.38083000
C	-3.34808200	0.34777000	-1.91420000
H	-1.55219700	-0.83772000	-1.92611100
C	-4.08833000	1.32598200	-1.24743200
H	-4.17439600	2.63816000	0.46424300
H	-3.72921900	-0.09431500	-2.83085700
H	-5.04781600	1.64686700	-1.64388600
C	0.38372200	-1.64230200	-0.21842900
C	-0.08143200	-2.78489500	0.45404000
C	1.11708000	-1.79879700	-1.40428000
C	0.16779200	-4.05745700	-0.05989400
H	-0.63231400	-2.67730300	1.38533100
C	1.36963200	-3.07450200	-1.91272200
H	1.49867100	-0.92673800	-1.92677200
C	0.89387100	-4.20411800	-1.24403900
H	-0.19609800	-4.93310600	0.47038800
H	1.94151300	-3.18420400	-2.83026400
H	1.09497300	-5.19587700	-1.63992700
C	1.23027400	1.15359900	-0.22046100
C	2.45263300	1.32211600	0.45149400
C	0.99932600	1.86508700	-1.40755100
C	3.43072000	2.17254600	-0.06408200
H	2.63434300	0.79267500	1.38371800
C	1.97850000	2.71998900	-1.91755500
H	0.05313600	1.75891000	-1.92963700
C	3.19489900	2.87283200	-1.24933300
H	4.37127200	2.29534700	0.46567600
H	1.78799100	3.26879700	-2.83596500
H	3.95365300	3.54159600	-1.64658600
P	-0.00040700	0.00066800	0.51830100
Cu	0.00032300	0.00330000	2.78429100
H	0.00159100	0.00513300	4.30518800

TS A

Zero-point correction= 0.538740 (Hartree/Particle)
Thermal correction to Energy= 0.571474
Thermal correction to Enthalpy= 0.572418
Thermal correction to Gibbs Free Energy= 0.467005
Sum of electronic and zero-point Energies= -1927.053666
Sum of electronic and thermal Energies= -1927.020932
Sum of electronic and thermal Enthalpies= -1927.019988
Sum of electronic and thermal Free Energies= -1927.125401
Electronic energy -1925.72720394

H	-1.24561200	0.91514800	1.40141300
O	-1.61359200	-0.27001900	-0.70358500
C	-2.26716400	0.35987400	0.22485400
C	-2.83806800	1.71777200	-0.10157800
C	-3.54031200	2.43315800	0.88780400
C	-2.67686700	2.25805200	-1.38209100
C	-4.06692400	3.68770700	0.57081800
C	-3.20588100	3.51263900	-1.68477900
H	-2.13790500	1.67583800	-2.12269900
C	-3.89917400	4.22969300	-0.70603500
H	-4.61523200	4.24290700	1.32921500
H	-3.08231900	3.92886200	-2.68145800
H	-4.31650500	5.20645300	-0.93794500
C	-3.12488600	-0.51667800	1.20613900
C	-3.70025700	1.78897600	2.24023500
H	-2.75038500	1.84051800	2.79295500
H	-4.44976600	2.32478600	2.83509600
C	-4.11795500	0.31693100	2.06579100
H	-4.24209200	-0.16506500	3.04281600
H	-5.10403300	0.30333200	1.58749400
Cu	-0.04001600	0.32361100	0.48427900
P	2.07166600	-0.05980400	0.07719400
C	2.32112300	-1.62132200	-0.86761900
C	1.22950900	-2.12714700	-1.59380800
C	3.54228500	-2.31480300	-0.88520100
C	1.37009300	-3.29882500	-2.34061400
H	0.27006100	-1.61595000	-1.56009600
C	3.67456900	-3.48611700	-1.63163700
H	4.38650400	-1.94827300	-0.30833100
C	2.58969800	-3.97744200	-2.36251400
H	0.51935600	-3.68404100	-2.89578100
H	4.62258500	-4.01752200	-1.63717000
H	2.69371200	-4.89261400	-2.93944600
C	3.19289200	-0.20150500	1.53217200
C	2.67230600	-0.73674100	2.72061900
C	4.53861300	0.19391200	1.49329800
C	3.48645500	-0.89162400	3.84250100
H	1.62341300	-1.01883500	2.76865100
C	5.34976200	0.04427600	2.62000600
H	4.95109600	0.62974000	0.58799500
C	4.82669300	-0.50123800	3.79393400
H	3.07099700	-1.30611000	4.75697400
H	6.38939000	0.35832300	2.58019400
H	5.45859600	-0.61332500	4.67083100
C	2.81387900	1.27674700	-0.95093500
C	2.54016900	2.60770300	-0.59076900
C	3.61268700	1.02422000	-2.07510100
C	3.06876100	3.66289700	-1.33270900
H	1.90651800	2.81558200	0.26798300
C	4.13520600	2.08461100	-2.81983700
H	3.82069600	0.00224800	-2.37566200
C	3.86717400	3.40303500	-2.44974400
H	2.84849400	4.68703200	-1.04438500
H	4.74951700	1.87660600	-3.69181000
H	4.27222100	4.22568000	-3.03282600
C	-3.84412300	-1.61914000	0.43870100
C	-3.58618300	-2.96399700	0.72899900
C	-4.79310200	-1.32557700	-0.55283200
C	-4.25582100	-3.98979700	0.05729600
H	-2.85186700	-3.21128700	1.49279300
C	-5.45889000	-2.34534400	-1.23143400
H	-5.00452100	-0.28921700	-0.80417700
C	-5.19557400	-3.68360300	-0.92679200
H	-4.04086400	-5.02666100	0.30419000
H	-6.18542300	-2.09478700	-2.00048800
H	-5.71840300	-4.47835000	-1.45271500
H	-2.40815900	-1.00636300	1.87024300

IN B

Zero-point correction= 0.563933 (Hartree/Particle)
Thermal correction to Energy= 0.601931
Thermal correction to Enthalpy= 0.602876
Thermal correction to Gibbs Free Energy= 0.484017
Sum of electronic and zero-point Energies= -2467.973864
Sum of electronic and thermal Energies= -2467.935866
Sum of electronic and thermal Enthalpies= -2467.934921
Sum of electronic and thermal Free Energies= -2468.053780
Electronic energy -2465.49494448

C	-0.59653600	1.63744600	-4.16558400

C	-0.36646600	2.85811800	-3.51105000
C	-1.30124000	1.63867400	-5.37887300
C	-0.81393800	4.05624700	-4.06789100
H	0.15142600	2.86594400	-2.55509100
C	-1.75629500	2.83849100	-5.93037200
H	-1.50448800	0.70165600	-5.88923900
C	-1.51030600	4.04847800	-5.27875000
H	-0.62991500	4.99332100	-3.54934400
H	-2.30584200	2.82569000	-6.86806600
H	-1.86766800	4.98079400	-5.70793300
C	-1.00881900	-1.24260200	-4.06989800
C	-2.24092800	-1.49257400	-3.44052100
C	-0.63784400	-2.02383900	-5.17387200
C	-3.08813600	-2.49261000	-3.91703700
H	-2.53008100	-0.90408800	-2.57305700
C	-1.48601200	-3.02980700	-5.64384000
H	0.31544700	-1.85294300	-5.66437400
C	-2.71192400	-3.26424500	-5.01946100
H	-4.03761600	-2.67486400	-3.42084700
H	-1.18491200	-3.63075100	-6.49801500
H	-3.36897500	-4.04868500	-5.38564400
C	1.70014200	-0.14832700	-4.15972800
C	2.65548800	-0.89177400	-3.44771000
C	2.03857500	0.36609600	-5.42128400
C	3.91607600	-1.13203900	-3.99632400
H	2.41034100	-1.26477900	-2.45620000
C	3.30350800	0.13071800	-5.96363900
H	1.31905800	0.95924700	-5.97777700
C	4.24249700	-0.62143800	-5.25438400
H	4.64613200	-1.70812600	-3.43402800
H	3.55539500	0.53868100	-6.93914700
H	5.22753500	-0.80100000	-5.67725800
C	0.59653600	-1.63744600	4.16558400
C	1.30124000	-1.63867400	5.37887300
C	0.36646600	-2.85811800	3.51105000
C	1.75629500	-2.83849100	5.93037200
H	1.50448800	-0.70165600	5.88923900
C	0.81393800	-4.05624700	4.06789100
H	-0.15142600	-2.86594400	2.55509100
C	1.51030600	-4.04847800	5.27875000
H	2.30584200	-2.82569000	6.86806600
H	0.62991500	-4.99332100	3.54934400
H	1.86766800	-4.98079400	5.70793300
C	1.00881900	1.24260200	4.06989800
C	2.24092800	1.49257400	3.44052100
C	0.63784400	2.02383900	5.17387200
C	3.08813600	2.49261000	3.91703700
H	2.53008100	0.90408800	2.57305700
C	1.48601200	3.02980700	5.64384000
H	-0.31544700	1.85294300	5.66437400
C	2.71192400	3.26424500	5.01946100
H	4.03761600	2.67486400	3.42084700
H	1.18491200	3.63075100	6.49801500
H	3.36897500	4.04868500	5.38564400
C	-1.70014200	0.14832700	4.15972800
C	-2.03857500	-0.36609600	5.42128400
C	-2.65548800	0.89177400	3.44771000
C	-3.30350800	-0.13071800	5.96363900
H	-1.31905800	-0.95924700	5.97777700
C	-3.91607600	1.13203900	3.99632400
H	-2.41034100	1.26477900	2.45620000
C	-4.24249700	0.62143800	5.25438400
H	-3.55539500	-0.53868100	6.93914700
H	-4.64613200	1.70812600	3.43402800
H	-5.22753500	0.80100000	5.67725800
P	-0.04742700	-0.10050300	3.37154800
P	0.04742700	0.10050300	-3.37154800
Cu	0.01831500	0.06082400	-1.14547900
Cu	-0.01831500	-0.06082400	1.14547900
H	-1.12404300	0.65467000	0.00960000
H	1.12404300	-0.65467000	-0.00960000

TS B

Zero-point correction= 0.821135 (Hartree/Particle)
Thermal correction to Energy= 0.874675
Thermal correction to Enthalpy= 0.875620
Thermal correction to Gibbs Free Energy= 0.719841
Sum of electronic and zero-point Energies= -3161.061522
Sum of electronic and thermal Energies= -3161.007982
Sum of electronic and thermal Enthalpies= -3161.007038
Sum of electronic and thermal Free Energies= -3161.162817
Electronic energy -3158.48725003

Cu	1.52952400	-0.04913100	-0.40829700
H	1.11417200	-1.61191300	-0.40883200
C	-0.52539900	-2.41383200	0.14547200
C	0.02846500	-2.67950000	1.50679400
C	1.06232100	-3.62633100	1.67850800
C	-0.50331500	-2.01571800	2.62262000
C	1.54641200	-3.87563300	2.96507100
C	-0.00847100	-2.27363200	3.89935200
H	-1.30957300	-1.30514700	2.47220600
C	1.01978300	-3.20453000	4.07246800
H	2.33908000	-4.60861400	3.10101100
H	-0.42870100	-1.75790600	4.75896900
H	1.40177500	-3.41826700	5.06773800
C	-0.42996700	-3.54716200	-0.91622300
H	0.04060100	-3.08673800	-1.78778100
C	1.60311300	-4.31833400	0.45656200
H	2.25776600	-3.62511100	-0.09259200
H	2.20427700	-5.18985400	0.74218400
C	0.44031700	-4.74412600	-0.45356000
H	0.81280900	-5.27773200	-1.33609300
H	-0.18765500	-5.45484200	0.09830800
C	-1.81137400	-4.02816600	-1.34372700
C	-2.74900200	-4.48950200	-0.40593500
C	-2.15440900	-4.07470700	-2.70013300
C	-3.98793000	-4.98201600	-0.81328900
H	-2.50794800	-4.45593300	0.65388100
C	-3.39141100	-4.57637700	-3.11525900
H	-1.44263600	-3.71737100	-3.44102100
C	-4.31342200	-5.03288500	-2.17221100
H	-4.70045000	-5.32970200	-0.06945400
H	-3.63084800	-4.61089700	-4.17530800
H	-5.27522500	-5.42688800	-2.49057100
O	-1.39527900	-1.52067500	-0.03137700
H	0.38014400	1.16153500	-0.31033500
Cu	-1.08963800	0.53674100	-0.10309600
P	-3.13030500	1.53033700	0.04630300
C	-3.12056100	3.35050700	-0.25790400
C	-2.19106800	3.85473100	-1.18197400
C	-3.99605000	4.24189300	0.38141100
C	-2.15165400	5.21845900	-1.47448000
H	-1.48903300	3.17617300	-1.65982100
C	-3.94855300	5.60763200	0.09380800
H	-4.71044100	3.87082900	1.11055200
C	-3.02979400	6.09755100	-0.83667500
H	-1.42780200	5.59445700	-2.19266000
H	-4.62945200	6.28813800	0.59853900
H	-2.99354700	7.16097100	-1.05822100
C	-3.90561400	1.37222000	1.71367600
C	-3.08576700	1.58545400	2.83611100
C	-5.25010800	1.02763900	1.91195300
C	-3.60449100	1.47194000	4.12543700
H	-2.03738400	1.84021600	2.69766500
C	-5.76490200	0.90515300	3.20535000
H	-5.89553900	0.84998400	1.05741800
C	-4.94630500	1.12908700	4.31291500
H	-2.95919800	1.64316600	4.98299600

H	-6.80818600	0.63378700	3.34431200
H	-5.34911700	1.03230300	5.31761300
C	-4.39430100	0.87741200	-1.12623100
C	-4.30295600	-0.47663100	-1.49246900
C	-5.42495500	1.66605900	-1.66207000
C	-5.24135200	-1.03235000	-2.36470500
H	-3.49523200	-1.09198400	-1.10425100
C	-6.35540800	1.10684100	-2.53953400
H	-5.49733800	2.71772500	-1.40146300
C	-6.26682200	-0.24276500	-2.88877000
H	-5.15736400	-2.08049700	-2.63719000
H	-7.14717000	1.72716900	-2.95184300
H	-6.99175700	-0.67492900	-3.57399000
P	3.71063500	0.65918400	-0.38492300
C	4.41585100	0.44860000	1.30824500
C	4.08640500	2.42029400	-0.80618800
C	4.85264200	-0.29693400	-1.47685700
C	3.94065000	-0.62747200	2.07800300
C	5.37201500	1.31207700	1.86481600
C	3.12492200	3.38598100	-0.46397700
C	5.26373000	2.83299900	-1.44941900
C	4.37035200	-0.68372100	-2.73840300
C	6.15473800	-0.66620500	-1.10784200
C	4.42437000	-0.84272900	3.36878100
H	3.18327700	-1.29125200	1.66828200
C	5.85140500	1.09578000	3.15868300
H	5.73764300	2.15903900	1.29197300
C	3.34739500	4.73557800	-0.73903900
H	2.19569900	3.07158700	0.00449800
C	5.47917700	4.18361700	-1.73287400
H	6.01160500	2.09948800	-1.73538900
C	5.17811900	-1.40447700	-3.61790500
H	3.35287000	-0.42845600	-3.02503400
C	6.95918400	-1.39708900	-1.98566600
H	6.53988000	-0.39047100	-0.13056300
C	5.38103200	0.01805200	3.91168800
H	4.03813800	-1.67503300	3.95060000
H	6.59050400	1.77344200	3.57848700
C	4.52452900	5.13741300	-1.37483800
H	2.59538400	5.47141100	-0.46620900
H	6.39399000	4.48892100	-2.23451200
C	6.47496200	-1.76388200	-3.24247000
H	4.79071800	-1.69540200	-4.59079700
H	7.96459100	-1.68016700	-1.68461800
H	5.75266500	-0.14508900	4.92005600
H	4.69371500	6.18783700	-1.59717800
H	7.10171700	-2.33369800	-3.92341700

Explored heteroligated reduction transition states

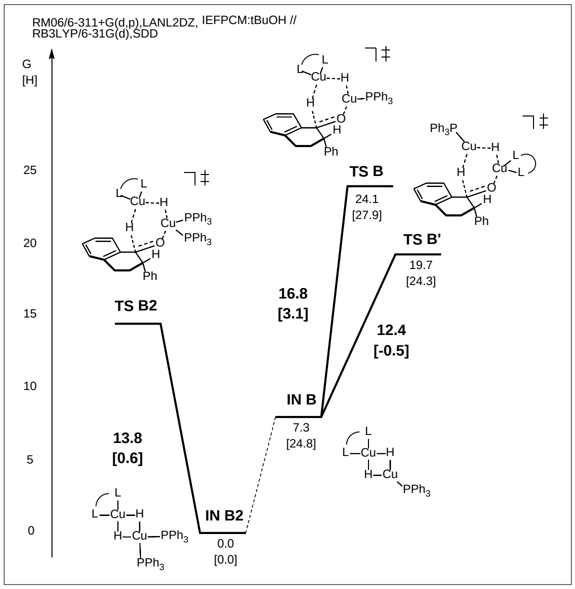

TS_B2 corresponds to **LP_TS** in the previous section in the previous section.

IN B2

Zero-point correction= 1.019966 (Hartree/Particle)
Thermal correction to Energy= 1.086140
Thermal correction to Enthalpy= 1.087084
Thermal correction to Gibbs Free Energy= 0.912691
Sum of electronic and zero-point Energies= -4155.866002
Sum of electronic and thermal Energies= -4155.799827
Sum of electronic and thermal Enthalpies= -4155.798883
Sum of electronic and thermal Free Energies= -4155.973277
Electronic energy -4153.39733126

C	5.02523500	2.20959900	-1.66987000
C	4.61970000	1.30960900	-0.66282200
C	5.53576500	0.33408000	-0.21267900
C	6.81888600	0.30878100	-0.79643400
C	7.19037500	1.21195600	-1.79251400
C	6.29596800	2.17804000	-2.24290400
H	7.52402300	-0.44927300	-0.47107600
H	8.18497900	1.15398100	-2.22658300
H	6.55644000	2.89338500	-3.01604400
C	5.29032200	-0.67169000	0.87137200
C	4.24173200	-1.61622800	0.87826500
C	6.24866900	-0.73506100	1.90246000
C	4.20683000	-2.59246500	1.89478900
C	6.18275800	-1.70561100	2.90293700
H	7.05047300	-0.00356000	1.91582400
C	5.16474100	-2.65481500	2.90689900
H	6.93402500	-1.71887700	3.68810000
H	5.09502800	-3.42468700	3.66834000
C	2.81060300	2.88783500	-1.60228400
H	2.32713600	3.83827300	-1.37029500
H	2.25069900	2.39830400	-2.40633100
C	2.15358600	-3.14039500	0.95929800
H	1.33649800	-2.70703500	1.54393300
H	1.79228500	-4.04231500	0.46177000
O	4.14233000	3.16476100	-2.07189200
O	3.21191300	-3.51875100	1.86115700
C	3.39491700	-2.80022600	-1.74955100
C	2.13372600	-3.30844500	-2.47339700
H	1.44971500	-2.48500500	-2.70828400
H	1.58471800	-4.04360800	-1.87677800
H	2.42287000	-3.79394000	-3.41537800

C	4.31490300	-3.97475000	-1.38031000
H	4.61754700	-4.50879400	-2.29177000
H	3.82155400	-4.69840100	-0.72266800
H	5.22533400	-3.63008300	-0.87882200
C	4.13964300	-1.82668900	-2.68256600
H	4.40536600	-2.34739900	-3.61269700
H	5.06478300	-1.45199500	-2.23540800
H	3.51455800	-0.96673500	-2.94736700
C	3.10188300	2.85819100	1.37224400
C	1.77690300	3.62395600	1.54361200
H	1.81919900	4.22272200	2.46336600
H	0.92417300	2.94419500	1.62674600
H	1.58008600	4.31290500	0.71494400
C	3.32690200	1.95626400	2.60103300
H	3.36901000	2.57443400	3.50823100
H	4.26751800	1.39994500	2.53378000
H	2.51299900	1.23237700	2.71826400
C	4.26628800	3.85236000	1.22696400
H	4.31221000	4.49491000	2.11690500
H	4.14803800	4.50267400	0.35342600
H	5.22891900	3.33916500	1.13601000
P	2.78137700	-1.81985700	-0.22325300
P	2.89300400	1.70677000	-0.14367000
Cu	1.31547000	-0.00375200	-0.24261600
H	0.15446200	-0.17528700	1.04522200
H	0.09181900	0.04423400	-1.47607400
P	-2.28827200	1.96530600	0.00814100
C	-1.50196500	3.37471600	-0.90853200
C	-0.92587500	3.09712900	-2.16019500
C	-1.48713300	4.69710000	-0.43899000
C	-0.38004400	4.12349300	-2.93488400
H	-0.88342600	2.06994200	-2.51095900
C	-0.92028900	5.71824300	-1.20512200
H	-1.91355600	4.93562700	0.52994400
C	-0.37323300	5.43668600	-2.45898000
H	0.05167300	3.89263100	-3.90555000
H	-0.91308300	6.73582600	-0.82266200
H	0.05932500	6.23363200	-3.05835600
C	-2.46823800	2.60708600	1.73957700
C	-1.57475700	2.13819900	2.71533200
C	-3.45890900	3.52978600	2.11709400
C	-1.66005700	2.59345300	4.03350800
H	-0.82215300	1.40426200	2.43443700
C	-3.54473400	3.98041400	3.43532600
H	-4.17297700	3.88872000	1.38170600
C	-2.64376300	3.51477100	4.39647700
H	-0.95927500	2.22142300	4.77681700
H	-4.31773900	4.69301900	3.71191400
H	-2.71342200	3.86478000	5.42330400
C	-4.04115000	2.05045900	-0.60445600
C	-5.03451700	1.34649900	0.10018100
C	-4.40367800	2.71264900	-1.78727200
C	-6.34929600	1.31238300	-0.36235500
H	-4.78121100	0.82512500	1.01785500
C	-5.72099200	2.67112800	-2.25265700
H	-3.66023800	3.26909100	-2.34847500
C	-6.69803300	1.97268400	-1.54333500
H	-7.09915700	0.76272100	0.20045600
H	-5.98124900	3.19472700	-3.16928600
H	-7.72236400	1.94327600	-1.90542300
Cu	-1.07401400	-0.06489800	-0.19220900
P	-2.49905400	-1.92250000	-0.11846600
C	-3.71072900	-1.82186700	1.28551800
C	-5.03877300	-2.26951500	1.22025800
C	-3.25657800	-1.23410300	2.47904500
C	-5.88843900	-2.13515400	2.32069900
H	-5.41978400	-2.71146000	0.30566400
C	-4.10355900	-1.11049700	3.58241100
H	-2.23762200	-0.85981000	2.53327900
C	-5.42373300	-1.55880200	3.50490700
H	-6.91631000	-2.48240000	2.25047300
H	-3.73391400	-0.64862400	4.49397600
H	-6.08782800	-1.45405900	4.35892500
C	-3.55309800	-2.25196800	-1.60518600
C	-3.69664500	-1.22216900	-2.54649700
C	-4.20509400	-3.47557000	-1.84010200
C	-4.48563100	-1.40092300	-3.68529900
H	-3.18024300	-0.28058200	-2.38655500
C	-4.98967900	-3.65538200	-2.97955000
H	-4.08827500	-4.29568200	-1.13776500
C	-5.13386700	-2.61677500	-3.90354900
H	-4.58699100	-0.58993100	-4.40138500
H	-5.48530900	-4.60816400	-3.14791300
H	-5.74344400	-2.75941400	-4.79212900
C	-1.73665000	-3.59377800	0.16507100
C	-1.68058600	-4.18560500	1.43630600
C	-1.15262000	-4.27682900	-0.91569600
C	-1.07066800	-5.42886700	1.62039300
H	-2.12537900	-3.68351300	2.28927200
C	-0.55864600	-5.52692300	-0.73377500
H	-1.17709900	-3.83828900	-1.90903700
C	-0.51378700	-6.10836600	0.53633900
H	-1.04238100	-5.86936600	2.61364200
H	-0.13291600	-6.04789000	-1.58768800
H	-0.05143400	-7.08164100	0.67748000

IN B

Zero-point correction= 0.743626 (Hartree/Particle)
Thermal correction to Energy= 0.792411
Thermal correction to Enthalpy= 0.793355
Thermal correction to Gibbs Free Energy= 0.654407
Sum of electronic and zero-point Energies= -3119.855255
Sum of electronic and thermal Energies= -3119.806470
Sum of electronic and thermal Enthalpies= -3119.805526
Sum of electronic and thermal Free Energies= -3119.944474
Electronic energy -3117.44927822

C	3.93474400	2.53541200	-1.67624900
C	3.78079500	1.53822400	-0.69167200
C	4.80723600	0.58441100	-0.51586200
C	5.94542600	0.67752400	-1.34134100
C	6.07135500	1.67730700	-2.30681300
C	5.06746400	2.62440700	-2.48467600
H	6.73500200	-0.05805700	-1.22551000
H	6.95999900	1.71119900	-2.93133400
H	5.13841700	3.41051700	-3.22910100
C	4.83751400	-0.48742800	0.53358400
C	3.86793500	-1.49943400	0.70777200
C	5.97807800	-0.51464500	1.36053200
C	4.07876000	-2.48690400	1.69165900
C	6.16085700	-1.50604400	2.32559400
H	6.72366500	0.26567500	1.24600100
C	5.21380600	-2.51026900	2.50144300
H	7.04920500	-1.48851300	2.95121200
H	5.32959000	-3.29160400	3.24526700
C	1.73325700	3.04430700	-1.15404300
H	1.26121200	3.93444300	-0.73183400
H	1.05767400	2.59575200	-1.89011400
C	1.91179700	-3.12499300	1.16576700
H	1.20863400	-2.71957500	1.90101100
H	1.49534500	-4.04151600	0.74134200
O	2.94036400	3.45452500	-1.82161400
O	3.14004600	-3.46297400	1.83514700
C	2.62129000	-2.77748800	-1.71548500
C	1.27741000	-3.37874900	-2.17500800
H	0.51101000	-2.60409400	-2.29009400
H	0.89950500	-4.13282200	-1.47417200
H	1.41120100	-3.87408500	-3.14570900
C	3.66654000	-3.88636600	-1.51241400

H	3.81916000	-4.42749000	-2.45620200
H	3.35610700	-4.61808400	-0.75863500
H	4.63373900	-3.47623800	-1.20351900
C	3.11238700	-1.77691200	-2.77905600
H	3.25395700	-2.29923000	-3.73485200
H	4.06930100	-1.32215700	-2.50334900
H	2.38506800	-0.97328800	-2.93836100
C	2.45959600	2.74613000	1.72751700
C	1.08322800	3.27184800	2.18360400
H	1.18744100	3.77576600	3.15353200
H	0.36117100	2.45578100	2.29874000
H	0.66359800	4.00171400	1.48113500
C	3.00542300	1.77840400	2.79482400
H	3.11841100	2.31161200	3.74843800
H	3.98581100	1.37556700	2.52065800
H	2.32331700	0.93675400	2.95766800
C	3.44033700	3.91196400	1.52165800
H	3.56110900	4.46390000	2.46379200
H	3.08910500	4.62234100	0.76535000
H	4.42955300	3.55658600	1.21456900
P	2.25014400	-1.79609700	-0.11967600
P	2.14767900	1.73996500	0.13414800
Cu	0.75360800	-0.07251200	0.00735200
H	-0.41318000	-0.35403600	1.27911100
H	-0.44180400	0.16000000	-1.24707200
Cu	-1.56213200	-0.10803500	0.02785700
P	-3.79012000	-0.04605900	0.02115400
C	-4.42403300	1.63535100	-0.40884400
C	-3.75830200	2.33532400	-1.43073300
C	-5.50928800	2.24581600	0.23669700
C	-4.18167600	3.60971900	-1.80733300
H	-2.90277700	1.87827400	-1.92202100
C	-5.92410000	3.52701400	-0.13632700
H	-6.02943200	1.72575100	1.03494700
C	-5.26473100	4.20953800	-1.15951300
H	-3.65898500	4.13777900	-2.60052100
H	-6.76373800	3.98992800	0.37570600
H	-5.58888800	5.20618400	-1.44735100
C	-4.64079100	-0.46781800	1.60739100
C	-3.92413300	-0.28873400	2.80109700
C	-5.95576400	-0.95637800	1.66893100
C	-4.51802100	-0.57239200	4.03219000
H	-2.89327200	0.05424400	2.75675800
C	-6.54462700	-1.24671500	2.90092600
H	-6.51785400	-1.12002300	0.75397600
C	-5.82844300	-1.05147200	4.08431300
H	-3.95125900	-0.43067200	4.94860100
H	-7.56158000	-1.62898700	2.93540800
H	-6.28762000	-1.28104800	5.04233200
C	-4.59882100	-1.16091300	-1.20866400
C	-4.04564800	-2.43885400	-1.39333200
C	-5.72243500	-0.79236600	-1.96284500
C	-4.61328200	-3.33334400	-2.30019900
H	-3.16228900	-2.72835200	-0.82908300
C	-6.28440900	-1.68675000	-2.87730000
H	-6.15531800	0.19638900	-1.84329200
C	-5.73394200	-2.95822700	-3.04556000
H	-4.17413800	-4.31854900	-2.43270400
H	-7.15233400	-1.38680300	-3.45883900
H	-6.17122000	-3.65166700	-3.75898600

TS B

Zero-point correction= 1.000662 (Hartree/Particle)
Thermal correction to Energy= 1.064124
Thermal correction to Enthalpy= 1.065068
Thermal correction to Gibbs Free Energy= 0.893795
Sum of electronic and zero-point Energies= -3812.938882
Sum of electronic and thermal Energies= -3812.875420
Sum of electronic and thermal Enthalpies= -3812.874476
Sum of electronic and thermal Free Energies= -3813.045749
Electronic energy -3810.47137582

C	3.99229900	3.03055300	1.39502900
C	4.10670400	1.76521200	0.78453800
C	4.99768400	1.60679800	-0.29932700
C	5.73133700	2.73115200	-0.72609700
C	5.59492000	3.97317000	-0.10416400
C	4.72589300	4.13825100	0.97021000
H	6.41003200	2.62317200	-1.56628300
H	6.17008800	4.82104200	-0.46644400
H	4.60230600	5.09163900	1.47329900
C	5.30400500	0.31011900	-0.98723800
C	4.36305700	-0.51993000	-1.63400600
C	6.66145900	-0.06494400	-1.03314100
C	4.81467500	-1.68480700	-2.28663500
C	7.08001200	-1.22148400	-1.69256100
H	7.39207600	0.55918200	-0.52847800
C	6.16025100	-2.04804200	-2.33129900
H	8.13453800	-1.48391100	-1.69896100
H	6.45794000	-2.95459600	-2.84786400
C	2.25729800	2.03254400	2.56789000
H	2.11805700	1.81908100	3.63017800
H	1.29276500	2.30308000	2.12517200
C	2.55186300	-2.12446600	-2.51944300
H	2.22662000	-2.82284200	-1.74001300
H	1.90518600	-2.23699300	-3.39260800
O	3.14819100	3.15804300	2.45483800
O	3.89248300	-2.46682700	-2.91394400
C	2.21179300	0.73209600	-3.30860200
C	0.74435000	0.50064500	-3.72281400
H	0.05786800	0.66601500	-2.88463100
H	0.57413700	-0.51245700	-4.10632900
H	0.47541100	1.20057600	-4.52510700
C	3.15206100	0.44656700	-4.49041200
H	2.90644800	1.11401100	-5.32794300
H	3.06401000	-0.58366500	-4.85230600
H	4.19940300	0.61984700	-4.22239300
C	2.37745800	2.19128400	-2.84234000
H	2.11586100	2.87136600	-3.66448300
H	3.40767100	2.41126800	-2.54386900
H	1.72252200	2.41546900	-1.99313300
C	3.98726500	-0.38366600	2.88392400
C	2.98040800	-1.01063300	3.86926500
H	3.51481500	-1.66776800	4.56829100
H	2.22412200	-1.61078700	3.35200300
H	2.45959600	-0.25536100	4.46923500
C	4.72535600	-1.51361500	2.14040100
H	5.25880200	-2.14228400	2.86627600
H	5.46272900	-1.12712000	1.42984200
H	4.02527700	-2.15324700	1.59111500
C	4.99638400	0.49867400	3.63568000
H	5.53711200	-0.10606700	4.37676500
H	4.51079500	1.32108300	4.17220200
H	5.73683800	0.93289700	2.95593900
P	2.53085300	-0.39154800	-1.79196900
P	2.95343300	0.58039200	1.59661600
Cu	1.30843100	-0.33272700	0.20046200
H	0.64437500	-1.79836600	0.47182300
C	-1.21545100	-2.38635800	0.65584000
C	-1.03526200	-2.55824400	2.12611900
C	-0.20976600	-3.58951800	2.62460800
C	-1.72915400	-1.72719500	3.01867900
C	-0.08624600	-3.75214300	4.00632700
C	-1.59461200	-1.90086300	4.39436500
H	-2.37914400	-0.95564700	2.61869100
C	-0.76903500	-2.91357700	4.89120600
H	0.54837400	-4.54710700	4.39260700
H	-2.14100500	-1.25625000	5.07834400
H	-0.66567900	-3.05848900	5.96365900

C	-1.01337700	-3.63045500	-0.25705500
H	-0.28378600	-3.31970700	-1.00781600
C	0.51017800	-4.46359300	1.63471200
H	1.35757800	-3.90310400	1.21174000
H	0.91025100	-5.35760000	2.12788300
C	-0.44907700	-4.85933600	0.50128200
H	0.04729900	-5.52527800	-0.21499200
H	-1.27994400	-5.43132000	0.93320500
C	-2.29604600	-3.99674000	-0.99299400
C	-3.49535000	-4.23362000	-0.30256800
C	-2.29047700	-4.16009700	-2.38334100
C	-4.64976400	-4.61980800	-0.98221600
H	-3.52613200	-4.10642600	0.77690900
C	-3.44239500	-4.55569200	-3.06952500
H	-1.37093700	-3.98020400	-2.93616600
C	-4.62772400	-4.78702600	-2.37017600
H	-5.56862100	-4.79278300	-0.42763700
H	-3.41010400	-4.68455000	-4.14870200
H	-5.52557600	-5.09664700	-2.89885100
O	-1.88837100	-1.42951500	0.19632900
H	0.22285600	0.98884400	0.23727200
Cu	-1.31672600	0.59411000	0.15595000
P	-3.29620200	1.69317400	-0.10224700
C	-3.16308700	3.52567600	-0.27457400
C	-1.98218600	4.05132500	-0.82229900
C	-4.18413200	4.40788300	0.11380200
C	-1.83278800	5.42822100	-0.99550600
H	-1.17518900	3.37670000	-1.09639200
C	-4.02980700	5.78521000	-0.05391100
H	-5.09753800	4.01969700	0.55545600
C	-2.85602300	6.29723900	-0.61159100
H	-0.91238600	5.82139800	-1.41893800
H	-4.82613800	6.45800000	0.25372200
H	-2.73652400	7.37002800	-0.73831200
C	-4.46552600	1.48463400	1.31126700
C	-3.96158900	1.67780200	2.60984400
C	-5.80912800	1.11664800	1.15461000
C	-4.78637600	1.51916900	3.72273900
H	-2.91848900	1.95326200	2.74809000
C	-6.63149300	0.94943200	2.27233800
H	-6.21461800	0.95597700	0.16061000
C	-6.12455200	1.15161300	3.55625700
H	-4.38287500	1.67582900	4.71969600
H	-7.67036500	0.66060600	2.13553500
H	-6.76591900	1.01985300	4.42356900
C	-4.25226700	1.15106000	-1.58377400
C	-4.16285400	-0.20336000	-1.95195100
C	-5.04543700	2.01918500	-2.35046600
C	-4.87163000	-0.67847600	-3.05717000
H	-3.53599100	-0.88263200	-1.37965800
C	-5.74353400	1.53997500	-3.46060000
H	-5.11435600	3.07022600	-2.08731700
C	-5.66050400	0.19122400	-3.81326100
H	-4.79592800	-1.72867400	-3.32452400
H	-6.35100600	2.22244200	-4.04960300
H	-6.20502000	-0.17870400	-4.67838900

TS B'

Zero-point correction= 0.999859 (Hartree/Particle)
Thermal correction to Energy= 1.063664
Thermal correction to Enthalpy= 1.064608
Thermal correction to Gibbs Free Energy= 0.892106
Sum of electronic and zero-point Energies= -3812.936554
Sum of electronic and thermal Energies= -3812.872749
Sum of electronic and thermal Enthalpies= -3812.871805
Sum of electronic and thermal Free Energies= -3813.044308
Electronic energy -3810.47671835

C	5.30331800	0.63865800	1.08022900
C	4.42606600	-0.44750900	0.88505800
C	4.82717500	-1.51239600	0.05083300
C	6.10464400	-1.44799300	-0.53860600
C	6.95761100	-0.36493900	-0.31878300
C	6.56805800	0.69445500	0.49485700
H	6.41976000	-2.25609600	-1.19105700
H	7.93231600	-0.34408800	-0.79875500
H	7.20792800	1.55194300	0.67453800
C	4.02969100	-2.75222700	-0.22038100
C	2.73959000	-2.78231400	-0.79409600
C	4.66704200	-3.97920500	0.04778900
C	2.14828300	-4.03083600	-1.07623000
C	4.05509100	-5.20034900	-0.23983700
H	5.65469900	-3.96639600	0.49790000
C	2.78723400	-5.24223500	-0.81119900
H	4.57306000	-6.12765800	-0.01025900
H	2.28900800	-6.17679300	-1.04696400
C	3.47793100	1.59436900	2.13812200
H	3.30458800	1.93842000	3.16026900
H	2.96319200	2.25838000	1.43597000
C	0.27908700	-2.75416100	-1.57201100
H	-0.41188500	-2.75827300	-0.72172400
H	-0.29582000	-2.60230700	-2.48756400
O	4.89542500	1.65335300	1.88867500
O	0.91647100	-4.04184400	-1.65326300
C	2.08676700	-0.84867900	-3.01118600
C	0.86278600	-0.11772200	-3.60047300
H	0.50942100	0.67730600	-2.93504100
H	0.02373800	-0.79606100	-3.79442300
H	1.14150800	0.33930500	-4.55945900
C	2.51267400	-2.00481000	-3.92941200
H	2.76231100	-1.61348300	-4.92519600
H	1.71790100	-2.74831500	-4.05336400
H	3.39735700	-2.52044500	-3.54203700
C	3.24125200	0.15887400	-2.84549900
H	3.50907400	0.57163400	-3.82749600
H	4.13780800	-0.31158900	-2.42908100
H	2.95605300	0.99466000	-2.19787000
C	3.06977800	-1.04565900	3.49725900
C	1.99267000	-0.46012100	4.43313800
H	1.99572300	-1.00892400	5.38408500
H	0.99075600	-0.54215600	3.99687100
H	2.17711000	0.59527700	4.66662000
C	2.77855800	-2.54129300	3.26716700
H	2.81320300	-3.07333200	4.22743200
H	3.51515000	-3.00466200	2.60291600
H	1.78456700	-2.69552300	2.83229900
C	4.46736100	-0.87123900	4.11454500
H	4.51254100	-1.39465500	5.07924600
H	4.71221100	0.18091100	4.29607900
H	5.24692200	-1.29070200	3.47016800
P	1.57847500	-1.43030800	-1.26429600
P	2.87077600	-0.15302800	1.82325300
H	-1.38237800	1.53392000	-0.60326600
O	0.97166500	2.00939500	0.07682400
C	-0.06520700	2.70795900	-0.08262500
C	-0.86578000	3.17493300	1.09890700
C	-2.03098300	3.94966300	0.90918300
C	-0.44325200	2.86700500	2.39973300
C	-2.74084200	4.39563100	2.02690800
C	-1.16543700	3.31146900	3.50600100
H	0.45103800	2.26663100	2.52366000
C	-2.31778200	4.07952200	3.32087200
H	-3.63185100	5.00344300	1.88089000
H	-0.82952200	3.06469300	4.51007000
H	-2.88090200	4.43786800	4.17898300
Cu	0.91860400	-0.12130900	0.56488700
H	-0.55847700	-0.55163000	1.16814300
Cu	-1.71875800	0.15417500	0.20816100
P	-3.78734300	-0.79710200	0.04272400

C	-4.30088700	-2.03000800	1.32262900
C	-5.64039200	-2.35034200	1.59693700
C	-3.28601900	-2.66427200	2.05752200
C	-5.95560500	-3.29527400	2.57466300
H	-6.43951700	-1.85512000	1.05338900
C	-3.60389700	-3.61460200	3.02962200
H	-2.24883900	-2.39443400	1.87394000
C	-4.93828000	-3.93203400	3.28968100
H	-6.99662800	-3.53148200	2.77984400
H	-2.80836000	-4.09755500	3.59106900
H	-5.18614800	-4.66577700	4.05229800
C	-5.22902800	0.35224300	-0.05194400
C	-6.34234000	0.13117100	-0.87740000
C	-5.19332100	1.50691200	0.74689700
C	-7.40187700	1.04103700	-0.89505600
H	-6.37910400	-0.74789900	-1.51445600
C	-6.25754400	2.40922000	0.73482800
H	-4.32413300	1.70824700	1.36785200
C	-7.36369500	2.17859200	-0.08620300
H	-8.25664200	0.85998900	-1.54174500
H	-6.21484300	3.29809300	1.35858700
H	-8.18856700	2.88613800	-0.10184200
C	-3.86534400	-1.73040300	-1.55008000
C	-4.30674000	-3.05712100	-1.65699600
C	-3.40138800	-1.07413600	-2.70583600
C	-4.28917100	-3.71245200	-2.89163900
H	-4.66235600	-3.58298900	-0.77654100
C	-3.39599300	-1.72730700	-3.93817800
H	-3.03861700	-0.05188400	-2.63101700
C	-3.83747800	-3.05067600	-4.03373900
H	-4.63117700	-4.74206800	-2.95738000
H	-3.04202800	-1.20460700	-4.82312400
H	-3.82603800	-3.56201200	-4.99263400
C	-2.46307900	4.25413300	-0.49944100
H	-2.92391900	3.35687100	-0.93919700
H	-3.21733700	5.05023900	-0.50713700
C	-1.24201500	4.66458600	-1.33554000
H	-0.81062900	5.57200900	-0.89464400
H	-1.53904900	4.92738400	-2.35812700
C	-0.15758400	3.55838000	-1.39142200
H	-0.42491300	2.85497900	-2.18353000
C	1.19620900	4.15780700	-1.75115500
C	1.73514800	3.97565400	-3.03000500
C	1.91155800	4.94881800	-0.83835200
C	2.95071700	4.56236400	-3.39300600
H	1.19543400	3.36733200	-3.75253400
C	3.12761600	5.53125900	-1.19249000
H	1.51428700	5.10374600	0.16206000
C	3.65274600	5.34184500	-2.47415700
H	3.34725300	4.40729900	-4.39349900
H	3.66718500	6.13509900	-0.46701300
H	4.59970400	5.79783800	-2.75111000

5 NMR and HPLC data

^{1}H NMR spectra of **2a**:

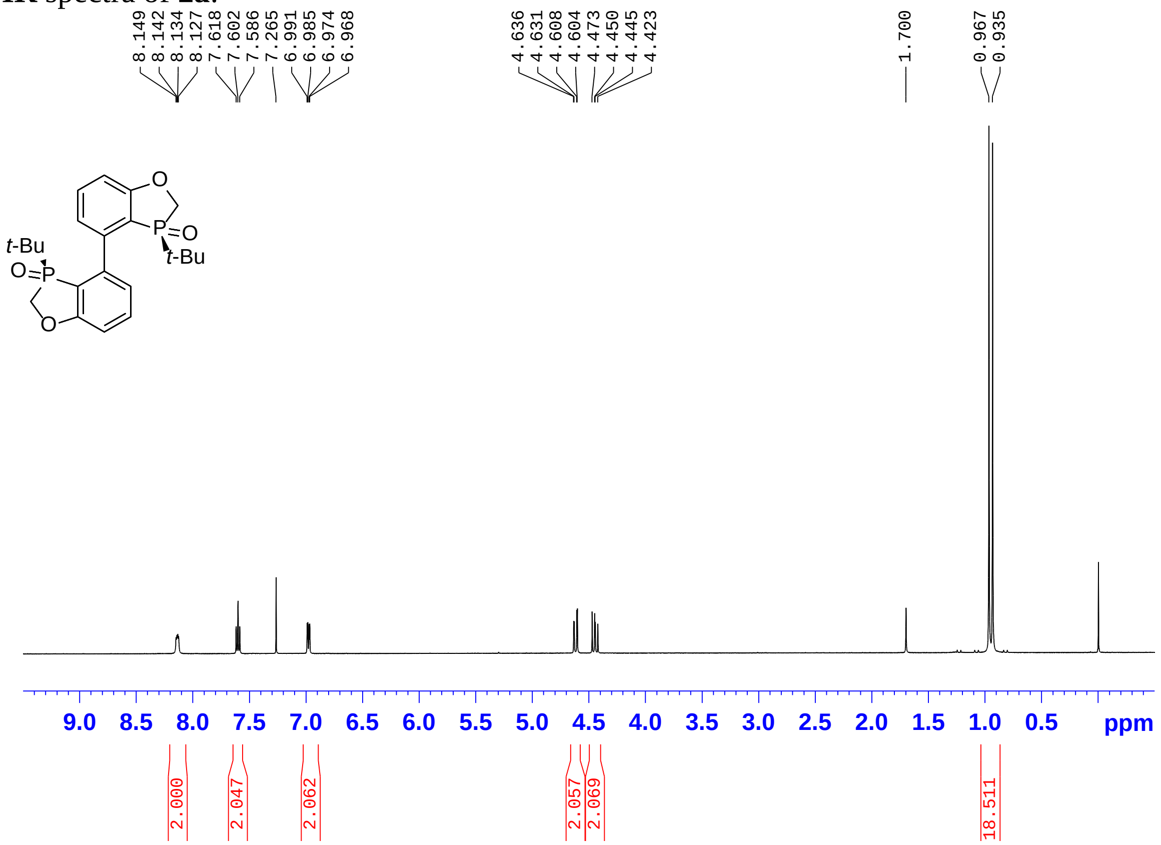

^{13}C NMR spectra of **2a**:

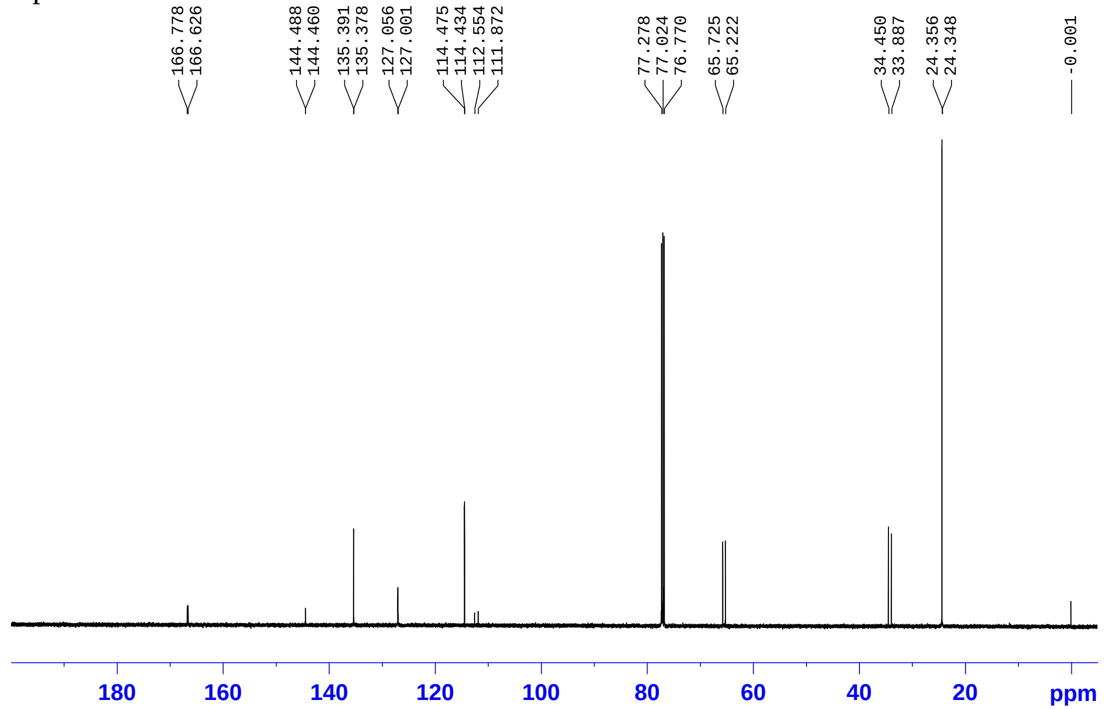

^{31}P NMR spectra of **2a**:

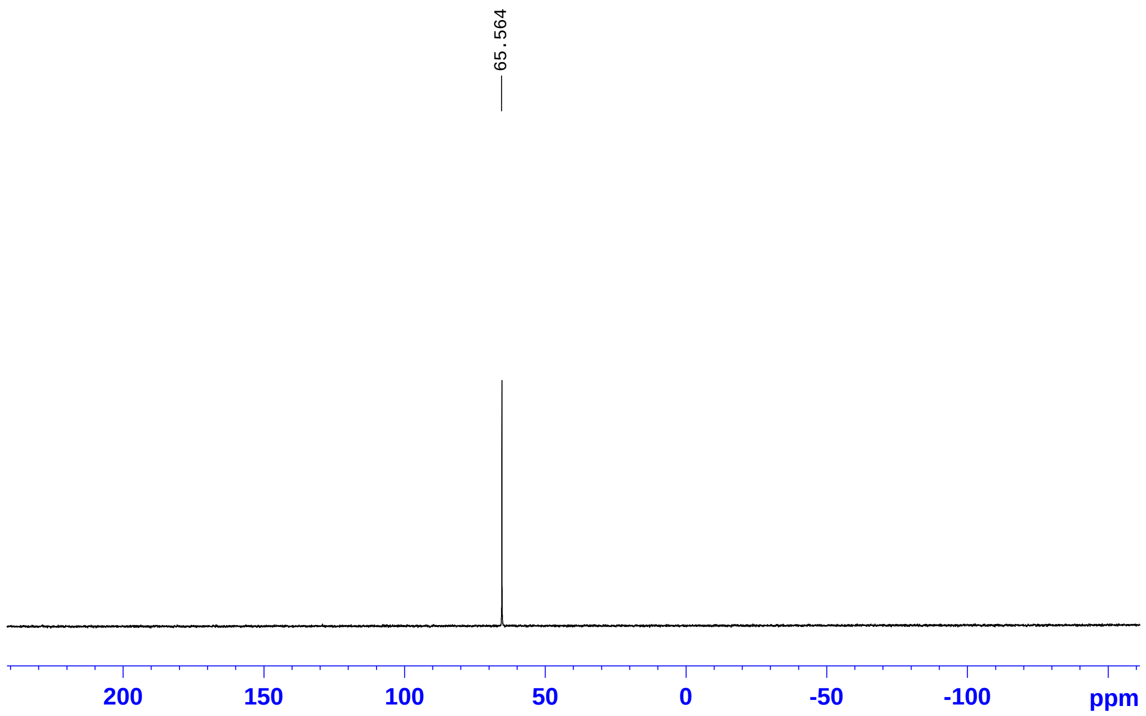
65.564
200
150
100
50
0
-50
-100
ppm

^{1}H NMR spectra of **3a**:

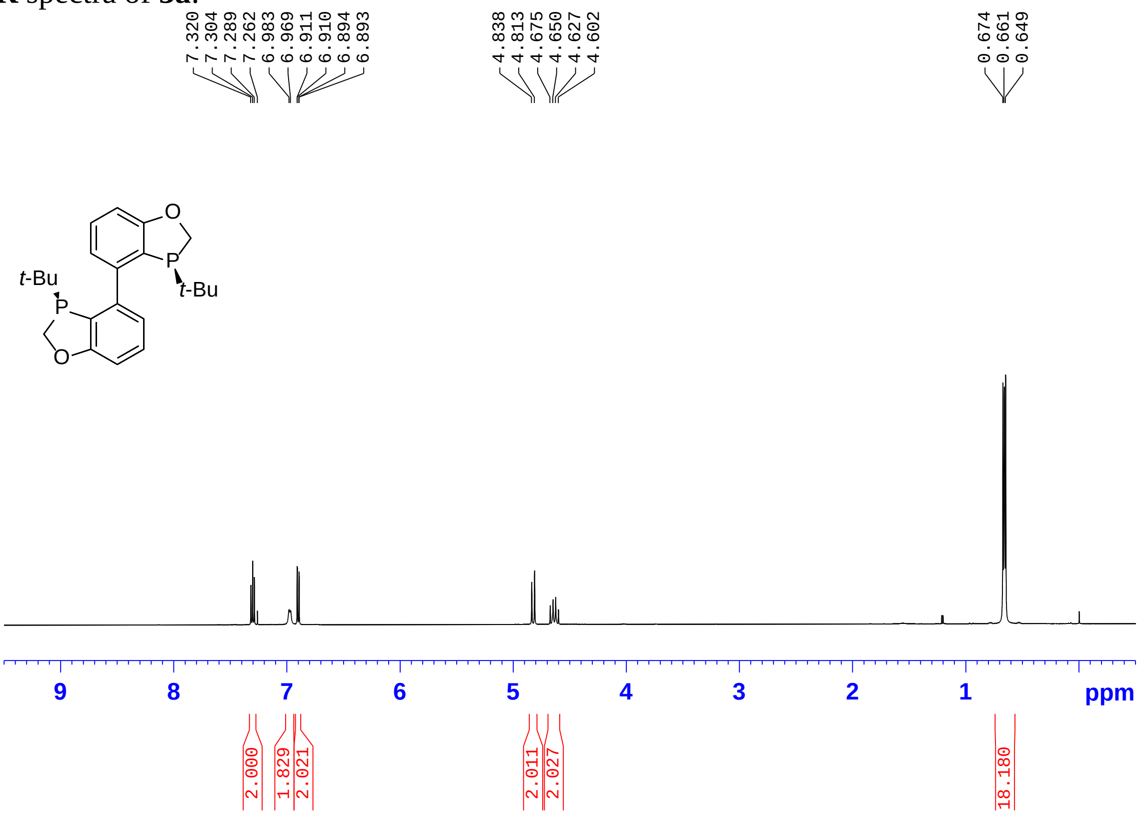

^{13}C NMR spectra of **3a**:

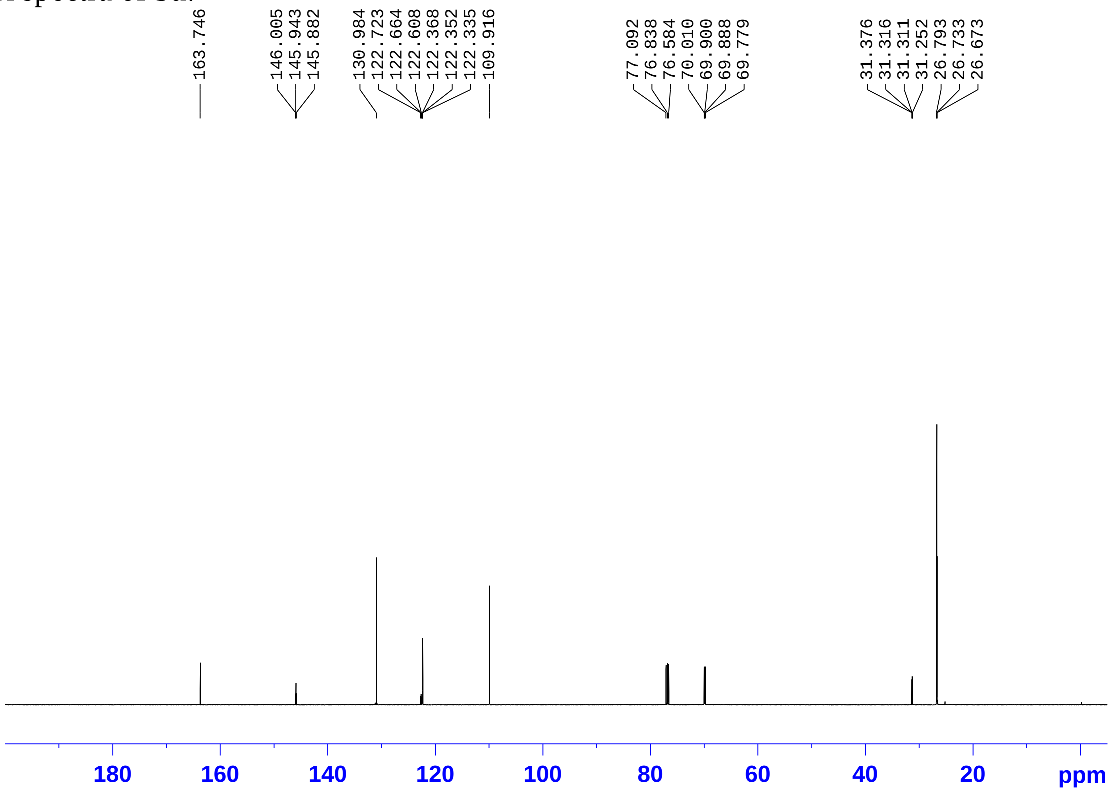

^{31}P NMR spectra of **3a**:

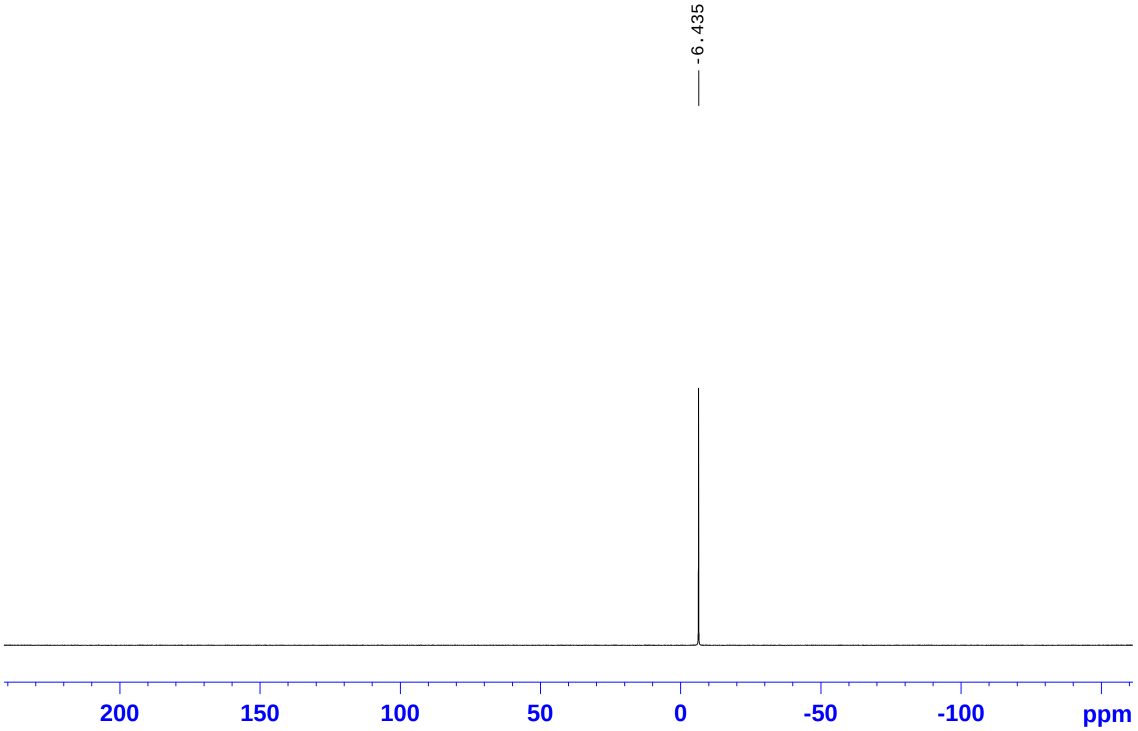
-6.435
200
150
100
50
0
-50
-100
ppm

^{1}H NMR spectra of **2c**:

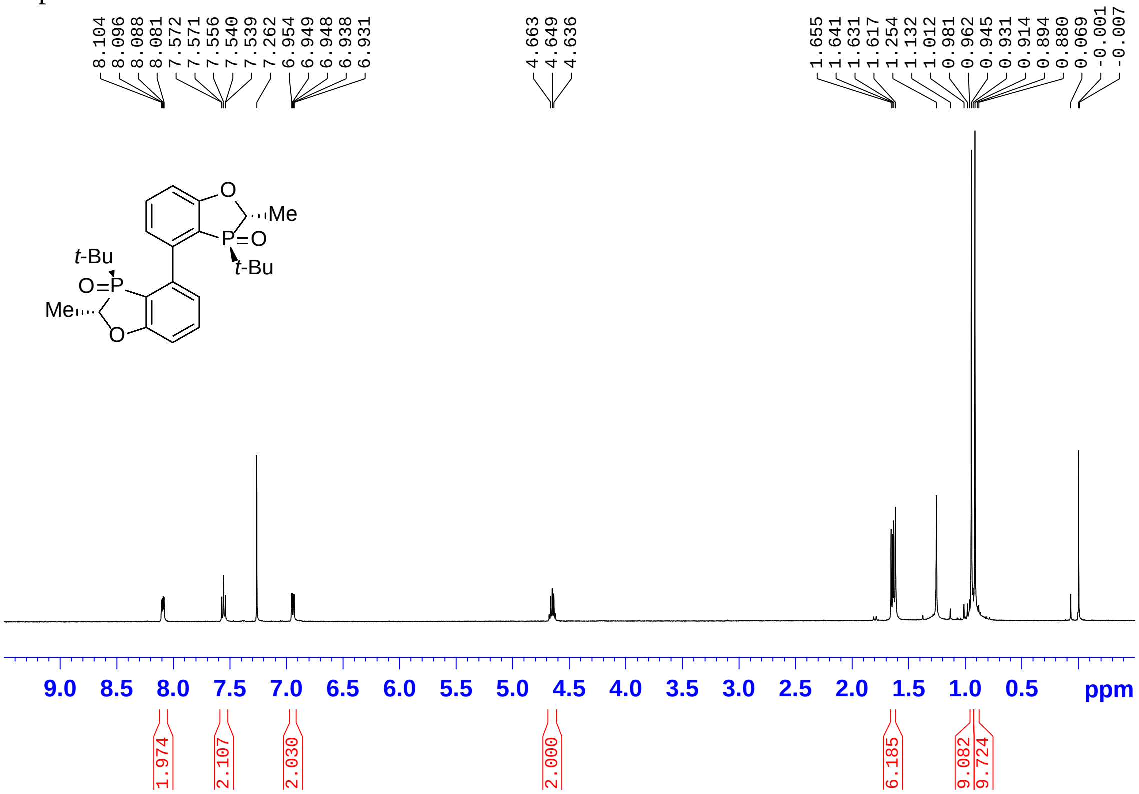

^{13}C NMR spectra of **2c**:

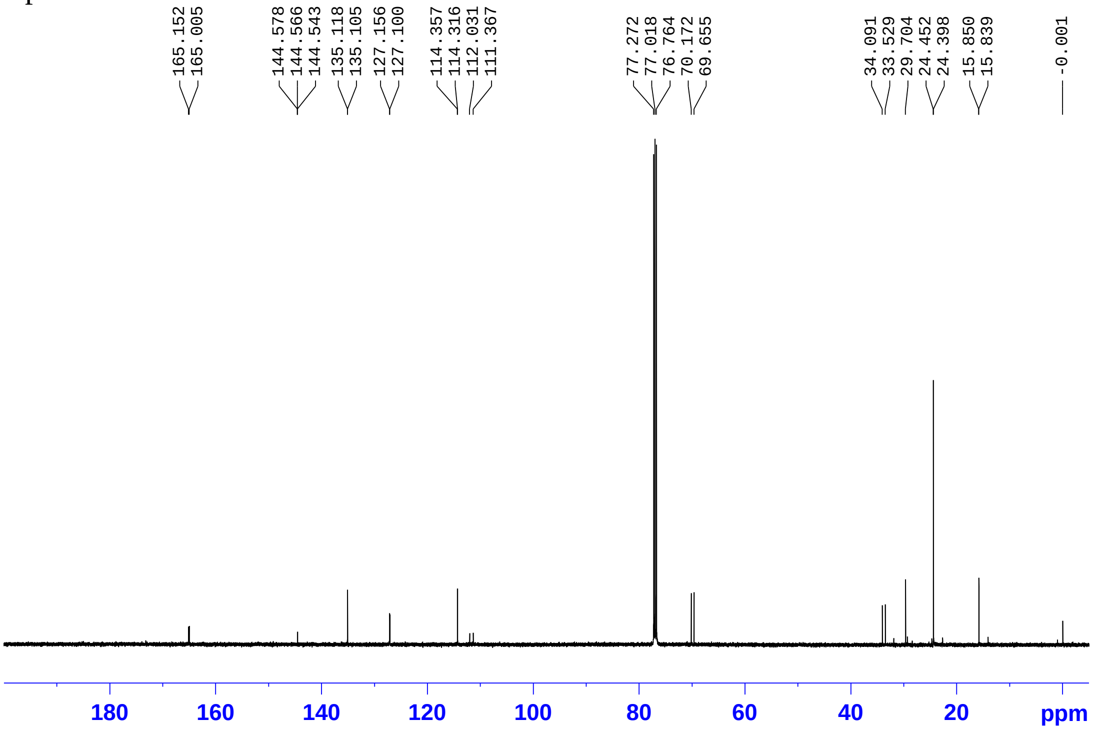

^{31}P NMR spectra of **2c**:

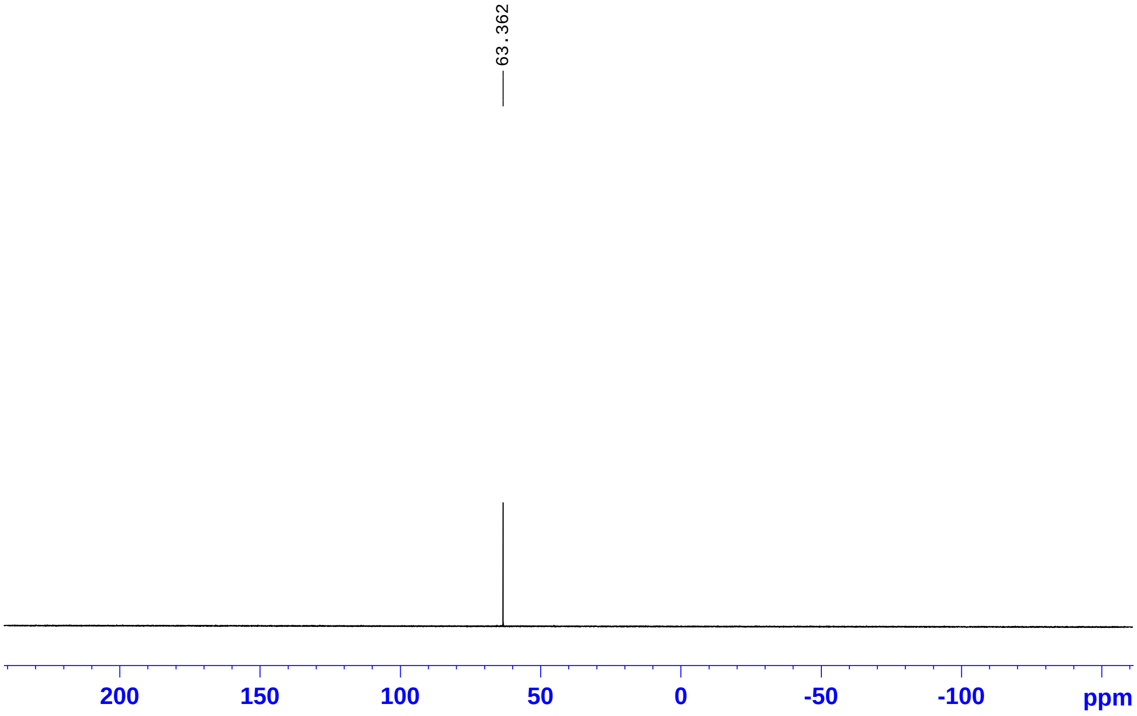
63.362
200
150
100
50
0
-50
-100
ppm

^{1}**H NMR** spectra of **3c**:

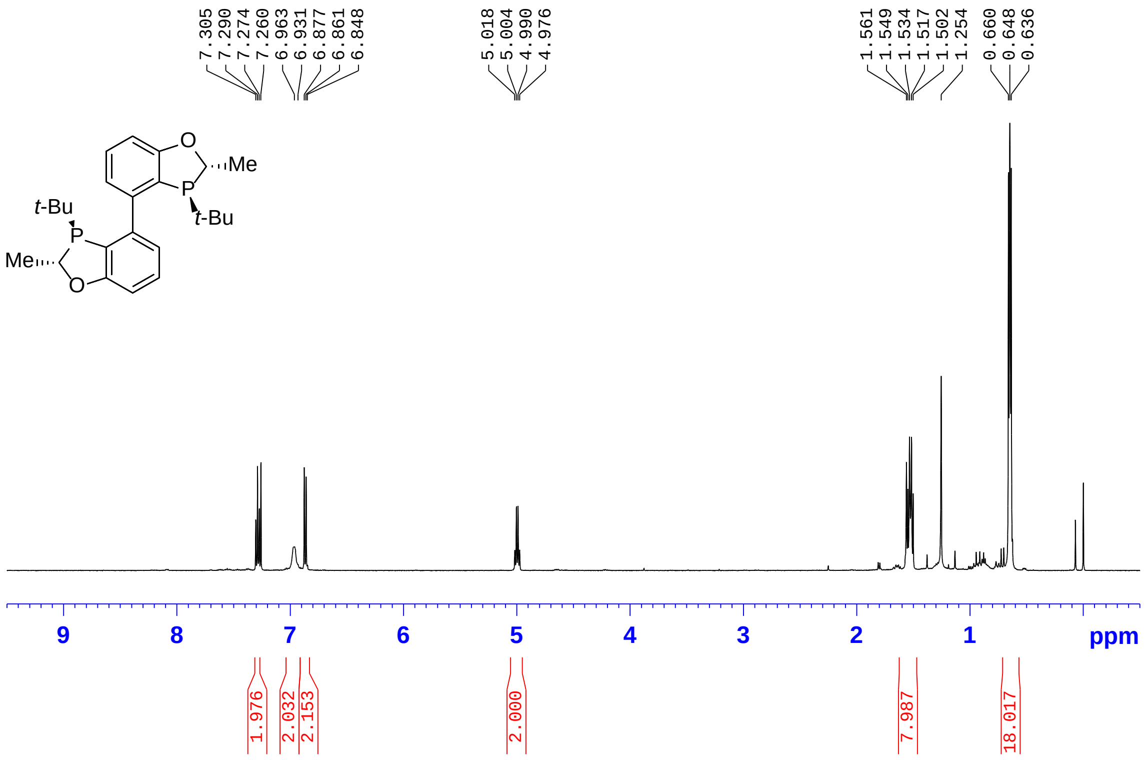

^{13}**C NMR** spectra of **3c**:

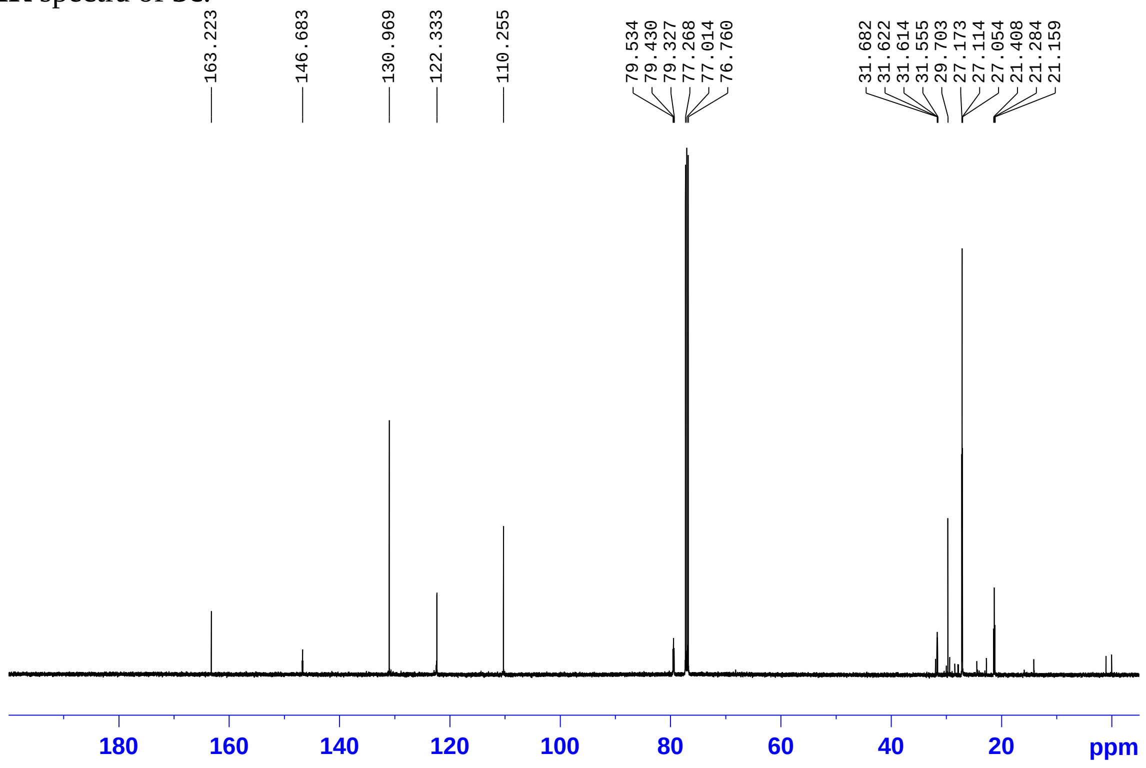

^{31}**P NMR** spectra of **3c**:

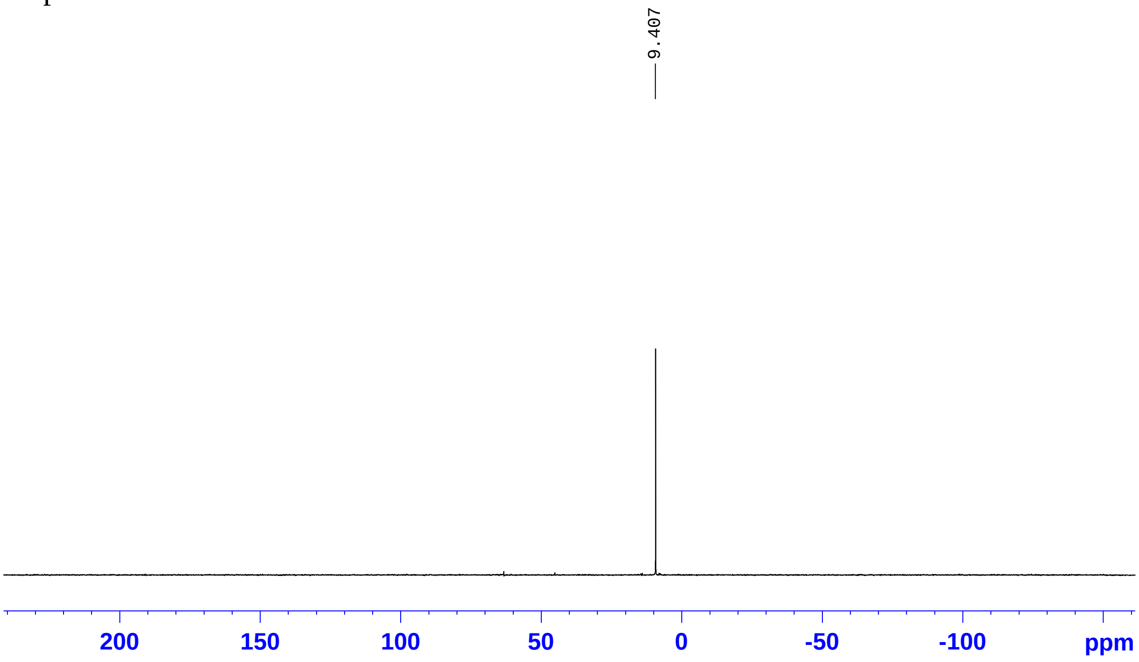

^{1}H NMR spectra of **2d**:

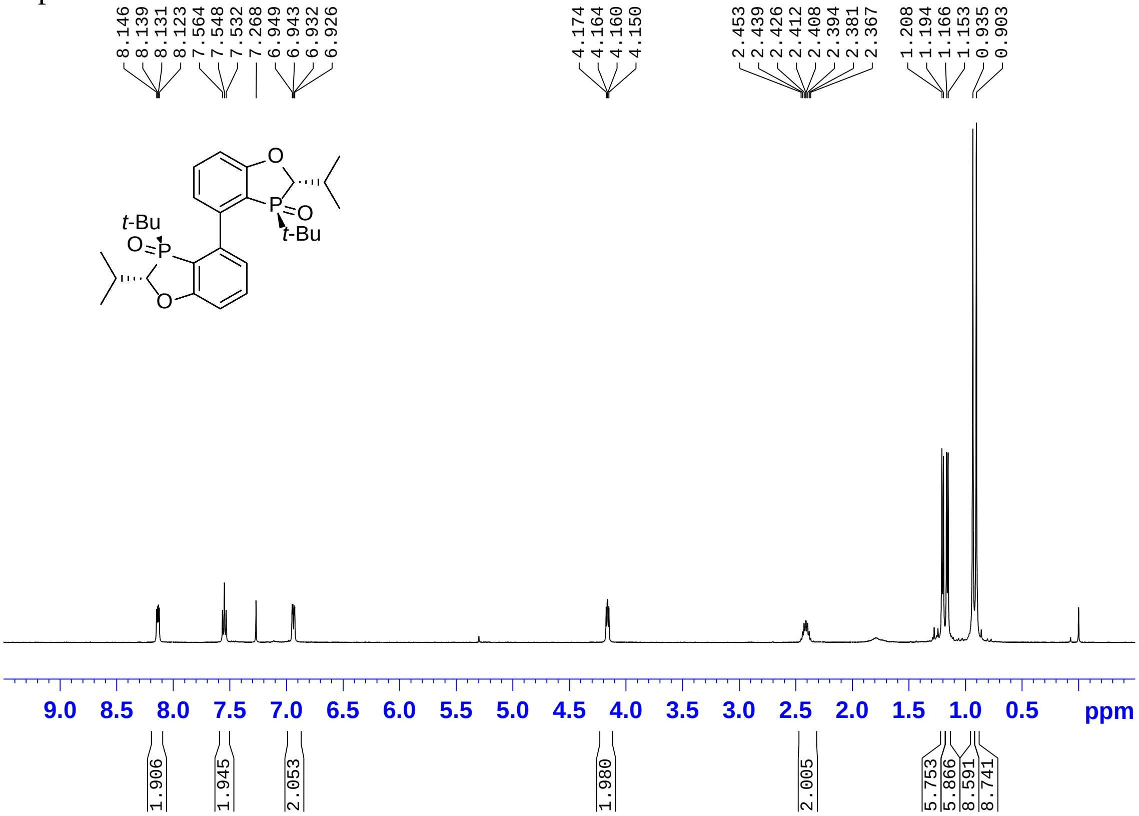

[31]C NMR spectra of **2d**:

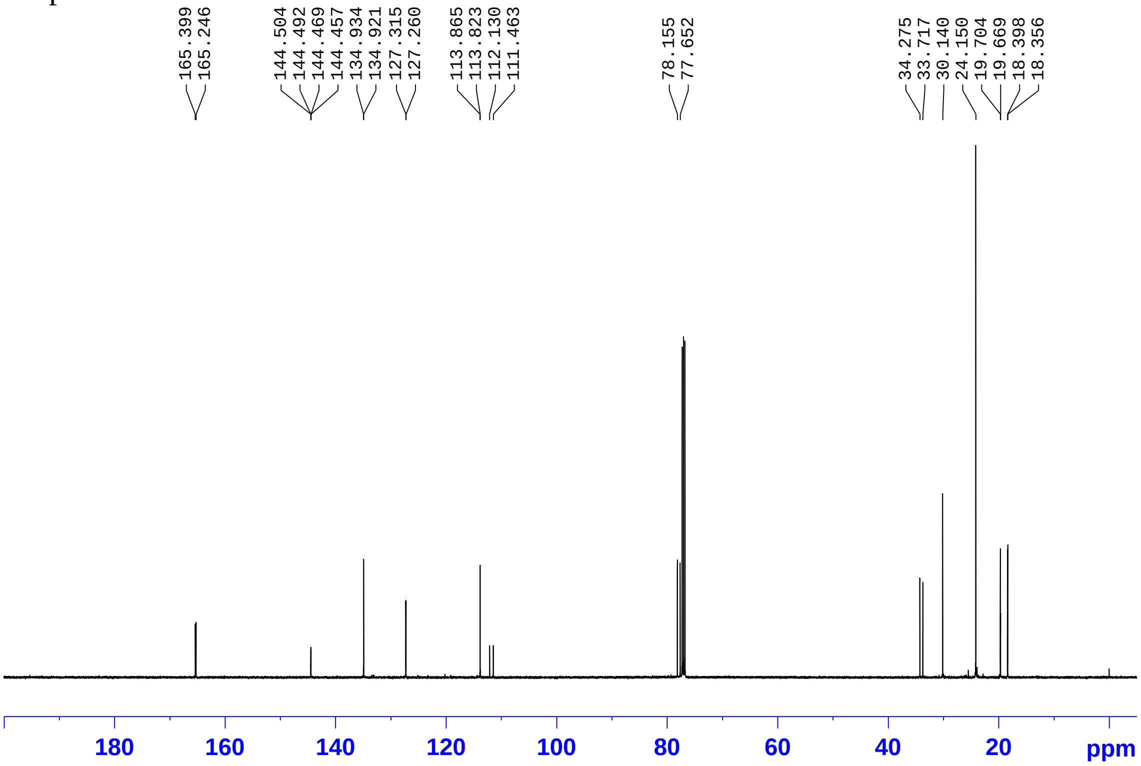

^{31}**P NMR** spectra of **2d**:

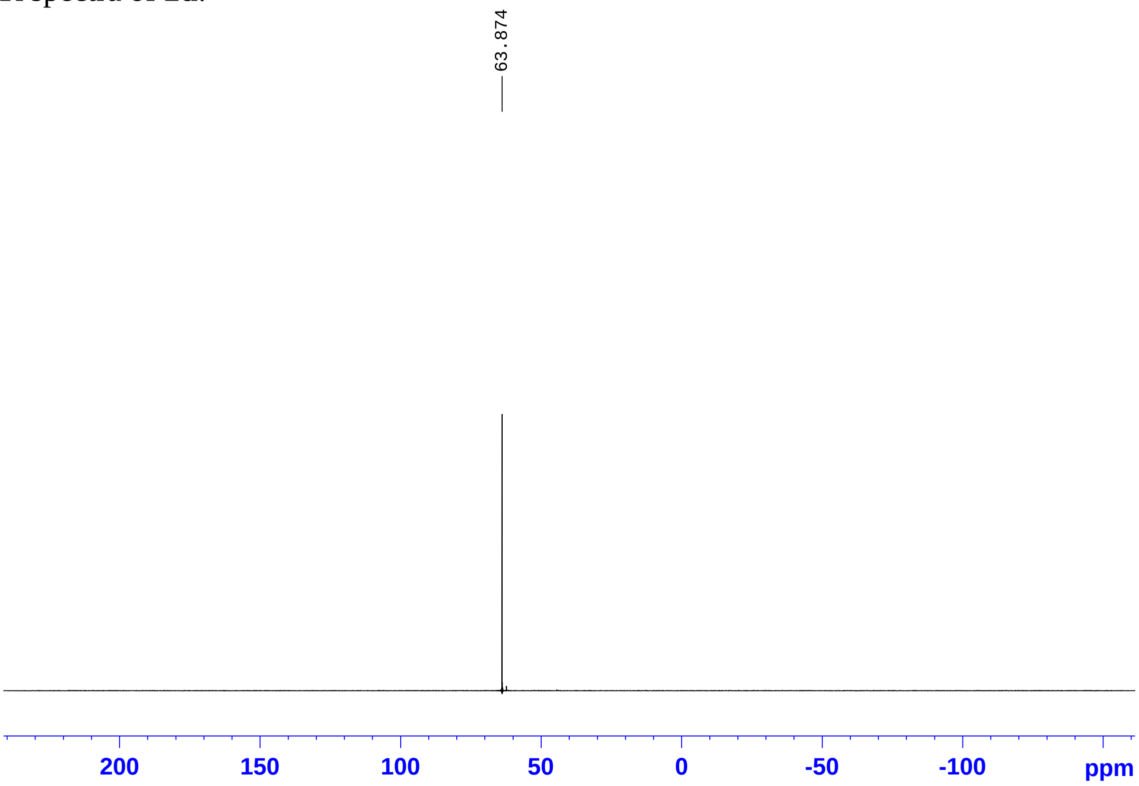

^{1}H **NMR** spectra of **3d**:

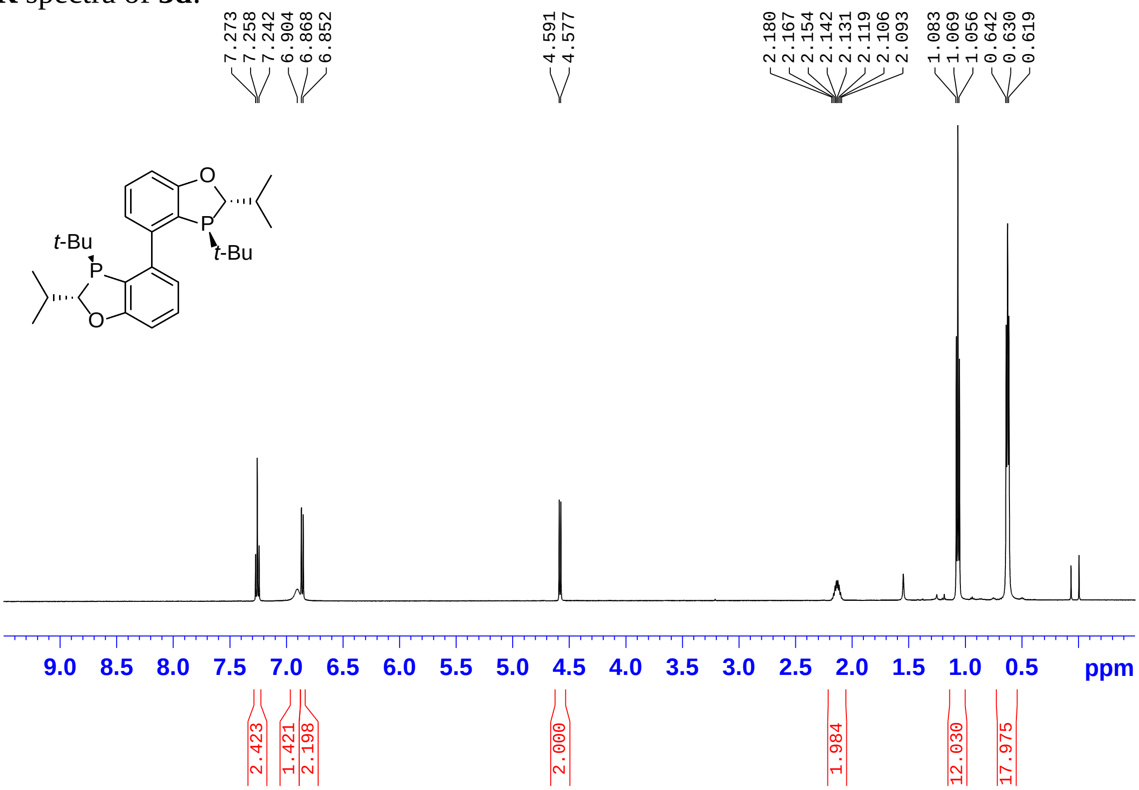

^{31}C **NMR** spectra of **3d**:

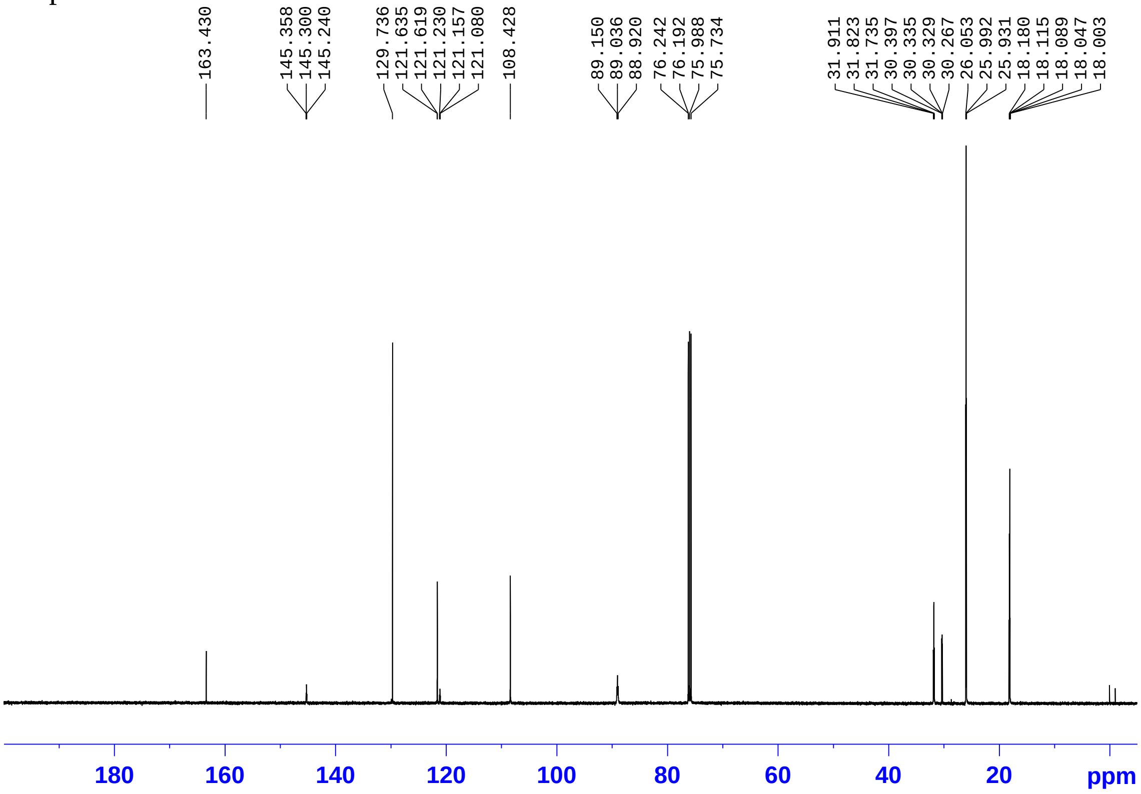

^{31}P **NMR** spectra of **3d**:

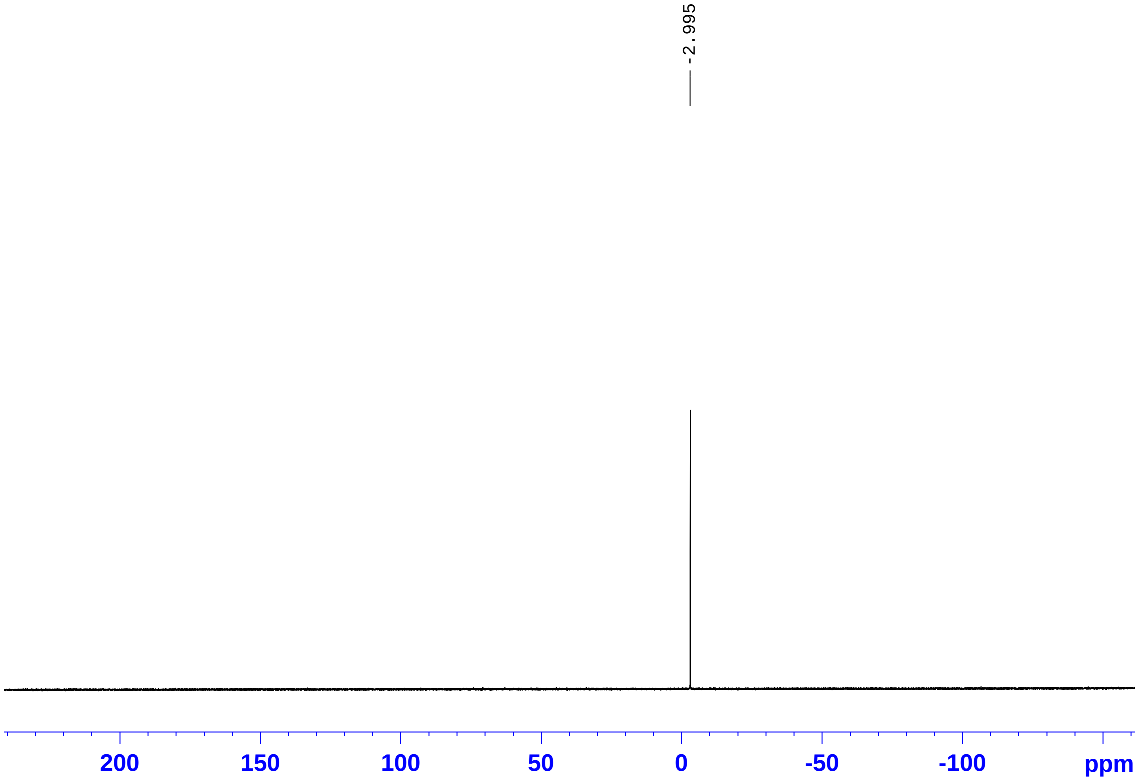
-2.995
200
150
100
50
0
-50
-100
ppm

^{1}**H NMR** spectra of **2e**:

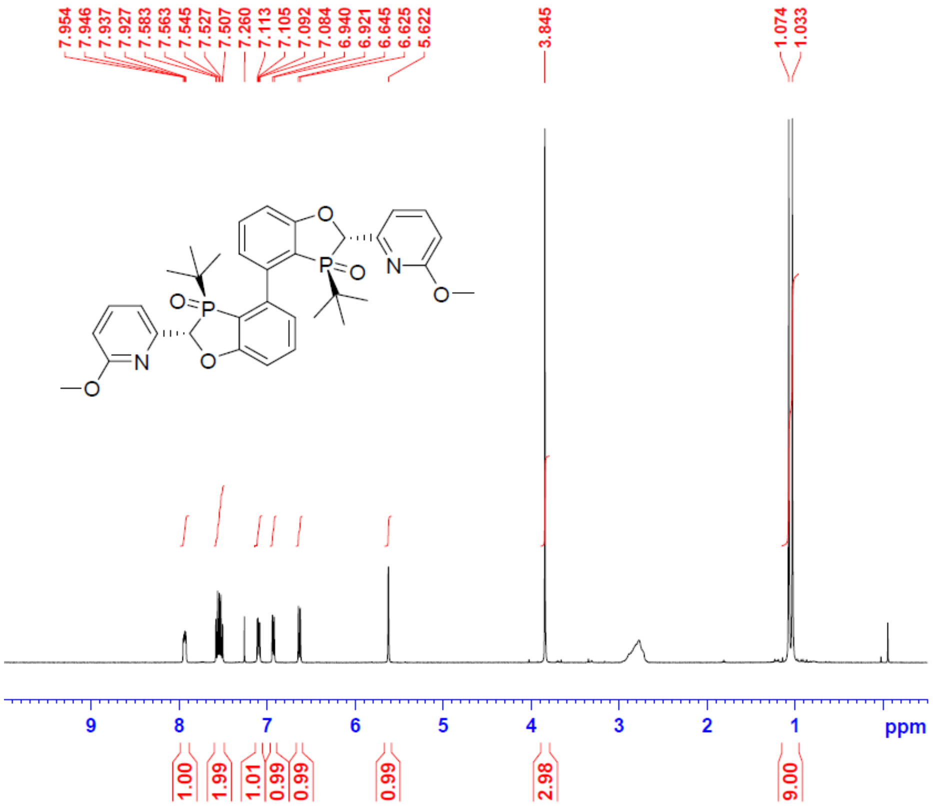

^{31}**C NMR** spectra of **2e**:

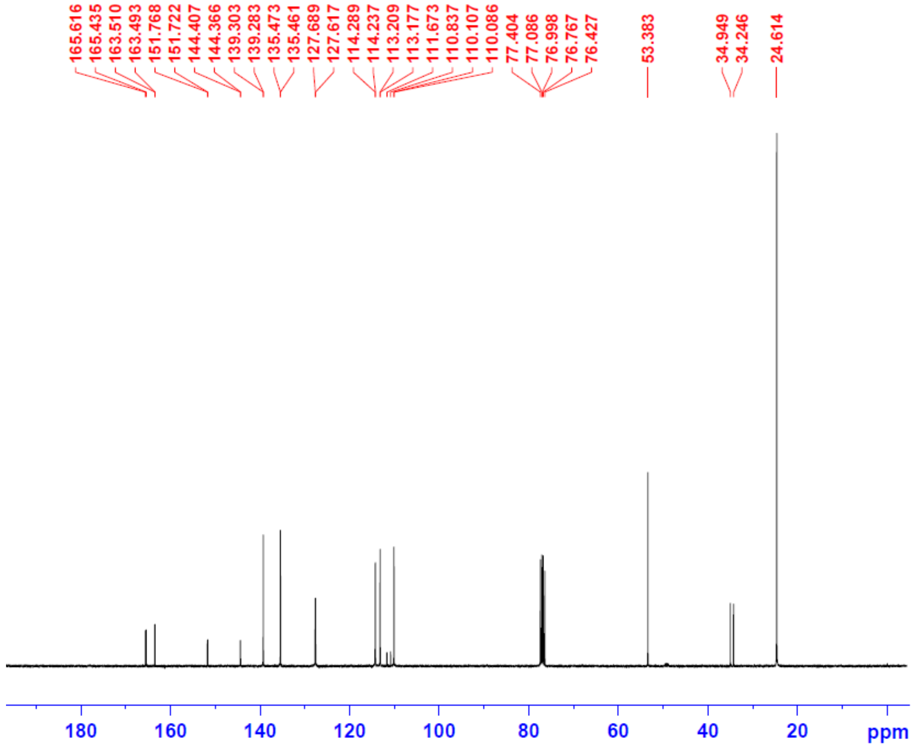

^{31}**P NMR** spectra of **2e**:

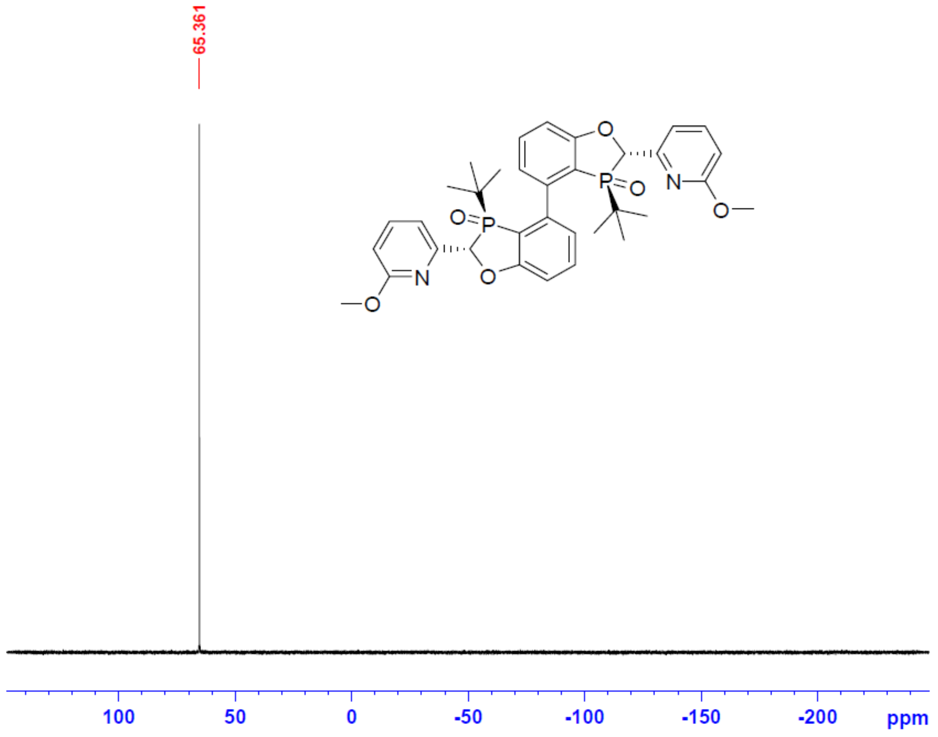

^{1}**H NMR** spectra of **3e**:

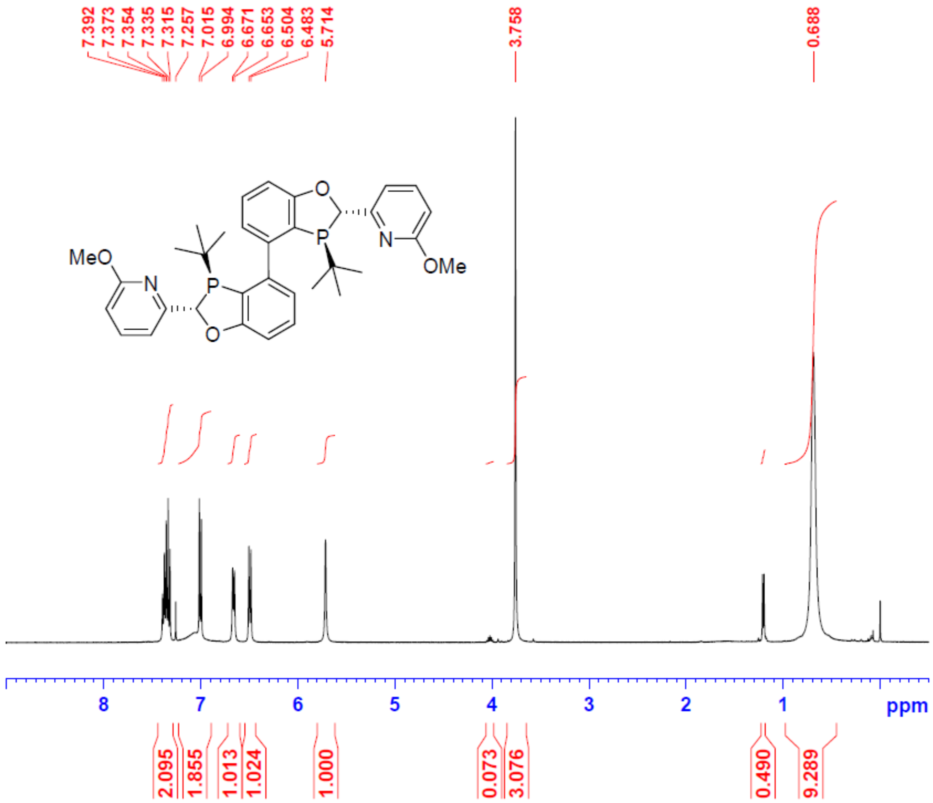

^{31}**C NMR** spectra of **3e**:

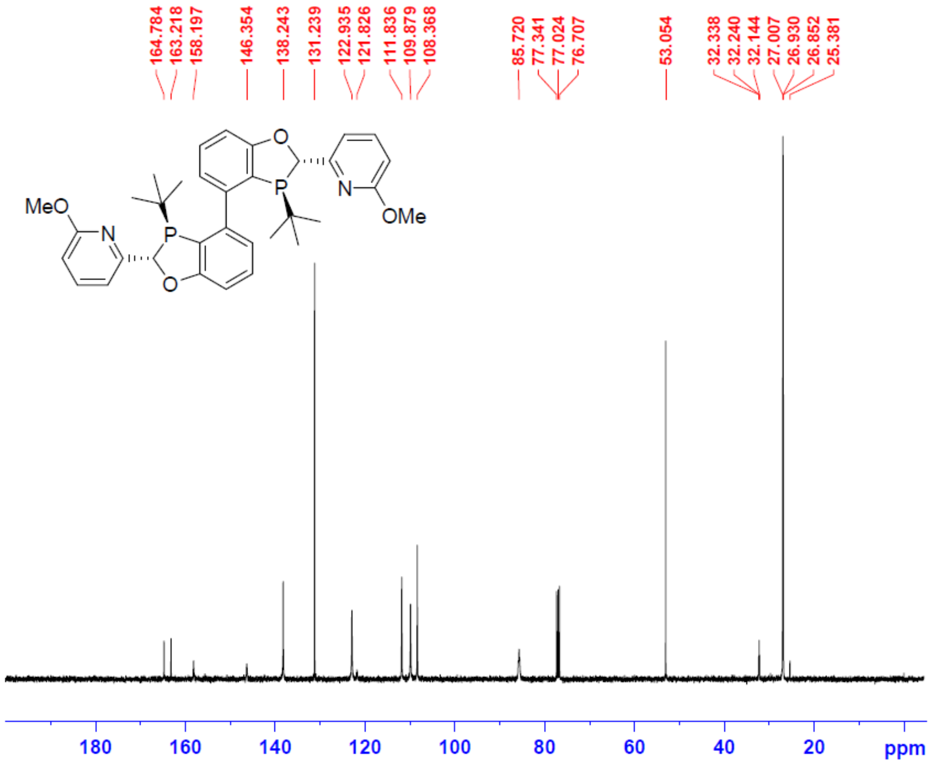

^{31}**P NMR** spectra of **3e**:

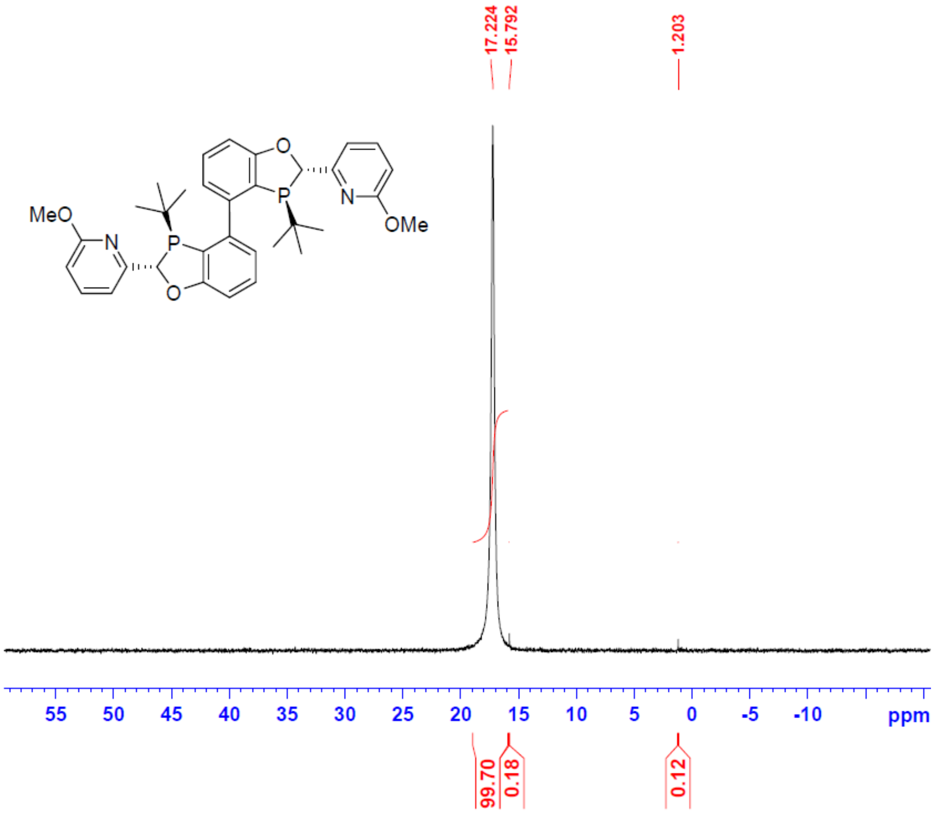
17.224
15.792
1.203
MeO
N
P
O
O
P
N
OMe
55
50
45
40
35
30
25
20
15
10
5
0
-5
-10
ppm
99.70
0.18
0.12

^{1}H NMR spectra of *N*-(3-Hydroxyphenyl)pivalamide:

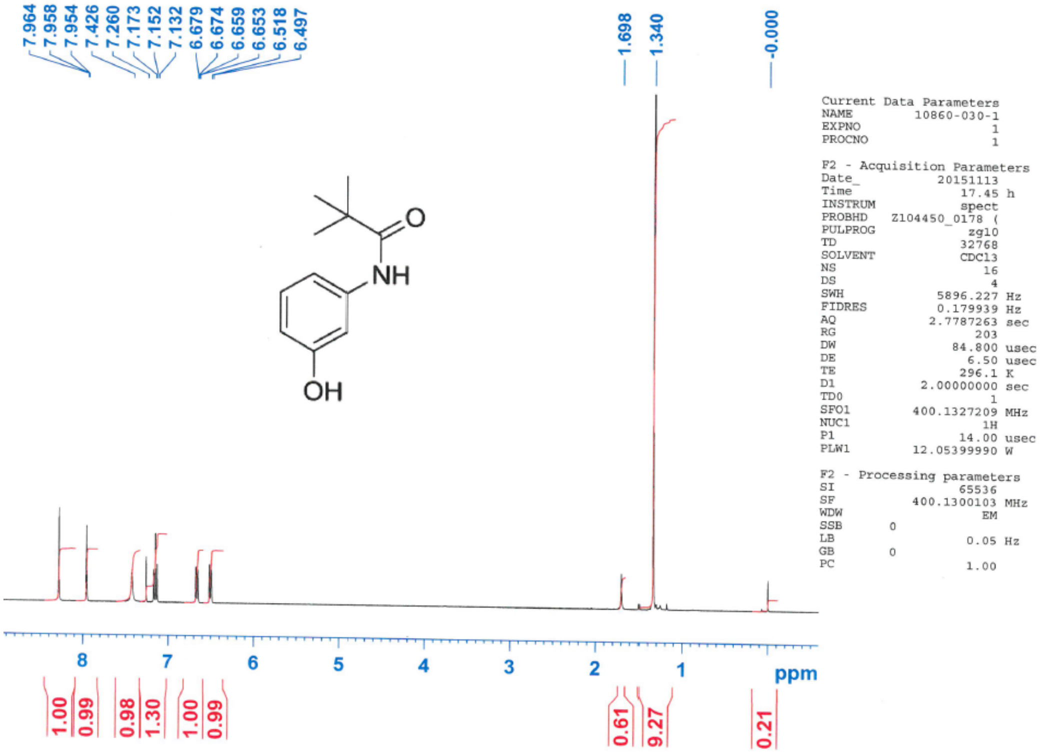

^{13}C NMR spectra of *N*-(3-Hydroxyphenyl)pivalamide:

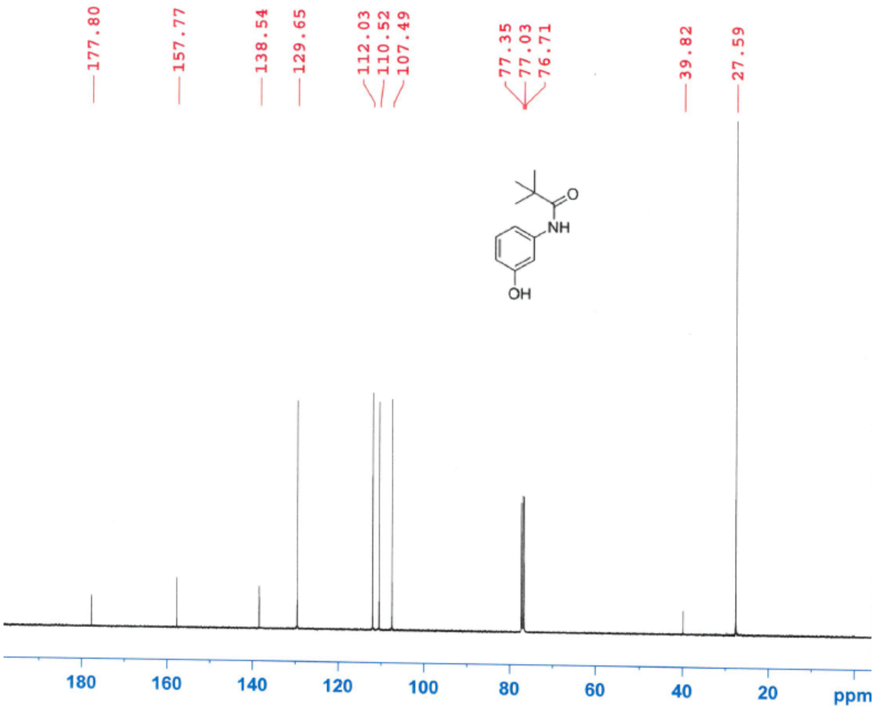

^{1}H NMR spectra of **s4**:

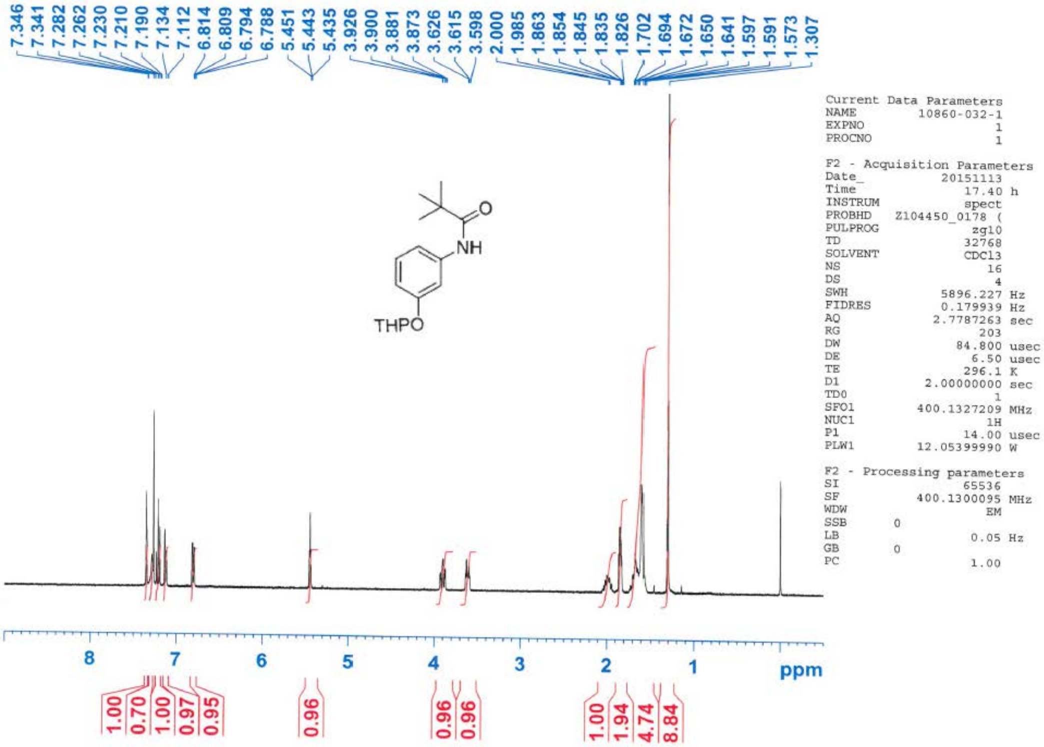

^{13}C NMR spectra of **s4**:

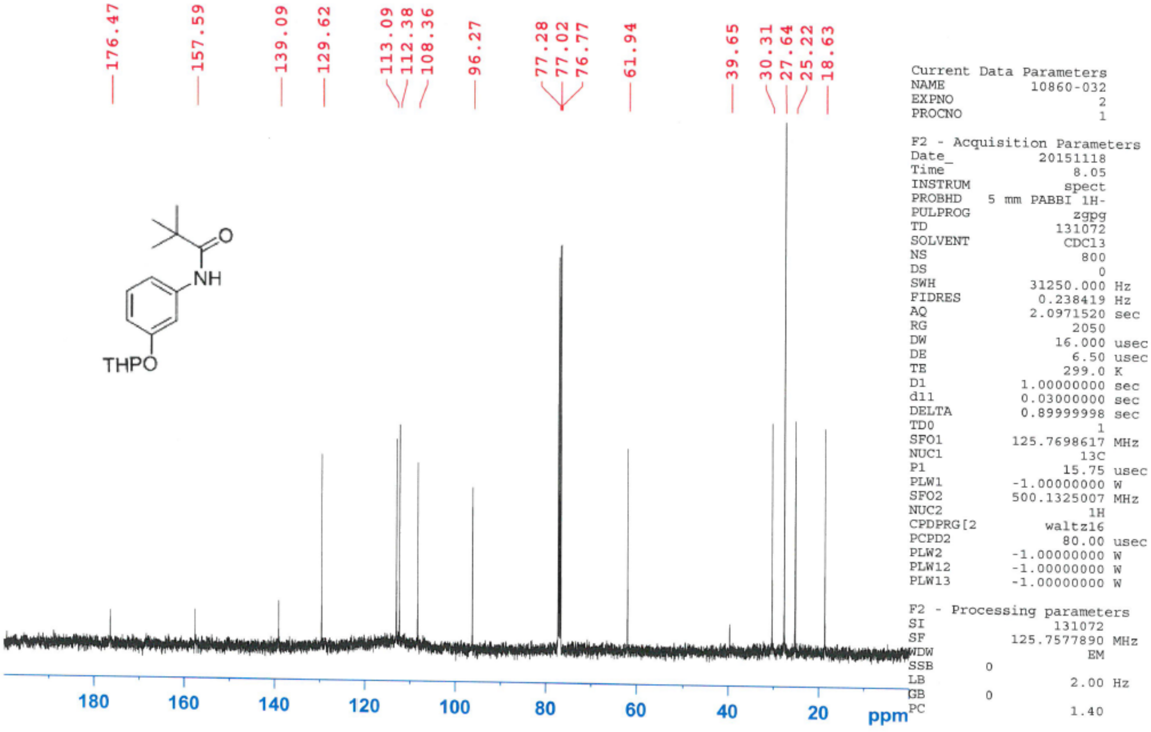

^{1}**H NMR** spectra of **s6**:

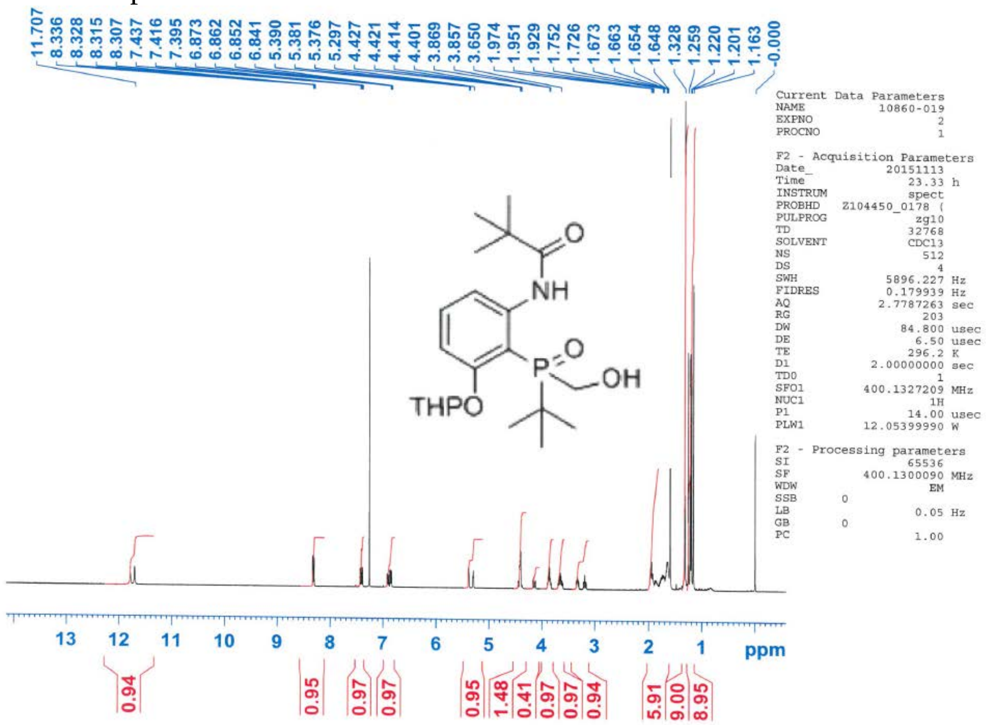

^{13}**C NMR** spectra of **s6**:

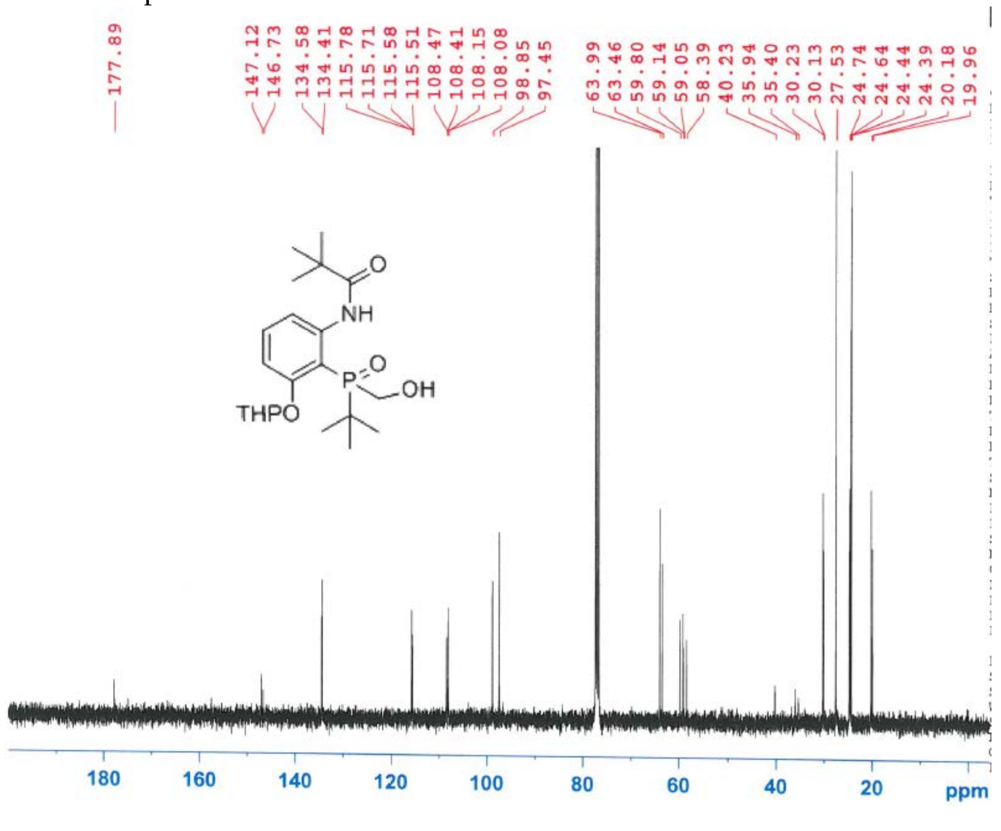

^{31}P NMR spectra of **s6**:

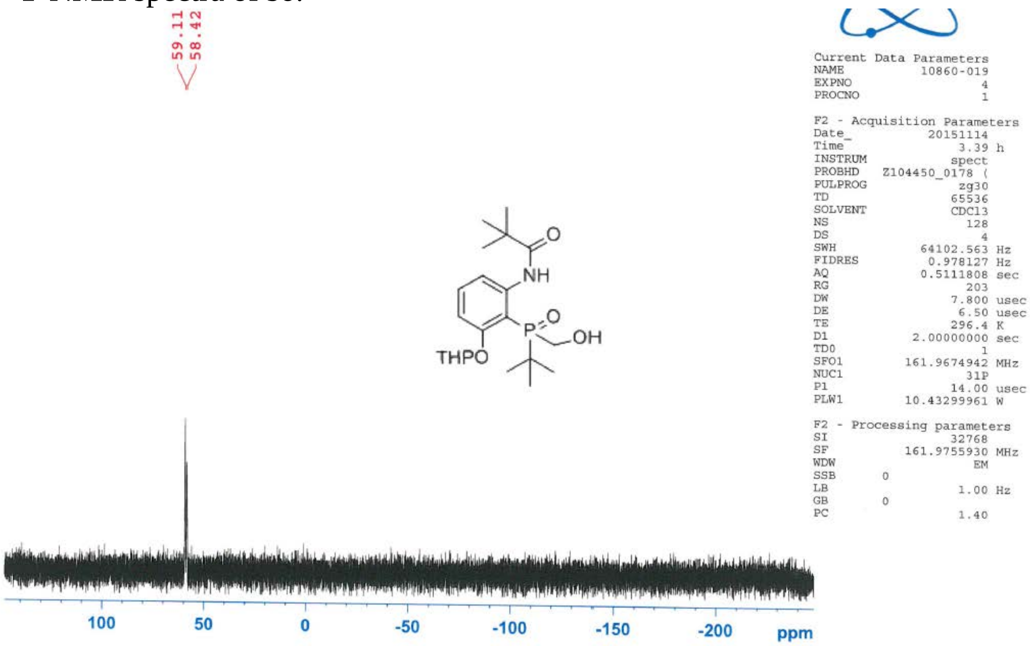

^{1}H NMR spectra of **s7**:

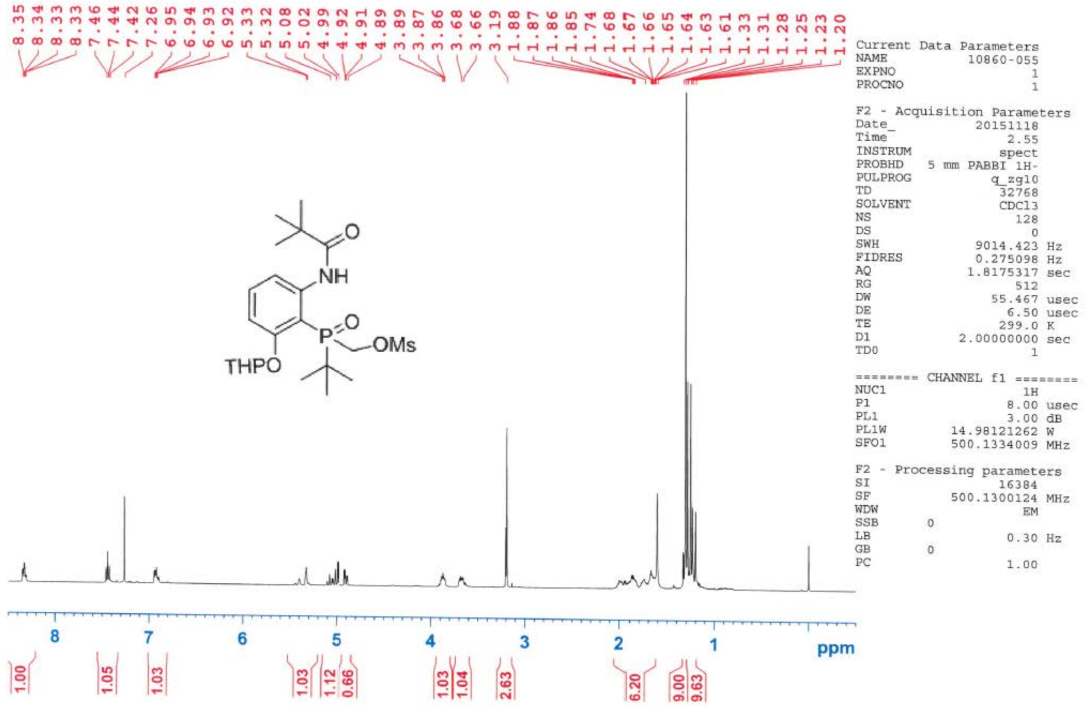

^{13}C NMR spectra of **s7**:

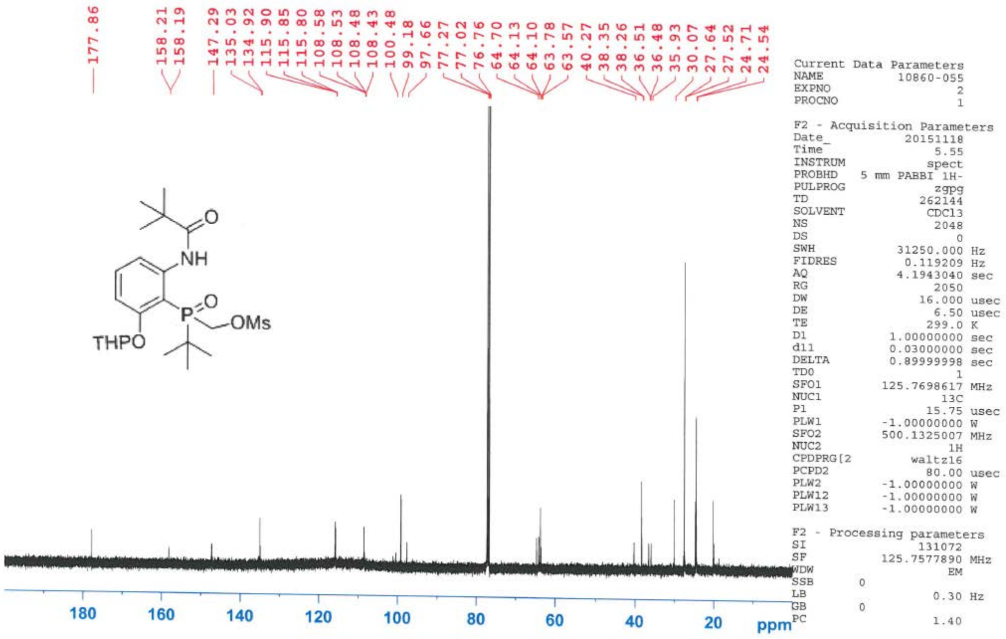

^{31}P NMR spectra of **s7**:

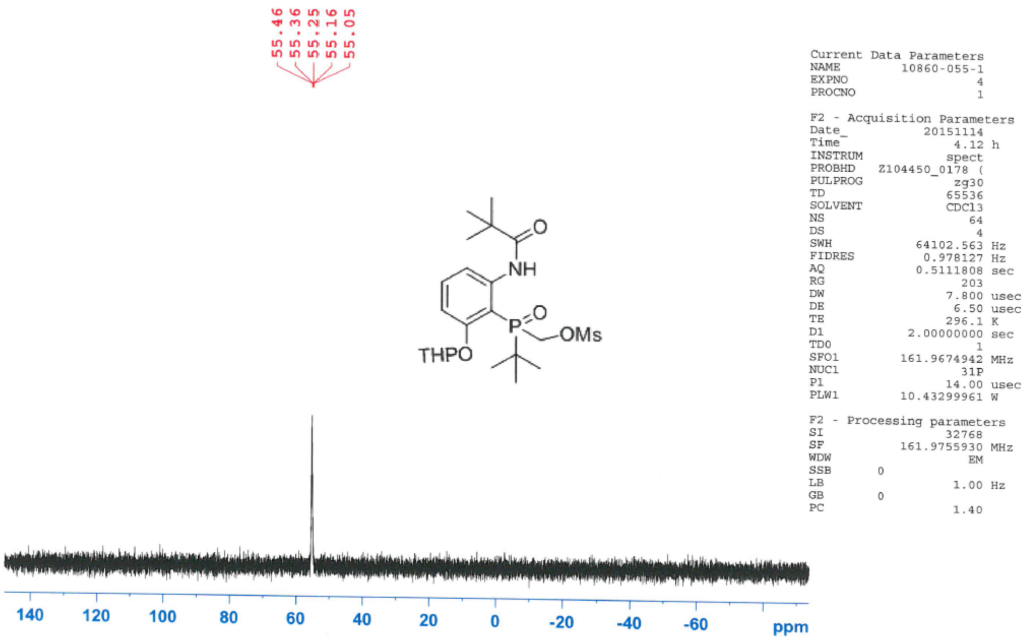

55.46
55.36
55.25
55.16
55.05
Current Data Parameters
NAME 10860-055-1
EXPNO 4
PROCNO 1
F2 - Acquisition Parameters
Date_ 20151114
Time 4.12 h
INSTRUM spect
PROBHD Z104450_0178 (
PULPROG zg30
TD 65536
SOLVENT CDCl3
NS 64
DS 4
SWH 64102.563 Hz
FIDRES 0.978127 Hz
AQ 0.5111808 sec
RG 203
DW 7.800 usec
DE 6.50 usec
TE 296.1 K
D1 2.00000000 sec
TD0 1
SFO1 161.9674942 MHz
NUC1 31P
P1 14.00 usec
PLW1 10.43299961 W
F2 - Processing parameters
SI 32768
SF 161.9755930 MHz
WDW EM
SSB 0
LB 1.00 Hz
GB 0
PC 1.40
NH
O
P
O
OMs
THPO
140
120
100
80
60
40
20
0
-20
-40
-60
ppm

^{1}H NMR spectra of s8:

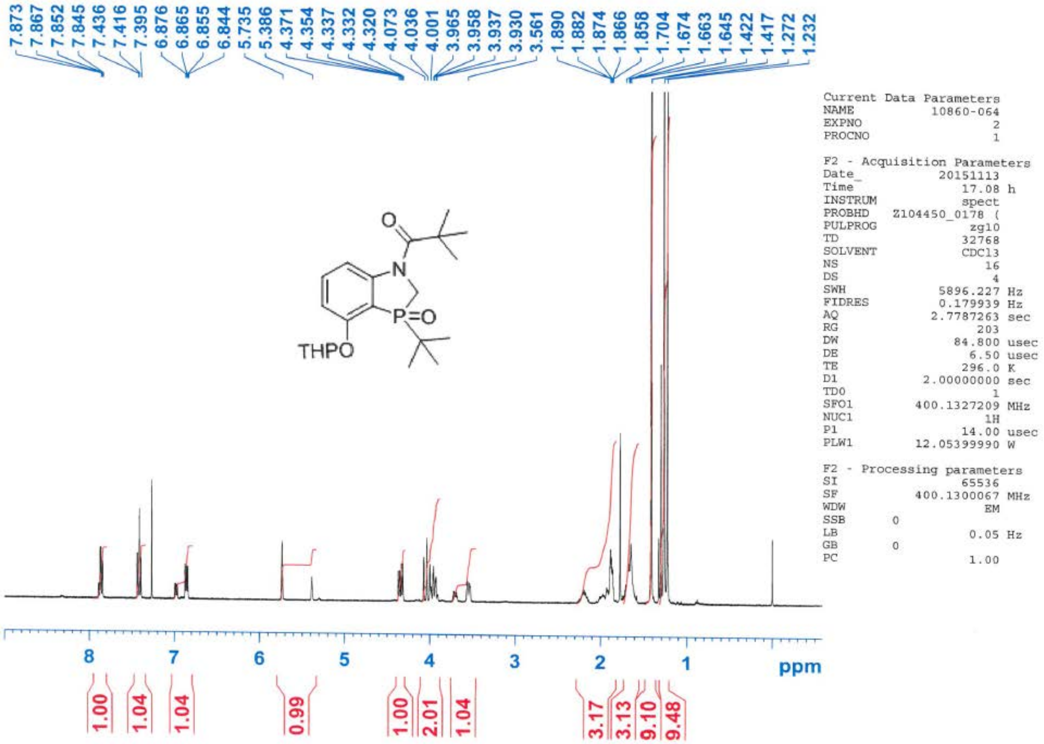

^{13}C NMR spectra of s8:

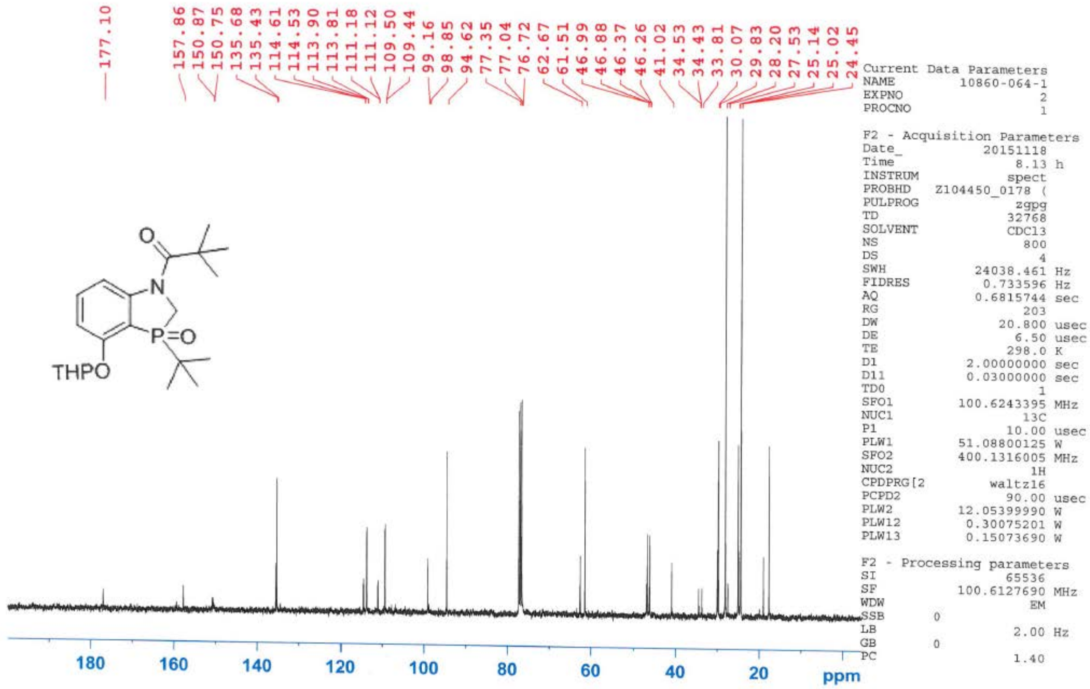

^{31}P NMR spectra of s8:

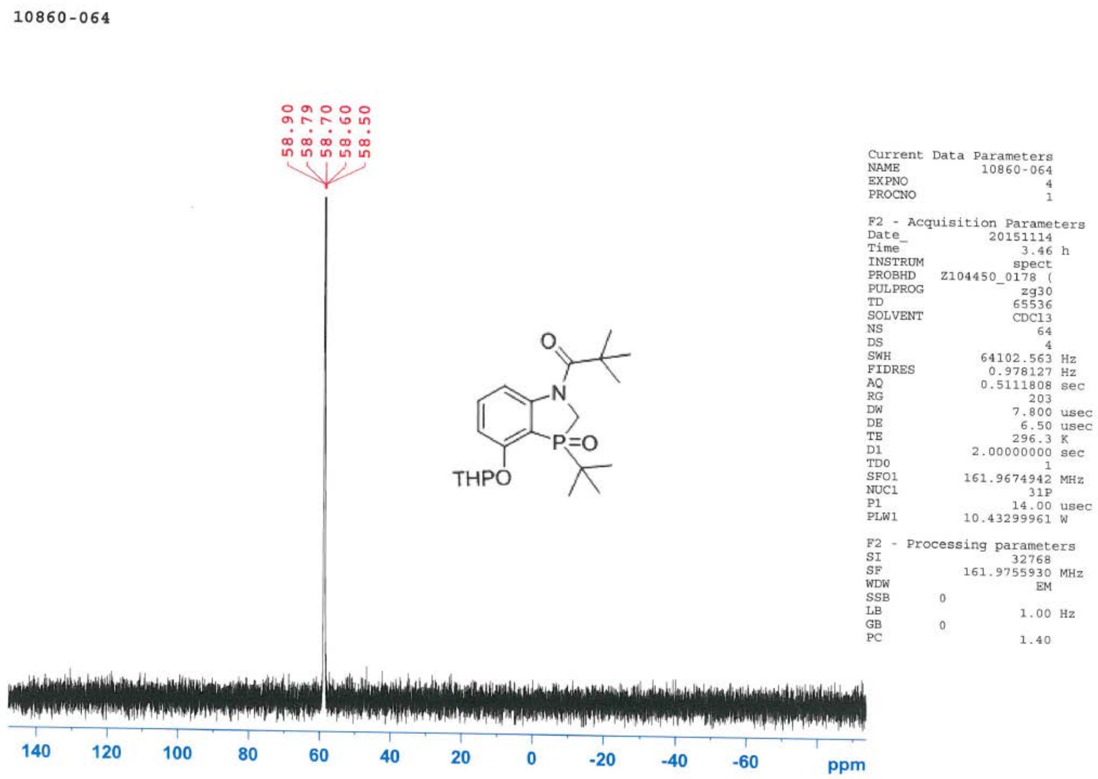
10860-064
58.90
58.79
58.70
58.60
58.50
THPO
N
O
P=O
Current Data Parameters
NAME 10860-064
EXPNO 4
PROCNO 1
F2 - Acquisition Parameters
Date_ 20151114
Time 3.46 h
INSTRUM spect
PROBHD Z104450_0178 (
PULPROG zg30
TD 65536
SOLVENT CDCl3
NS 64
DS 4
SWH 64102.563 Hz
FIDRES 0.978127 Hz
AQ 0.5111808 sec
RG 203
DW 7.800 usec
DE 6.50 usec
TE 296.3 K
D1 2.00000000 sec
TD0 1
SFO1 161.9674942 MHz
NUC1 31P
P1 14.00 usec
PLW1 10.43299961 W
F2 - Processing parameters
SI 32768
SF 161.9755930 MHz
WDW EM
SSB 0
LB 1.00 Hz
GB 0
PC 1.40
140
120
100
80
60
40
20
0
-20
-40
-60
ppm

[1]**H NMR** spectra of **s9**:

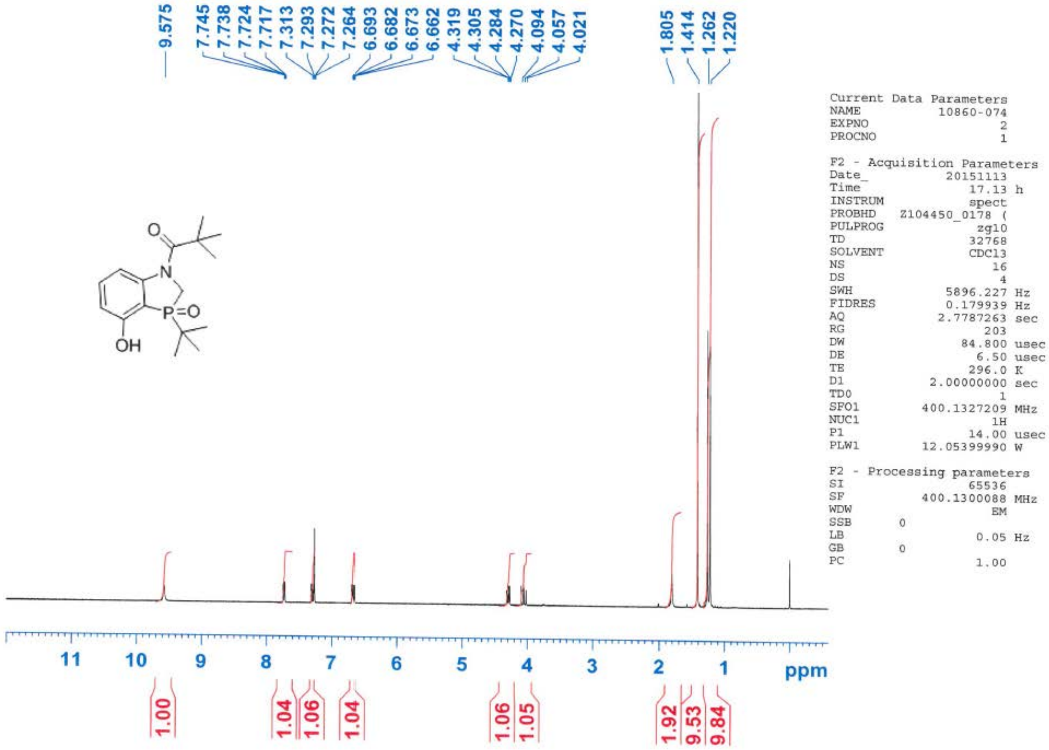

[13]**C NMR** spectra of **s9**:

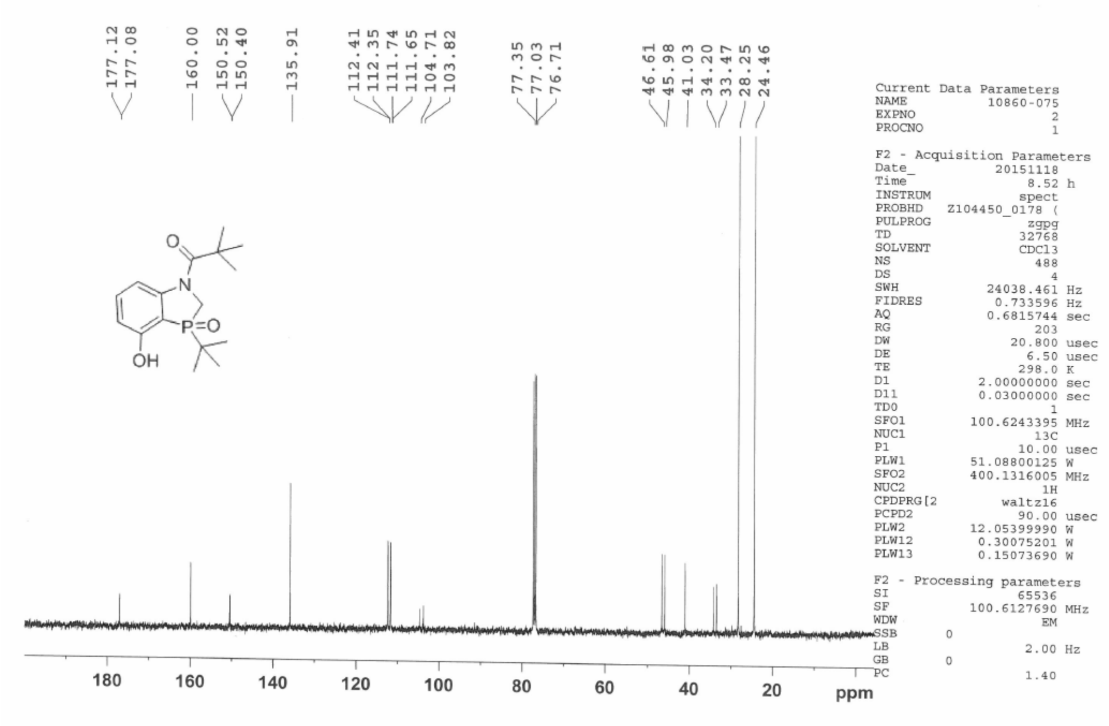

[31]**P NMR** spectra of **s9**:

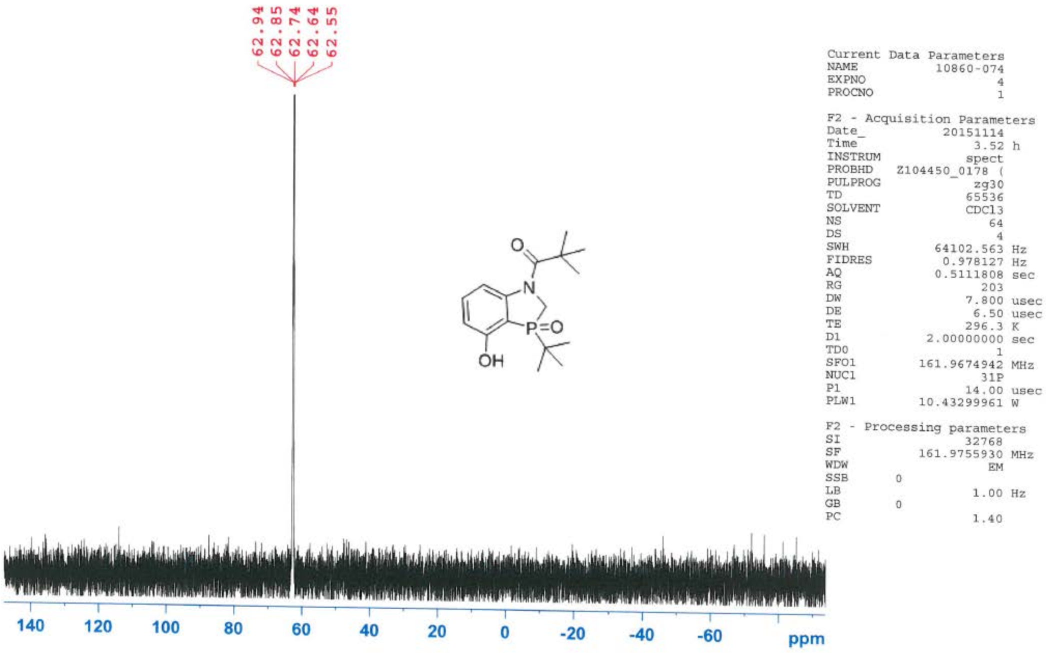

Chiral SFC chromatogram of racemic **s9:**

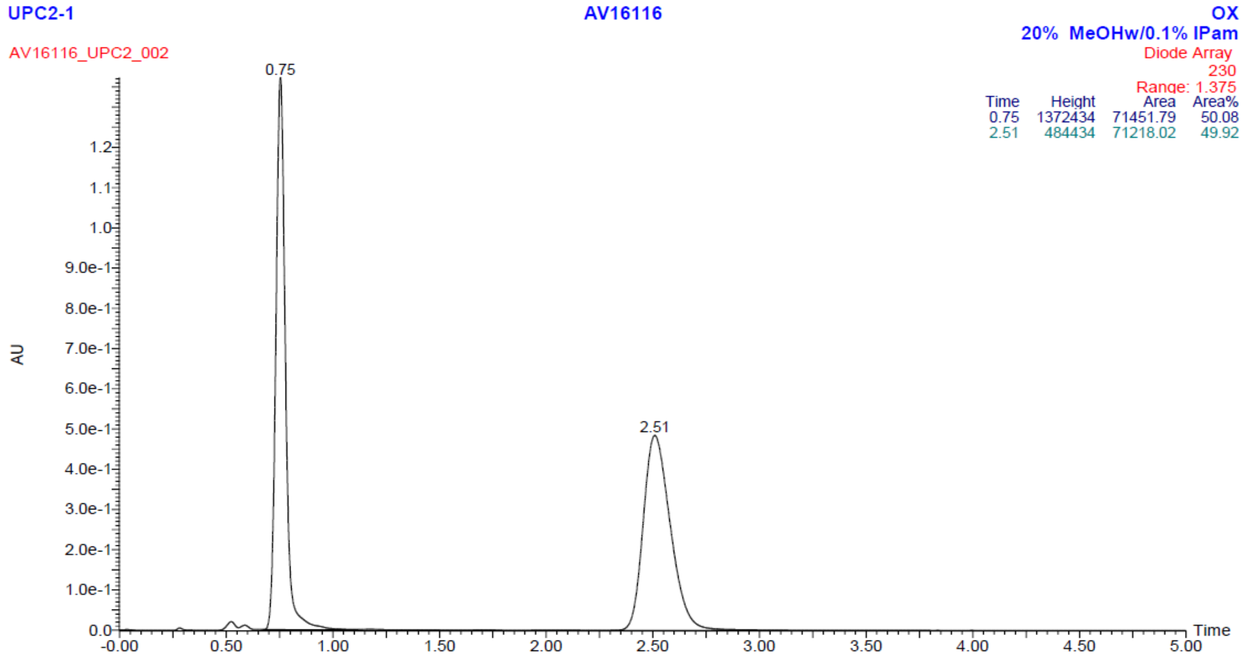

Chiral SFC chromatogram of racemic **(*S*)-s9**:

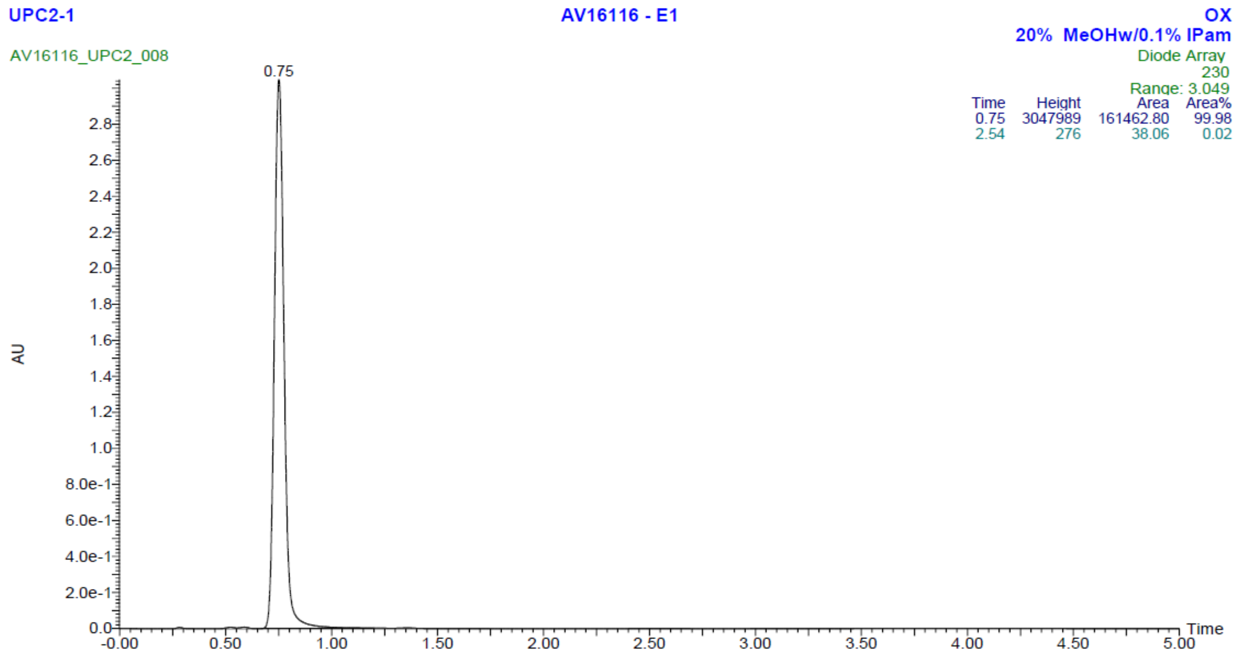

Chiral SFC chromatogram of racemic ***(R)*-s9**:

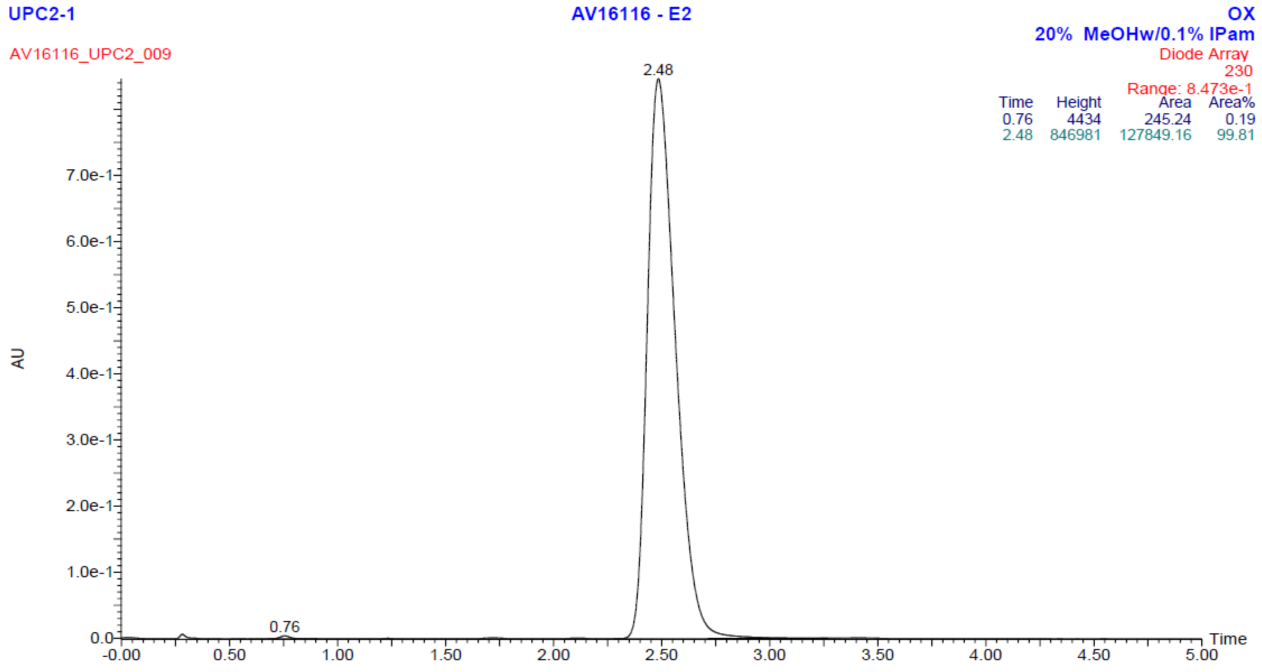

^{1}H **NMR** spectra of **1b**:

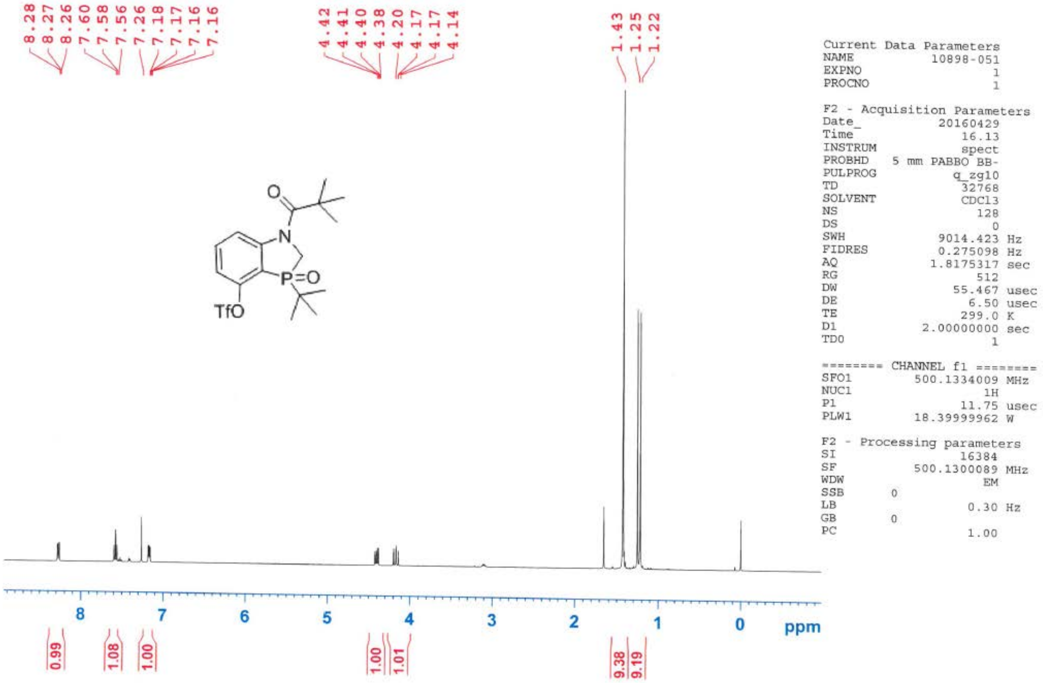

^{13}C **NMR** spectra of **1b**:

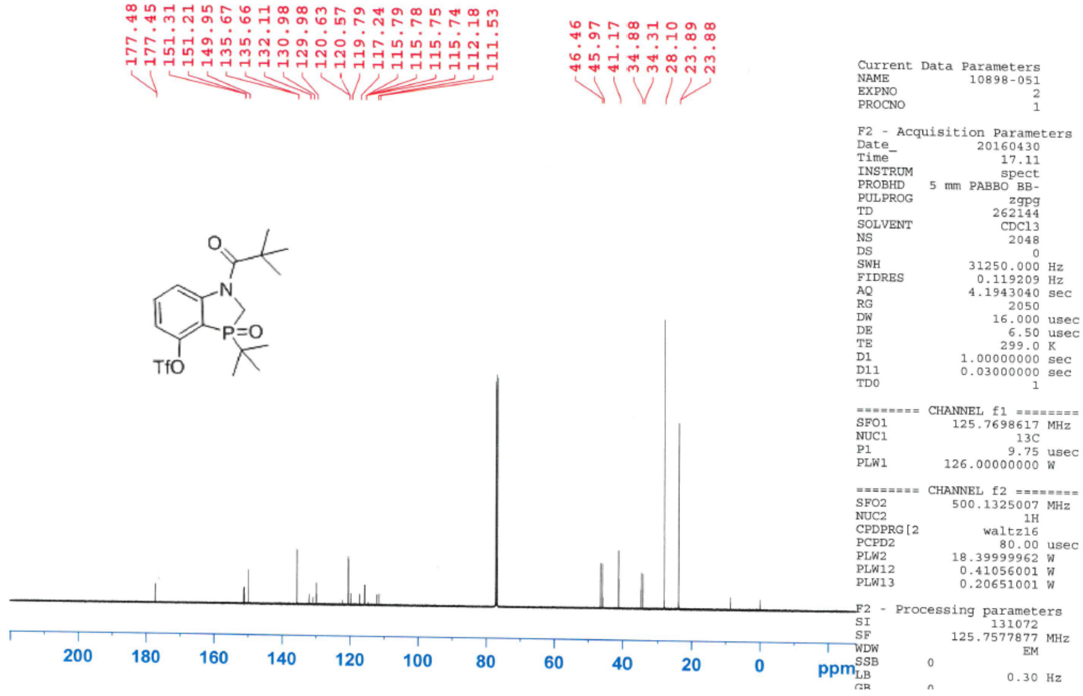

^{31}P **NMR** spectra of **1b**:

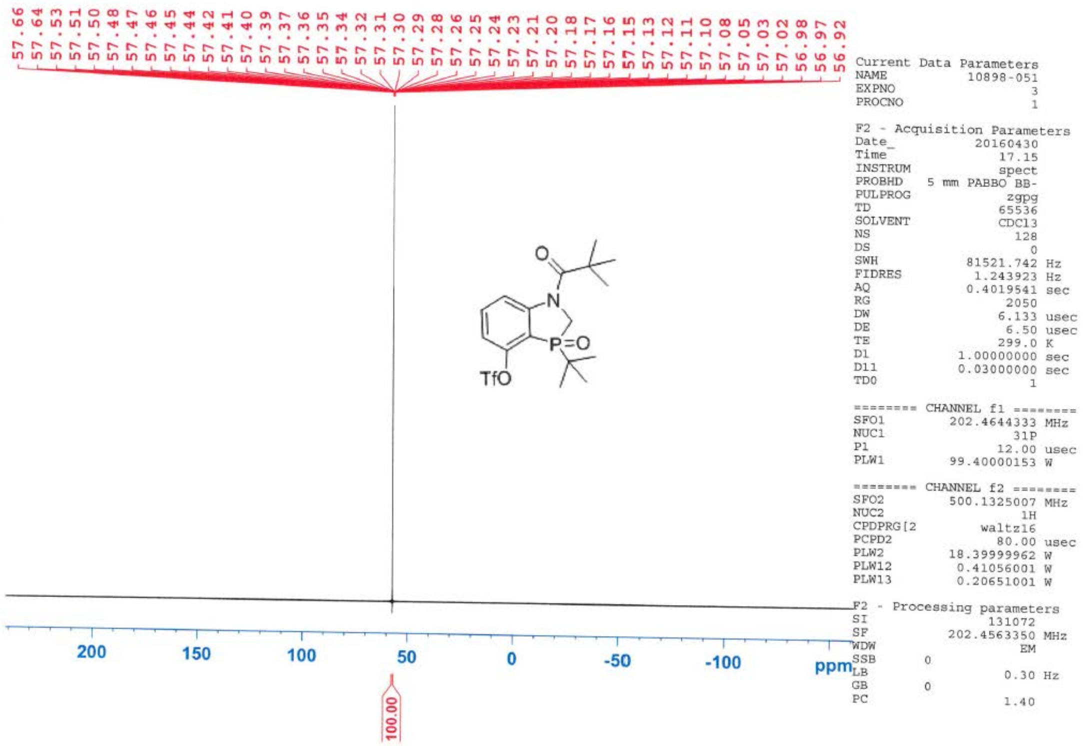
57.66 57.64 57.53 57.51 57.50 57.48 57.47 57.46 57.45 57.44 57.42 57.41 57.40 57.39 57.37 57.36 57.35 57.34 57.32 57.31 57.30 57.29 57.28 57.26 57.25 57.24 57.23 57.21 57.20 57.18 57.17 57.16 57.15 57.13 57.12 57.11 57.10 57.08 57.05 57.03 57.02 56.98 56.97 56.92
TfO
Current Data Parameters
NAME 10898-051
EXPNO 3
PROCNO 1
F2 - Acquisition Parameters
Date_ 20160430
Time 17.15
INSTRUM spect
PROBHD 5 mm PABBO BB-
PULPROG zgpg
TD 65536
SOLVENT CDCl3
NS 128
DS 0
SWH 81521.742 Hz
FIDRES 1.243923 Hz
AQ 0.4019541 sec
RG 2050
DW 6.133 usec
DE 6.50 usec
TE 299.0 K
D1 1.00000000 sec
D11 0.03000000 sec
TD0 1
======== CHANNEL f1 ========
SFO1 202.4644333 MHz
NUC1 31P
P1 12.00 usec
PLW1 99.40000153 W
======== CHANNEL f2 ========
SFO2 500.1325007 MHz
NUC2 1H
CPDPRG[2 waltz16
PCPD2 80.00 usec
PLW2 18.39999962 W
PLW12 0.41056001 W
PLW13 0.20651001 W
F2 - Processing parameters
SI 131072
SF 202.4563350 MHz
WDW EM
SSB 0
LB 0.30 Hz
GB 0
PC 1.40
200 150 100 50 0 -50 -100 ppm
100.00

^{1}H NMR spectra of **2b**:

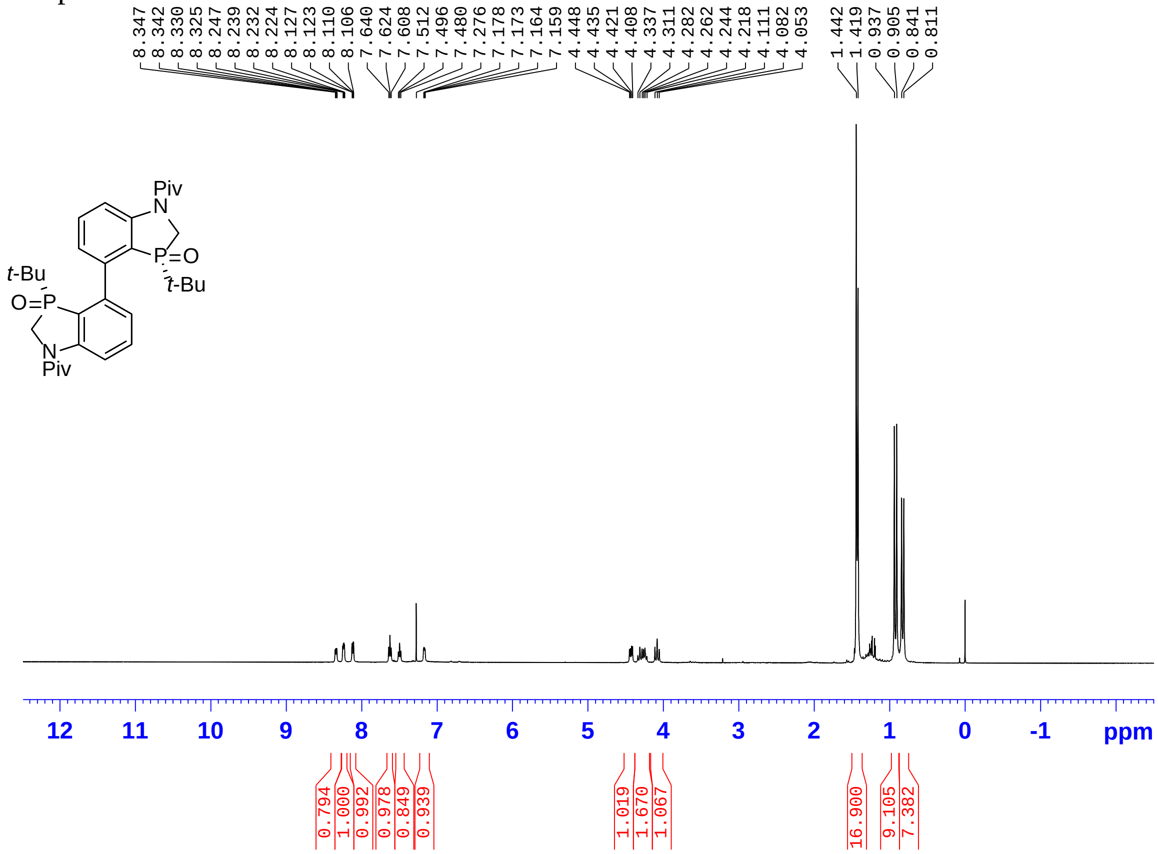

^{31}C NMR spectra of **2b**:

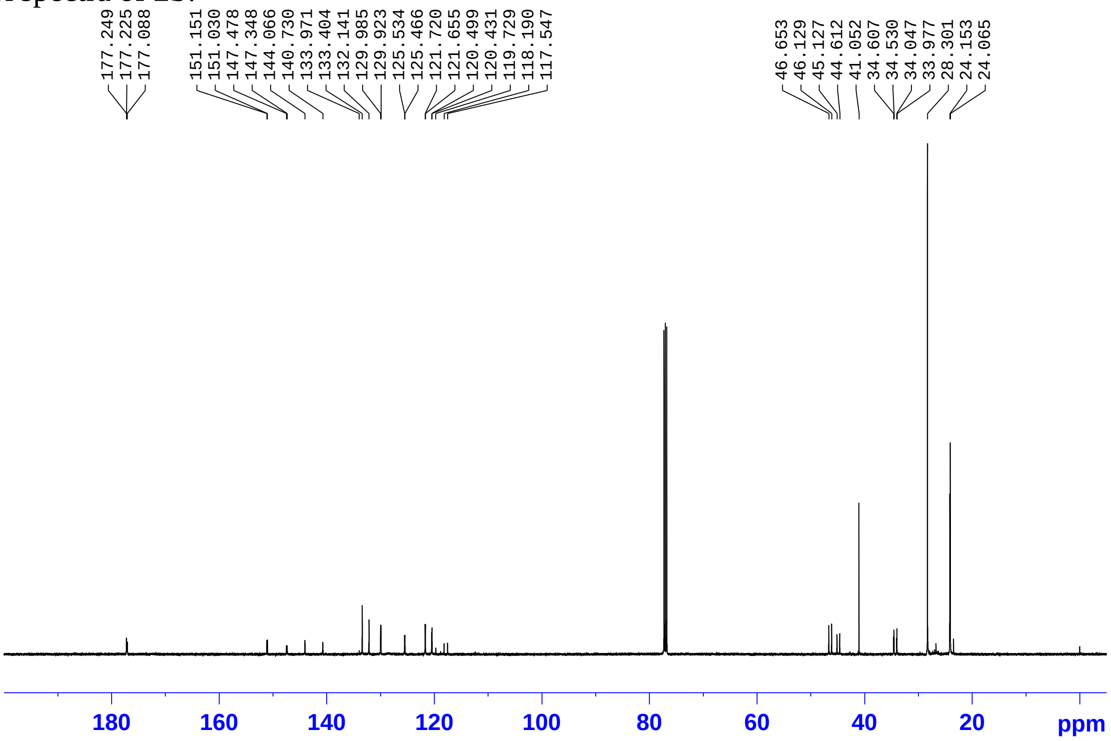

^{31}P NMR spectra of **2b**:

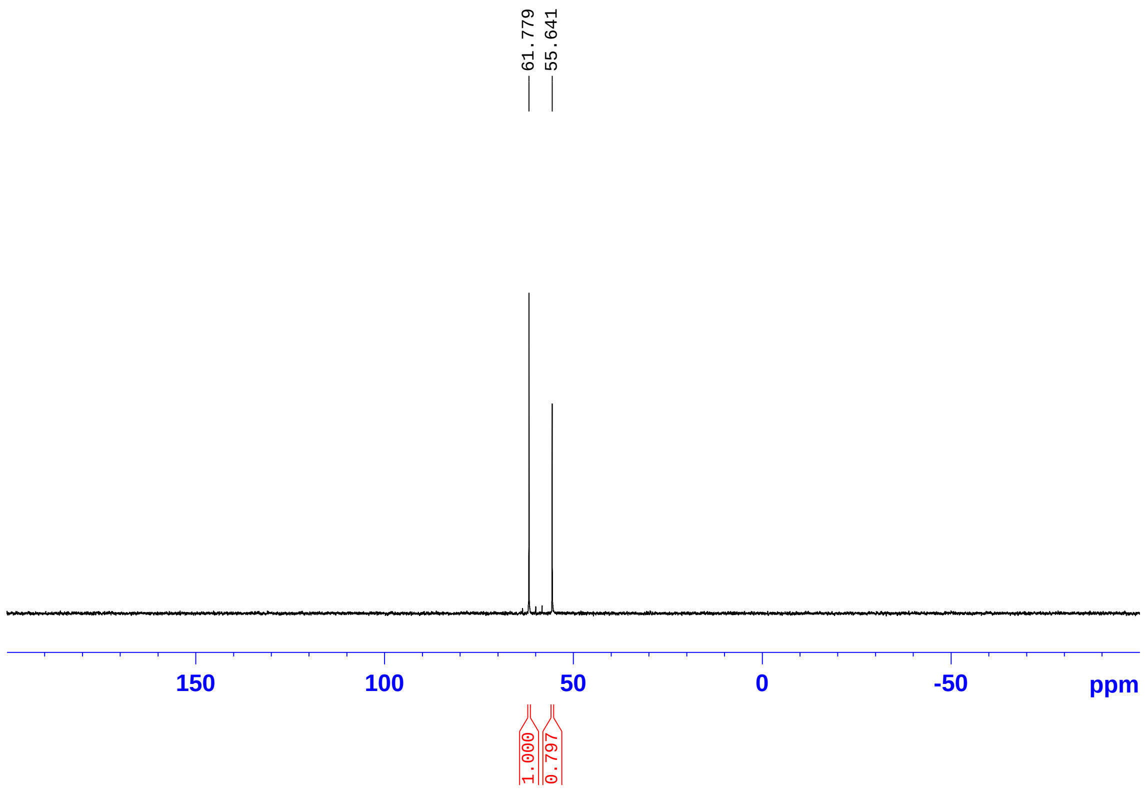
61.779
55.641
150
100
50
0
-50
ppm
1.000
0.797

^{1}H NMR spectra of **3b**:

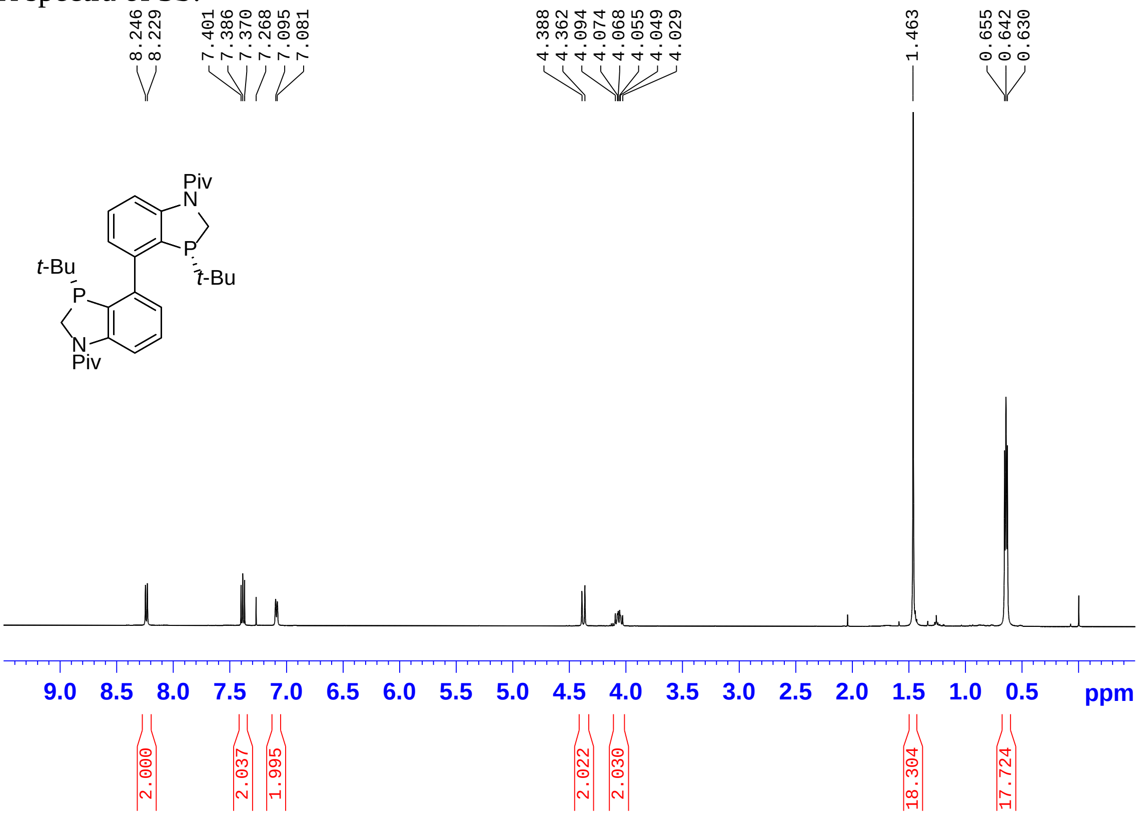

^{13}C NMR spectra of **3b**:

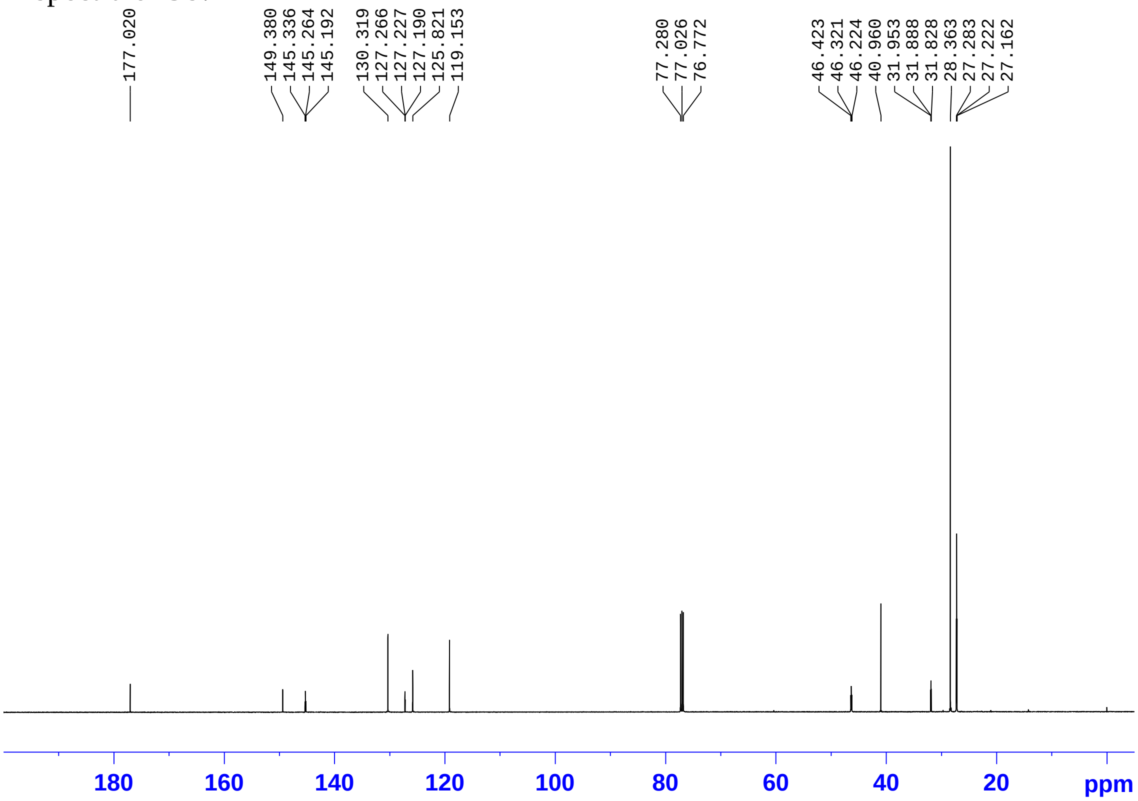

^{31}P NMR spectra of **3b**:

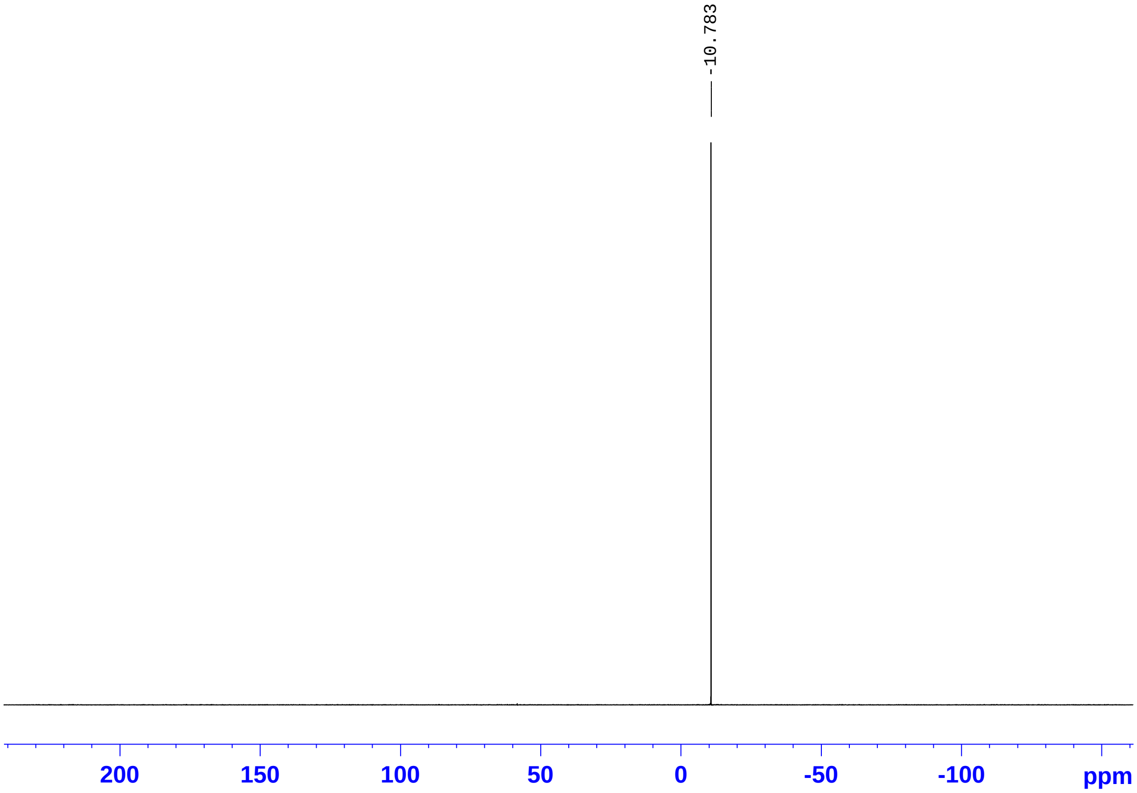

-10.783
200
150
100
50
0
-50
-100
ppm

^{1}H NMR spectra of **4a**:

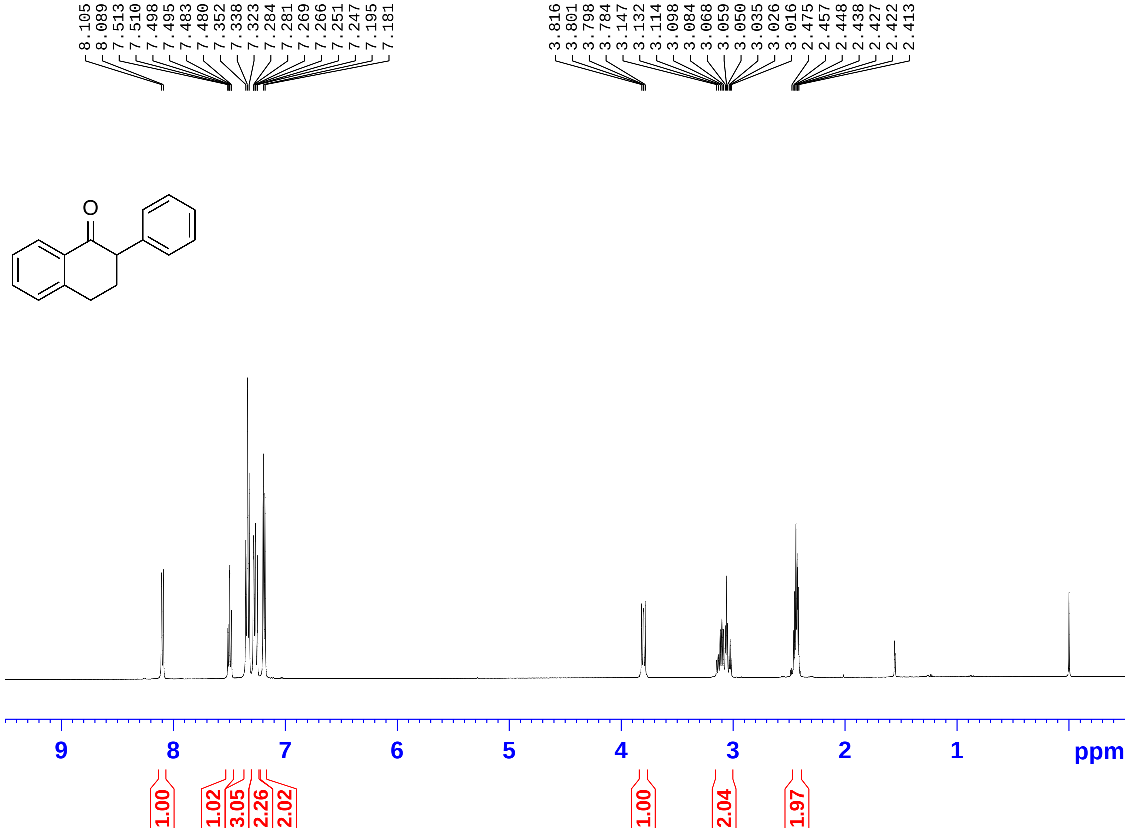

^{1}H NMR spectra of **4b**:

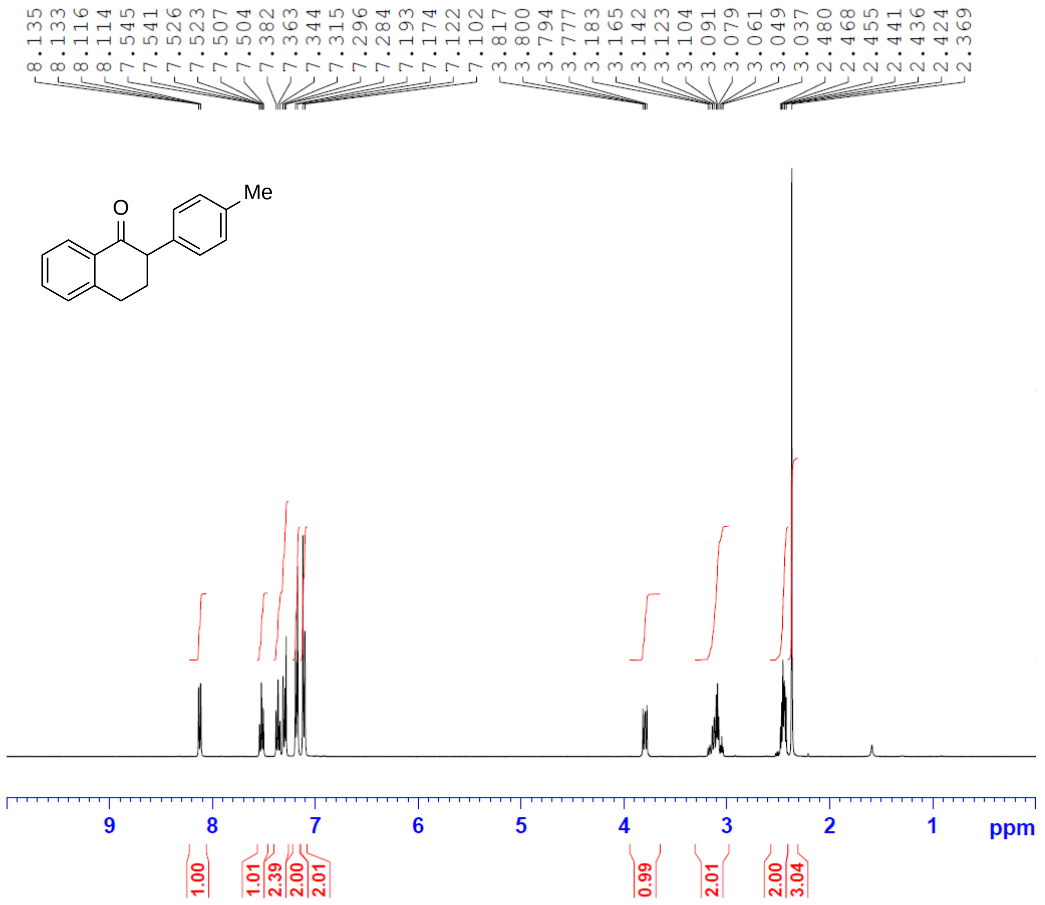

^{1}H NMR spectra of **4c**:

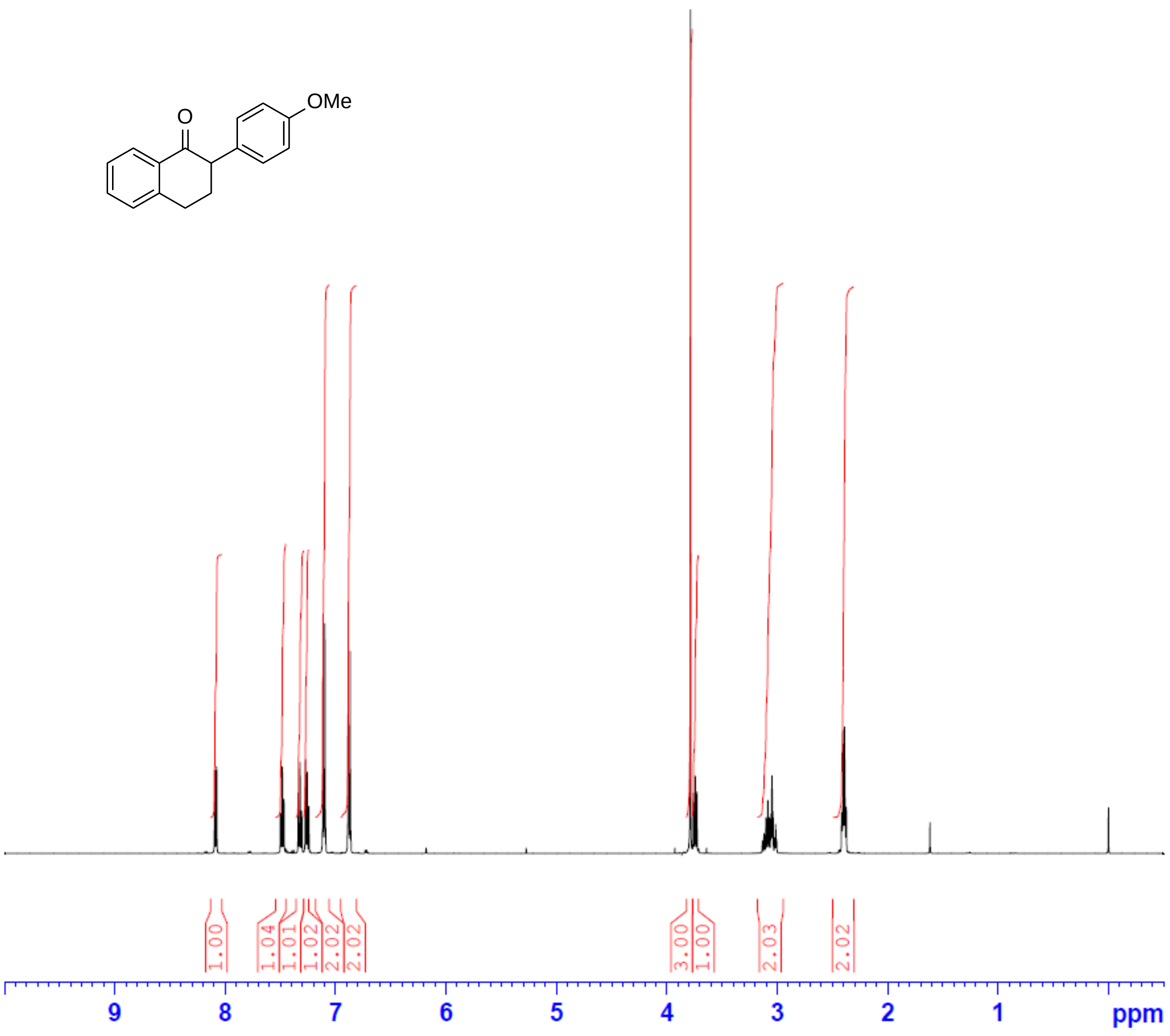

^{1}**H NMR** spectra of **4d**:

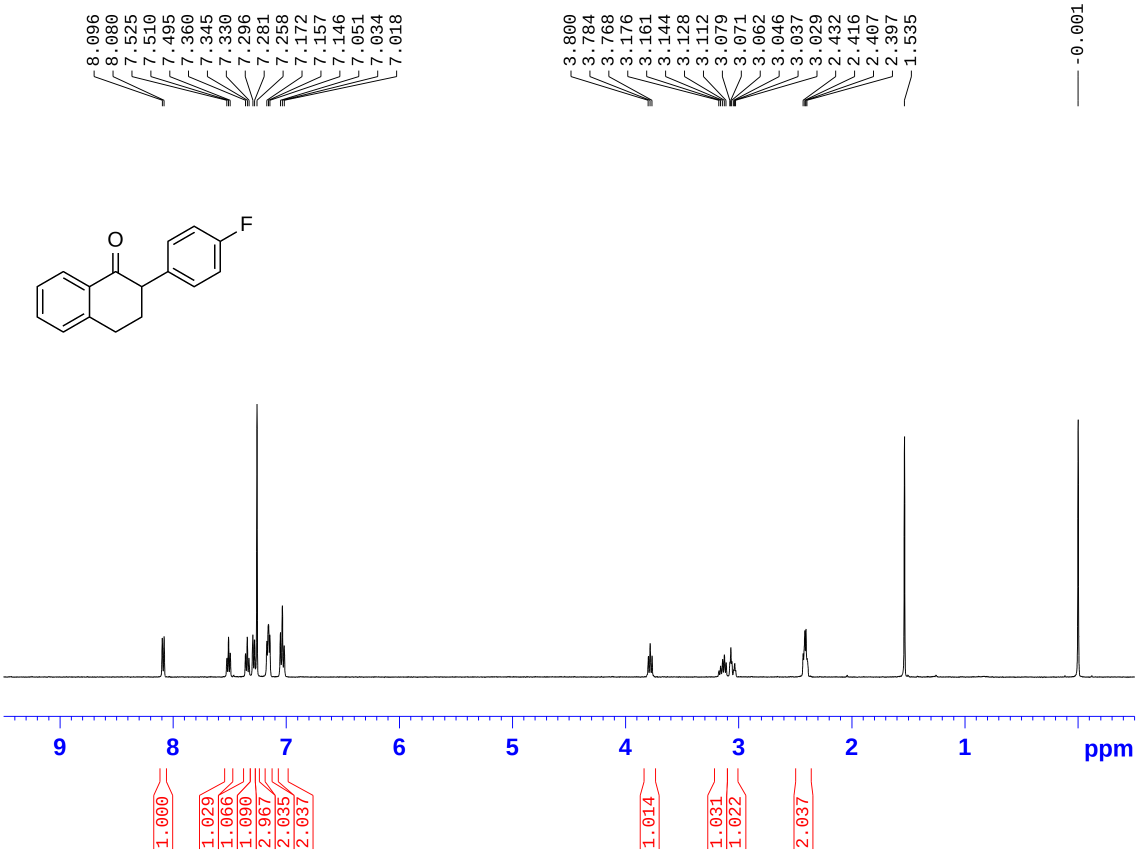

^{1}H NMR spectra of **4e**:

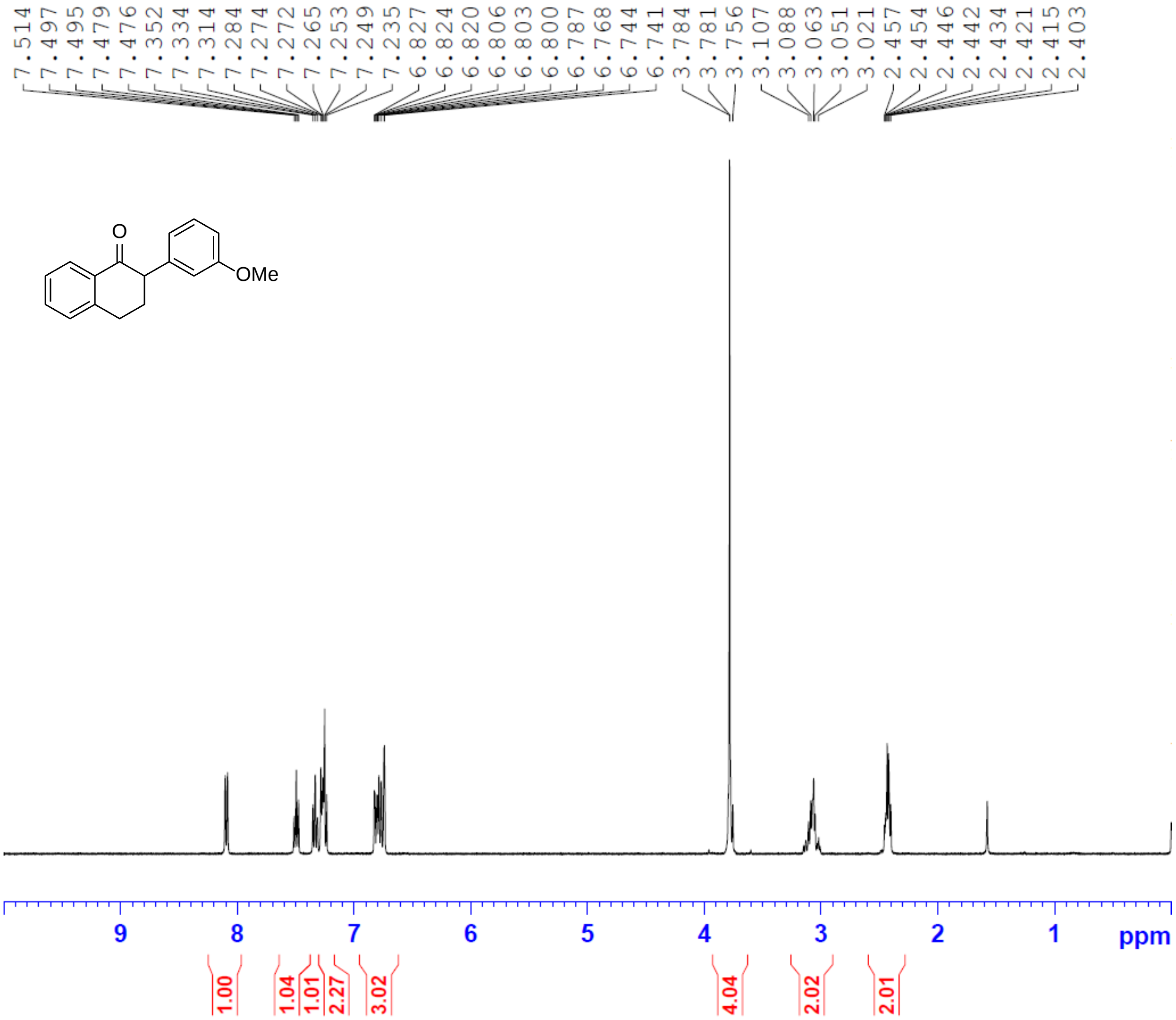

^{1}H NMR spectra of **4f**:

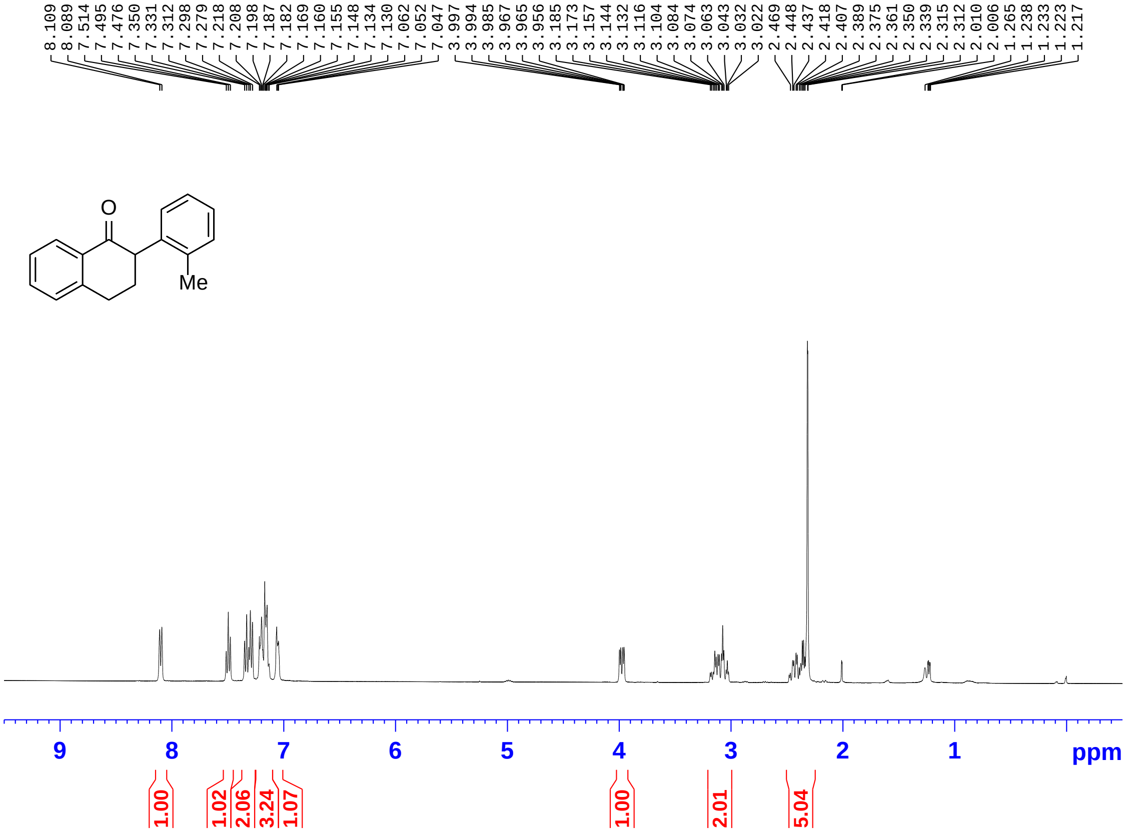

^{1}**H NMR** spectra of **4g**:

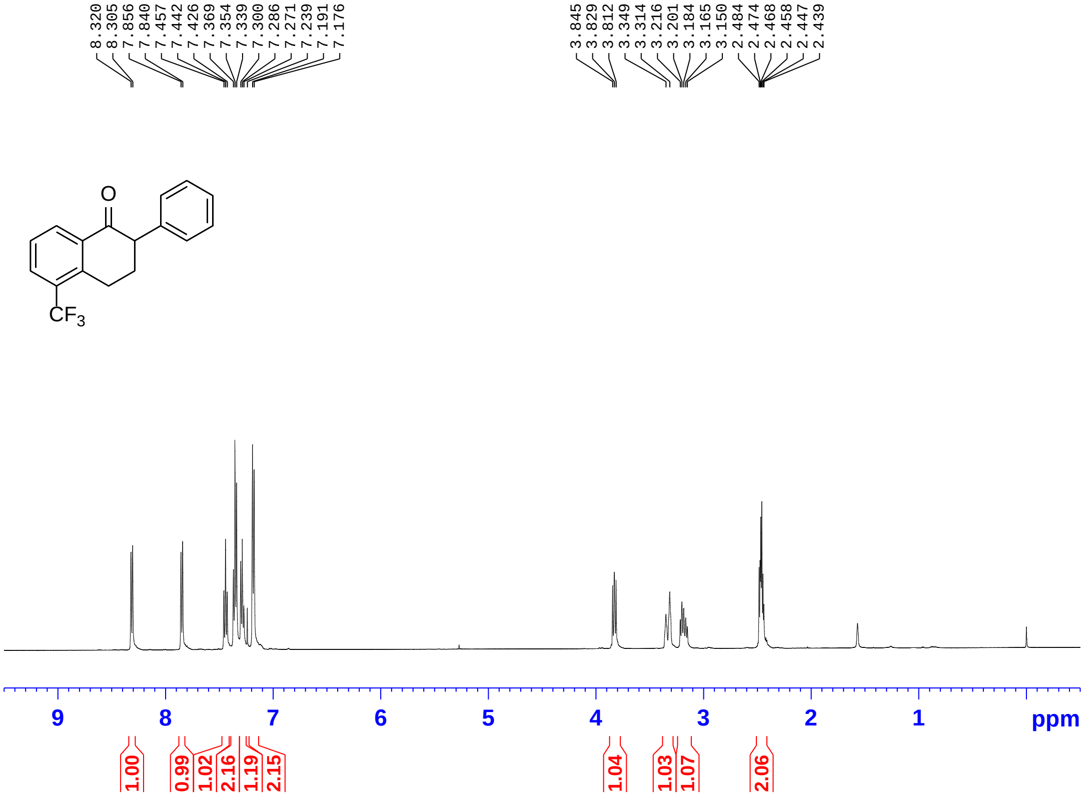

^{13}**C NMR** spectra of **4g**:

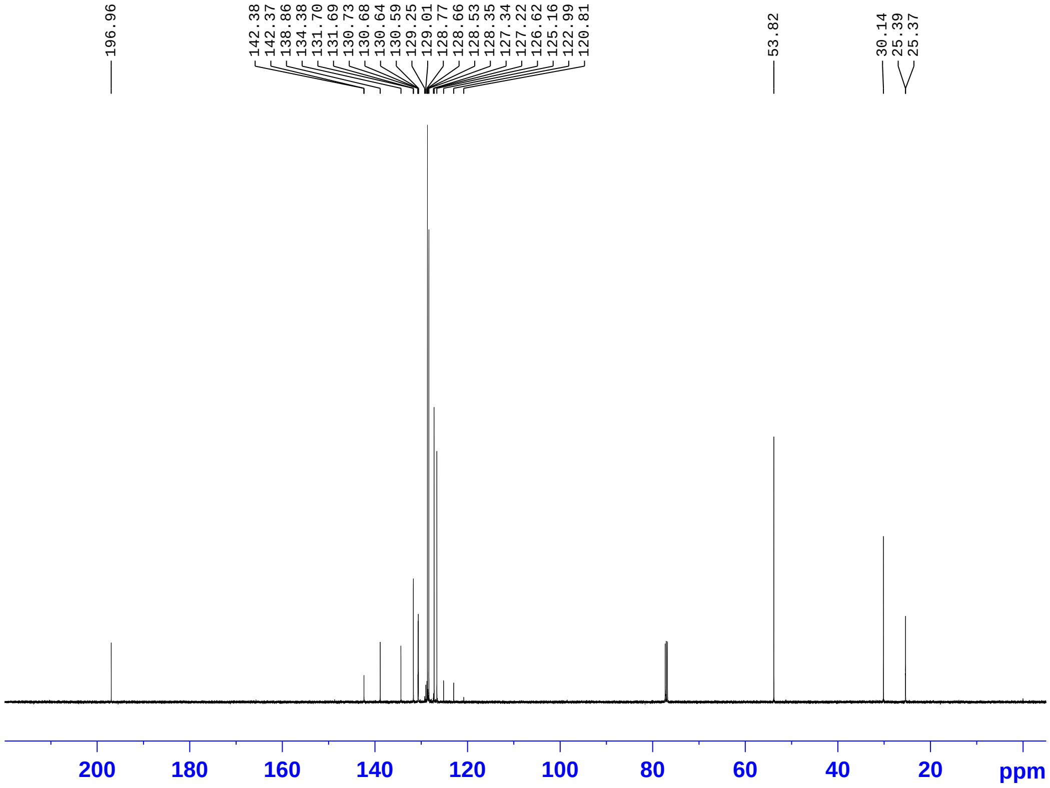

^{19}**F NMR** spectra of **4g**:

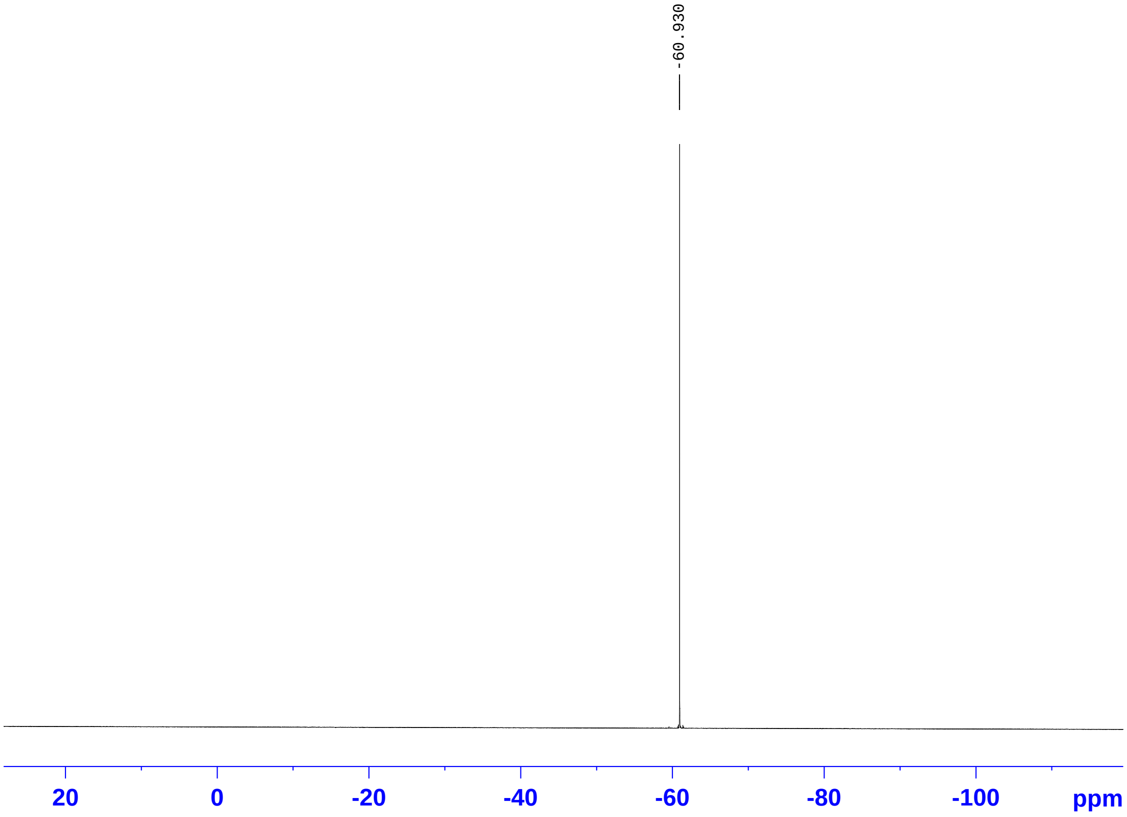
-60.930
20
0
-20
-40
-60
-80
-100
ppm

^{1}H NMR spectra of **4h**:

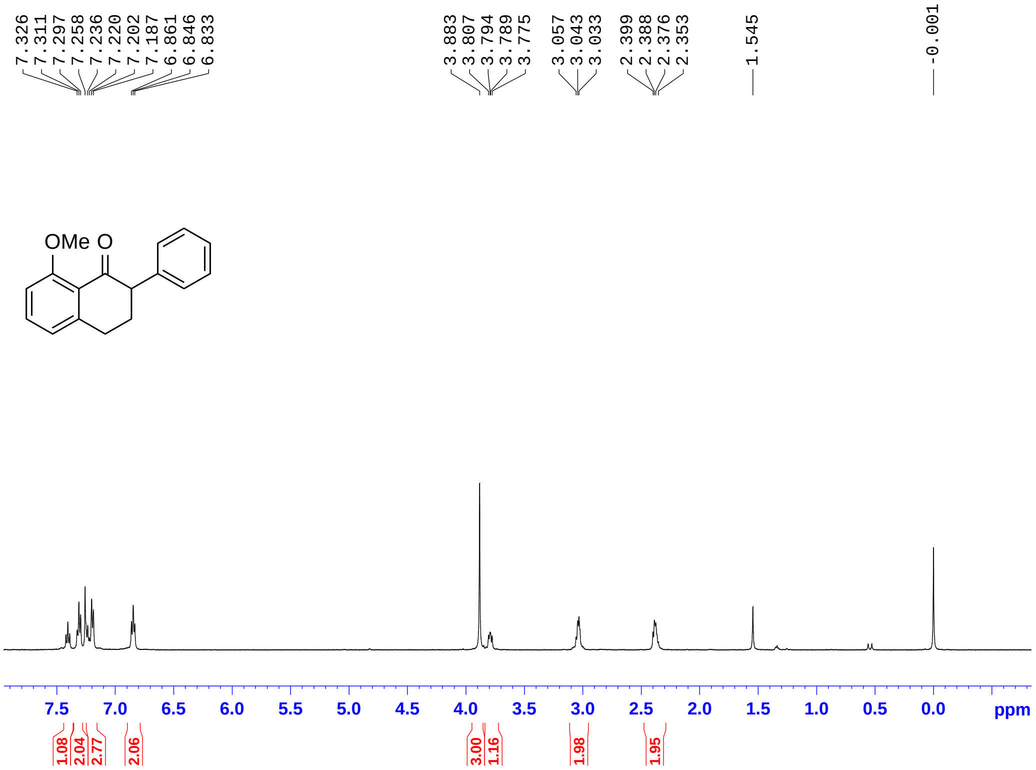

^{13}C NMR spectra of **4h**:

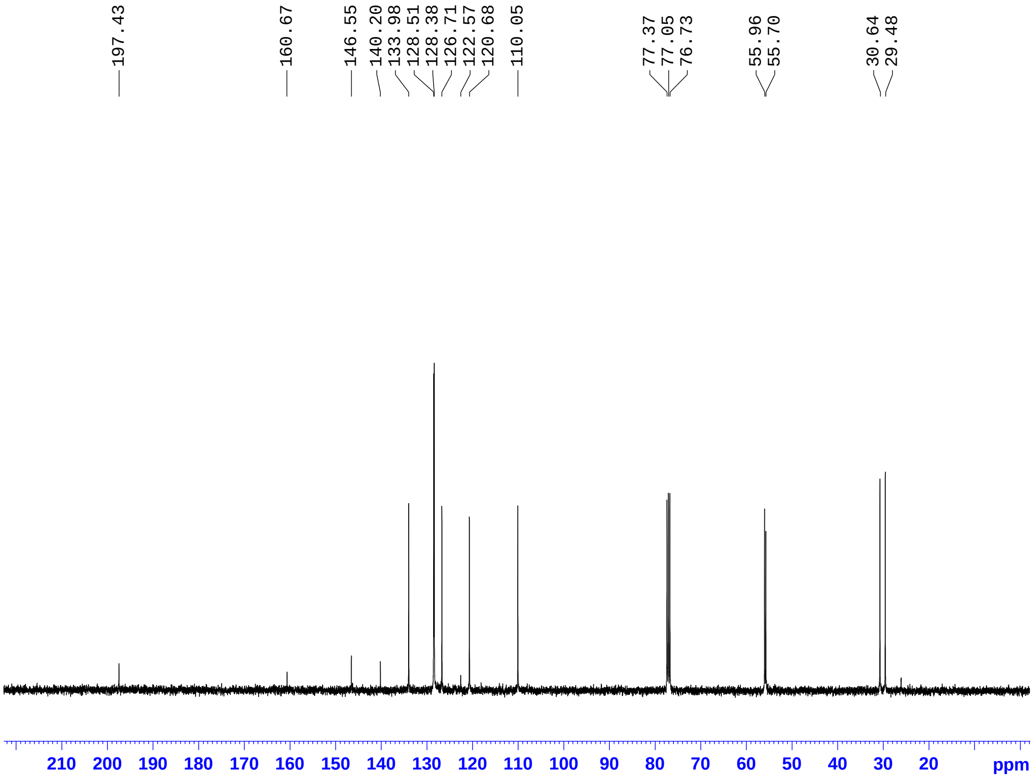

^{1}**H NMR** spectra of **4i**:

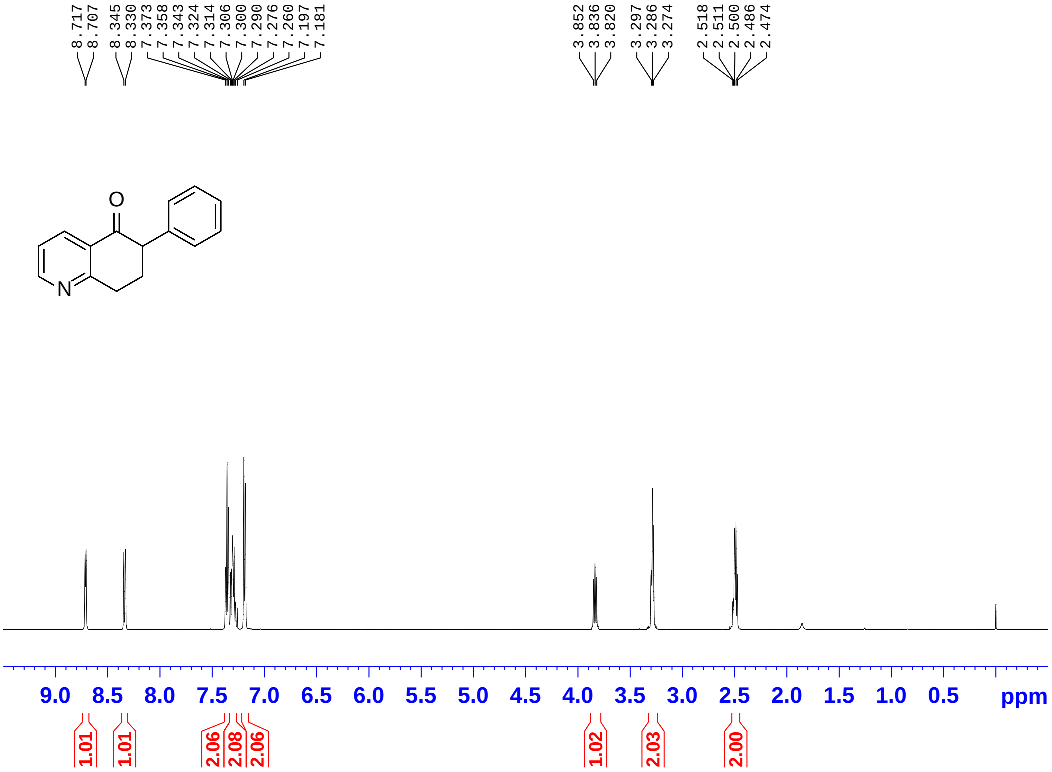

^{13}**C NMR** spectra of **4i**:

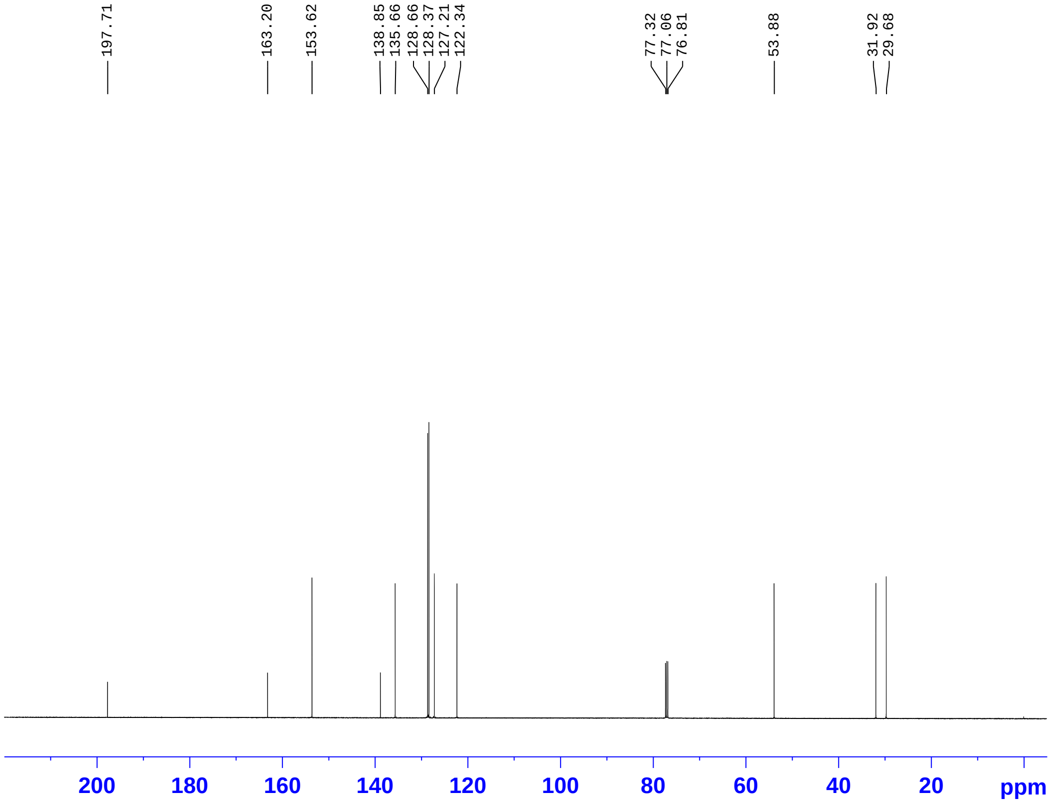

^{1}H NMR spectra of **4j**:

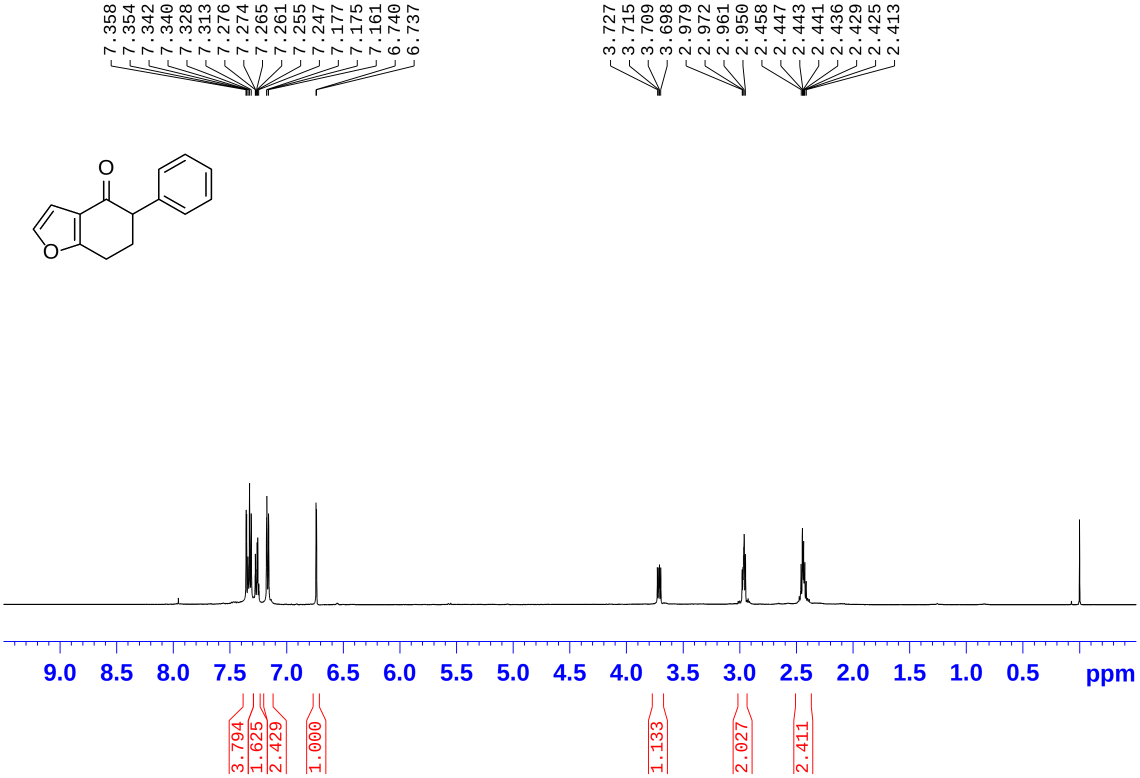

^{13}C NMR spectra of **4j**:

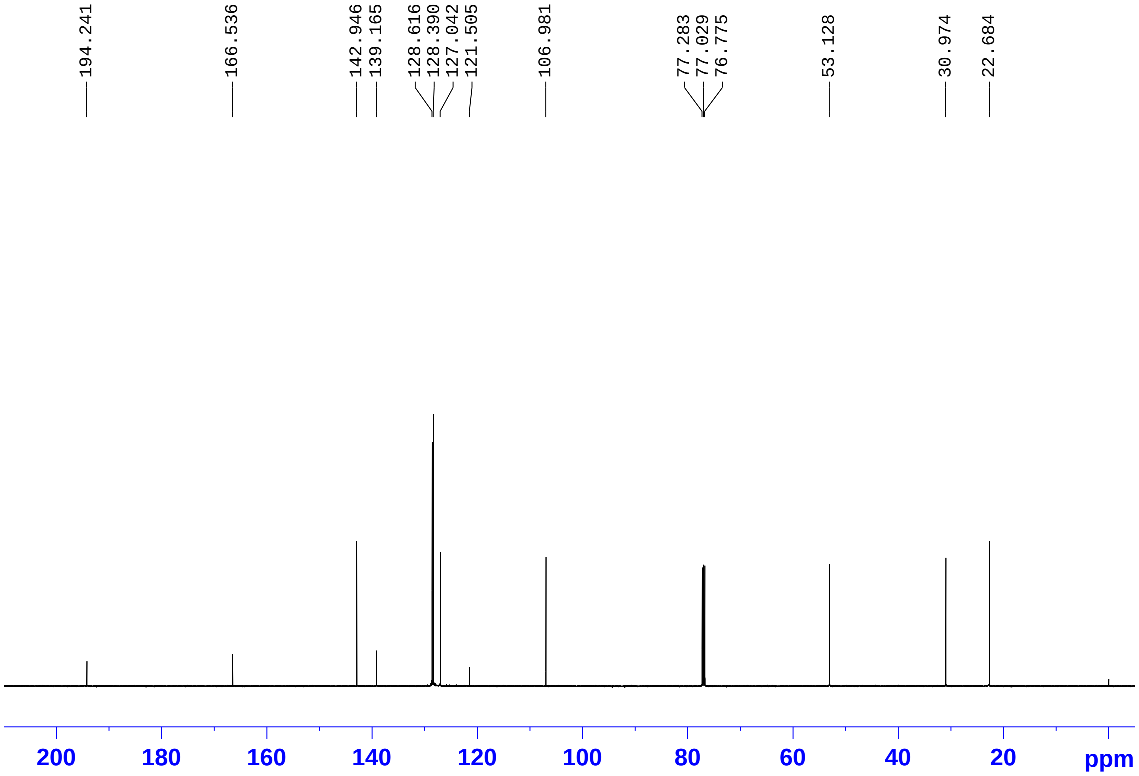

^{1}H **NMR** spectra of **4k**:

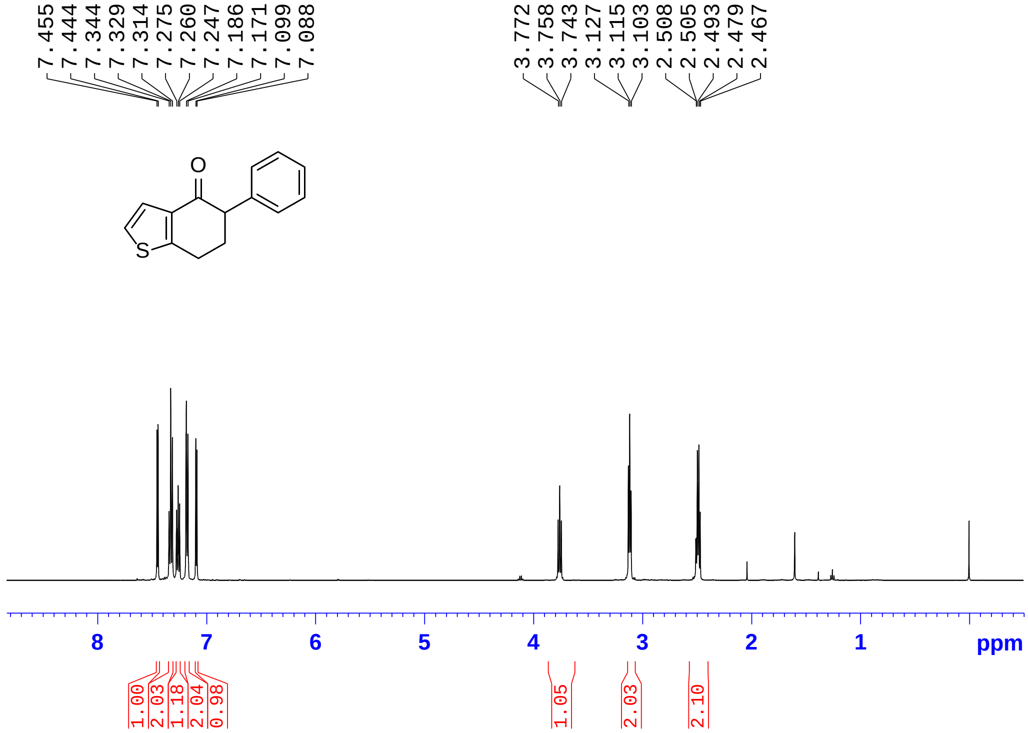

^{13}C **NMR** spectra of **4k**:

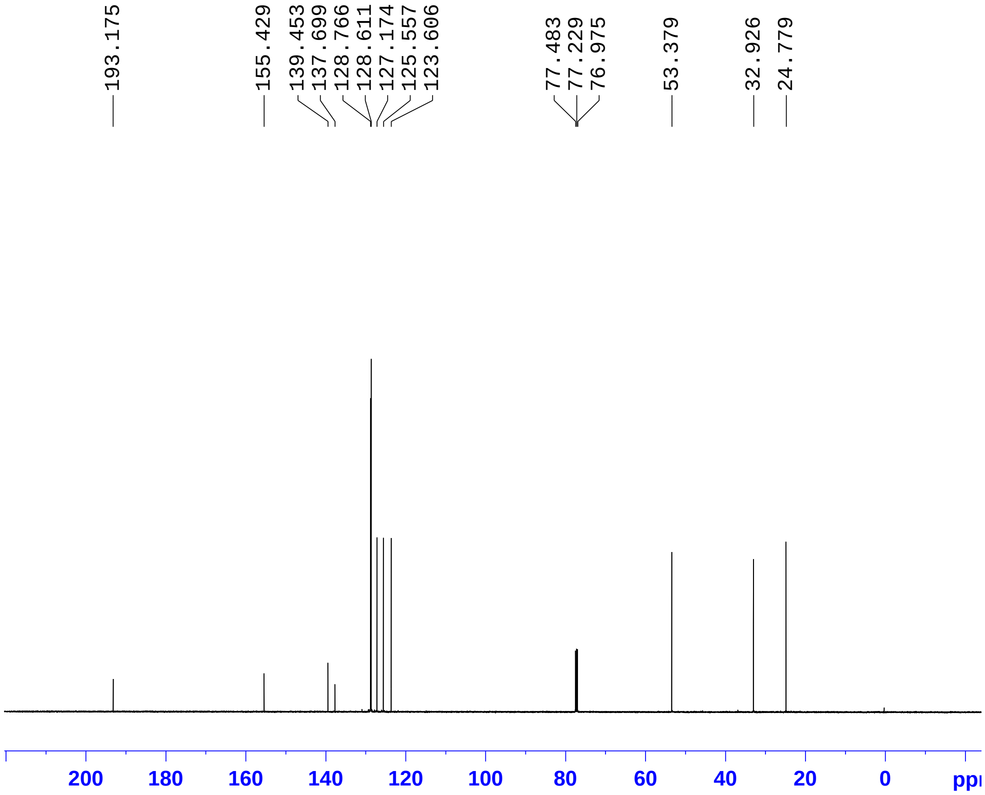

^{1}H NMR spectra of **4l**:

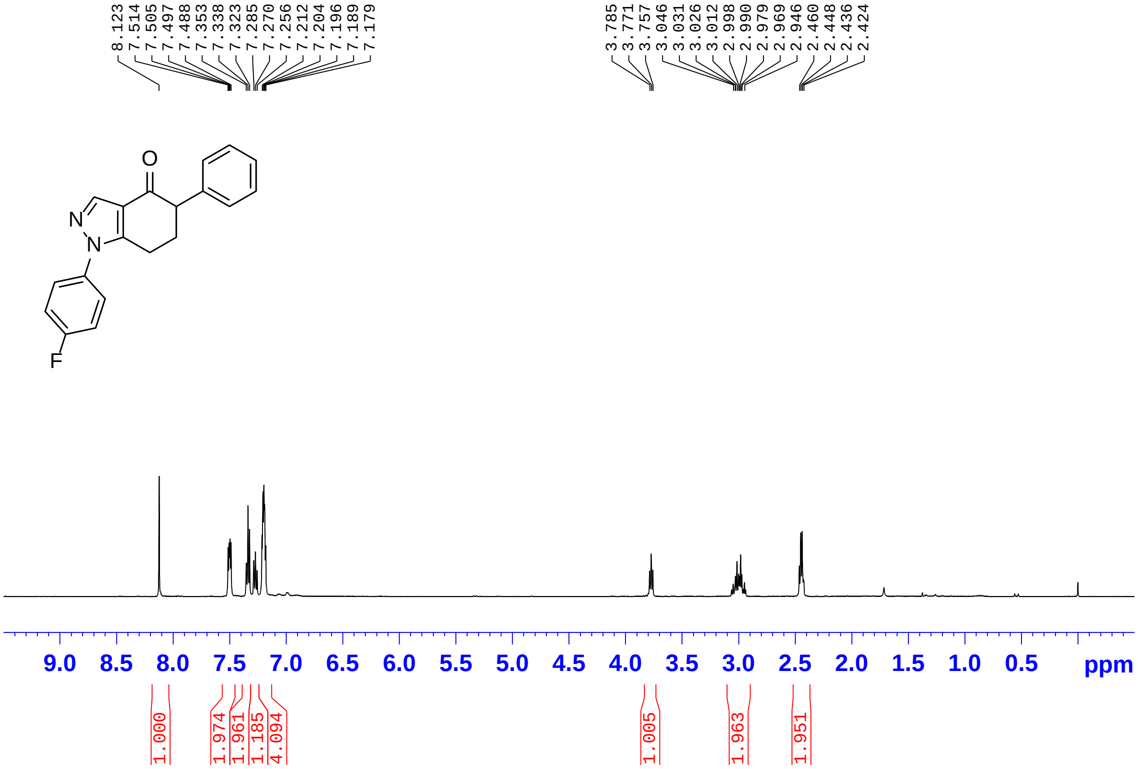

^{13}C NMR spectra of **4l**:

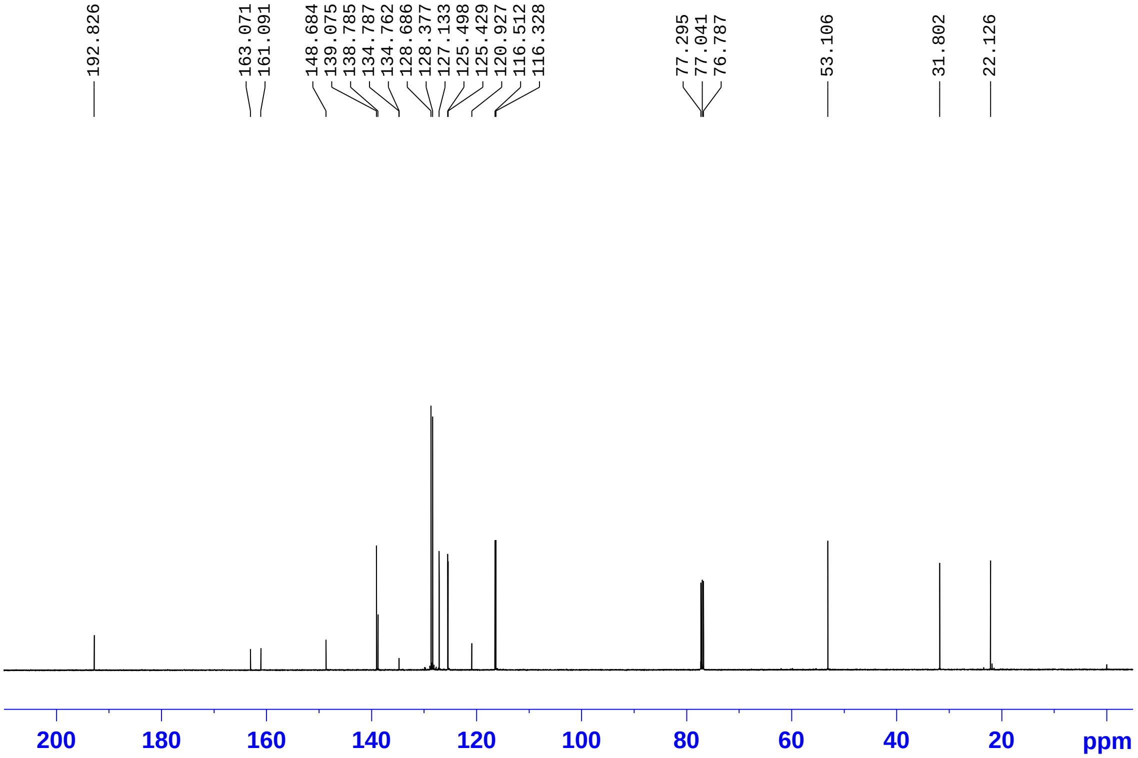

^{19}F NMR spectra of **4l**:

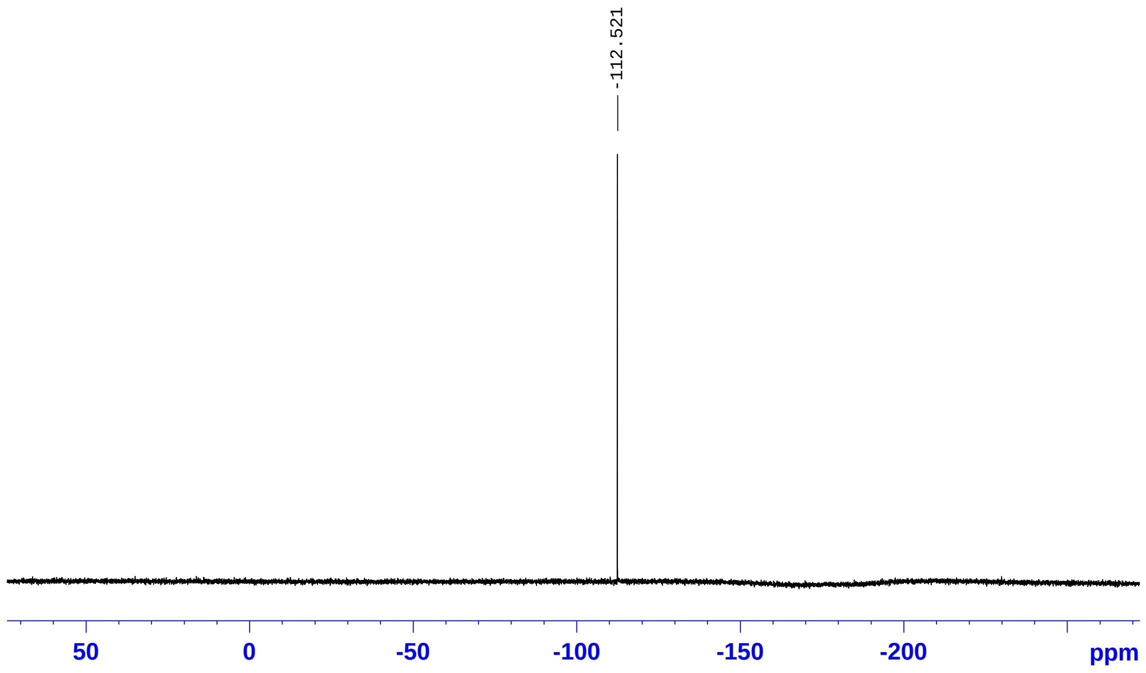
-112.521
50
0
-50
-100
-150
-200
ppm

^{1}H NMR spectra of **4o**:

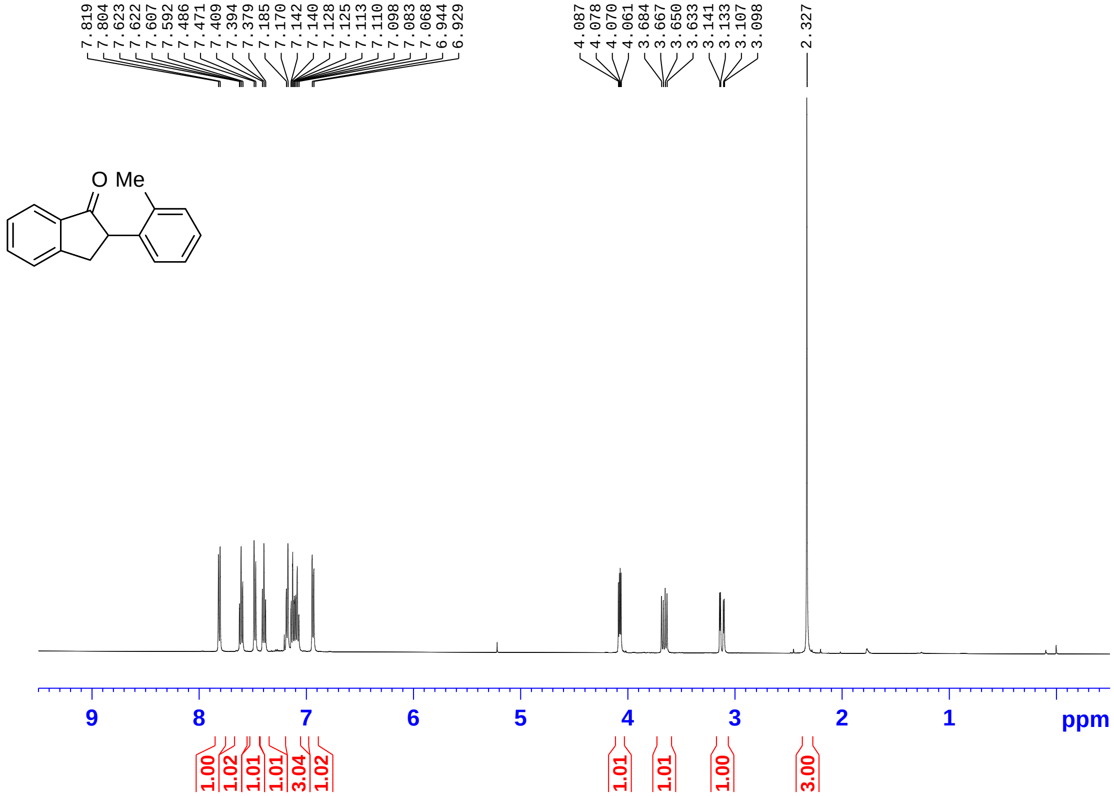

^{13}C NMR spectra of **4o**:

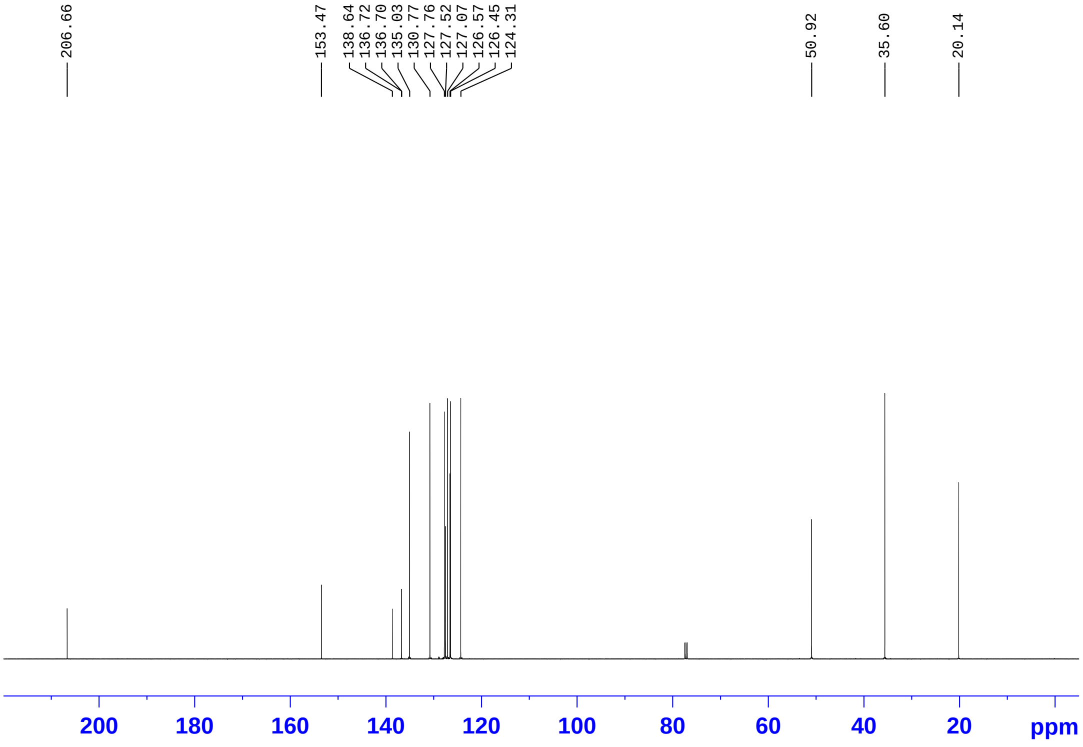

^{1}H NMR spectra of **4q**:

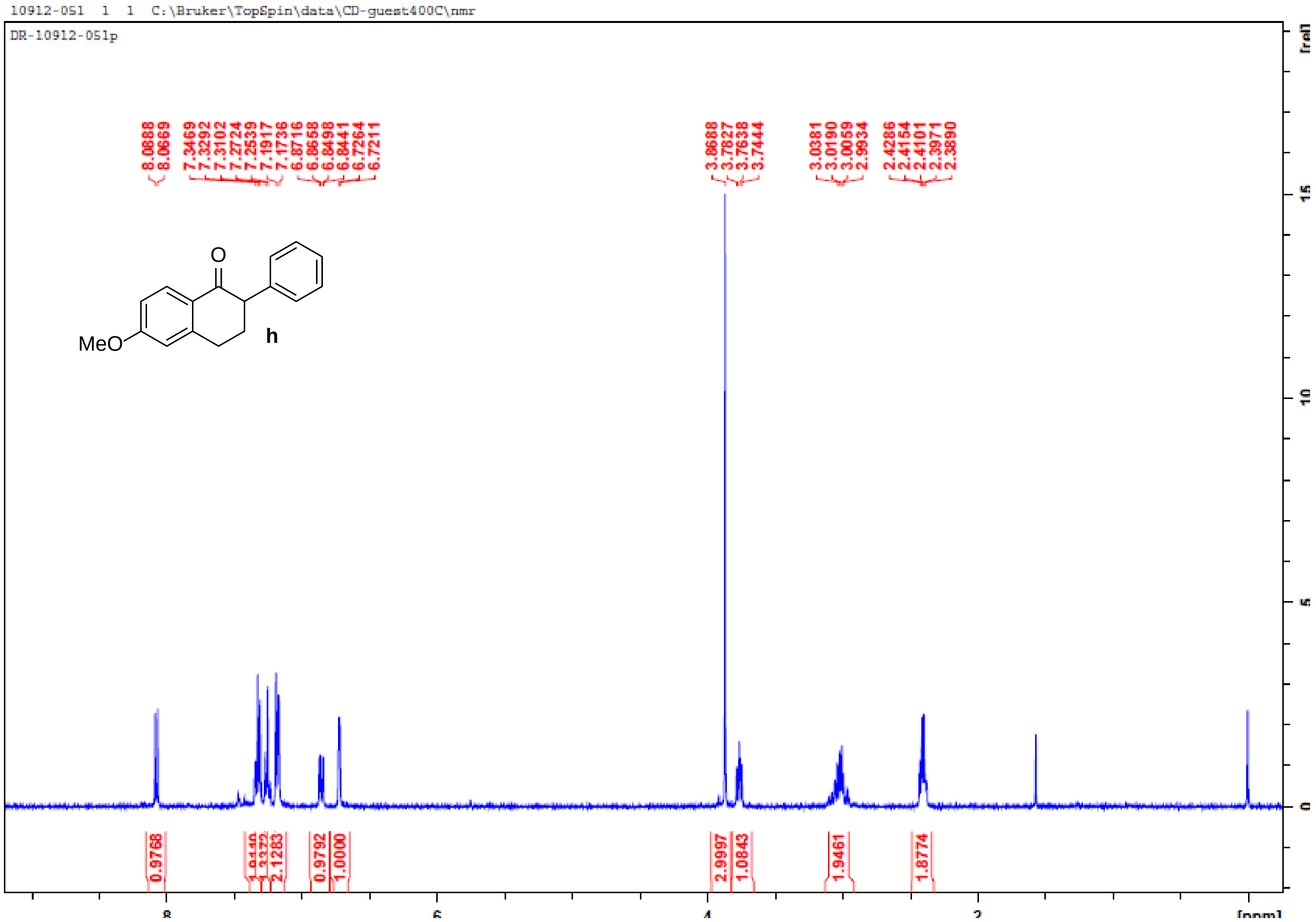
10912-051 1 1 C:\Bruker\TopSpin\data\CD-guest400C\nmr
DR-10912-051p
8.0888
8.0669
7.3469
7.3292
7.3102
7.2724
7.2539
7.1917
7.1736
6.8716
6.8658
6.8498
6.8441
6.7264
6.7211
3.8688
3.7827
3.7638
3.7444
3.0381
3.0190
3.0059
2.9934
2.4286
2.4154
2.4101
2.3971
2.3890
O
MeO
h
0.9768
2.1283
0.9792
1.0000
2.9997
1.0843
1.9461
1.8774
[rel]
15
10
5
0
8
6
4
2
[ppm]

^{1}H NMR spectra of **5a:**

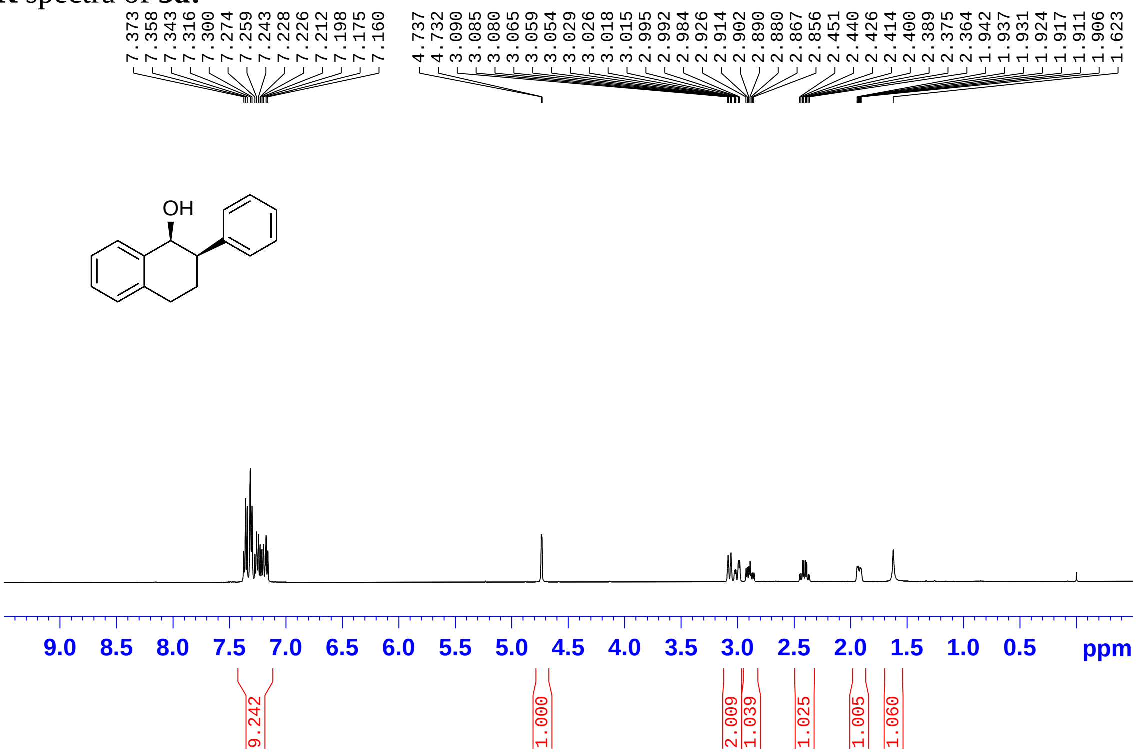

^{13}C NMR spectra of **5a:**

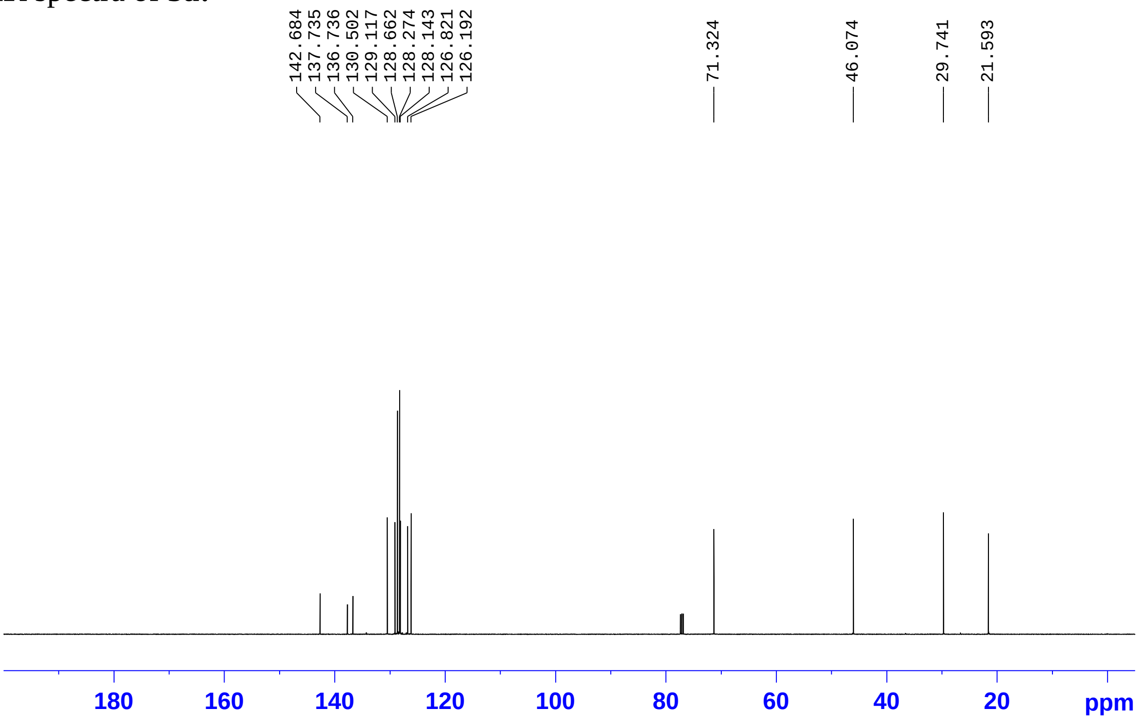

Chiral HPLC chromatogram of **5a**:

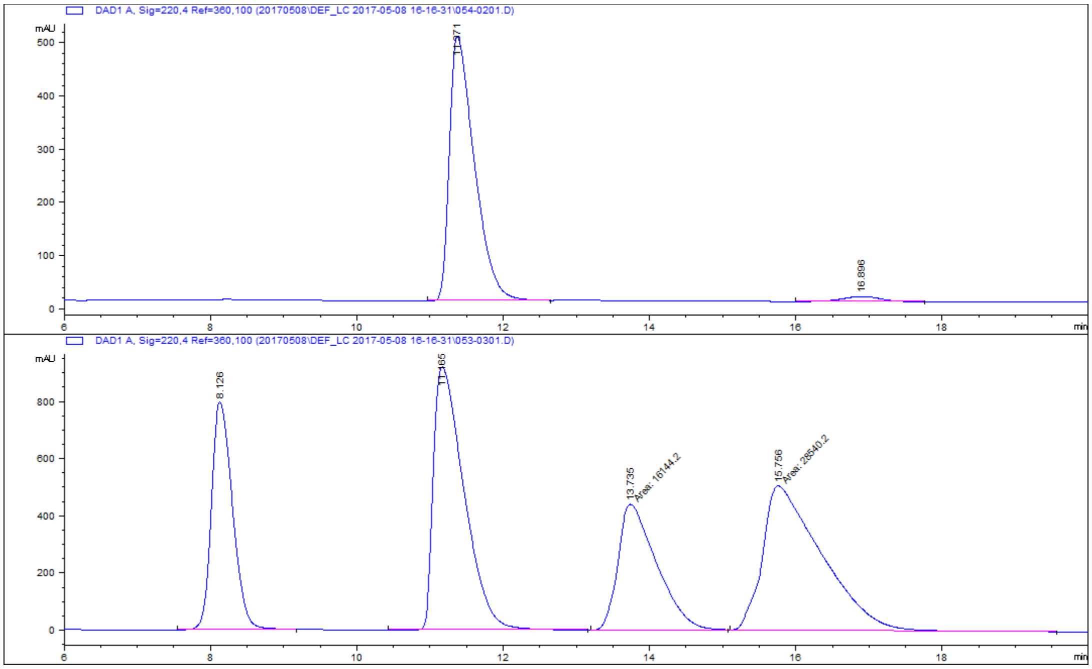

Integration results of **5a**:

Peak #	RetTime [min]	Type	Width [min]	Area [mAU*s]	Height [mAU]	Area %
1	11.371	BB	0.3574	1.18046e4	496.45444	97.1440
2	16.896	BB	0.5493	347.05096	9.65596	2.8560

Integration results of racemic sample:

Peak #	RetTime [min]	Type	Width [min]	Area [mAU*s]	Height [mAU]	Area %
1	8.126	BB	0.3102	1.58454e4	795.98370	18.1495
2	11.165	BB	0.4430	2.67753e4	921.35162	30.6686
3	13.735	MM	0.6114	1.61442e4	440.10245	18.4917
4	15.756	MM	0.9391	2.85402e4	506.52402	32.6902

^{1}**H NMR** spectra of **5b**:

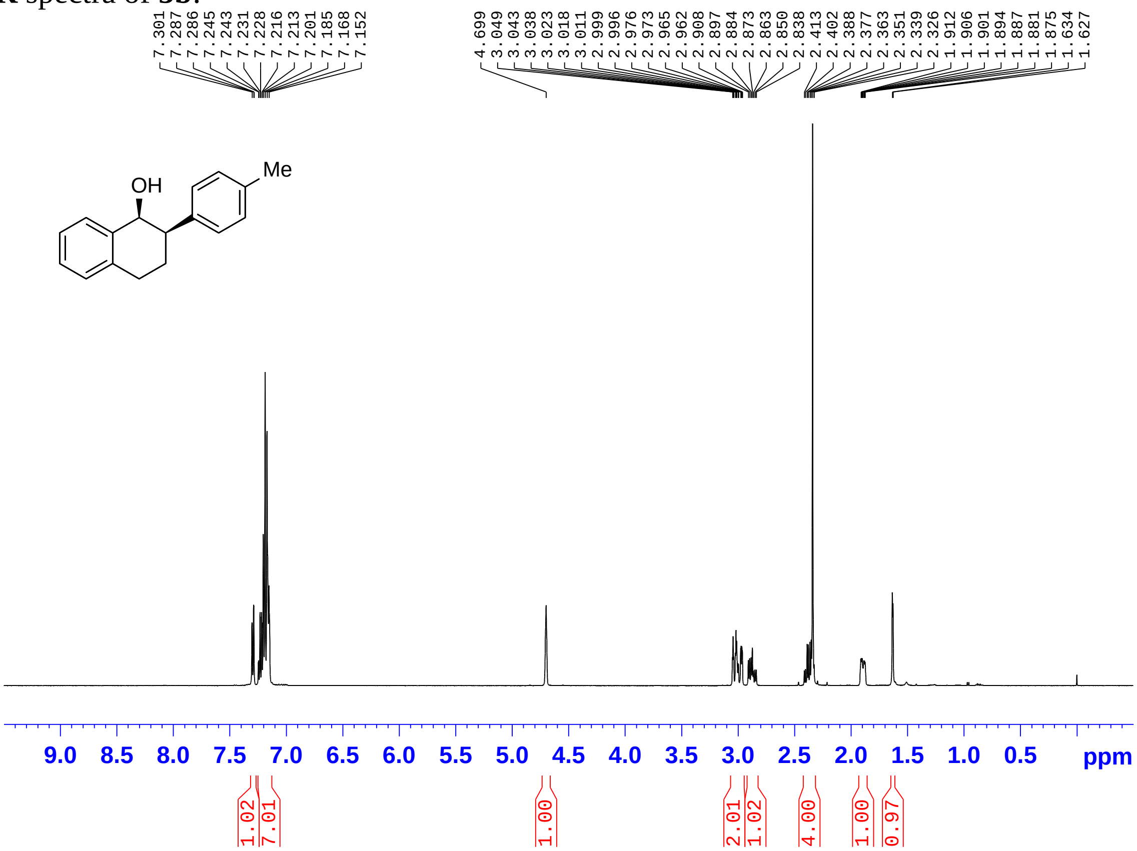

^{13}**C NMR** spectra of **5b**:

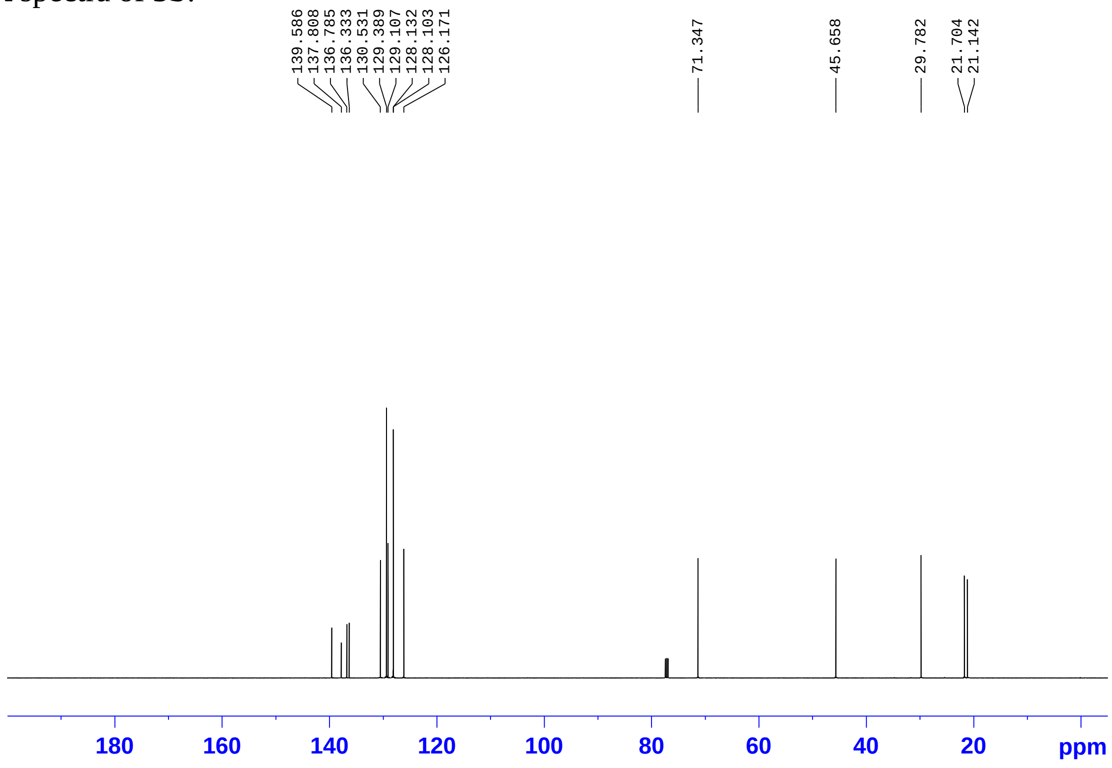

Chiral HPLC chromatogram of **5b**:

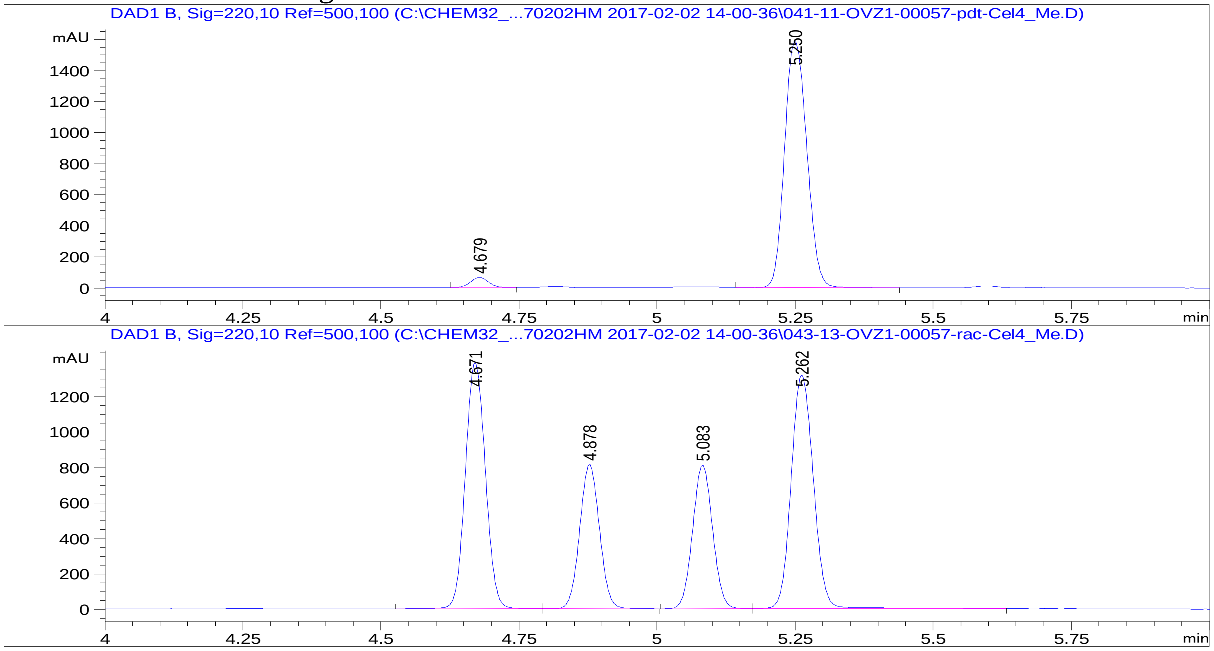

Integration results of **5b**:

Signal 1: DAD1 B, Sig=220,10 Ref=500,100

Peak #	RetTime [min]	Type	Width [min]	Area [mAU*s]	Height [mAU]	Area %
1	4.679	BB	0.0348	145.03253	64.22317	3.1680
2	5.250	VV R	0.0447	4433.04883	1581.95068	96.8320

Integration results of racemic sample:

Peak #	RetTime [min]	Type	Width [min]	Area [mAU*s]	Height [mAU]	Area %
1	4.671	VB R	0.0395	3502.89209	1385.60474	31.1185
2	4.878	BB	0.0390	2021.61511	812.61169	17.9594
3	5.083	BB	0.0394	2034.47156	807.88593	18.0736
4	5.262	BV R	0.0438	3697.63062	1316.00940	32.8485

^{1}H NMR spectra of **5c**:

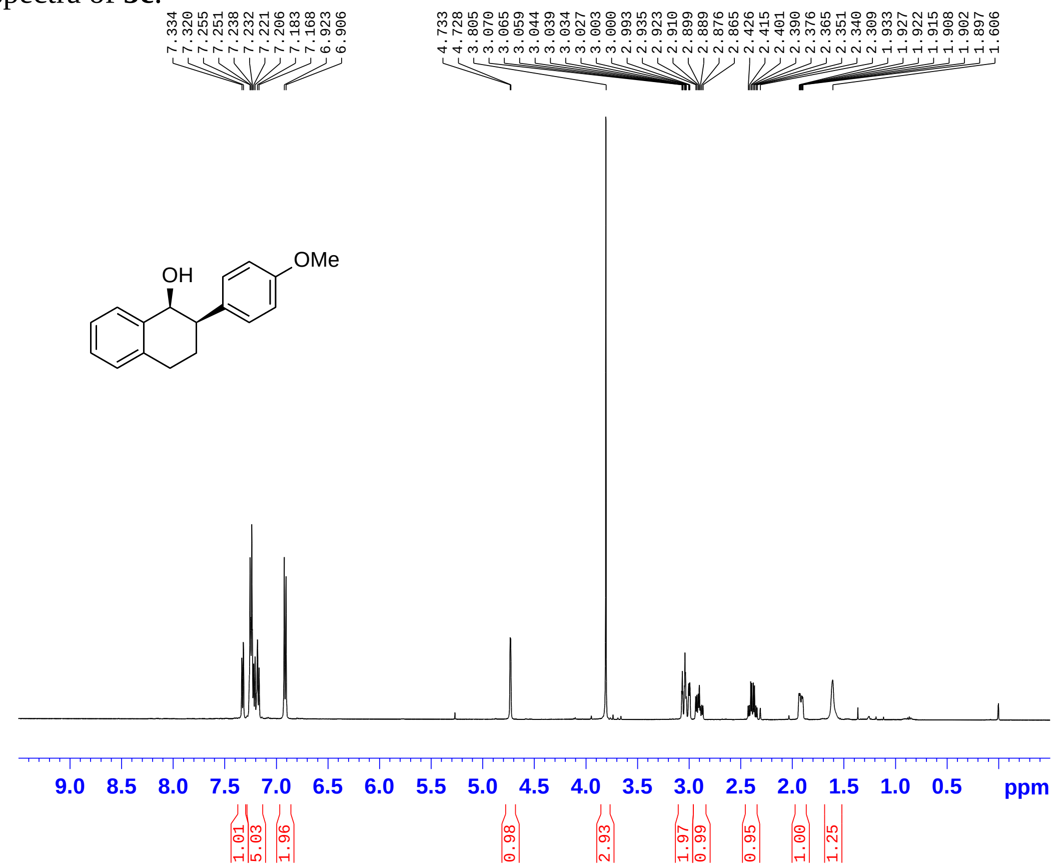

^{13}C NMR spectra of **5c**:

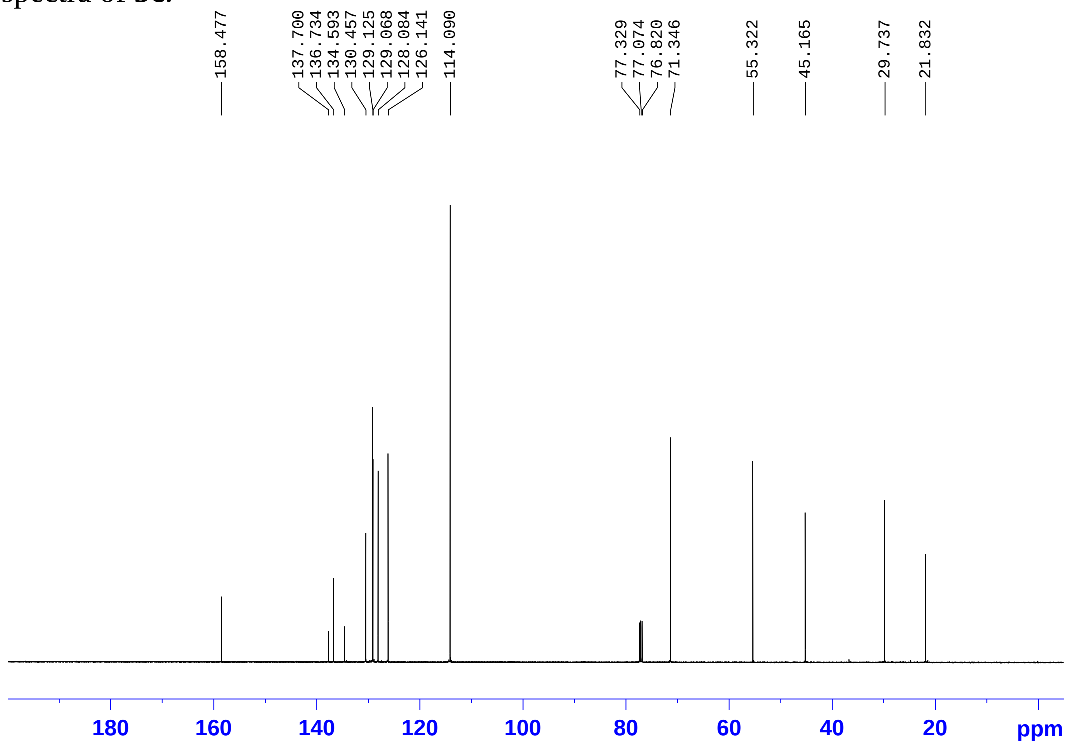

Chiral HPLC chromatogram of **5c**:

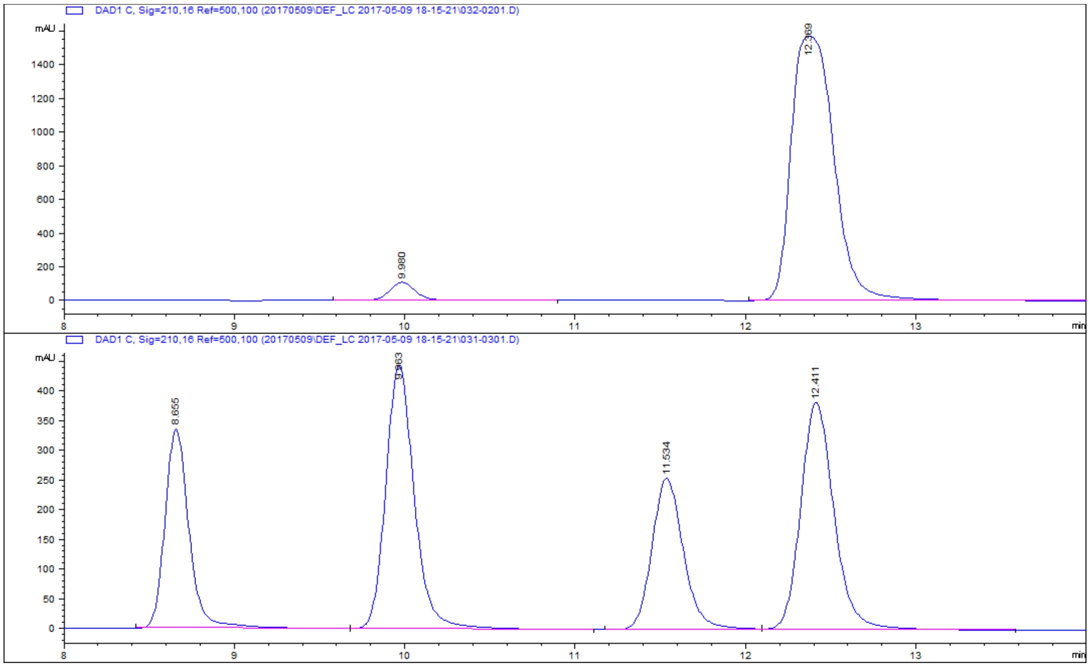

Integration results of **5c**:

Peak #	RetTime [min]	Type	Width [min]	Area [mAU*s]	Height [mAU]	Area %
1	9.980	BB	0.1607	1124.09387	106.89407	3.9482
2	12.369	BB	0.2774	2.73470e4	1568.77405	96.0518

Integration results of racemic sample:

Peak #	RetTime [min]	Type	Width [min]	Area [mAU*s]	Height [mAU]	Area %
1	8.655	BB	0.1502	3291.96118	336.30981	19.3968
2	9.963	BB	0.1779	5160.78076	443.16150	30.4083
3	11.534	BV	0.1985	3289.01587	255.02499	19.3795
4	12.411	VB	0.2095	5229.88818	382.59079	30.8154

^{1}H NMR spectra of **5d**:

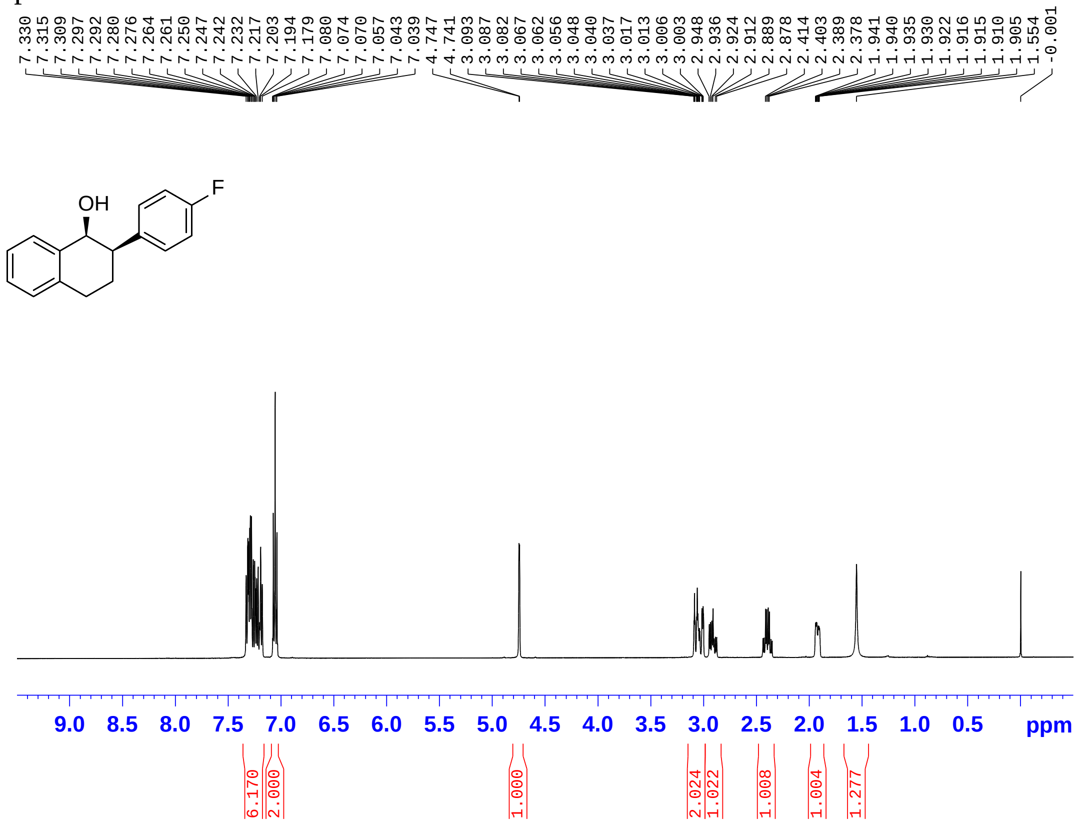

^{13}C NMR spectra of **5d**:

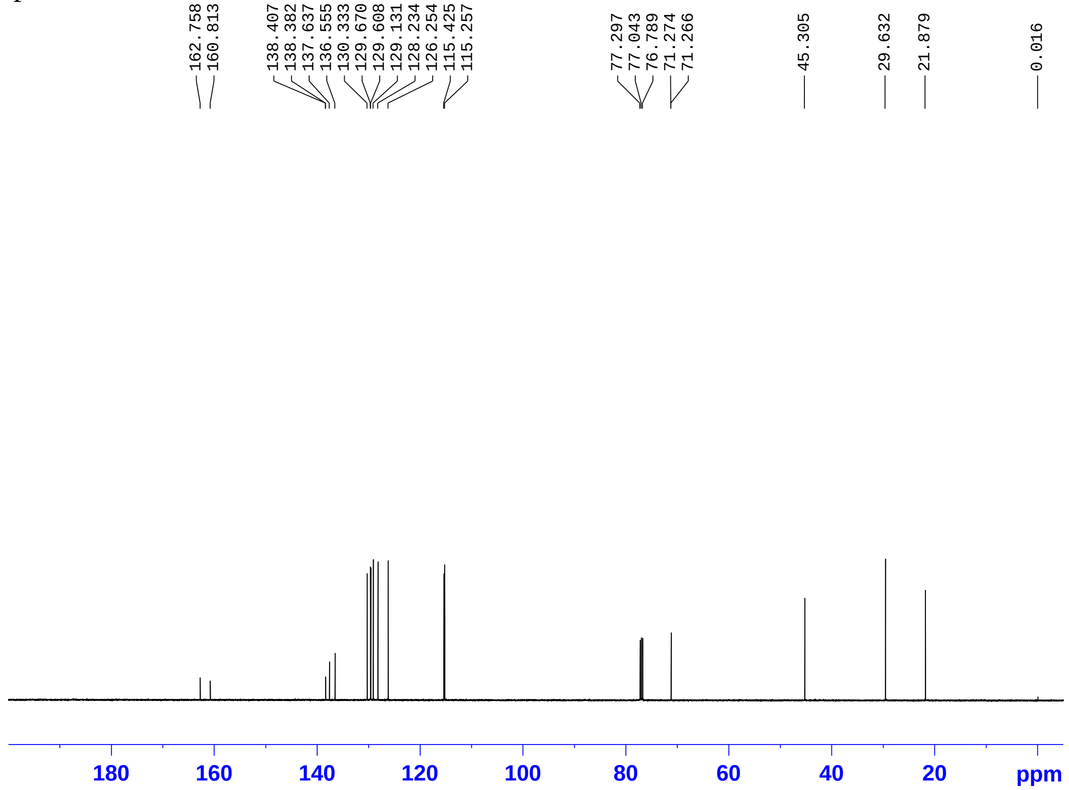

^{19}F NMR spectra of **5d**:

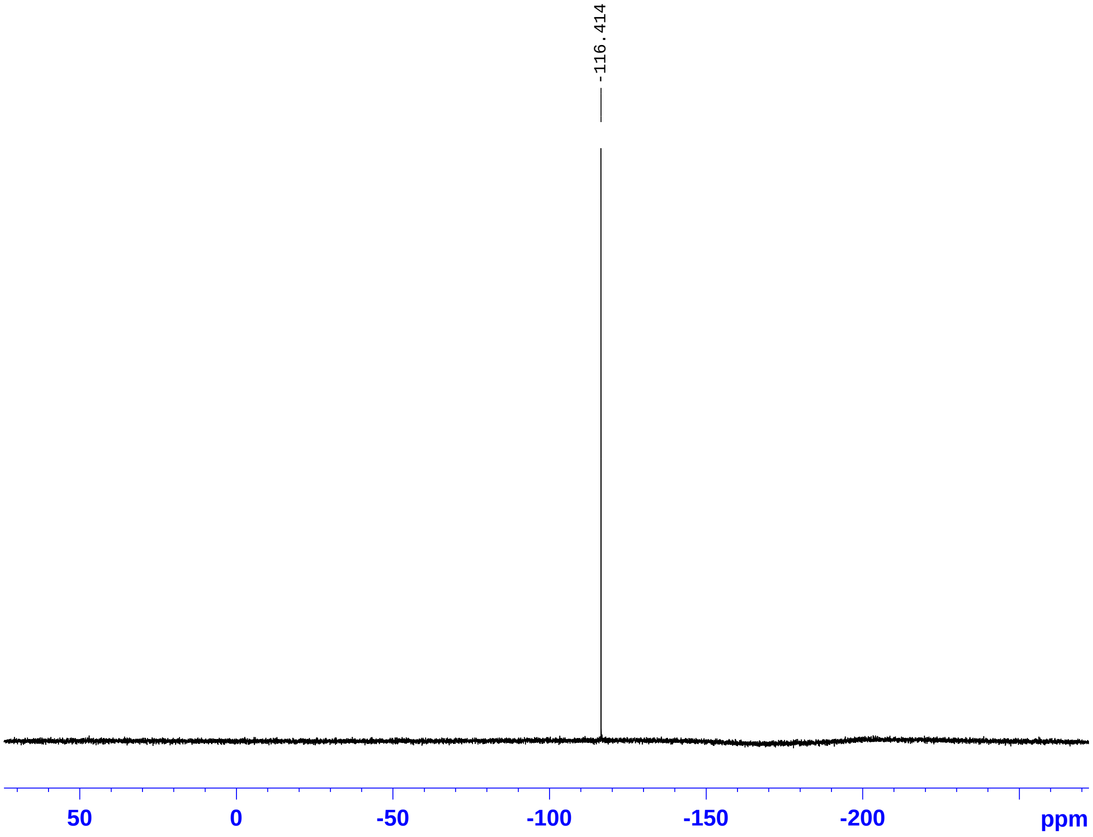
-116.414
50
0
-50
-100
-150
-200
ppm

Chiral HPLC chromatogram of **5d**:

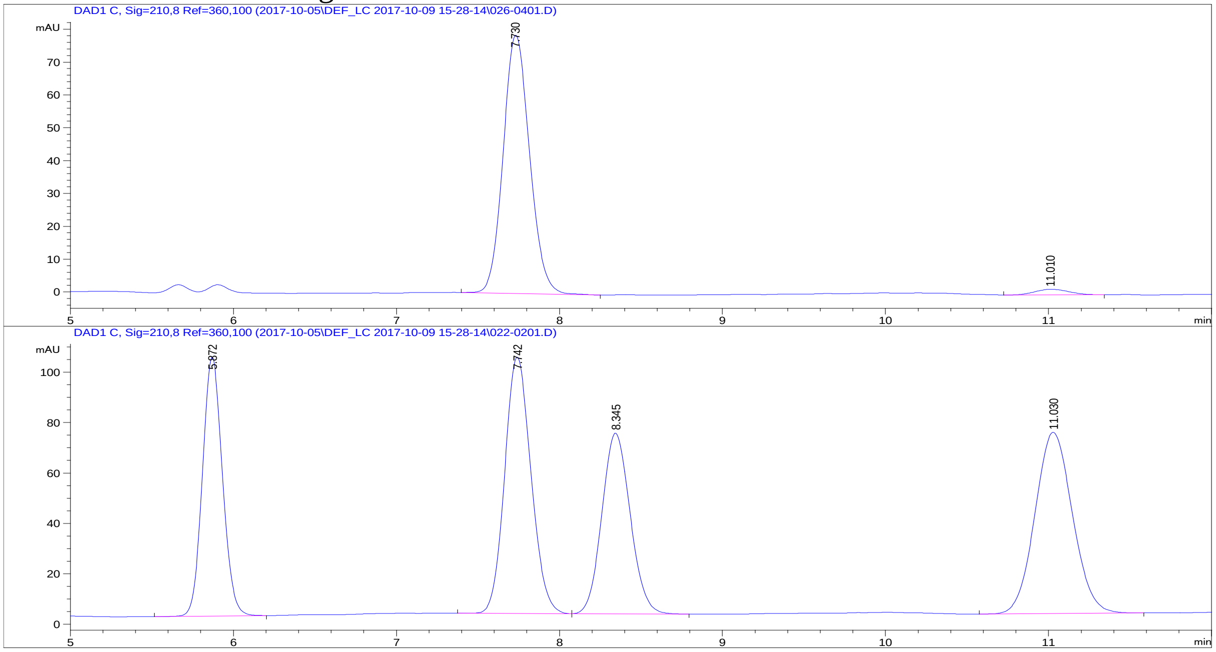

Integration results of **5d**:

Peak #	RetTime [min]	Type	Width [min]	Area [mAU*s]	Height [mAU]	Area %
1	7.730	BB	0.1756	887.13446	78.66509	97.2412
2	11.010	BB	0.2289	25.16884	1.73887	2.7588

Integration results of racemic sample:

Peak #	RetTime [min]	Type	Width [min]	Area [mAU*s]	Height [mAU]	Area %
1	5.872	BB	0.1313	875.51825	102.87659	21.7183
2	7.742	BB	0.1747	1140.68054	101.80892	28.2960
3	8.345	BB	0.1890	867.59888	71.79945	21.5219
4	11.030	BB	0.2482	1147.44360	71.90237	28.4638

^{1}H NMR spectra of **5e**:

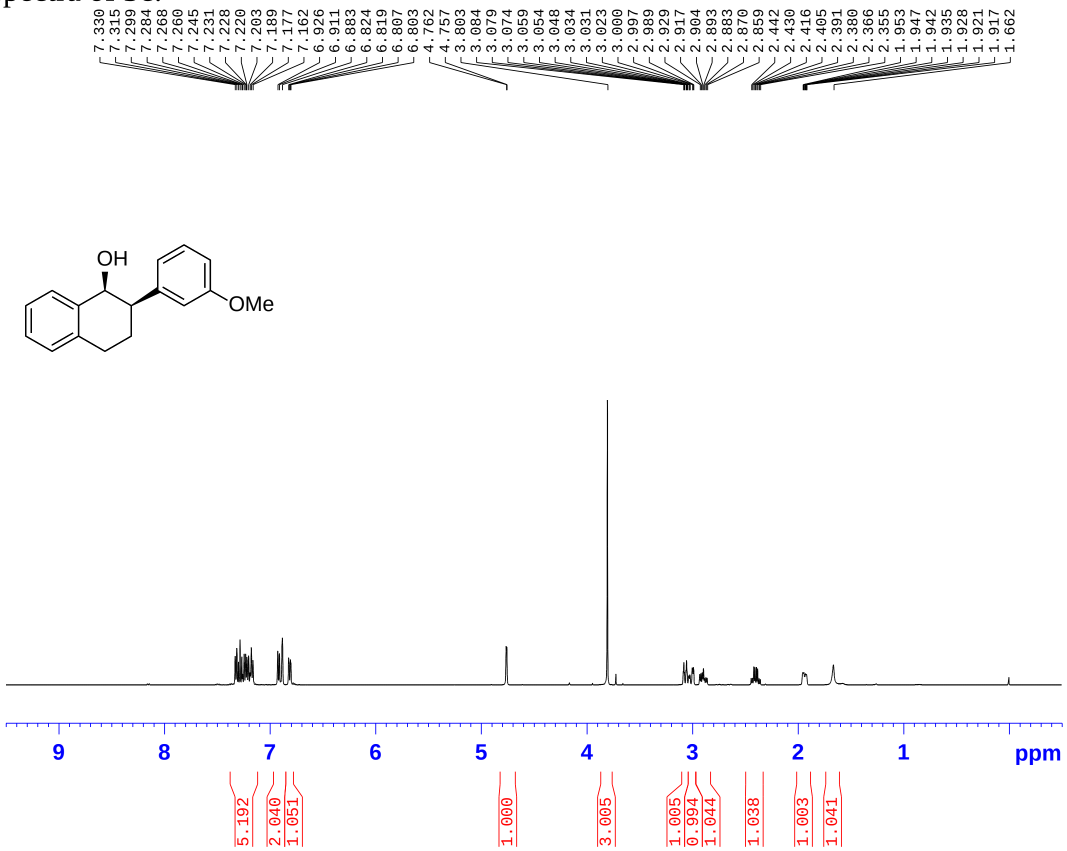

^{13}C NMR spectra of **5e**:

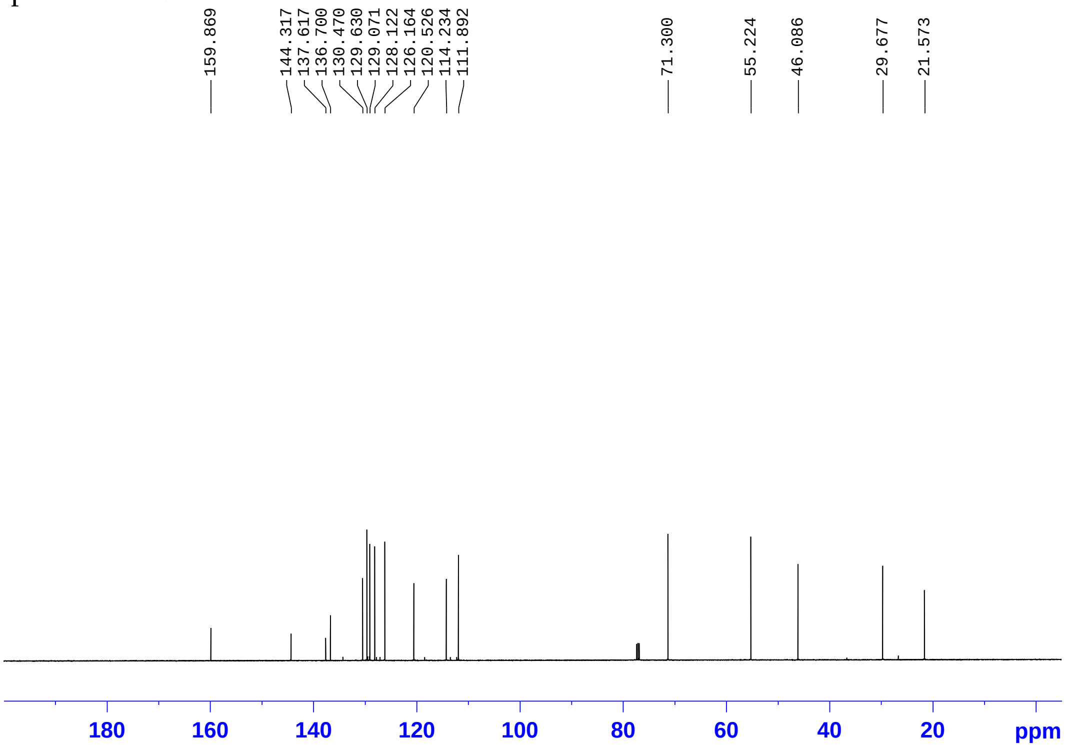

Chiral HPLC chromatogram of **5e**:

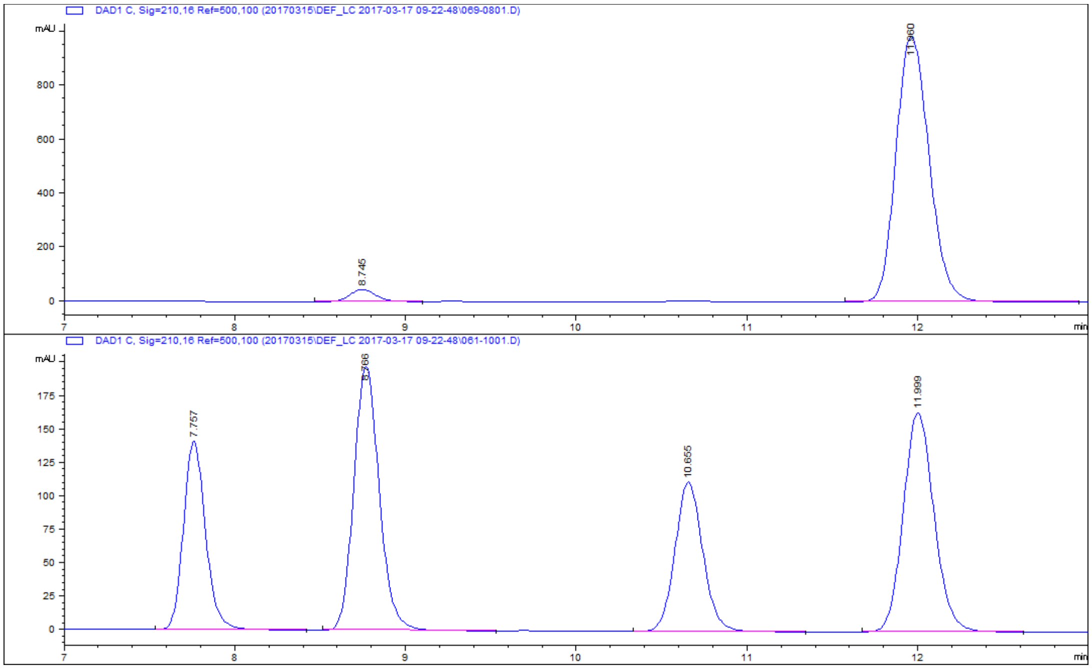

Integration results of **5e**:

Peak #	RetTime [min]	Type	Width [min]	Area [mAU*s]	Height [mAU]	Area %
1	8.745	BB	0.1610	466.65134	45.00516	3.4057
2	11.960	BB	0.2112	1.32356e4	982.58521	96.5943

Integration results of racemic sample:

Peak #	RetTime [min]	Type	Width [min]	Area [mAU*s]	Height [mAU]	Area %
1	7.757	BB	0.1385	1261.76868	140.91795	19.0583
2	8.766	BB	0.1608	2041.47729	197.23346	30.8353
3	10.655	BB	0.1739	1265.79041	111.93693	19.1190
4	11.999	BB	0.1940	2051.55005	163.93501	30.9874

^{1}**H NMR** spectra of **5f**:

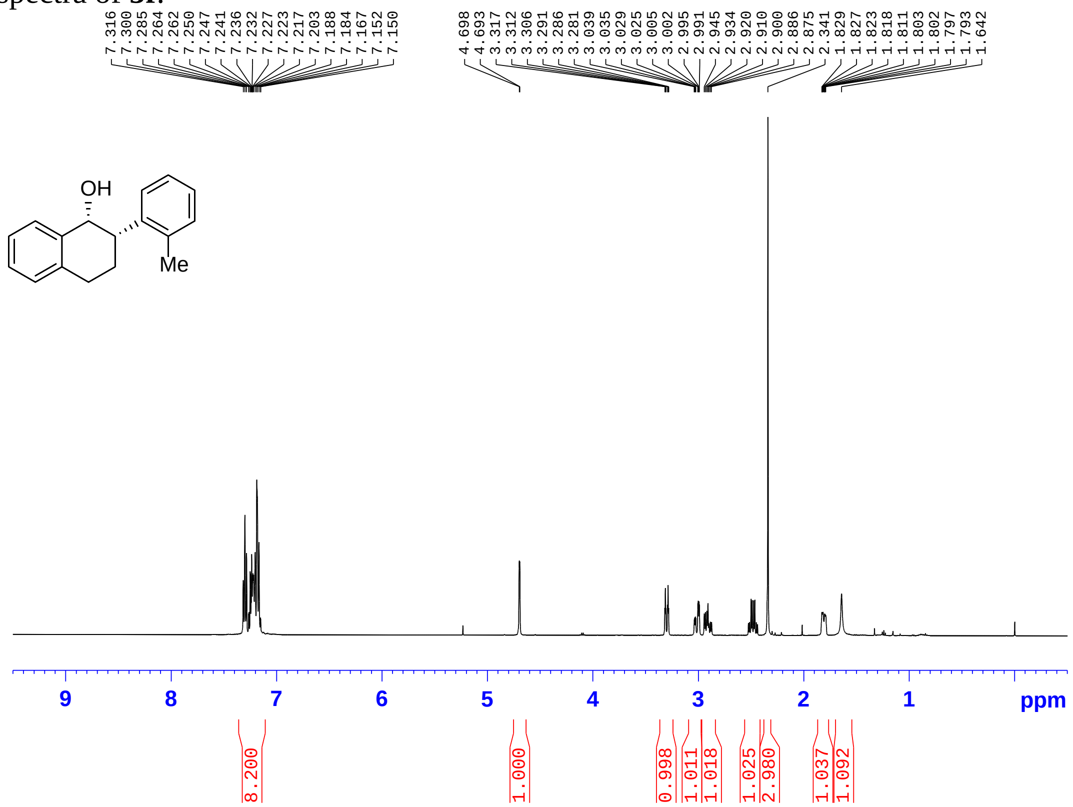

^{13}**C NMR** spectra of **5f**:

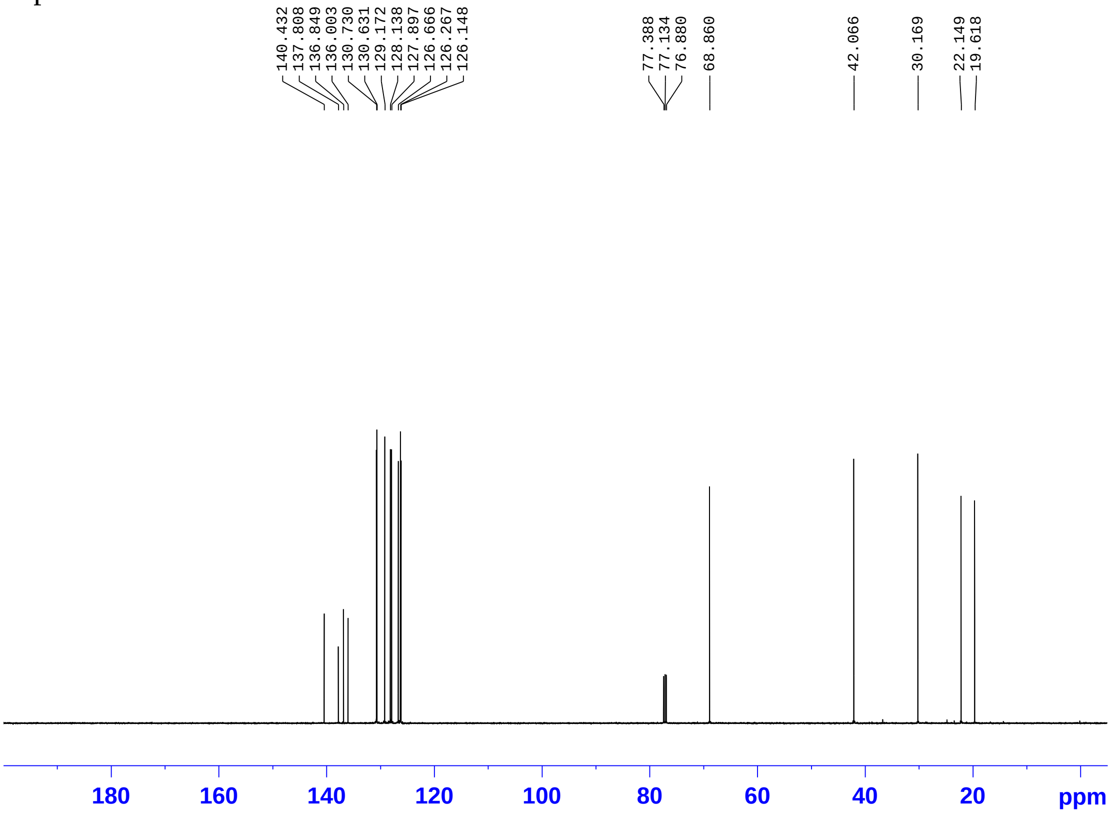

Chiral HPLC chromatogram of **5f**:

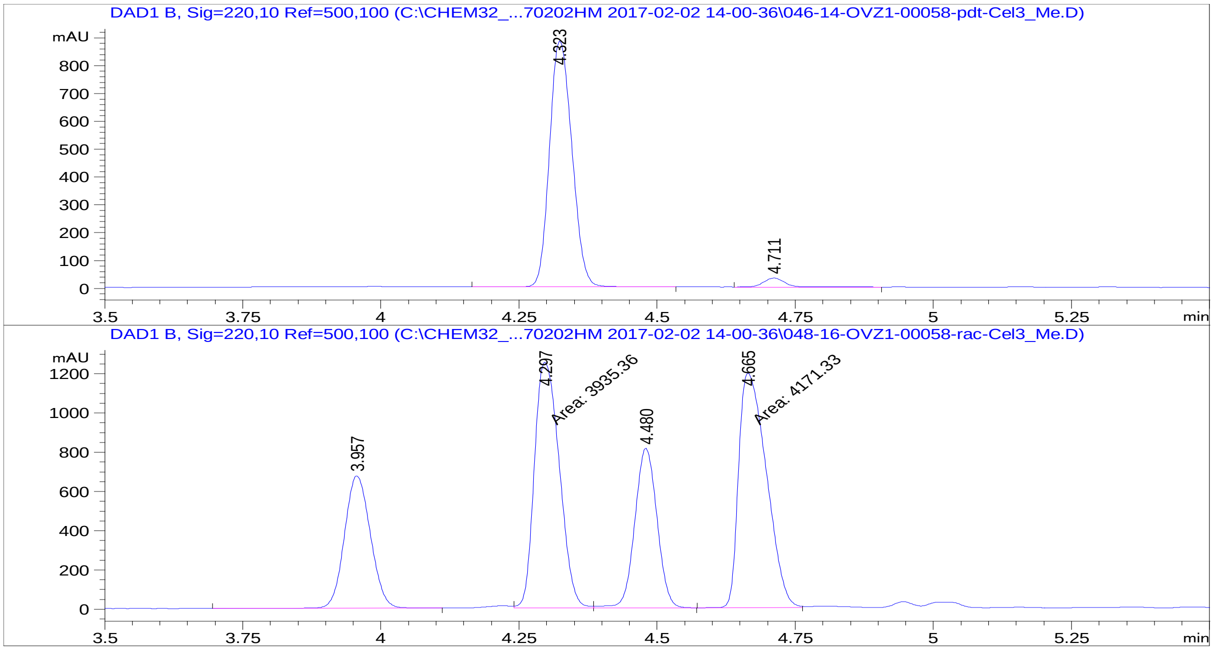

Integration results of **5f**:

Peak #	RetTime [min]	Type	Width [min]	Area [mAU*s]	Height [mAU]	Area %
1	4.323	VV R	0.0446	2500.51440	882.63251	96.6223
2	4.711	BV R	0.0416	87.41162	32.28168	3.3777

Integration results of racemic sample:

Peak #	RetTime [min]	Type	Width [min]	Area [mAU*s]	Height [mAU]	Area %
1	3.957	VV R	0.0517	2211.04126	674.94653	17.5616
2	4.297	FM	0.0524	3935.35913	1251.77527	31.2574
3	4.480	VB	0.0441	2272.45264	812.69342	18.0494
4	4.665	MF	0.0581	4171.33008	1196.09436	33.1316

^{1}H NMR spectra of **5g**:

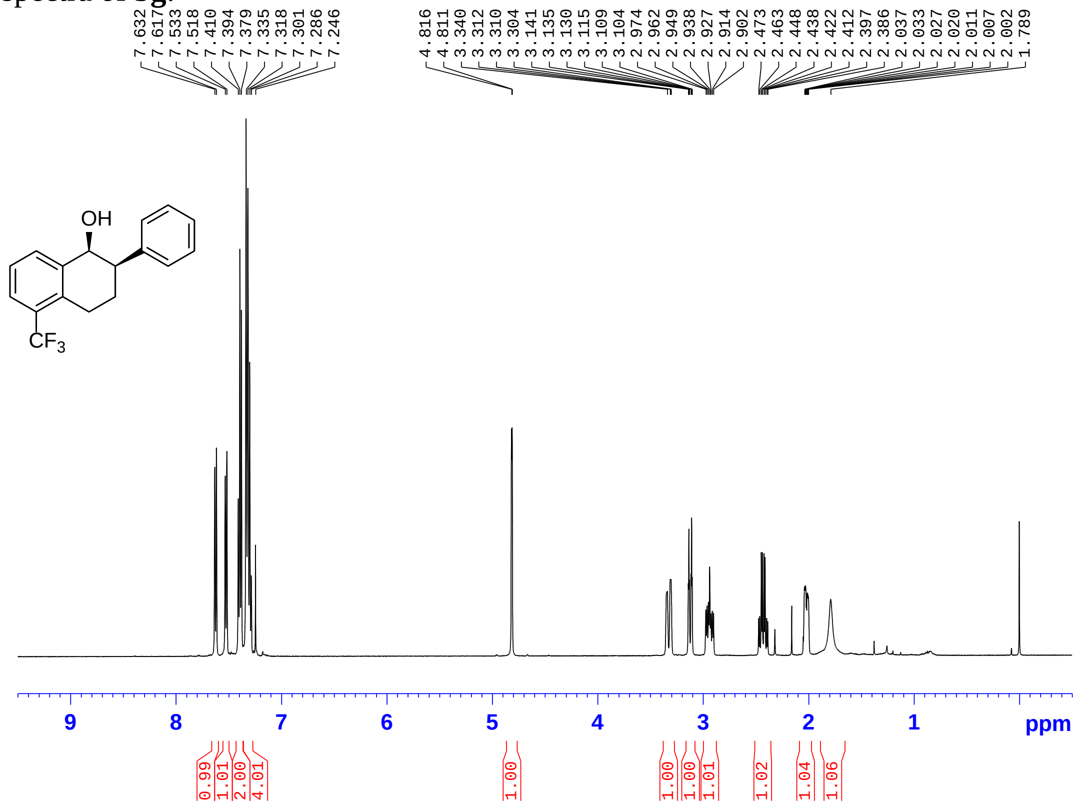

^{13}C NMR spectra of **5g**:

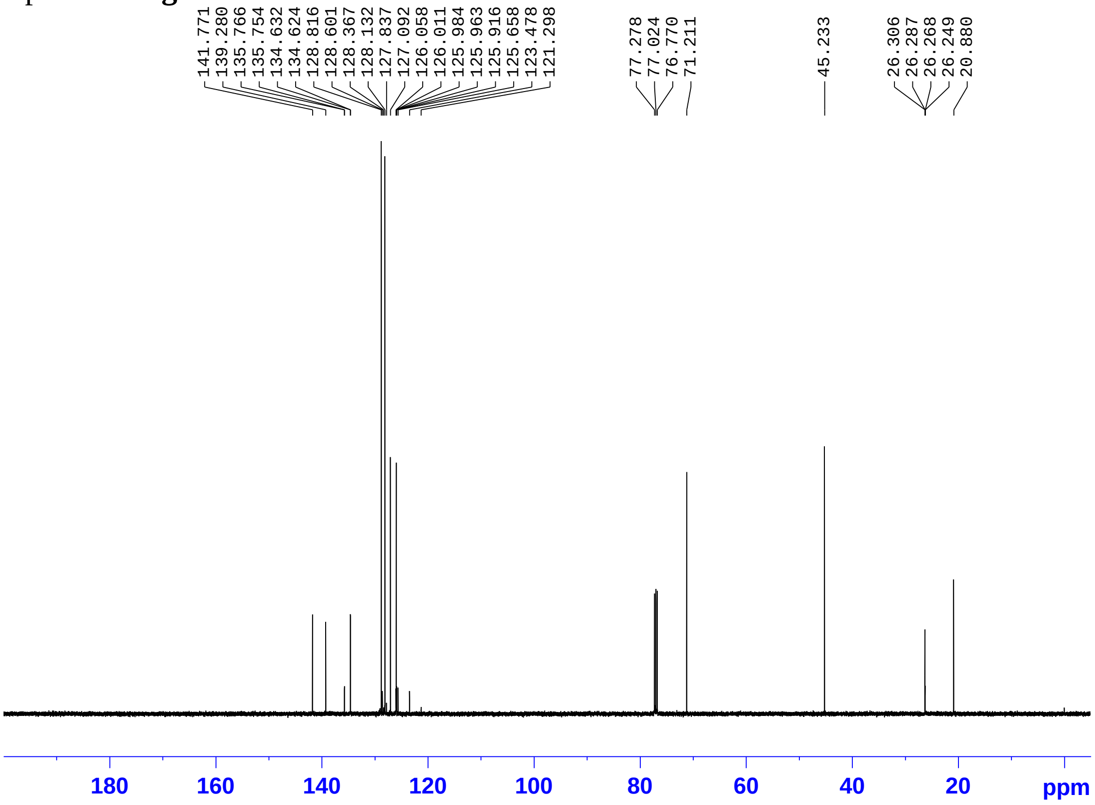

^{19}F NMR spectra of **5g**:

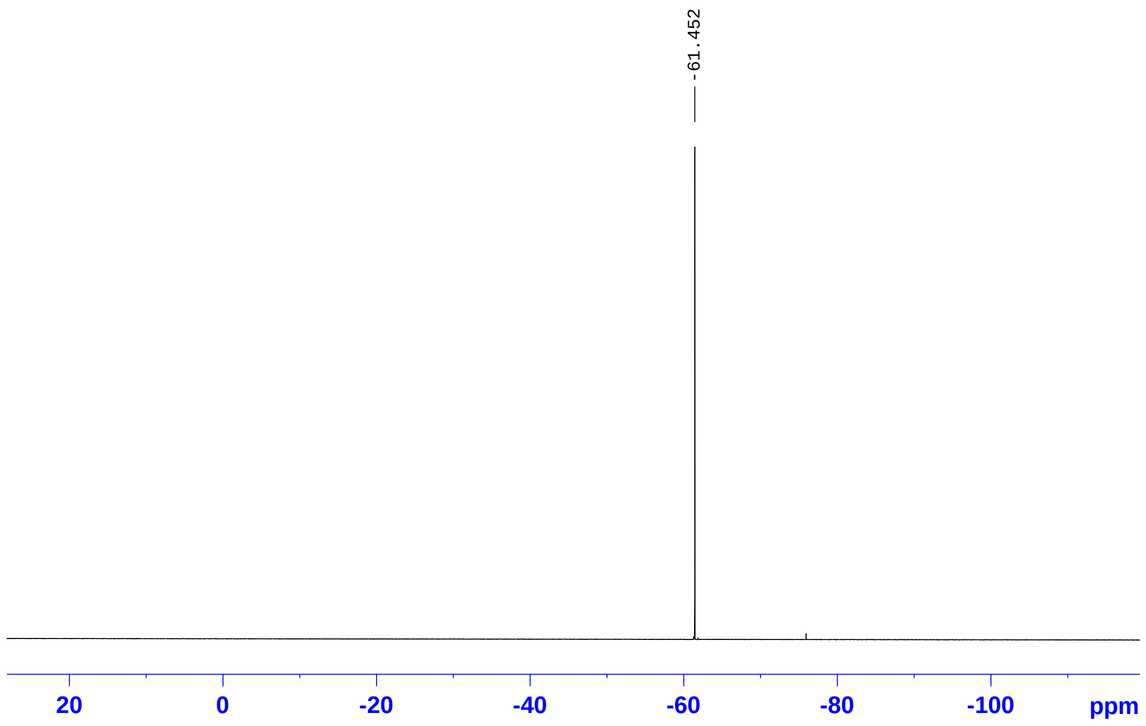
-61.452
20
0
-20
-40
-60
-80
-100
ppm

Chiral HPLC chromatogram of **5g**:

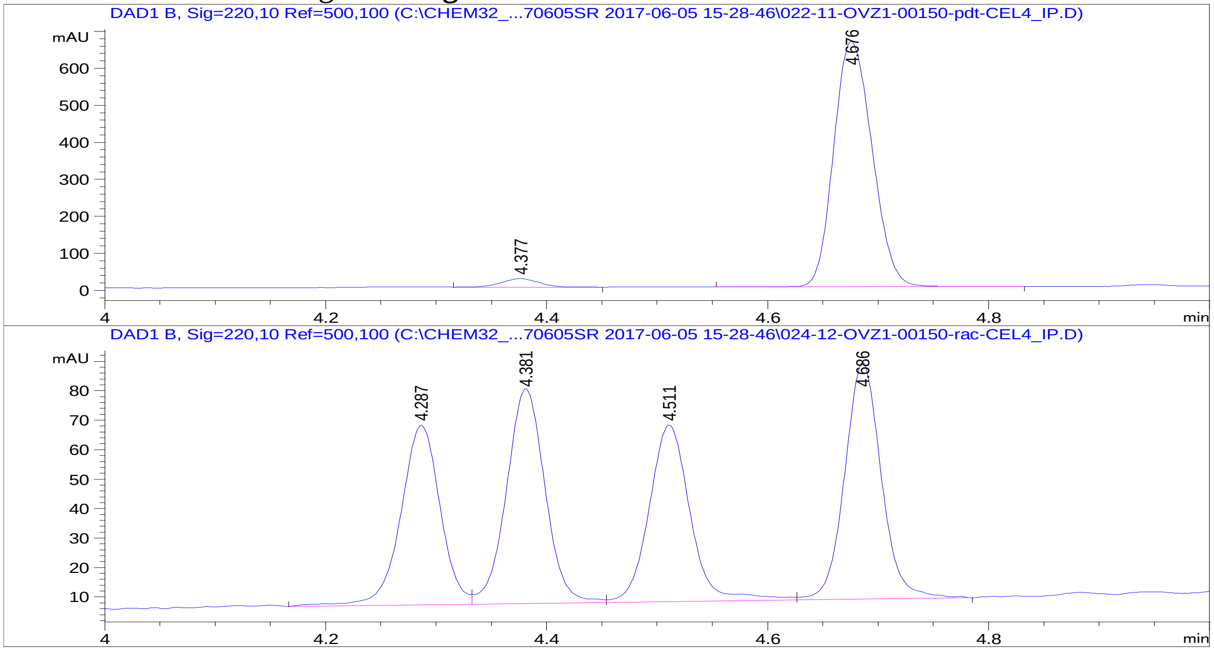

Integration results of **5g**:

Peak #	RetTime [min]	Type	Width [min]	Area [mAU*s]	Height [mAU]	Area %
1	4.377	BB	0.0369	53.93461	22.95625	3.2530
2	4.676	VB R	0.0387	1604.05469	663.29297	96.7470

Integration results of racemic sample:

Peak #	RetTime [min]	Type	Width [min]	Area [mAU*s]	Height [mAU]	Area %
1	4.287	BV	0.0379	151.18619	60.97048	22.6410
2	4.381	VV	0.0378	176.89326	72.93613	26.4908
3	4.511	VV R	0.0401	154.80957	59.97435	23.1837
4	4.686	VV R	0.0359	184.86415	80.26593	27.6845

^{1}H NMR spectra of **5h**:

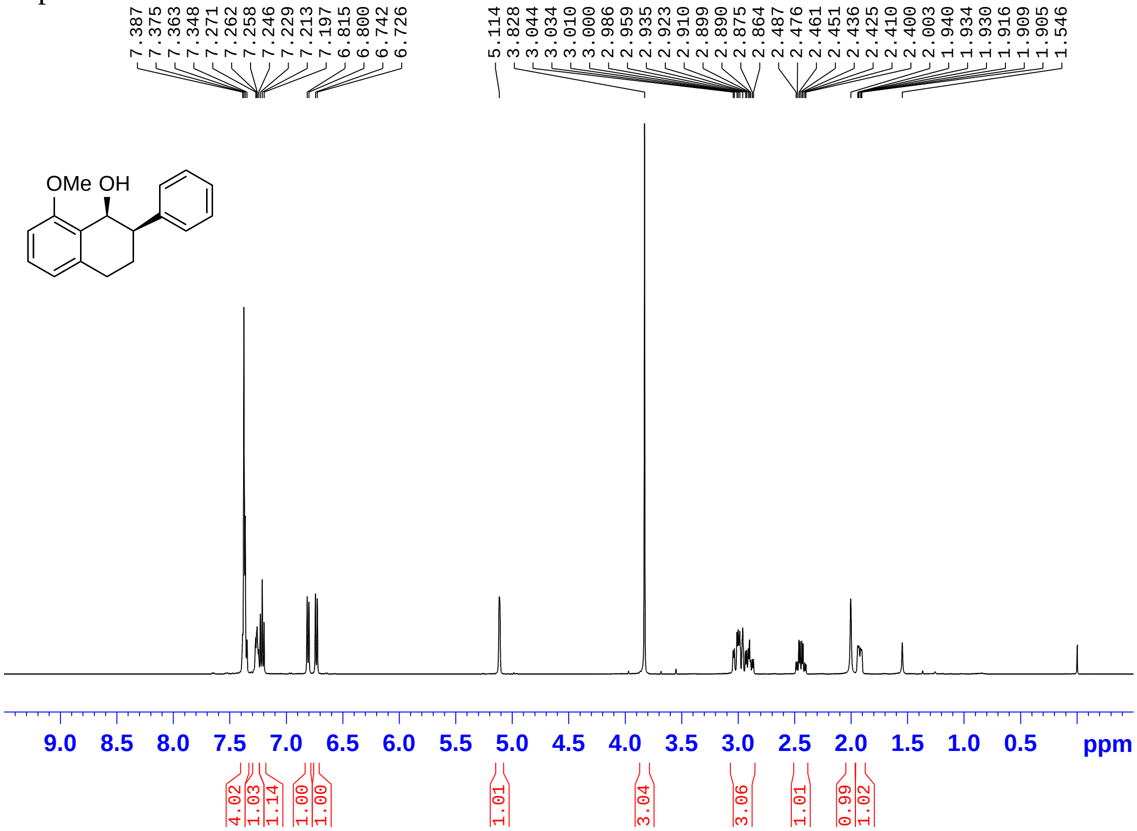

^{13}C NMR spectra of **5h**:

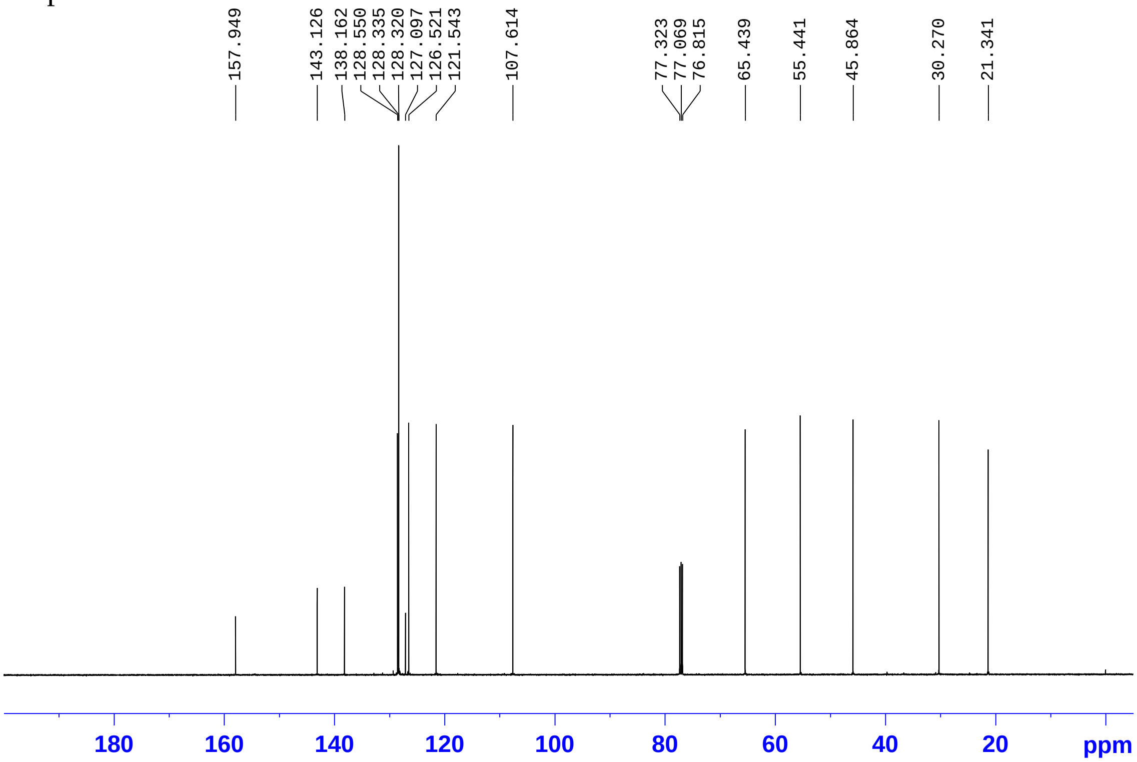

Chiral HPLC chromatogram of **5h**:

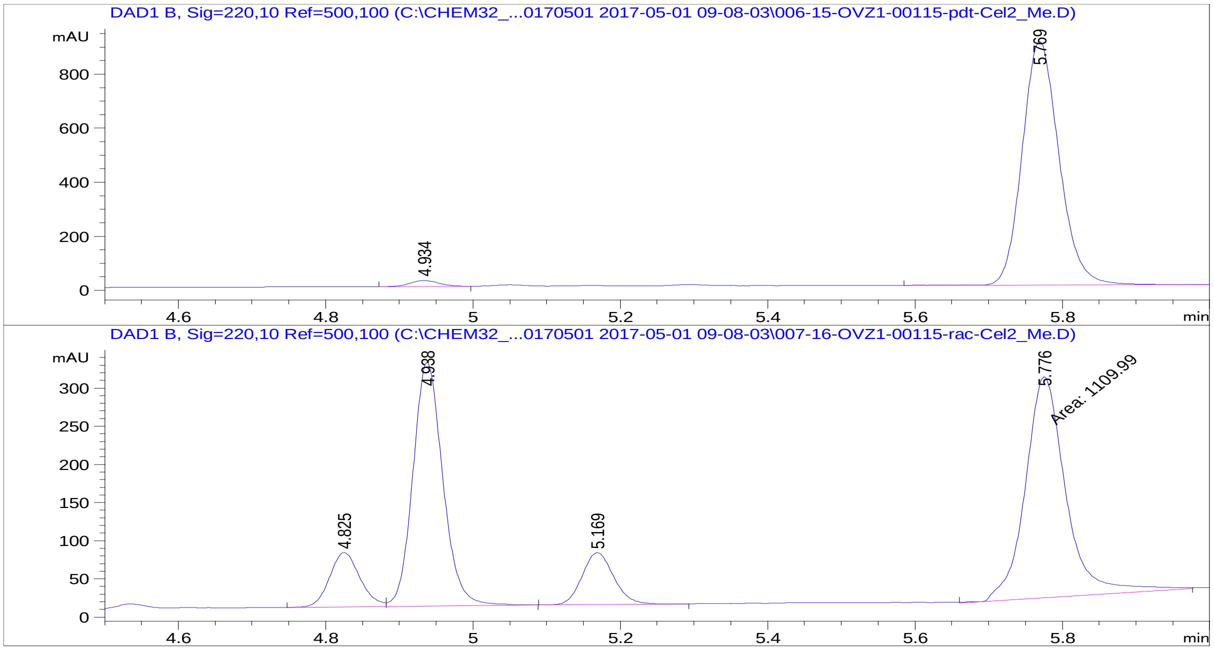

Integration results of **5h**:

Peak #	RetTime [min]	Type	Width [min]	Area [mAU*s]	Height [mAU]	Area %
1	4.934	BV	0.0424	61.89943	22.67056	1.9334
2	5.769	VV R	0.0536	3139.69214	902.41913	98.0666

Integration results of racemic sample:

Peak #	RetTime [min]	Type	Width [min]	Area [mAU*s]	Height [mAU]	Area %
1	4.825	BV	0.0440	204.35185	71.22705	8.4490
2	4.938	VV R	0.0429	900.47974	319.49039	37.2309
3	5.169	BV R	0.0465	203.81975	68.00600	8.4270
4	5.776	MM	0.0639	1109.98718	289.64865	45.8931

^{1}H NMR spectra of **5i**:

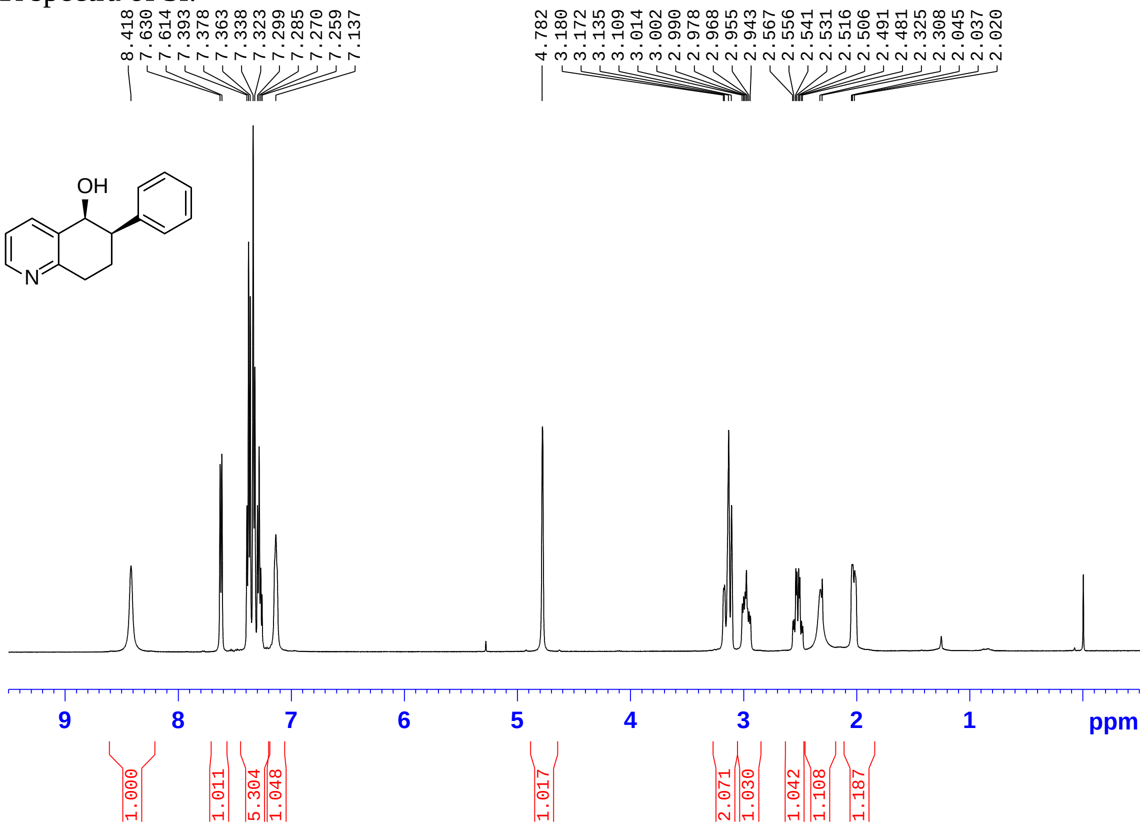

^{13}C NMRspectra of**5i**:

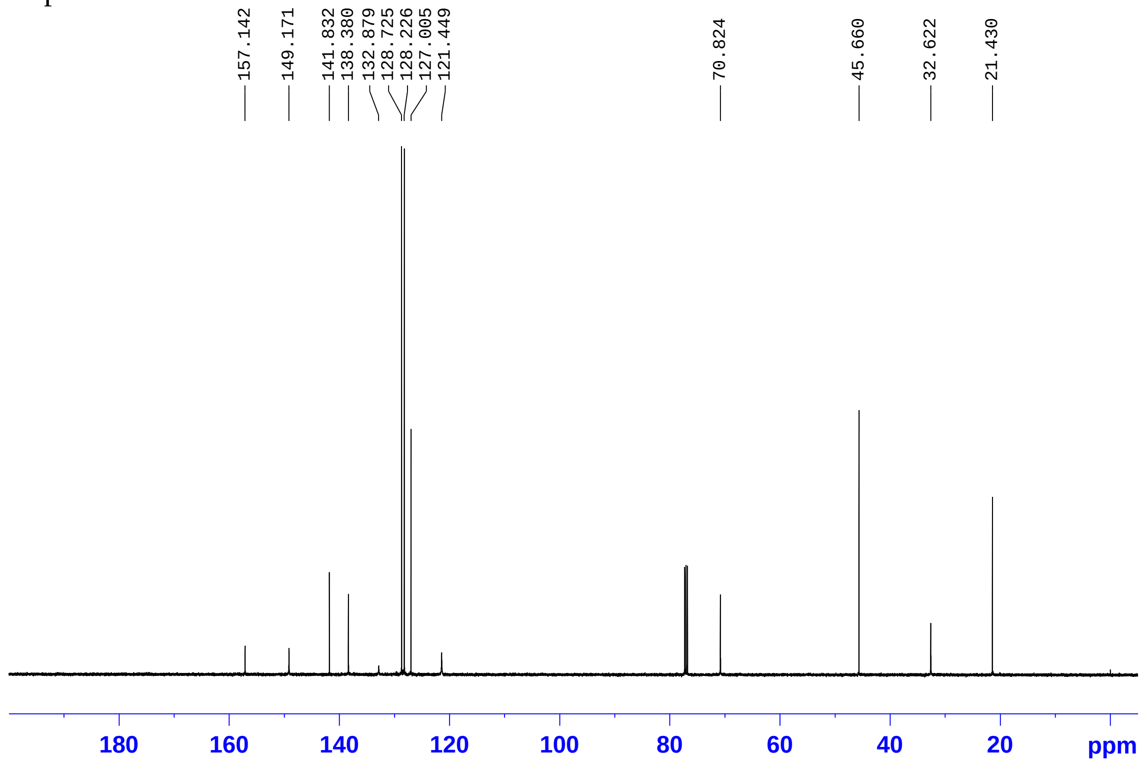

Chiral HPLC chromatogram of 5i:

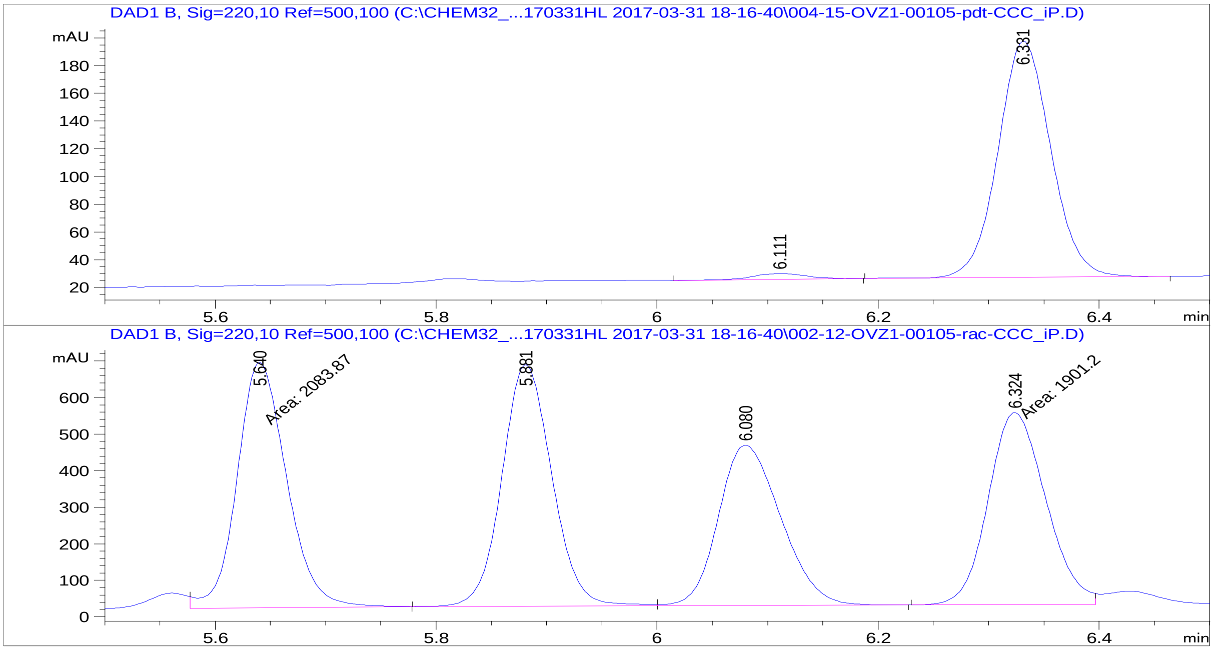

Integration results of 5i:

Peak #	RetTime [min]	Type	Width [min]	Area [mAU*s]	Height [mAU]	Area %
1	6.111	BV R	0.0495	14.56060	4.14607	2.5025
2	6.331	VV R	0.0513	567.28076	170.38843	97.4975

Integration results of racemic sample:

Peak #	RetTime [min]	Type	Width [min]	Area [mAU*s]	Height [mAU]	Area %
1	5.634	VB R	0.0490	2267.73779	677.10815	27.6588
2	5.878	BV	0.0501	2154.50073	668.93634	26.2777
3	6.076	VB	0.0626	1735.58203	434.11475	21.1683
4	6.325	BV R	0.0580	2041.16125	521.90222	24.8953

^{1}H NMR spectra of **5j**:

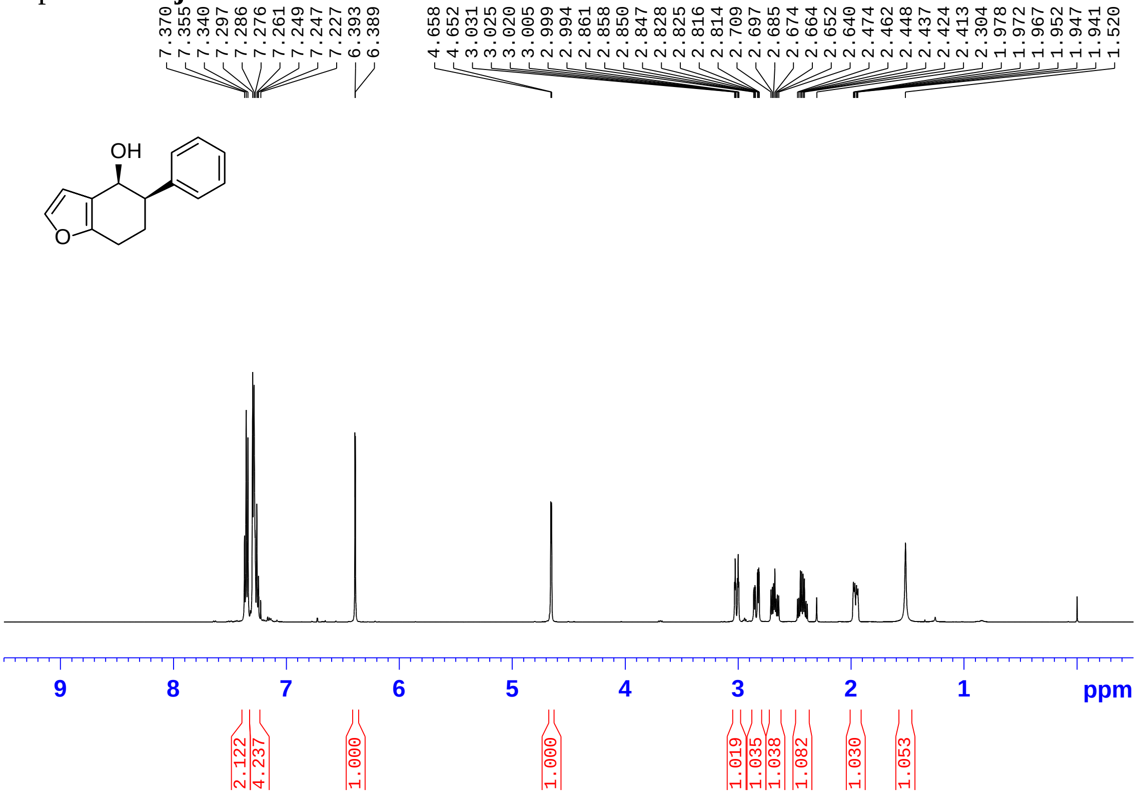

^{13}C NMR spectra of **5j**:

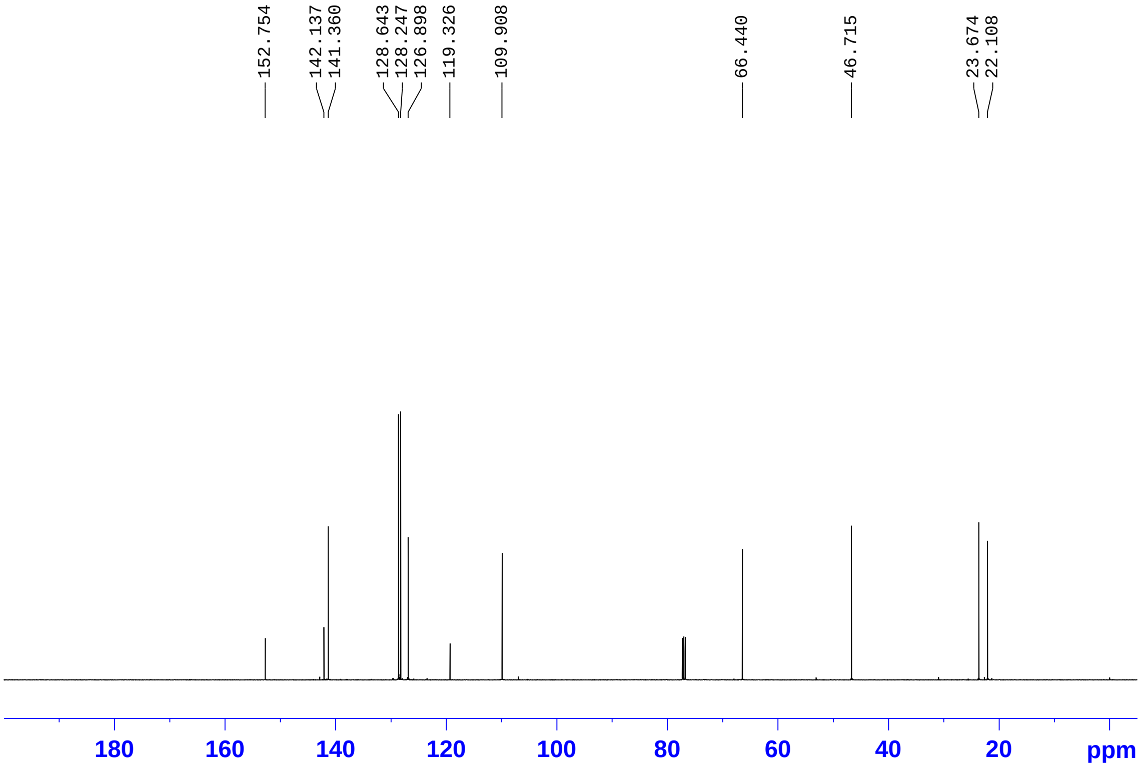

Chiral HPLC chromatogram of 5j:

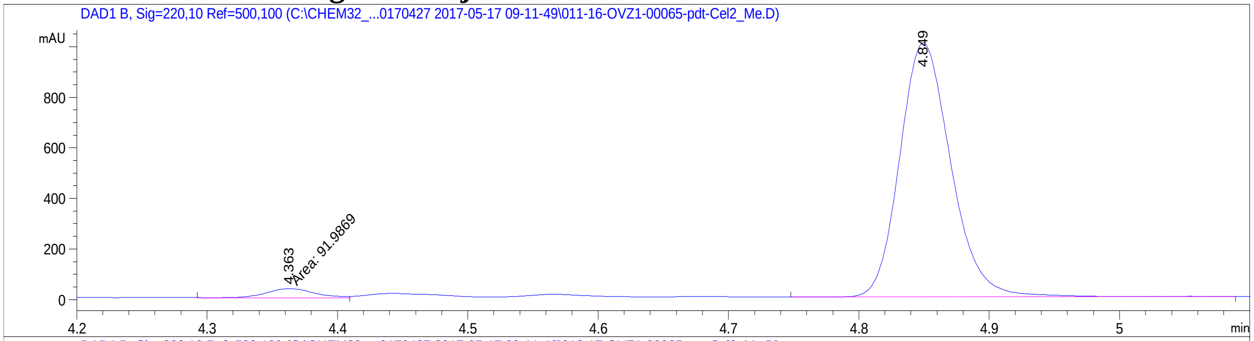

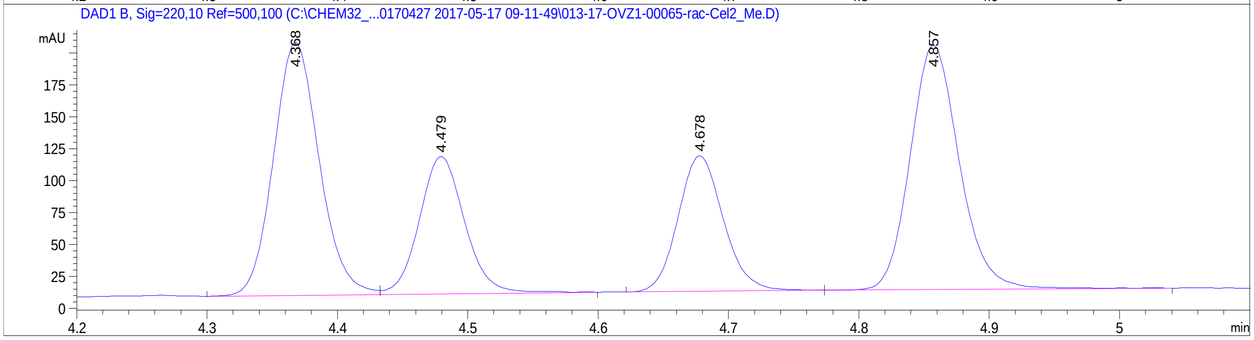

Integration results of 5j:

Peak #	RetTime [min]	Type	Width [min]	Area [mAU*s]	Height [mAU]	Area %
1	4.363	MF	0.0428	91.98691	35.84142	3.2520
2	4.849	BV R	0.0423	2736.67798	1005.24390	96.7480

Integration results of racemic sample:

Peak #	RetTime [min]	Type	Width [min]	Area [mAU*s]	Height [mAU]	Area %
1	4.368	BV	0.0375	482.77505	197.87148	32.1888
2	4.479	VV R	0.0372	259.70877	107.42774	17.3160
3	4.678	BB	0.0371	255.50360	106.06575	17.0356
4	4.857	BV R	0.0406	501.83496	191.41882	33.4596

^{1}**H NMR** spectra of **5k**:

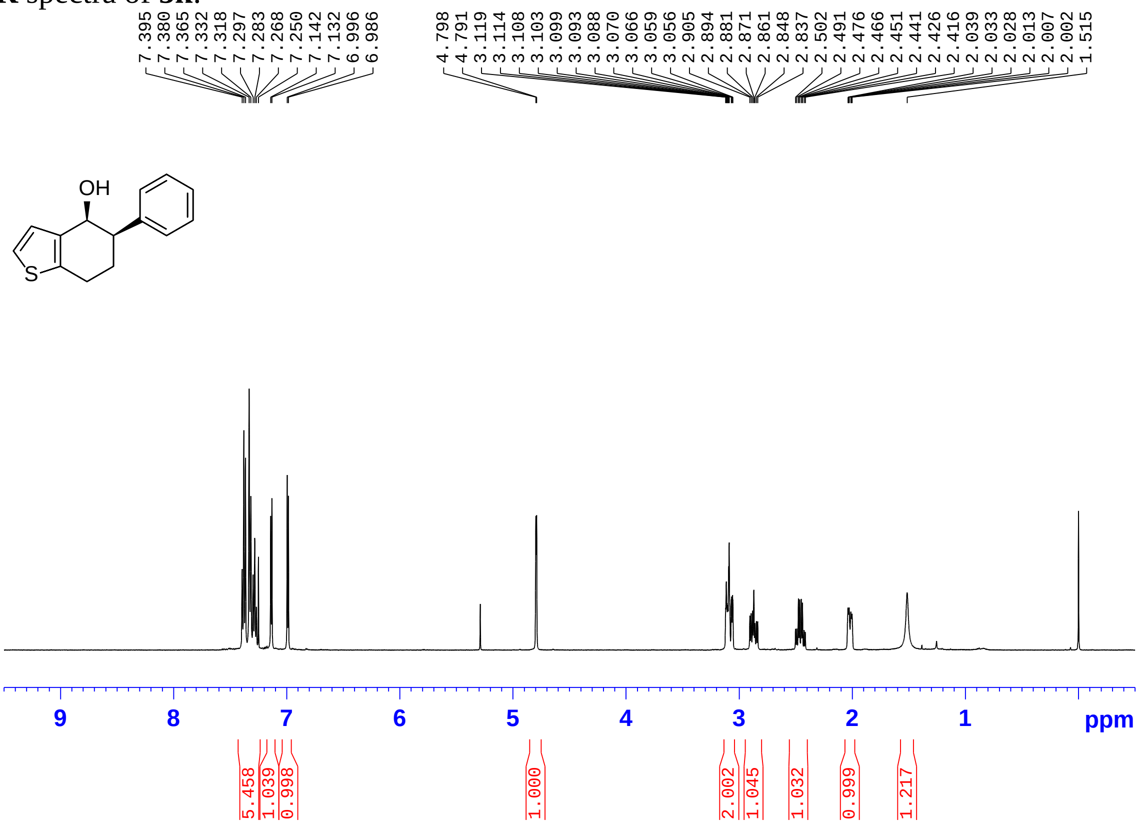

^{13}**C NMR** spectra of **5k**:

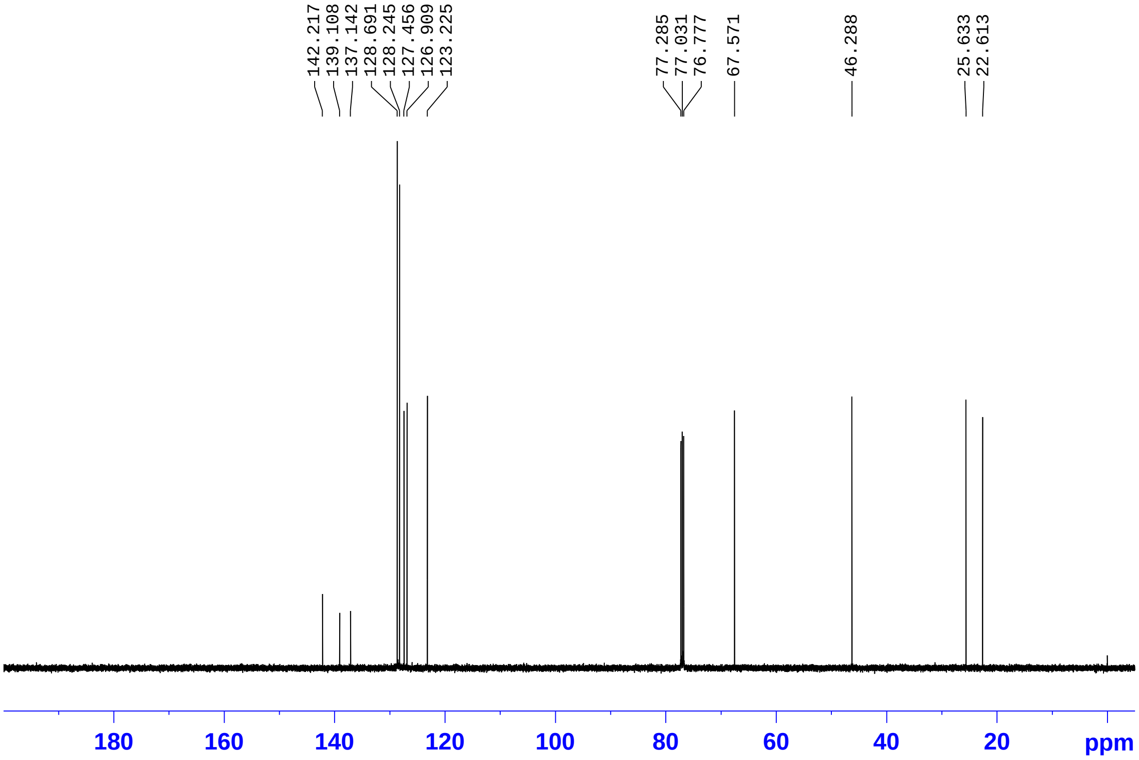

Chiral HPLC chromatogram of 5k:

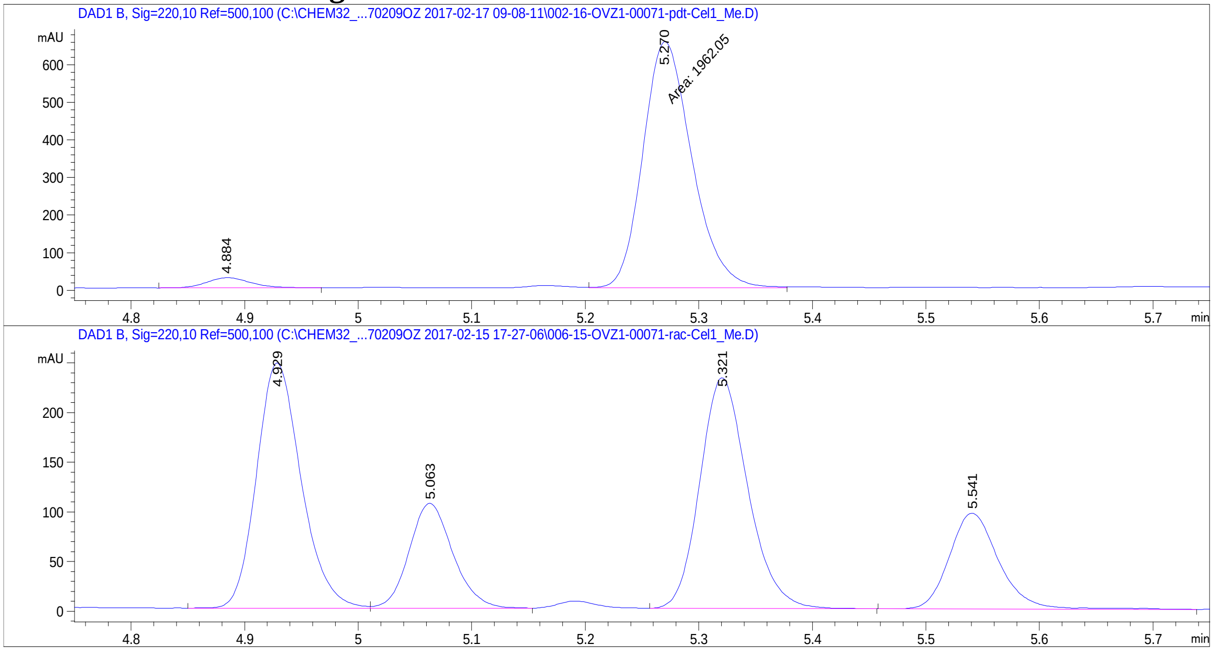

Integration results of 5k:

Peak #	RetTime [min]	Type	Width [min]	Area [mAU*s]	Height [mAU]	Area %
1	4.884	BB	0.0409	73.43101	27.25506	3.6075
2	5.270	MF	0.0499	1962.05078	654.94037	96.3925

Integration results of racemic sample:

Peak #	RetTime [min]	Type	Width [min]	Area [mAU*s]	Height [mAU]	Area %
1	4.929	BV	0.0423	673.57269	246.94211	34.9015
2	5.063	VB	0.0412	282.13770	105.54321	14.6191
3	5.321	BV R	0.0452	681.55988	232.24731	35.3153
4	5.541	VV R	0.0464	292.65717	96.51958	15.1642

^{1}**H NMR** spectra of **5l**:

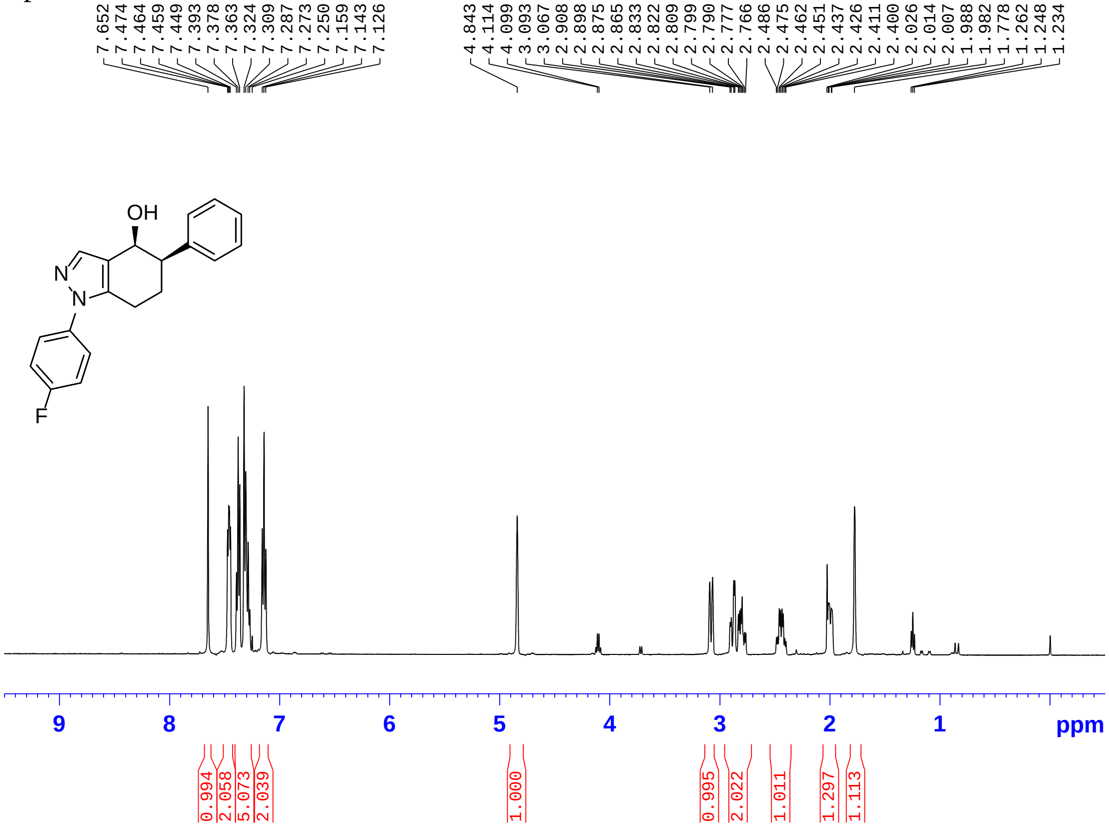

^{13}**C NMR** spectra of **5l**:

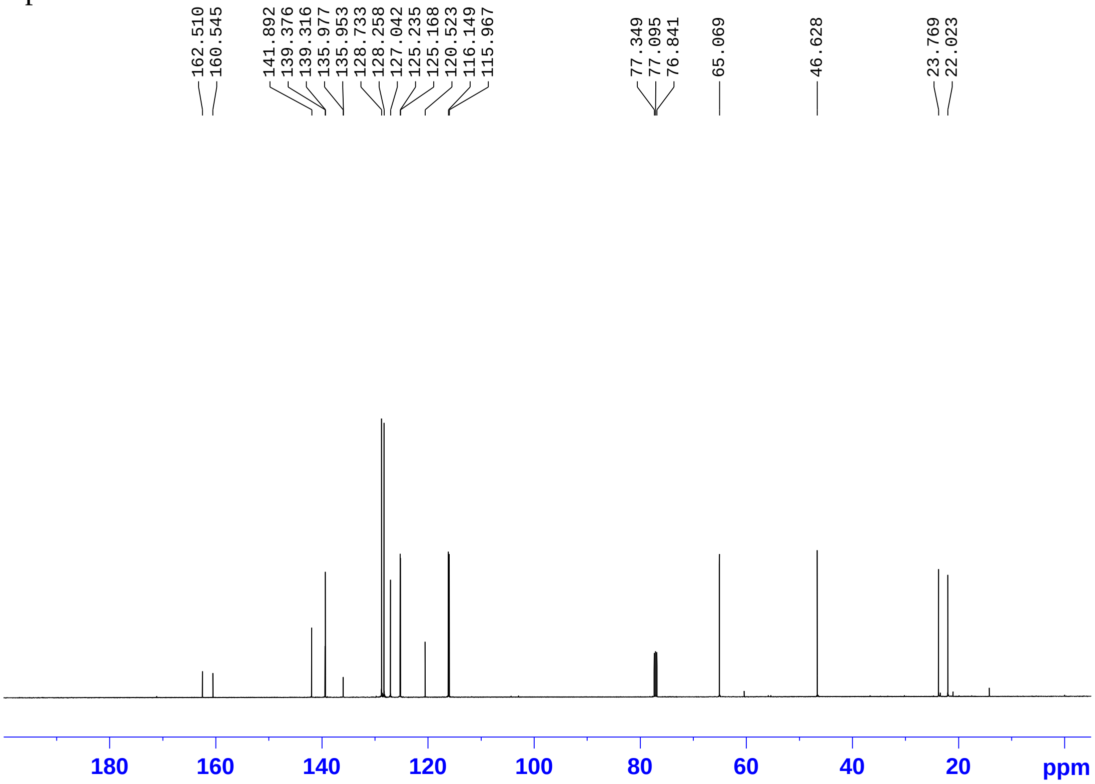

Chiral HPLC chromatogram of 5l:

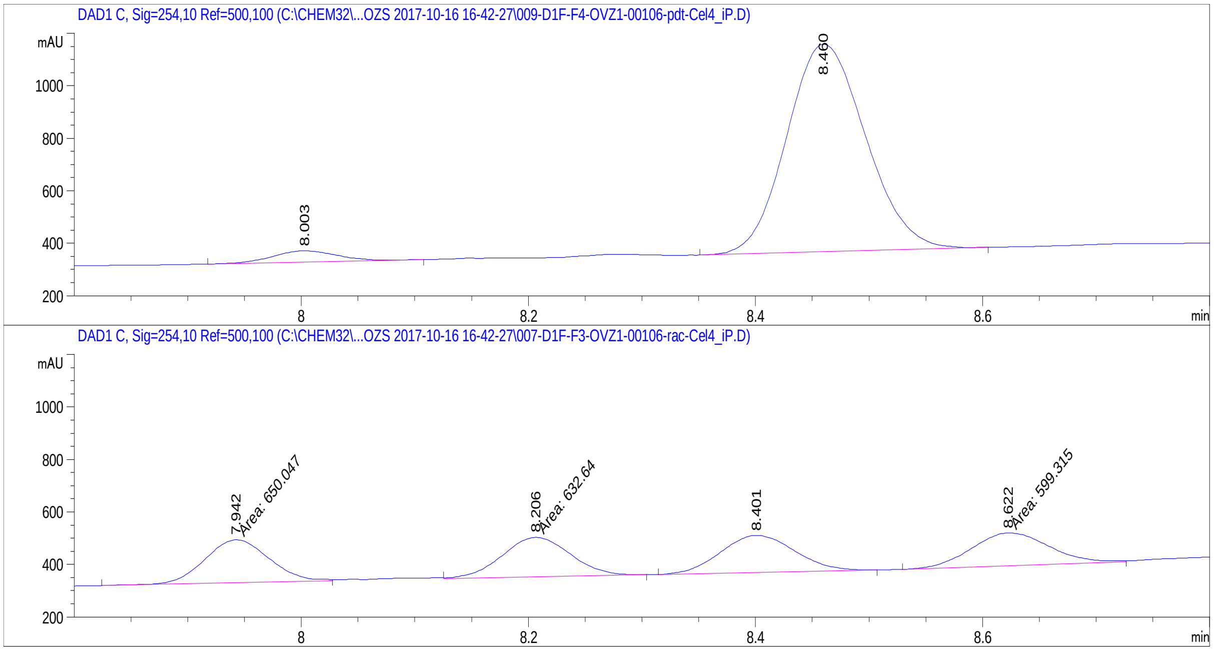

Integration results of 5l:

Peak #	RetTime [min]	Type	Width [min]	Area [mAU*s]	Height [mAU]	Area %
1	8.003	BV R	0.0639	173.32777	43.53936	4.3377
2	8.460	BV R	0.0748	3822.51221	791.87671	95.6623

Integration results of racemic sample:

Peak #	RetTime [min]	Type	Width [min]	Area [mAU*s]	Height [mAU]	Area %
1	7.942	MF	0.0659	650.04706	164.47346	25.7404
2	8.206	MM	0.0698	632.63965	151.09621	25.0511
3	8.401	BB	0.0714	643.39124	141.80176	25.4769
4	8.622	MF	0.0796	599.31488	125.44913	23.7316

^{1}H NMR spectra of **5m**:

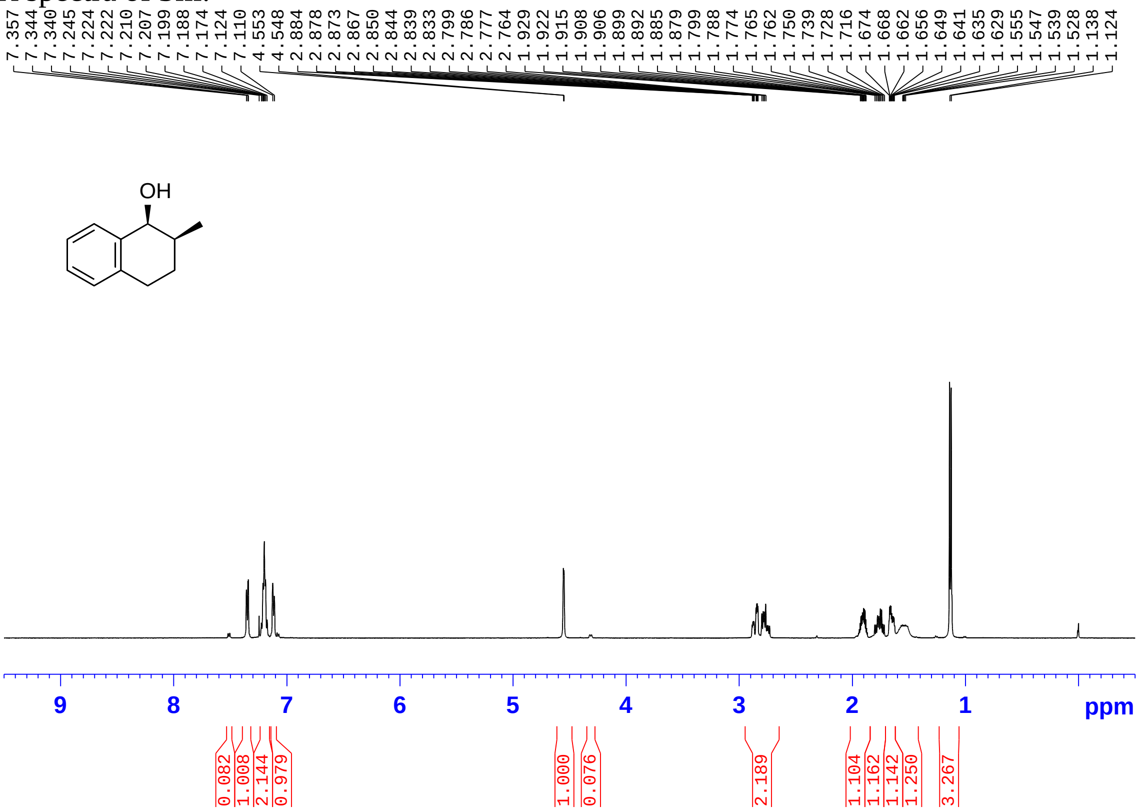

^{13}C NMR spectra of **5m**:

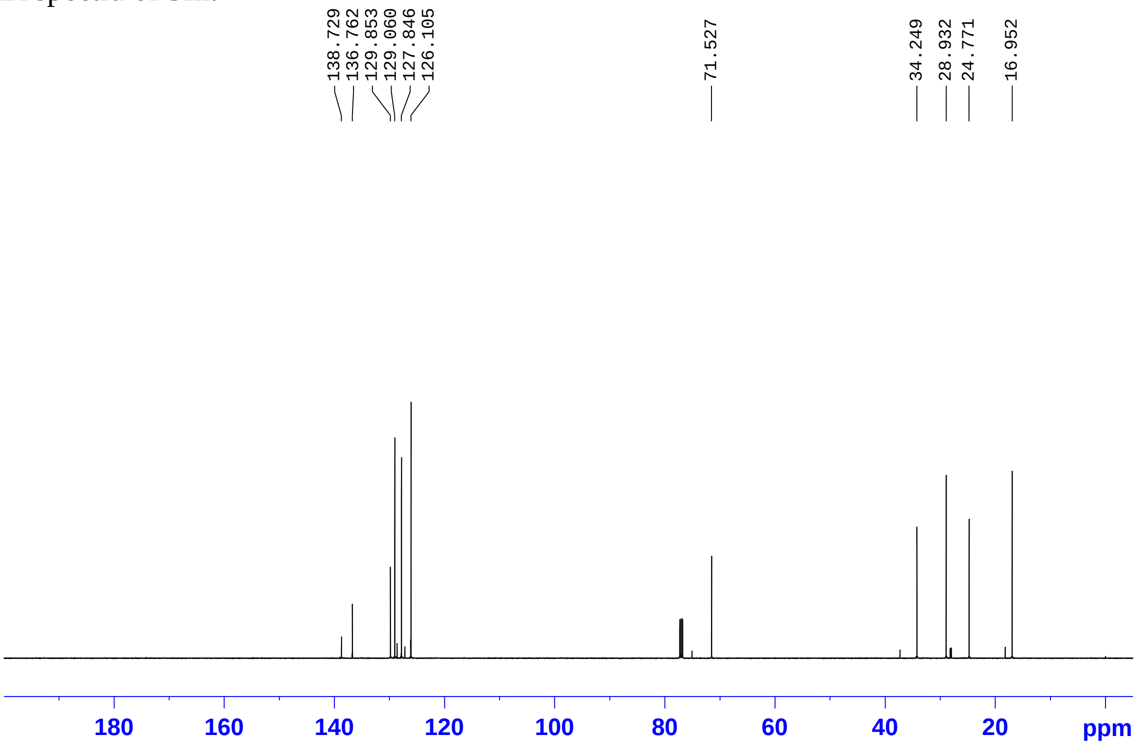

Chiral HPLC chromatogram of 5m:

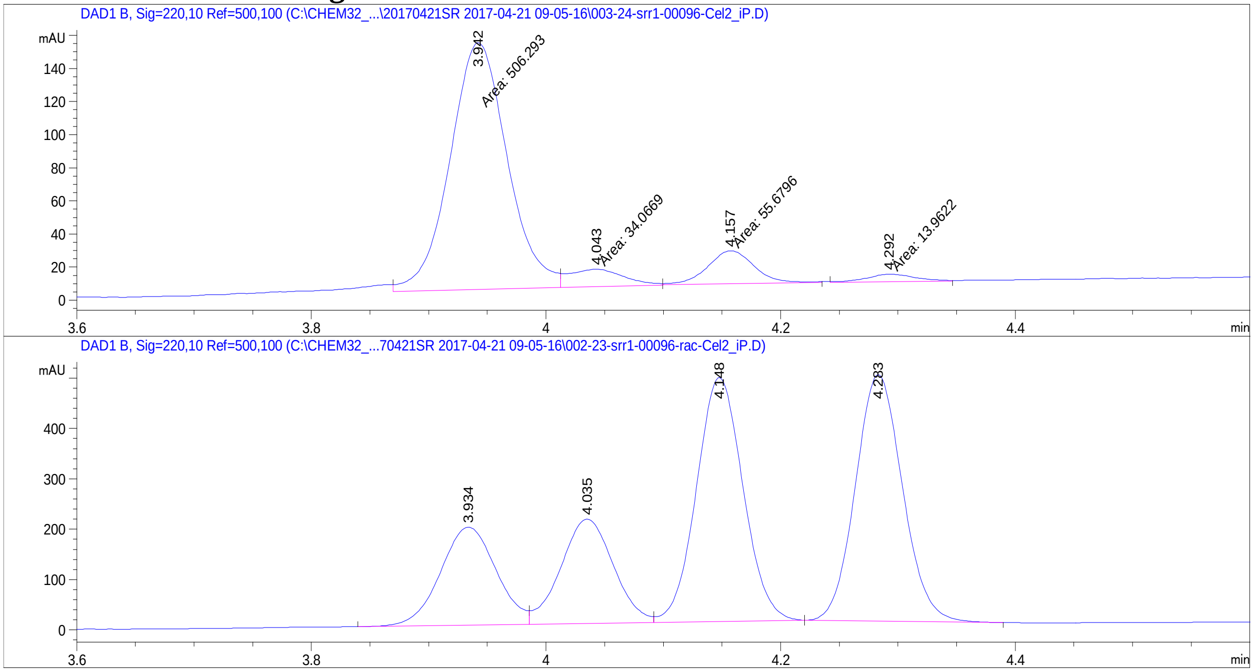

Integration results of 5m:

Peak #	RetTime [min]	Type	Width [min]	Area [mAU*s]	Height [mAU]	Area %
1	3.942	MF	0.0566	506.29269	149.06206	82.9986
2	4.043	FM	0.0533	34.06693	10.64416	5.5847
3	4.157	MM	0.0464	55.67962	19.98605	9.1278
4	4.292	MM	0.0498	13.96221	4.66979	2.2889

Integration results of racemic sample:

Peak #	RetTime [min]	Type	Width [min]	Area [mAU*s]	Height [mAU]	Area %
1	3.934	BV	0.0510	641.67706	194.59303	16.1159
2	4.035	VV	0.0483	642.88092	206.78848	16.1462
3	4.148	VB	0.0432	1335.01990	484.24341	33.5295
4	4.283	BB	0.0435	1362.05493	489.07391	34.2085

^{1}H NMR spectra of **5n**:

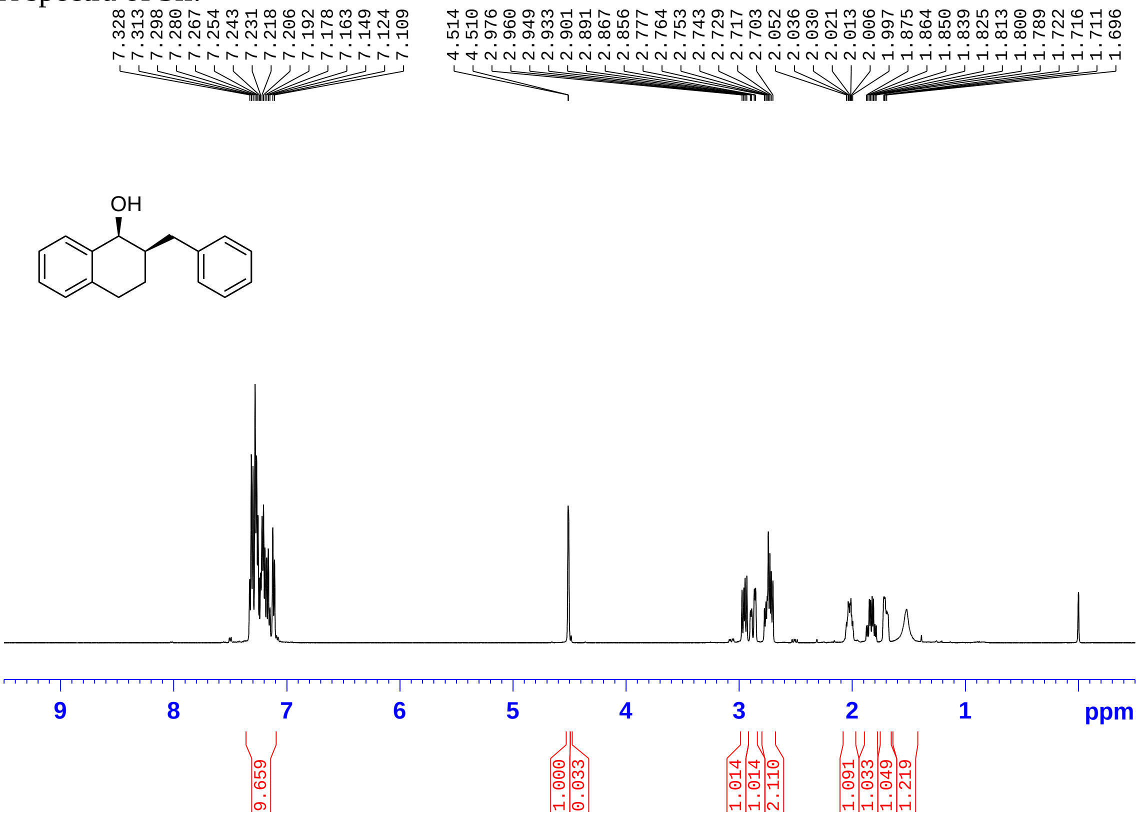

^{13}C NMR spectra of **5n**:

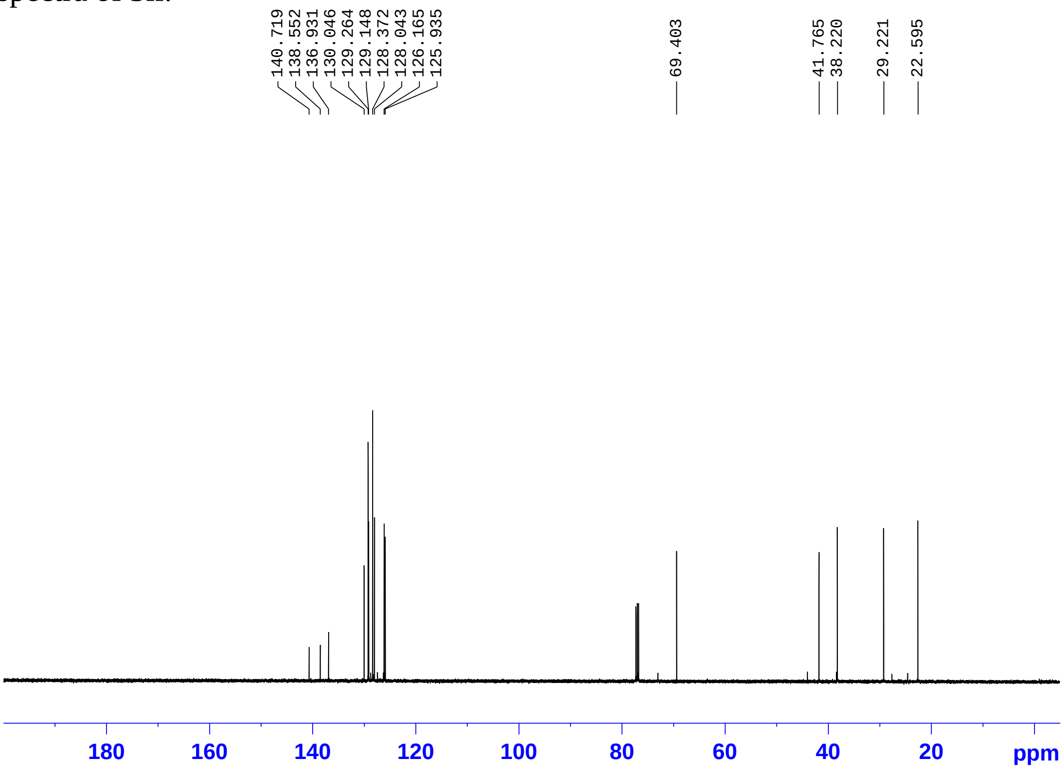

Chiral HPLC chromatogram of 5n:

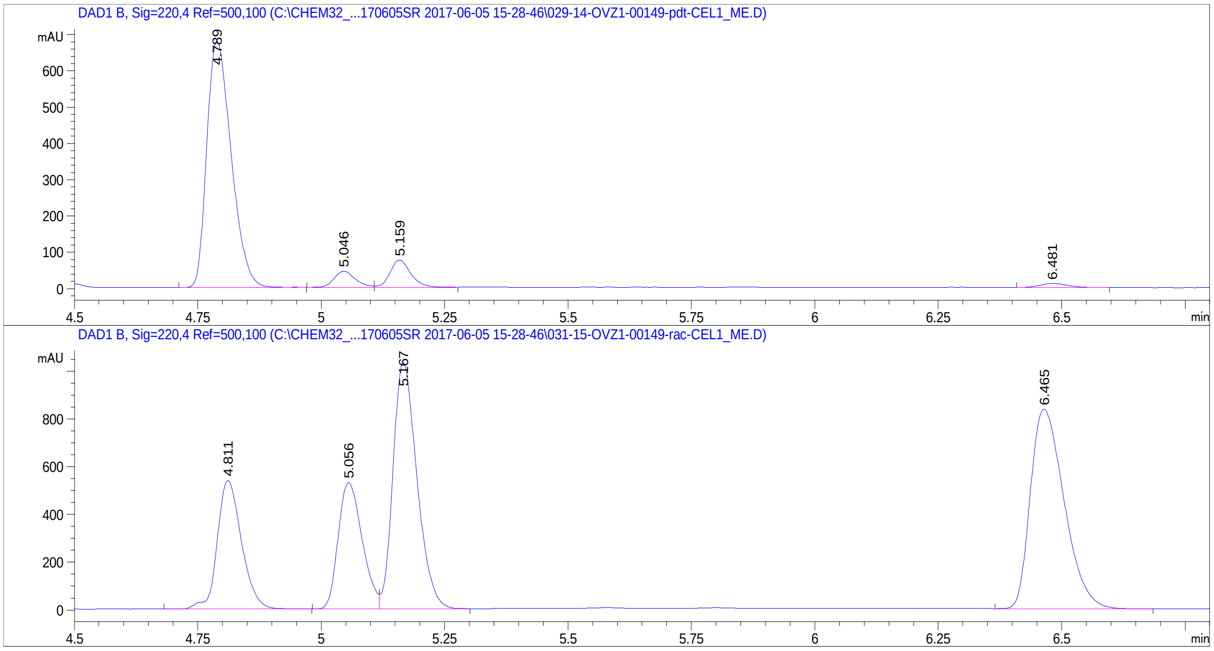

Integration results of 5n:

Peak #	RetTime [min]	Type	Width [min]	Area [mAU*s]	Height [mAU]	Area %
1	4.789	BV R	0.0540	2328.05664	678.69550	85.4845
2	5.046	BV	0.0449	132.14410	44.14441	4.8522
3	5.159	VB	0.0442	218.14244	75.53025	8.0100
4	6.481	BB	0.0524	45.02358	11.31267	1.6532

Integration results of racemic sample:

Peak #	RetTime [min]	Type	Width [min]	Area [mAU*s]	Height [mAU]	Area %
1	4.811	VV R	0.0522	1832.01831	538.08551	16.5666
2	5.056	BV	0.0528	1783.92419	528.95483	16.1317
3	5.167	VB	0.0523	3473.44312	1030.43298	31.4098
4	6.465	BB	0.0749	3969.09106	835.41168	35.8918

^{1}H NMR spectra of **5o**:

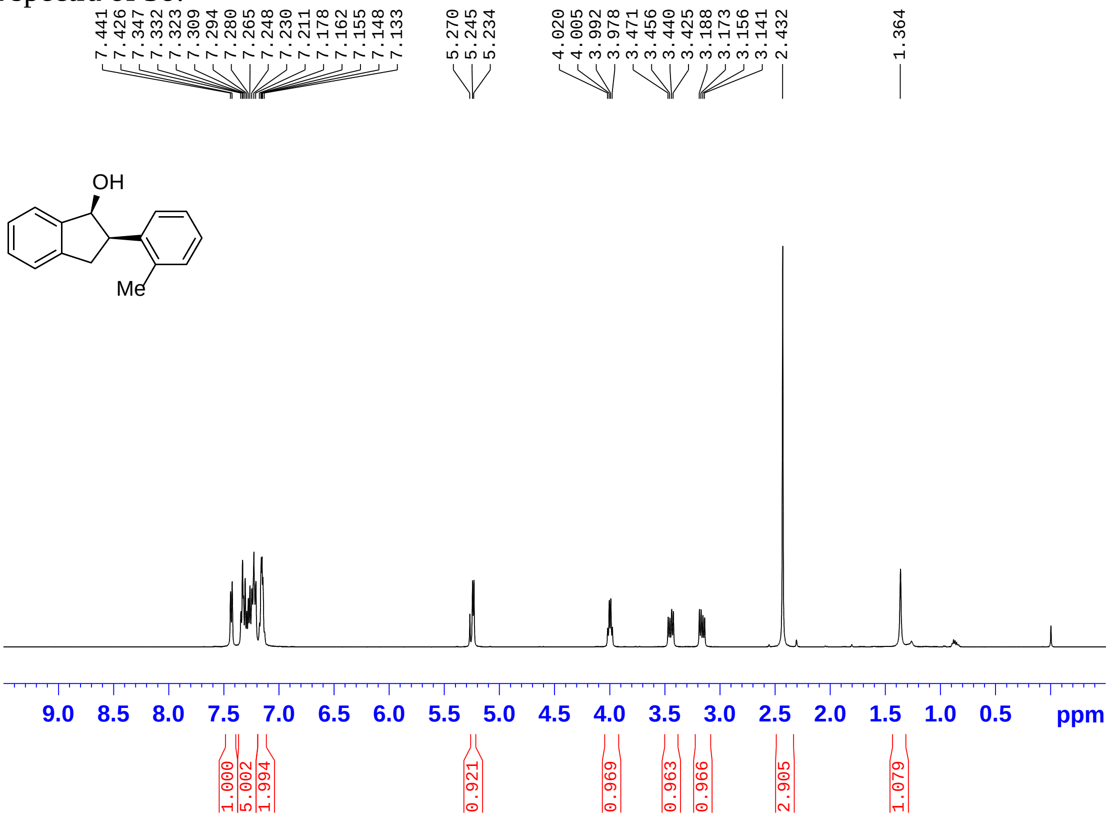

^{13}C NMR spectra of **5o**:

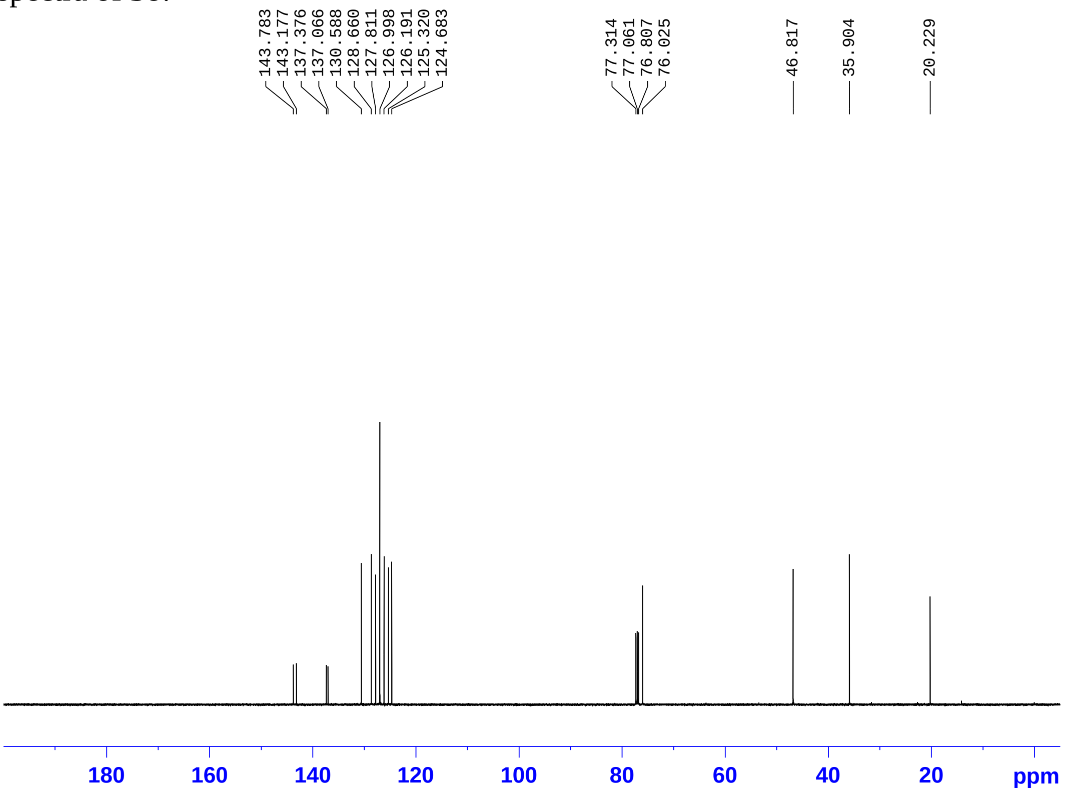

Chiral HPLC chromatogram of 5o:

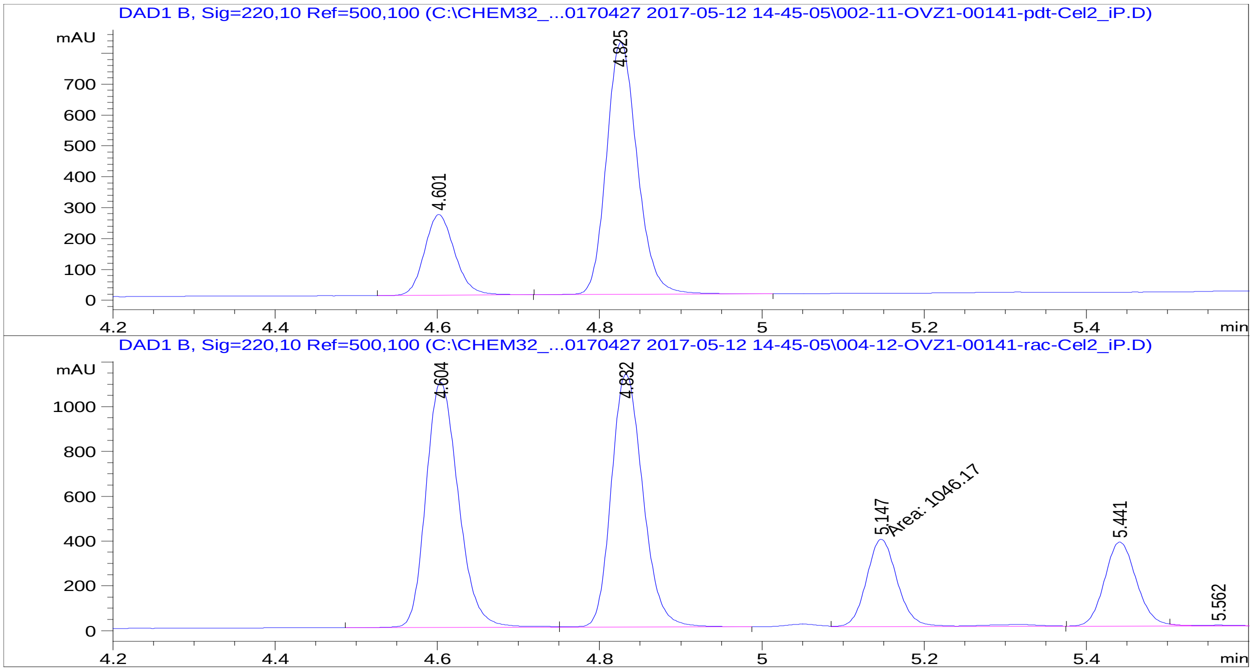

Integration results of 5o:

Peak #	RetTime [min]	Type	Width [min]	Area [mAU*s]	Height [mAU]	Area %
1	4.601	BB	0.0397	676.48572	260.91144	23.9063
2	4.825	BV R	0.0408	2153.25830	816.17120	76.0937

Peak #	RetTime [min]	Type	Width [min]	Area [mAU*s]	Height [mAU]	Area %
1	4.604	BV	0.0434	3042.49170	1095.88232	37.1966
2	4.832	VB	0.0425	3053.40210	1130.20178	37.3300
3	5.147	FM	0.0448	1046.16943	389.11877	12.7902
4	5.441	BV R	0.0430	1028.19458	374.68881	12.5704

^{1}**H NMR** spectra of **5p**:

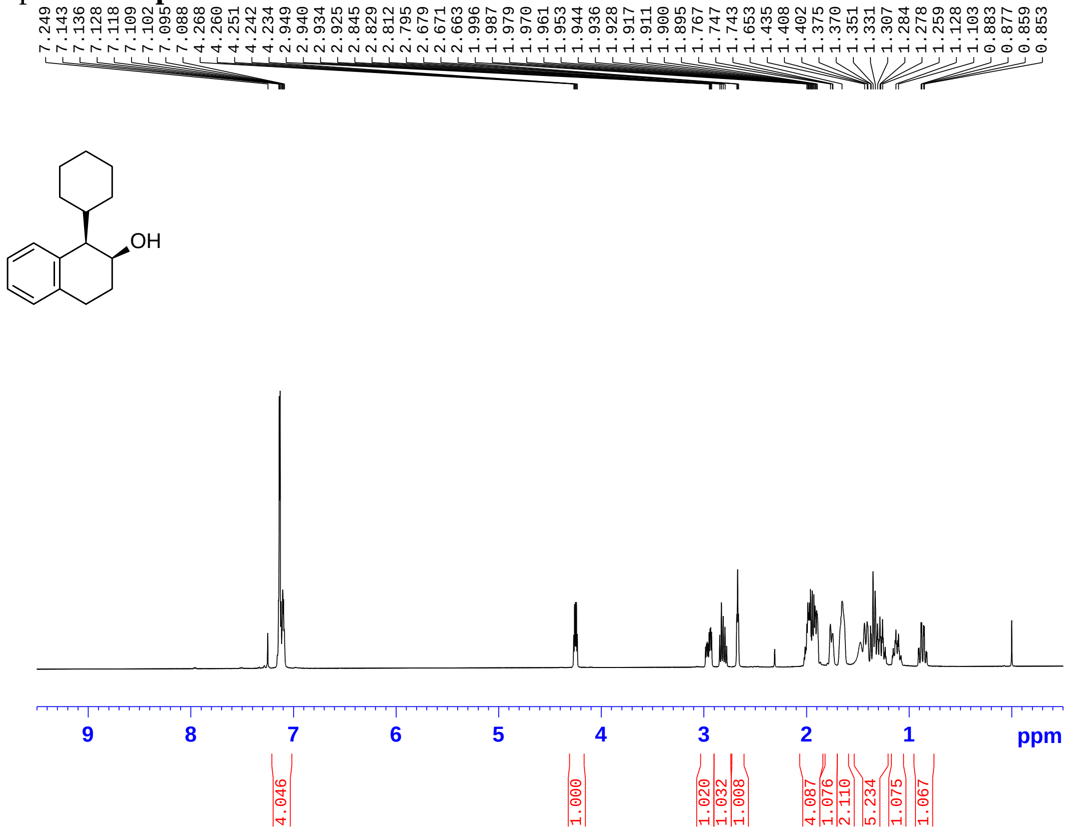

^{13}**C NMR** spectra of **5p**:

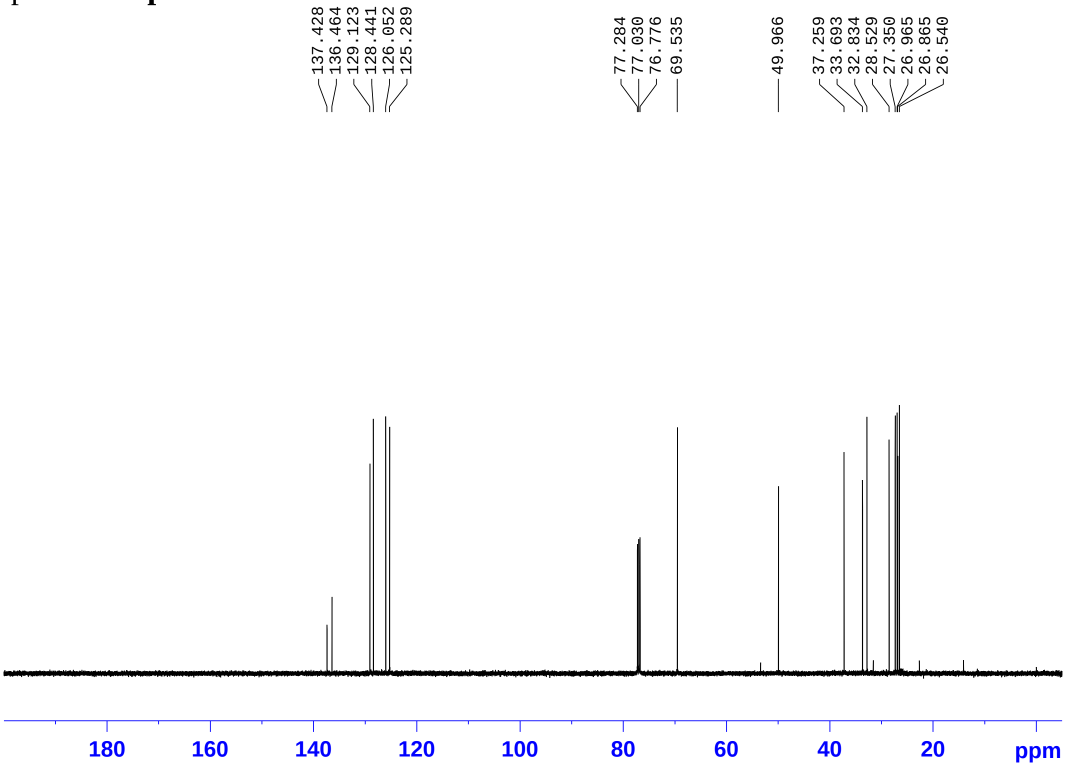

Chiral HPLC chromatogram of 5p:

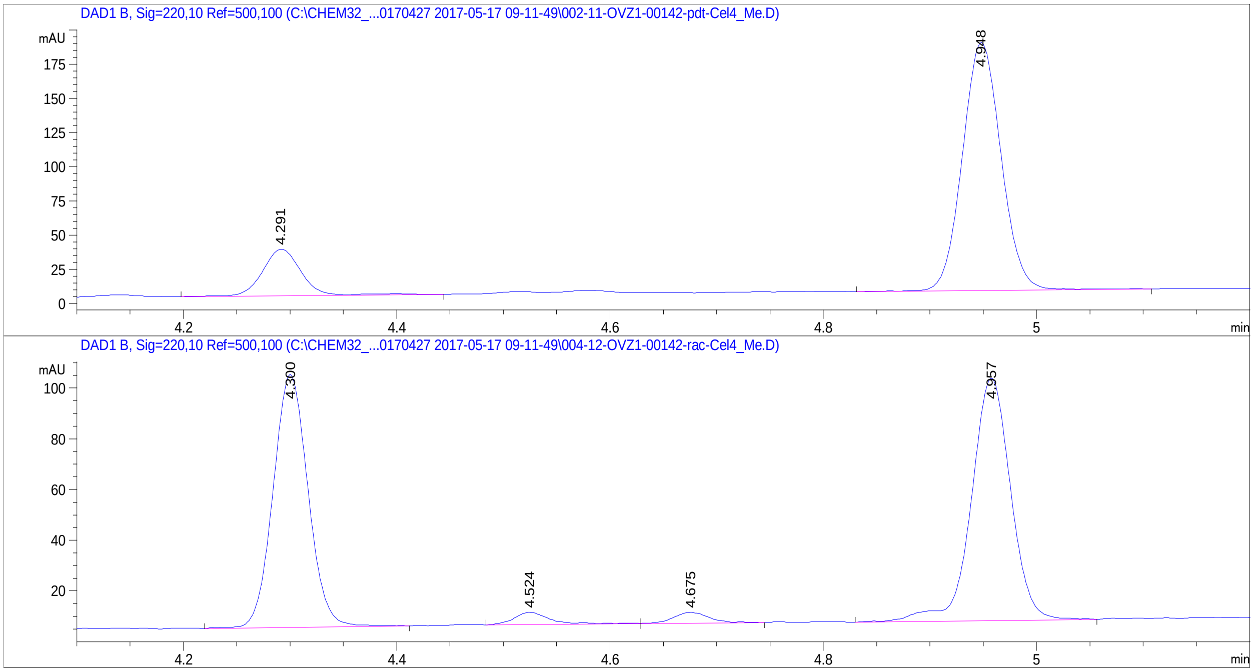

Integration results of 5p:

Peak #	RetTime [min]	Type	Width [min]	Area [mAU*s]	Height [mAU]	Area %
1	4.291	VV R	0.0390	85.88157	33.99078	15.8387
2	4.948	VV R	0.0394	456.34531	181.28902	84.1613

Integration results of racemic sample:

Peak #	RetTime [min]	Type	Width [min]	Area [mAU*s]	Height [mAU]	Area %
1	4.300	VV R	0.0350	226.42995	99.71259	45.9004
2	4.524	BV R	0.0337	10.89894	4.76253	2.2094
3	4.675	BV R	0.0376	9.89295	4.26256	2.0054
4	4.957	BB	0.0400	246.08511	95.59235	49.8848

^{1}H NMR spectra of **5q**:

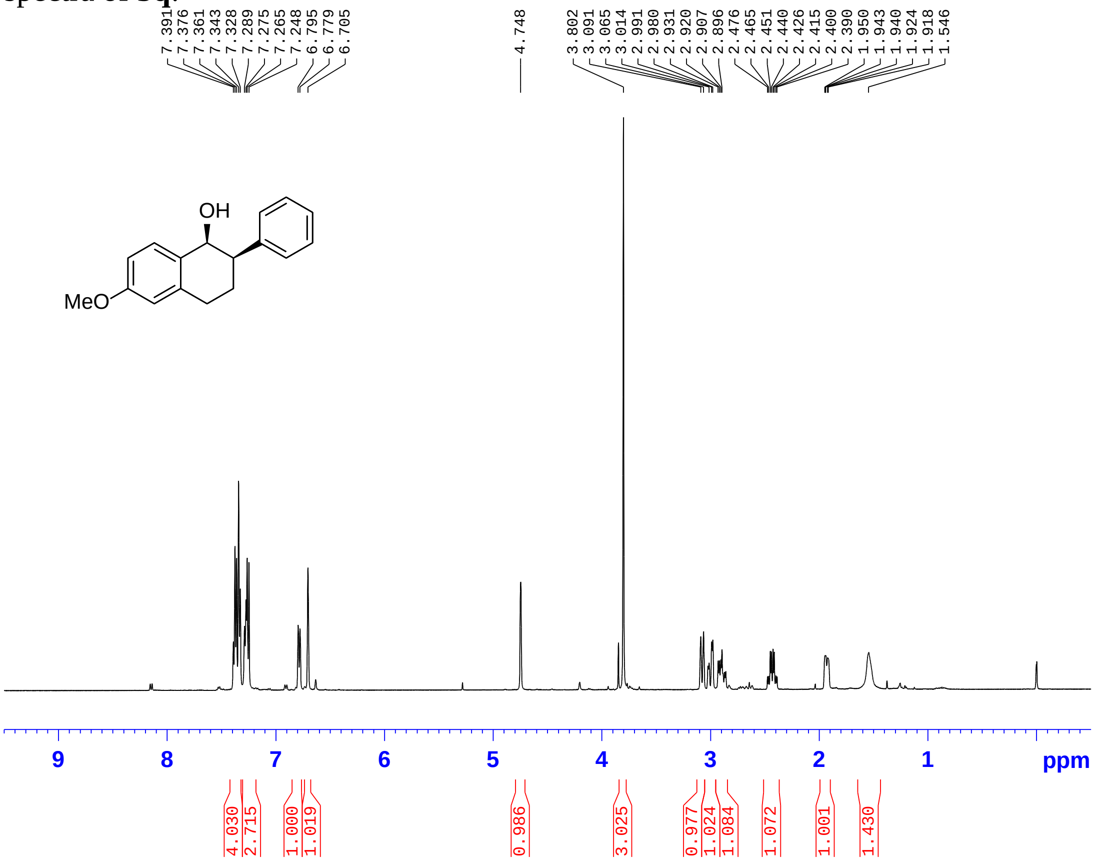

^{13}C NMR spectra of **5q:**

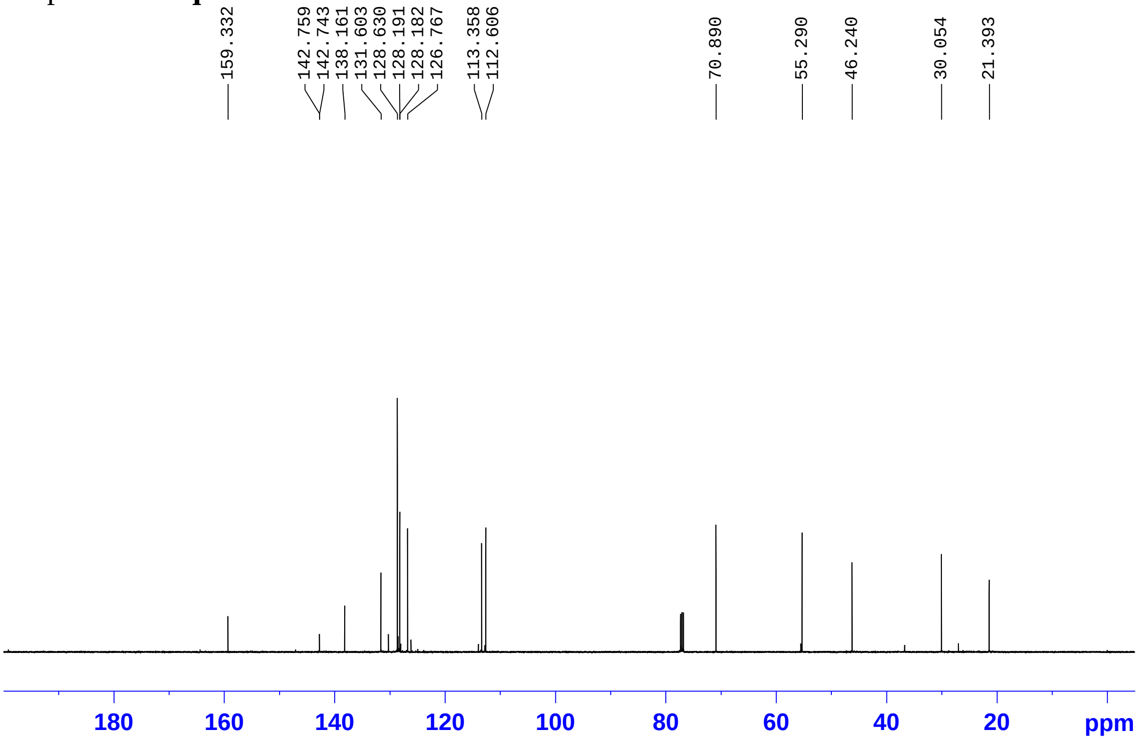

Chiral HPLC chromatogram of **5q:**

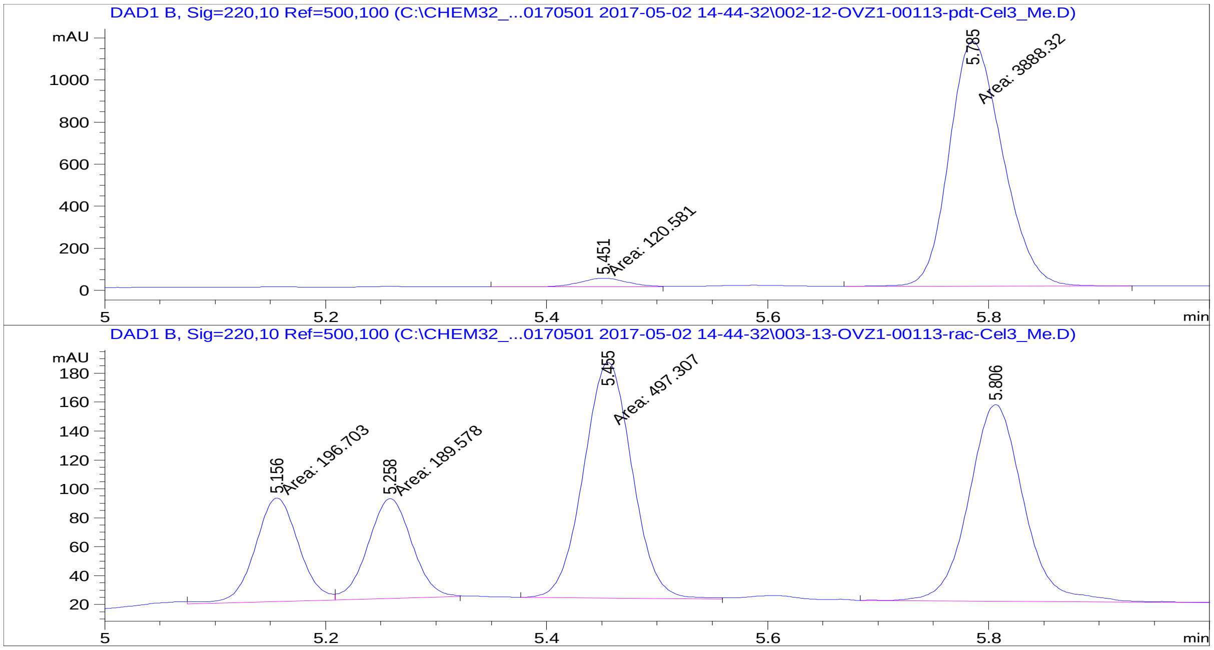

Integration results of **5q:**

Peak #	RetTime [min]	Type	Width [min]	Area [mAU*s]	Height [mAU]	Area %
1	5.451	MF	0.0494	120.58064	40.69883	3.0078
2	5.785	MM	0.0556	3888.32178	1164.60706	96.9922

Integration results of racemic sample:

Peak #	RetTime [min]	Type	Width [min]	Area [mAU*s]	Height [mAU]	Area %
1	5.156	MF	0.0456	196.70259	71.86555	14.5405
2	5.258	FM	0.0458	189.57776	68.97196	14.0138
3	5.455	MF	0.0508	497.30704	163.15952	36.7615
4	5.806	VV R	0.0532	469.20645	136.07162	34.6843

^{1}H NMR spectra of **6:**

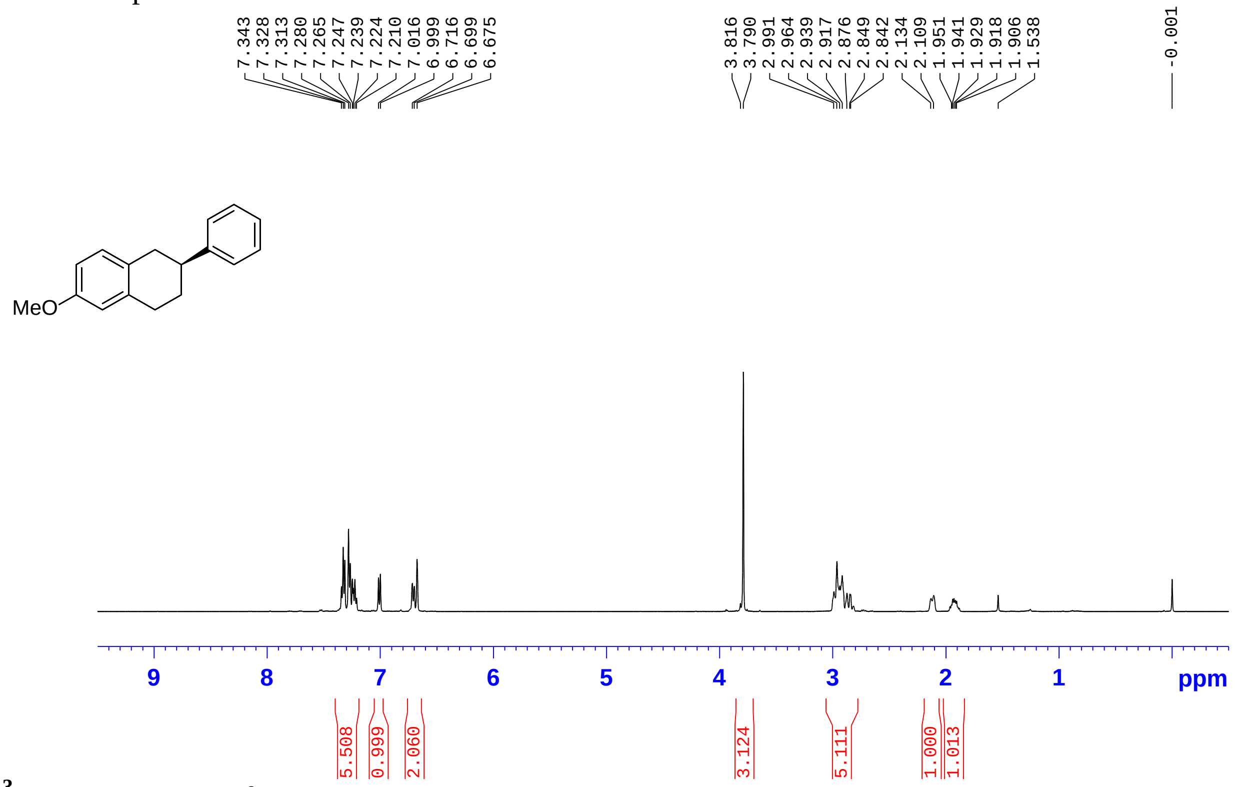

^{13}C NMR spectra of **6:**

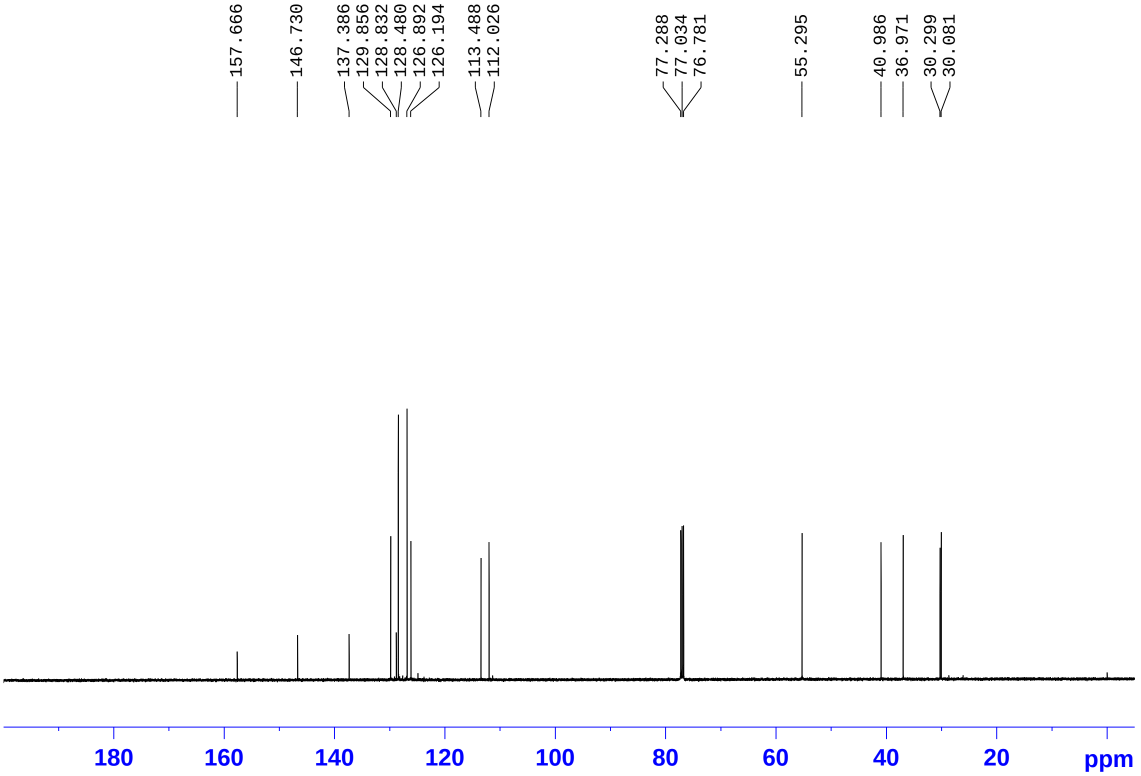

Chiral HPLC chromatogram of 6:

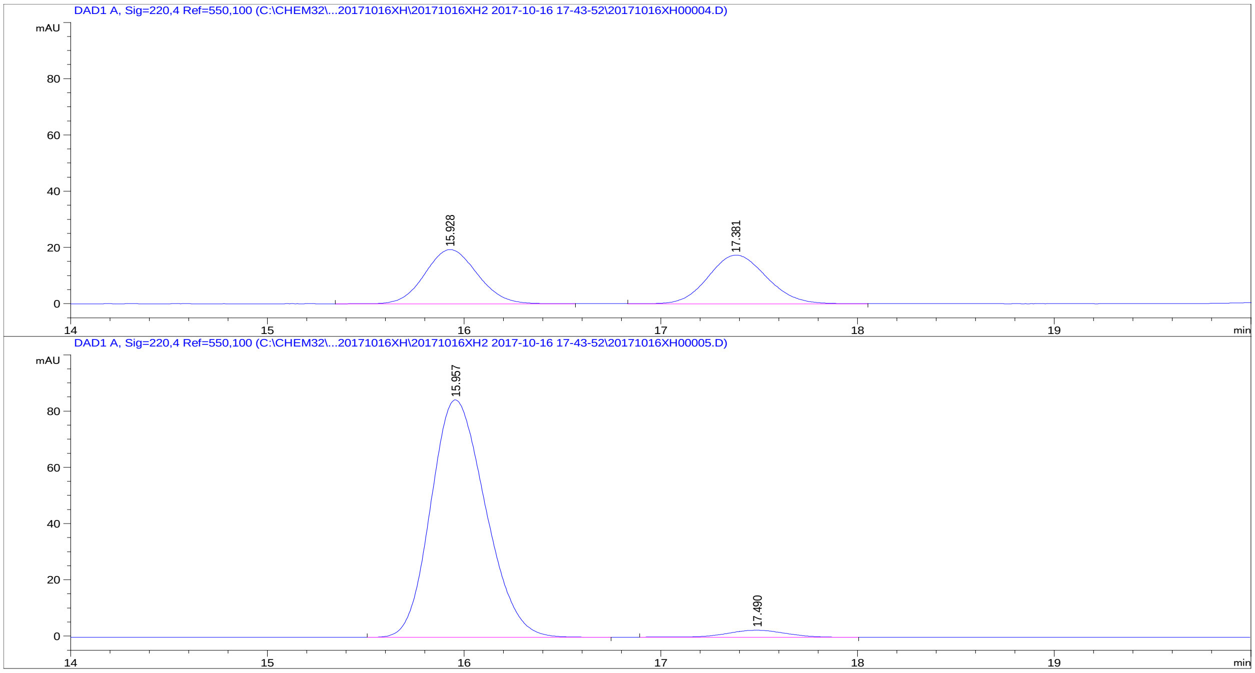

Integration results of 9:

Peak #	RetTime [min]	Type	Width [min]	Area [mAU*s]	Height [mAU]	Area %
1	15.957	BB	0.3032	1631.01648	84.48315	96.6808
2	17.490	VV	0.3230	55.99447	2.56233	3.3192

Integration results of racemic sample:

Peak #	RetTime [min]	Type	Width [min]	Area [mAU*s]	Height [mAU]	Area %
1	15.928	VB	0.2863	357.14005	19.26318	50.0190
2	17.381	VV	0.3224	356.86859	17.31737	49.9810
